# Dataset on the effect of carbon sources on the morphology and crystallite size of Fe/C composite microspheres prepared by the spray drying process

**DOI:** 10.1016/j.dib.2019.105052

**Published:** 2019-12-31

**Authors:** Sun Young Jeong, Jung Sang Cho

**Affiliations:** Department of Engineering Chemistry, Chungbuk National University, Chungbuk, 361-763, Republic of Korea

**Keywords:** Spray-drying, Fe/C composite, Microsphere, Phase, Crystallite size

## Abstract

The data presented in this manuscript showed the effect of the carbon sources on the morphology and crystallite size of Fe/C composite microspheres obtained after reduction of the as spray-dried powders. Each morphology, phase, and crystalline size of powders obtained after spray-drying and subsequent heat-treatment were investigated.

**Table:** Specifications Table

Subject	Chemistry
Specific subject area	Inorganic chemistry
Type of data	Figures
How data were acquired	FE-SEM (Hitachi S-4300), XRD (X'Pert PRO MPD)
Data format	Raw, analyzed data
Parameters for data collection	Carbon precursor, heat-treatment temperature
Description of data collection	Morphology, phase, crystallite size of Fe in Fe/C composite microspheres
Data source location	Cheongju, Chungbuk, Republic of Korea
Data accessibility	Data included in this article

**Table:** 

**Value of the Data** • These data provide a better understanding for the appropriate carbon sources and heat-treatment condition to obtain Fe/C composite microspheres with suitable crystallite size of Fe. • These data can be useful to those fabricate the Fe/C composite microspheres with suitable crystallite size of Fe. • These data can be applied for the synthesis of appropriate metal compound/C composite microspheres.

## 1 Data

**Fig. 1:**
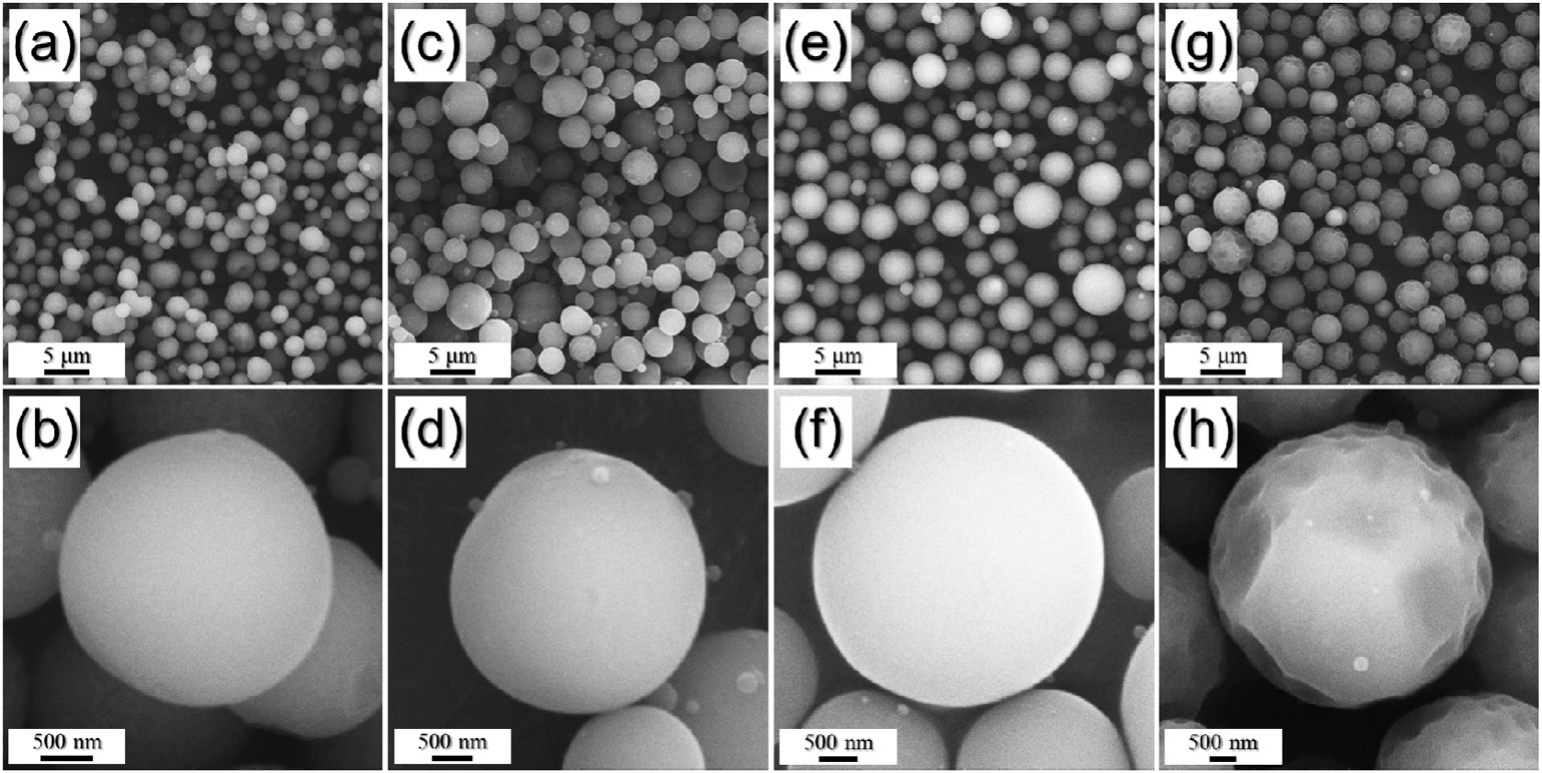
FE-SEM images of the precursor powders from the iron nitrate solution (a,b) without C precursors and with (c,d) citric acid, (e,f) sucrose, and (g,h) dextrin.

**Fig. 2:**
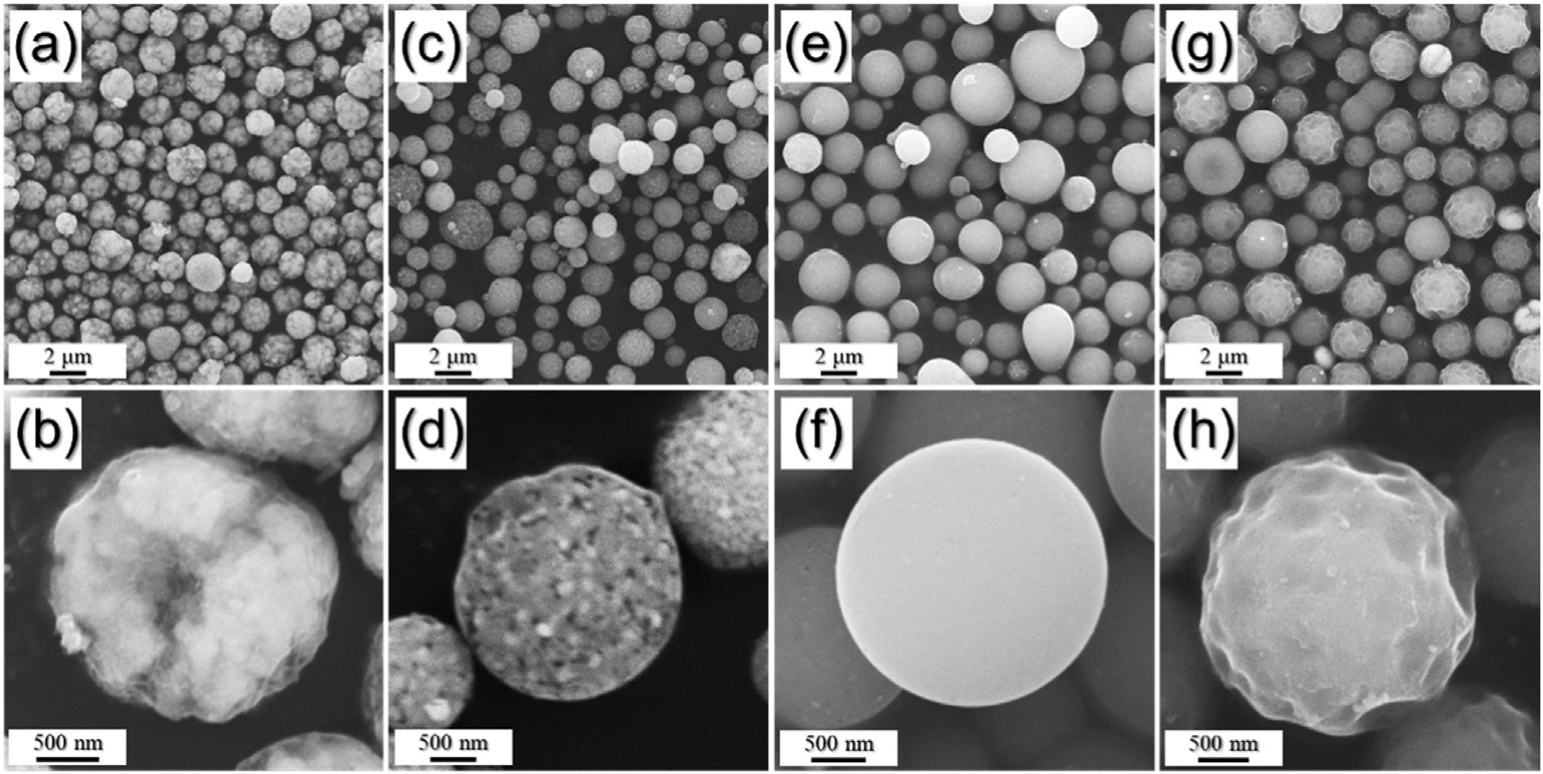
FE-SEM images of the powders obtained after heat-treatment of as-sprayed powders prepared from the solution (a,b) without C precursor and with (c,d) citric acid, (e,f) sucrose, and (g,h) dextrin, at 450 °C.

**Fig. 3:**
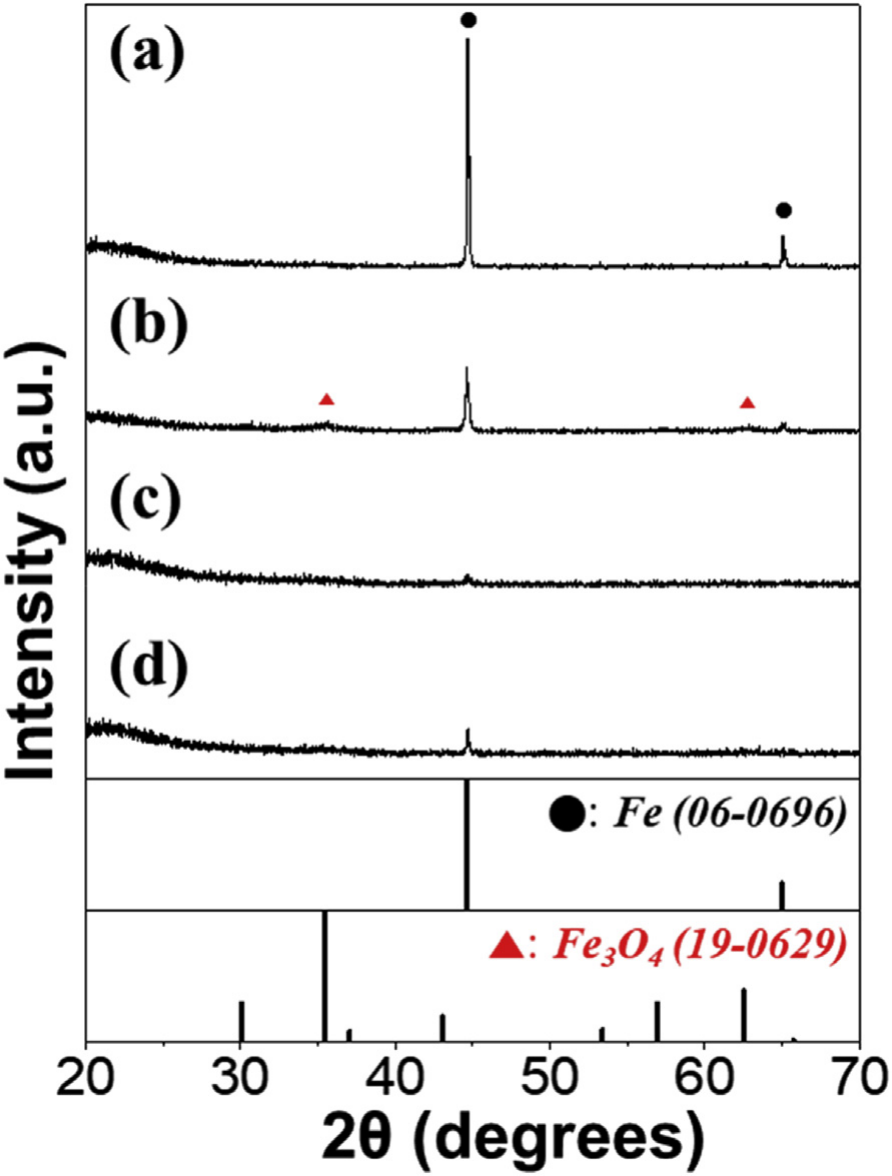
XRD patterns of the powders obtained after heat-treatment of as-sprayed powders prepared from the solution (a) without carbon precursors and with (b) citric acid, (c) sucrose, and (d) dextrin, at 450 °C.

**Fig. 4:**
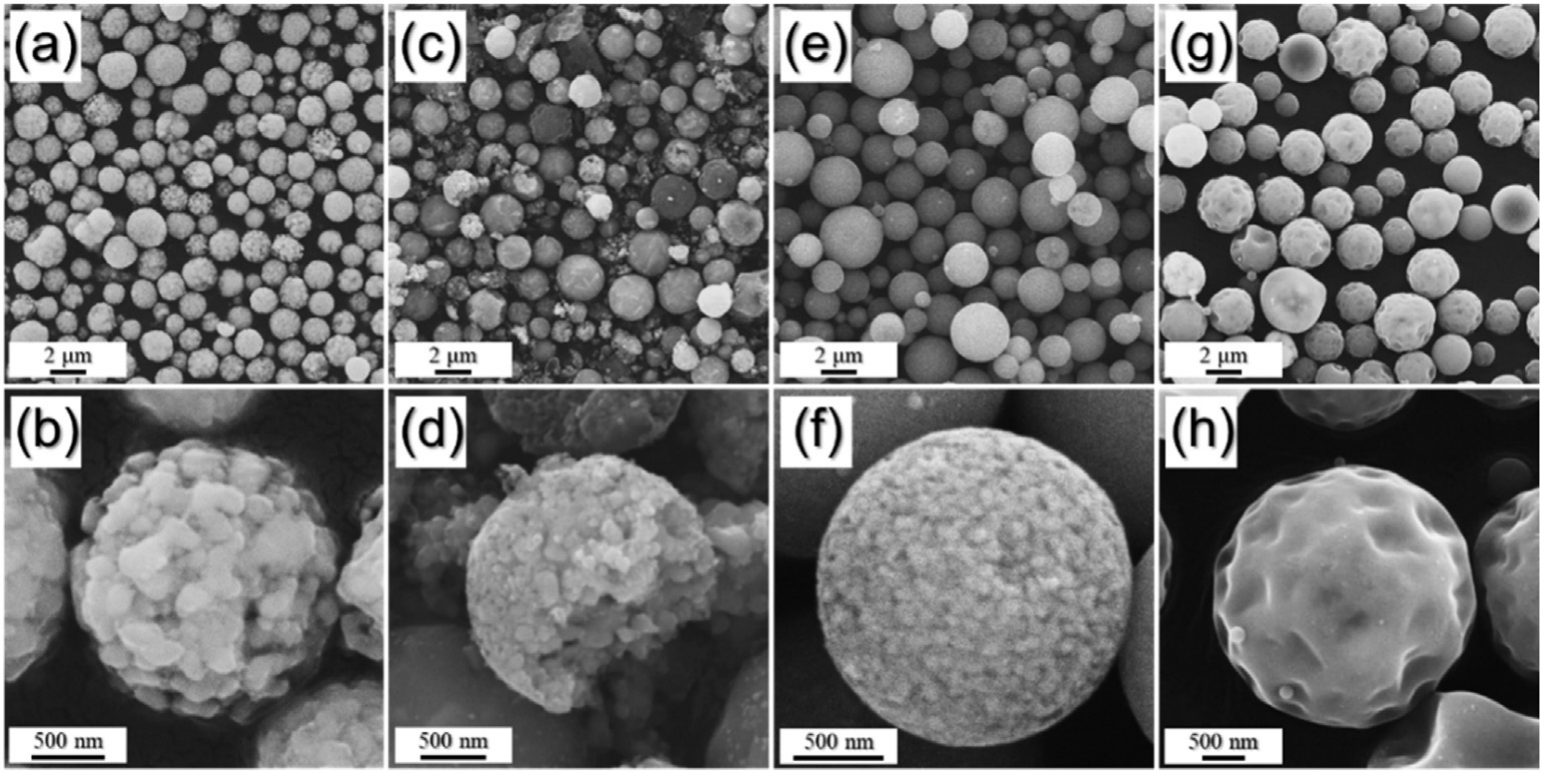
FE-SEM images of the powders obtained after heat-treatment of precursor powders prepared from the solution (a,b) without C precursor and with (c,d) citric acid, (e,f) sucrose, and (g,h) dextrin, at 500 °C.

**Fig. 5:**
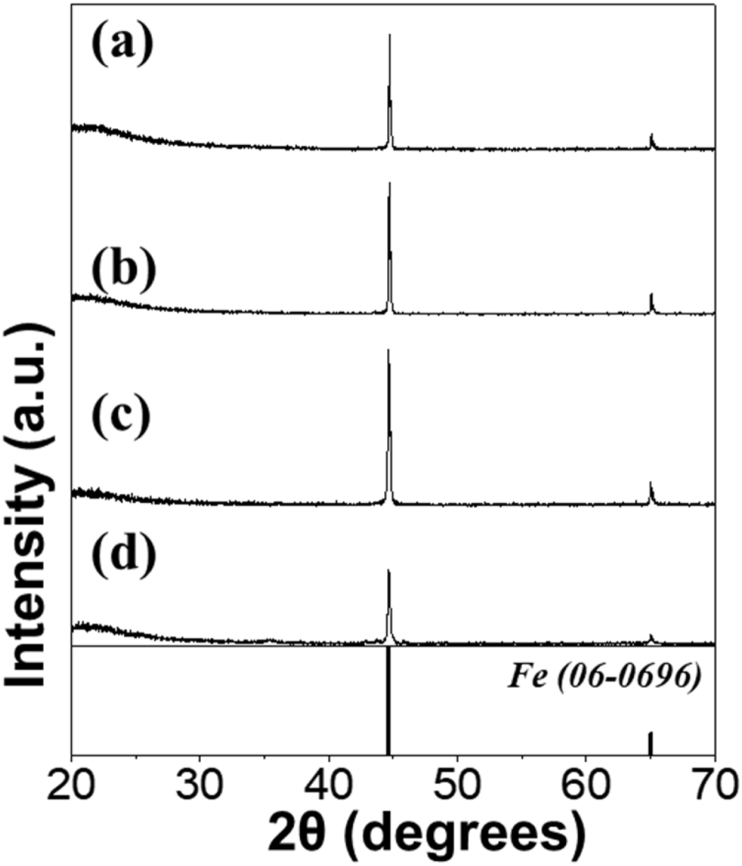
XRD patterns of the powders obtained after heat-treatment of as-sprayed powders prepared from the solution (a) without carbon precursors and with (b) citric acid, (c) sucrose, and (d) dextrin, at 500 °C.

The data exhibited in this manuscript include that showing the effect of the carbon source on the morphology, phase, and crystallite size of the Fe/C composite microspheres. Fig. 1 shows the as-sprayed microspheres obtained by spray drying process without carbon precursor and with citric acid, sucrose, and dextrin. Fig. 2, Fig. 4 exhibit morphologies of microspheres obtained after heat-treatment of as-sprayed powders at 450 °C and 500 °C. Fig. 3, Fig. 5 show XRD patterns of microspheres obtained after heat-treatment of as-sprayed powders at 450 °C and 500 °C. The raw data of XRD patterns could be shown in the supplemental file.

## 2 Experimental design, materials, and methods

Fe/C composite microspheres were prepared by spray drying process and subsequent heat-treatments for reduction. The spray-drying system applied in this data was described in detail in our previous literature [1]. For the synthesis of Fe/C composite microspheres, starting aqueous solution with 0.15 M iron nitrate nonahydrate (Fe(NO_3_)_3_∙9H_2_O, Sanchun, 98.5%) were prepared. Next, the various C precursors of 0.1 M citric acid monohydrate (C_6_H_10_O_8_, Junsei), sucrose (C_12_H_22_O_11_, Junsei), and dextrin [(C_6_H_10_O_5_)_n_, Samchun] were added into the above solutions, respectively. The inlet and outlet temperatures of the spray dryer were set to 300 and 120 °C, respectively. A two-fluid nozzle was used as an atomizer, and the atomization pressure was 2.4 bar. The obtained precursor powders were post heat-treated at 450 °C and 500 °C, respectively, under H_2_/Ar 10% gas atmosphere for 5 hours.

The FE-SEM images of the precursor powders obtained after spray drying were shown in Fig. 1. The samples exhibited spherical shape and had mean size of 3.4 μm, regardless of C precursors. However, the powders obtained from the solution with dextrin only showed wrinkled powder surface, as shown in Fig. 1g and h.

The precursor powders were then heat-treated at 450 °C for reduction, which were shown in Fig. 2. The powders obtained from the solutions with sucrose and dextrin maintained their original morphologies, as shown in Fig. 2e–h. However, the morphologies obtained from the solution without C precursor (Fig. 2a and b) and with citric acid (Fig. 2c and d) were changed from smooth to rough surface. It is because the powders obtained from the solutions without C precursor and with citric acid could not prevent the crystal growth of Fe metals during heat-treatment.

The phase of the microspheres obtained after heat-treatment at 450 °C were shown in Fig. 3. All samples exhibited the Fe metal phase (06-0696). However, a little amount of Fe_3_O_4_ phase (19-0629) was also detected along with Fe metal in the sample obtained from the solution with citric acid (Fig. 3b). By applying the Scherrer's equation to the (110) crystal plane of the cubic Fe peak, the mean crystallite sizes of Fe in Fe/C composite microspheres obtained from the solution without C precursor and with citric acid, sucrose, and dextrin were calculated to be 49, 30, 22, and 42 nm, respectively.

The morphologies of the microspheres obtained after heat-treatment of precursor powders at 500 °C were shown in Fig. 4. The powders obtained from the solution with dextrin still maintained their original morphologies, even after high treatment temperature of 500 °C, as shown in Fig. 4g and h. However, the grain growth of Fe metal embedded in C matrix was occurred in the samples obtained from the solution without C precursor (Fig. 2a and b) and with citric acid (Fig. 2c and d) and with sucrose (Fig. 2e and f).

The phase of the microspheres obtained after heat-treatment at 500 °C were shown in Fig. 5. All samples showed the pure Fe metal phase (06-0696) without Fe_3_O_4_. A little amount of Fe_3_O_4_ phase in the sample obtained from the solution with citric acid was transformed into Fe metal at 500 °C. By applying the Scherrer's equation to the (110) crystal plane of the cubic Fe peak, the mean crystallite sizes of Fe/C composite microspheres obtained from without C precursor and with citric acid, sucrose, and dextrin were calculated to be 52, 46, 39, and 36 nm, respectively.
