# Supplementary material for: Dataset on the effect of carbon sources on the morphology and crystallite size of Fe/C composite microspheres prepared by the spray drying process
Source: Data Brief. 2019 Dec 31;28:105052. doi: 10.1016/j.dib.2019.105052 (PMC6948126; doi:10.1016/j.dib.2019.105052)
Supplement: Multimedia component 1 [file mmc1.docx]

**Dataset on the effect of carbon sources on the morphology and crystallite size of Fe/C composite microspheres prepared by the spray drying process**

Sun Young Jeong and Jung Sang Cho*

Department of Engineering Chemistry, Chungbuk National University, Chungbuk, 361-763, Republic of Korea

*Corresponding authors.

E-mail: jscho@cbnu.ac.kr Tel.: +82-43-261-2489. Fax: +82-43-262-2380. (Jung Sang Cho)

**Raw data 1**. XRD raw data of the powder obtained after heat-treatment of as-sprayed powders prepared from the solution without carbon precursors at 450 ℃.

Goniometer RINT2000 vertical goniometer

Attachment Auto sample changer type B(6 samples)

Monochromater Fixed Monochromator

ScanningMode 2Theta/Theta

ScanningType Continuos Scanning

X-Ray 40kV/100mA

DivSlit 1 deg.

DivH.L.Slit 10mm

SctSlit 1 deg.

RecSlit 0.15mm

Monochro RS No Use

K-beta filter

Start 20

Stop 70

Step 0.01

20 433.333

20.01 333.333

20.02 408.333

20.03 433.333

20.04 441.667

20.05 366.667

20.06 366.667

20.07 475

20.08 350

20.09 475

20.1 350

20.11 383.333

20.12 375

20.13 425

20.14 416.667

20.15 391.667

20.16 433.333

20.17 491.667

20.18 433.333

20.19 491.667

20.2 375

20.21 350

20.22 358.333

20.23 500

20.24 291.667

20.25 341.667

20.26 375

20.27 475

20.28 383.333

20.29 608.333

20.3 433.333

20.31 416.667

20.32 425

20.33 350

20.34 333.333

20.35 383.333

20.36 366.667

20.37 400

20.38 525

20.39 408.333

20.4 441.667

20.41 550

20.42 450

20.43 358.333

20.44 541.667

20.45 450

20.46 325

20.47 475

20.48 408.333

20.49 508.333

20.5 425

20.51 525

20.52 366.667

20.53 466.667

20.54 358.333

20.55 416.667

20.56 450

20.57 425

20.58 425

20.59 541.667

20.6 450

20.61 391.667

20.62 650

20.63 333.333

20.64 433.333

20.65 400

20.66 391.667

20.67 466.667

20.68 466.667

20.69 416.667

20.7 441.667

20.71 358.333

20.72 375

20.73 400

20.74 475

20.75 383.333

20.76 383.333

20.77 433.333

20.78 466.667

20.79 433.333

20.8 508.333

20.81 500

20.82 425

20.83 483.333

20.84 425

20.85 441.667

20.86 516.667

20.87 383.333

20.88 450

20.89 466.667

20.9 391.667

20.91 400

20.92 458.333

20.93 516.667

20.94 383.333

20.95 350

20.96 491.667

20.97 375

20.98 400

20.99 391.667

21 491.667

21.01 375

21.02 475

21.03 433.333

21.04 475

21.05 400

21.06 433.333

21.07 400

21.08 450

21.09 400

21.1 500

21.11 475

21.12 408.333

21.13 375

21.14 483.333

21.15 350

21.16 483.333

21.17 458.333

21.18 308.333

21.19 500

21.2 433.333

21.21 375

21.22 333.333

21.23 491.667

21.24 350

21.25 450

21.26 458.333

21.27 400

21.28 375

21.29 441.667

21.3 416.667

21.31 475

21.32 491.667

21.33 450

21.34 408.333

21.35 416.667

21.36 350

21.37 383.333

21.38 441.667

21.39 408.333

21.4 508.333

21.41 391.667

21.42 375

21.43 558.333

21.44 300

21.45 433.333

21.46 483.333

21.47 408.333

21.48 441.667

21.49 483.333

21.5 433.333

21.51 491.667

21.52 375

21.53 500

21.54 458.333

21.55 441.667

21.56 500

21.57 383.333

21.58 383.333

21.59 516.667

21.6 391.667

21.61 400

21.62 375

21.63 516.667

21.64 408.333

21.65 491.667

21.66 450

21.67 475

21.68 441.667

21.69 450

21.7 450

21.71 258.333

21.72 316.667

21.73 383.333

21.74 491.667

21.75 391.667

21.76 383.333

21.77 533.333

21.78 433.333

21.79 416.667

21.8 325

21.81 441.667

21.82 516.667

21.83 316.667

21.84 366.667

21.85 441.667

21.86 316.667

21.87 391.667

21.88 366.667

21.89 350

21.9 433.333

21.91 516.667

21.92 333.333

21.93 391.667

21.94 425

21.95 341.667

21.96 441.667

21.97 408.333

21.98 300

21.99 425

22 325

22.01 391.667

22.02 366.667

22.03 383.333

22.04 316.667

22.05 500

22.06 391.667

22.07 350

22.08 383.333

22.09 375

22.1 408.333

22.11 408.333

22.12 366.667

22.13 383.333

22.14 441.667

22.15 341.667

22.16 358.333

22.17 433.333

22.18 433.333

22.19 433.333

22.2 225

22.21 441.667

22.22 383.333

22.23 366.667

22.24 458.333

22.25 375

22.26 350

22.27 408.333

22.28 391.667

22.29 350

22.3 416.667

22.31 425

22.32 275

22.33 316.667

22.34 316.667

22.35 325

22.36 466.667

22.37 441.667

22.38 408.333

22.39 450

22.4 433.333

22.41 350

22.42 458.333

22.43 375

22.44 333.333

22.45 458.333

22.46 350

22.47 483.333

22.48 400

22.49 433.333

22.5 375

22.51 358.333

22.52 450

22.53 341.667

22.54 358.333

22.55 358.333

22.56 416.667

22.57 308.333

22.58 491.667

22.59 391.667

22.6 308.333

22.61 383.333

22.62 333.333

22.63 366.667

22.64 308.333

22.65 425

22.66 441.667

22.67 241.667

22.68 341.667

22.69 433.333

22.7 308.333

22.71 466.667

22.72 425

22.73 358.333

22.74 350

22.75 300

22.76 525

22.77 333.333

22.78 441.667

22.79 400

22.8 308.333

22.81 383.333

22.82 491.667

22.83 375

22.84 383.333

22.85 366.667

22.86 366.667

22.87 375

22.88 308.333

22.89 366.667

22.9 275

22.91 325

22.92 383.333

22.93 316.667

22.94 508.333

22.95 358.333

22.96 308.333

22.97 383.333

22.98 316.667

22.99 441.667

23 283.333

23.01 316.667

23.02 333.333

23.03 383.333

23.04 441.667

23.05 375

23.06 266.667

23.07 341.667

23.08 341.667

23.09 341.667

23.1 258.333

23.11 391.667

23.12 333.333

23.13 341.667

23.14 383.333

23.15 408.333

23.16 408.333

23.17 258.333

23.18 333.333

23.19 441.667

23.2 358.333

23.21 366.667

23.22 308.333

23.23 283.333

23.24 308.333

23.25 325

23.26 375

23.27 308.333

23.28 408.333

23.29 325

23.3 341.667

23.31 325

23.32 358.333

23.33 275

23.34 341.667

23.35 391.667

23.36 316.667

23.37 383.333

23.38 433.333

23.39 316.667

23.4 408.333

23.41 291.667

23.42 291.667

23.43 391.667

23.44 341.667

23.45 333.333

23.46 341.667

23.47 233.333

23.48 366.667

23.49 308.333

23.5 375

23.51 275

23.52 450

23.53 441.667

23.54 300

23.55 333.333

23.56 291.667

23.57 366.667

23.58 366.667

23.59 266.667

23.6 316.667

23.61 225

23.62 266.667

23.63 366.667

23.64 425

23.65 258.333

23.66 266.667

23.67 283.333

23.68 241.667

23.69 350

23.7 366.667

23.71 208.333

23.72 233.333

23.73 241.667

23.74 275

23.75 275

23.76 375

23.77 308.333

23.78 208.333

23.79 350

23.8 250

23.81 316.667

23.82 291.667

23.83 425

23.84 300

23.85 316.667

23.86 350

23.87 316.667

23.88 408.333

23.89 275

23.9 316.667

23.91 300

23.92 366.667

23.93 225

23.94 300

23.95 233.333

23.96 416.667

23.97 316.667

23.98 200

23.99 283.333

24 391.667

24.01 283.333

24.02 358.333

24.03 258.333

24.04 358.333

24.05 341.667

24.06 233.333

24.07 275

24.08 291.667

24.09 266.667

24.1 375

24.11 283.333

24.12 241.667

24.13 300

24.14 241.667

24.15 266.667

24.16 233.333

24.17 341.667

24.18 266.667

24.19 325

24.2 208.333

24.21 258.333

24.22 300

24.23 325

24.24 241.667

24.25 308.333

24.26 250

24.27 308.333

24.28 275

24.29 341.667

24.3 225

24.31 266.667

24.32 216.667

24.33 241.667

24.34 325

24.35 275

24.36 316.667

24.37 258.333

24.38 333.333

24.39 191.667

24.4 241.667

24.41 291.667

24.42 291.667

24.43 308.333

24.44 183.333

24.45 241.667

24.46 341.667

24.47 241.667

24.48 233.333

24.49 316.667

24.5 275

24.51 225

24.52 258.333

24.53 241.667

24.54 233.333

24.55 233.333

24.56 250

24.57 216.667

24.58 200

24.59 191.667

24.6 266.667

24.61 133.333

24.62 300

24.63 258.333

24.64 241.667

24.65 358.333

24.66 250

24.67 250

24.68 216.667

24.69 258.333

24.7 291.667

24.71 291.667

24.72 208.333

24.73 258.333

24.74 225

24.75 191.667

24.76 283.333

24.77 183.333

24.78 258.333

24.79 283.333

24.8 175

24.81 166.667

24.82 283.333

24.83 175

24.84 183.333

24.85 258.333

24.86 225

24.87 333.333

24.88 233.333

24.89 250

24.9 141.667

24.91 241.667

24.92 150

24.93 300

24.94 308.333

24.95 241.667

24.96 208.333

24.97 258.333

24.98 225

24.99 233.333

25 250

25.01 200

25.02 250

25.03 225

25.04 283.333

25.05 300

25.06 175

25.07 225

25.08 233.333

25.09 158.333

25.1 150

25.11 183.333

25.12 200

25.13 291.667

25.14 258.333

25.15 216.667

25.16 208.333

25.17 300

25.18 266.667

25.19 241.667

25.2 150

25.21 175

25.22 225

25.23 133.333

25.24 191.667

25.25 225

25.26 283.333

25.27 241.667

25.28 200

25.29 208.333

25.3 175

25.31 191.667

25.32 266.667

25.33 216.667

25.34 233.333

25.35 183.333

25.36 208.333

25.37 283.333

25.38 275

25.39 250

25.4 225

25.41 225

25.42 250

25.43 175

25.44 250

25.45 183.333

25.46 225

25.47 183.333

25.48 158.333

25.49 233.333

25.5 191.667

25.51 183.333

25.52 250

25.53 158.333

25.54 225

25.55 341.667

25.56 200

25.57 208.333

25.58 233.333

25.59 241.667

25.6 208.333

25.61 208.333

25.62 225

25.63 225

25.64 241.667

25.65 216.667

25.66 275

25.67 250

25.68 108.333

25.69 208.333

25.7 200

25.71 150

25.72 208.333

25.73 183.333

25.74 183.333

25.75 308.333

25.76 216.667

25.77 150

25.78 200

25.79 183.333

25.8 241.667

25.81 216.667

25.82 216.667

25.83 158.333

25.84 158.333

25.85 233.333

25.86 175

25.87 125

25.88 250

25.89 275

25.9 166.667

25.91 233.333

25.92 241.667

25.93 175

25.94 233.333

25.95 275

25.96 166.667

25.97 283.333

25.98 241.667

25.99 266.667

26 250

26.01 200

26.02 125

26.03 166.667

26.04 225

26.05 200

26.06 158.333

26.07 225

26.08 200

26.09 191.667

26.1 150

26.11 225

26.12 125

26.13 191.667

26.14 241.667

26.15 175

26.16 233.333

26.17 150

26.18 216.667

26.19 200

26.2 158.333

26.21 225

26.22 191.667

26.23 158.333

26.24 133.333

26.25 166.667

26.26 108.333

26.27 158.333

26.28 200

26.29 175

26.3 133.333

26.31 150

26.32 216.667

26.33 225

26.34 133.333

26.35 216.667

26.36 150

26.37 208.333

26.38 150

26.39 216.667

26.4 150

26.41 183.333

26.42 125

26.43 175

26.44 200

26.45 233.333

26.46 183.333

26.47 150

26.48 191.667

26.49 175

26.5 200

26.51 191.667

26.52 225

26.53 141.667

26.54 108.333

26.55 133.333

26.56 158.333

26.57 175

26.58 175

26.59 183.333

26.6 216.667

26.61 183.333

26.62 183.333

26.63 200

26.64 158.333

26.65 233.333

26.66 108.333

26.67 175

26.68 183.333

26.69 216.667

26.7 141.667

26.71 116.667

26.72 133.333

26.73 175

26.74 183.333

26.75 150

26.76 200

26.77 200

26.78 133.333

26.79 175

26.8 91.6667

26.81 166.667

26.82 175

26.83 100

26.84 133.333

26.85 150

26.86 141.667

26.87 200

26.88 83.3333

26.89 225

26.9 133.333

26.91 183.333

26.92 158.333

26.93 191.667

26.94 200

26.95 200

26.96 233.333

26.97 125

26.98 125

26.99 158.333

27 141.667

27.01 200

27.02 141.667

27.03 100

27.04 141.667

27.05 141.667

27.06 166.667

27.07 150

27.08 183.333

27.09 175

27.1 150

27.11 125

27.12 141.667

27.13 91.6667

27.14 125

27.15 166.667

27.16 208.333

27.17 158.333

27.18 100

27.19 150

27.2 200

27.21 116.667

27.22 141.667

27.23 91.6667

27.24 125

27.25 141.667

27.26 133.333

27.27 166.667

27.28 175

27.29 166.667

27.3 150

27.31 133.333

27.32 100

27.33 108.333

27.34 133.333

27.35 191.667

27.36 116.667

27.37 100

27.38 133.333

27.39 183.333

27.4 133.333

27.41 125

27.42 150

27.43 133.333

27.44 158.333

27.45 208.333

27.46 166.667

27.47 133.333

27.48 125

27.49 191.667

27.5 141.667

27.51 200

27.52 116.667

27.53 133.333

27.54 141.667

27.55 125

27.56 83.3333

27.57 191.667

27.58 166.667

27.59 166.667

27.6 241.667

27.61 166.667

27.62 183.333

27.63 116.667

27.64 175

27.65 150

27.66 150

27.67 150

27.68 166.667

27.69 150

27.7 158.333

27.71 175

27.72 125

27.73 83.3333

27.74 133.333

27.75 200

27.76 191.667

27.77 208.333

27.78 191.667

27.79 141.667

27.8 100

27.81 166.667

27.82 150

27.83 150

27.84 100

27.85 108.333

27.86 150

27.87 133.333

27.88 100

27.89 133.333

27.9 133.333

27.91 191.667

27.92 83.3333

27.93 66.6667

27.94 100

27.95 166.667

27.96 116.667

27.97 133.333

27.98 133.333

27.99 125

28 133.333

28.01 58.3333

28.02 175

28.03 166.667

28.04 116.667

28.05 216.667

28.06 133.333

28.07 141.667

28.08 141.667

28.09 125

28.1 91.6667

28.11 100

28.12 191.667

28.13 166.667

28.14 125

28.15 141.667

28.16 183.333

28.17 158.333

28.18 175

28.19 158.333

28.2 175

28.21 116.667

28.22 91.6667

28.23 116.667

28.24 200

28.25 141.667

28.26 50

28.27 91.6667

28.28 116.667

28.29 141.667

28.3 100

28.31 150

28.32 158.333

28.33 150

28.34 83.3333

28.35 108.333

28.36 166.667

28.37 100

28.38 125

28.39 125

28.4 158.333

28.41 116.667

28.42 158.333

28.43 125

28.44 100

28.45 108.333

28.46 141.667

28.47 100

28.48 133.333

28.49 150

28.5 100

28.51 100

28.52 141.667

28.53 100

28.54 108.333

28.55 158.333

28.56 108.333

28.57 75

28.58 166.667

28.59 91.6667

28.6 141.667

28.61 141.667

28.62 150

28.63 166.667

28.64 208.333

28.65 166.667

28.66 133.333

28.67 175

28.68 91.6667

28.69 166.667

28.7 133.333

28.71 100

28.72 83.3333

28.73 125

28.74 116.667

28.75 150

28.76 83.3333

28.77 91.6667

28.78 116.667

28.79 133.333

28.8 150

28.81 158.333

28.82 116.667

28.83 133.333

28.84 91.6667

28.85 116.667

28.86 108.333

28.87 150

28.88 116.667

28.89 75

28.9 141.667

28.91 116.667

28.92 125

28.93 125

28.94 116.667

28.95 66.6667

28.96 100

28.97 141.667

28.98 125

28.99 83.3333

29 108.333

29.01 66.6667

29.02 158.333

29.03 125

29.04 141.667

29.05 166.667

29.06 116.667

29.07 108.333

29.08 141.667

29.09 141.667

29.1 83.3333

29.11 125

29.12 100

29.13 116.667

29.14 175

29.15 141.667

29.16 116.667

29.17 116.667

29.18 100

29.19 66.6667

29.2 133.333

29.21 133.333

29.22 158.333

29.23 83.3333

29.24 133.333

29.25 150

29.26 116.667

29.27 125

29.28 133.333

29.29 108.333

29.3 91.6667

29.31 91.6667

29.32 150

29.33 83.3333

29.34 141.667

29.35 158.333

29.36 150

29.37 191.667

29.38 175

29.39 83.3333

29.4 125

29.41 150

29.42 66.6667

29.43 150

29.44 166.667

29.45 133.333

29.46 91.6667

29.47 133.333

29.48 66.6667

29.49 141.667

29.5 108.333

29.51 116.667

29.52 100

29.53 175

29.54 141.667

29.55 125

29.56 150

29.57 100

29.58 100

29.59 116.667

29.6 108.333

29.61 83.3333

29.62 133.333

29.63 75

29.64 125

29.65 141.667

29.66 125

29.67 125

29.68 75

29.69 108.333

29.7 150

29.71 108.333

29.72 175

29.73 141.667

29.74 100

29.75 66.6667

29.76 91.6667

29.77 66.6667

29.78 50

29.79 66.6667

29.8 150

29.81 116.667

29.82 83.3333

29.83 175

29.84 116.667

29.85 83.3333

29.86 133.333

29.87 91.6667

29.88 66.6667

29.89 66.6667

29.9 108.333

29.91 58.3333

29.92 133.333

29.93 83.3333

29.94 100

29.95 108.333

29.96 116.667

29.97 100

29.98 91.6667

29.99 91.6667

30 116.667

30.01 116.667

30.02 91.6667

30.03 100

30.04 141.667

30.05 141.667

30.06 125

30.07 66.6667

30.08 83.3333

30.09 83.3333

30.1 133.333

30.11 100

30.12 66.6667

30.13 100

30.14 141.667

30.15 108.333

30.16 141.667

30.17 125

30.18 66.6667

30.19 91.6667

30.2 125

30.21 91.6667

30.22 108.333

30.23 116.667

30.24 158.333

30.25 83.3333

30.26 141.667

30.27 175

30.28 116.667

30.29 75

30.3 116.667

30.31 100

30.32 58.3333

30.33 91.6667

30.34 91.6667

30.35 83.3333

30.36 116.667

30.37 91.6667

30.38 141.667

30.39 83.3333

30.4 108.333

30.41 75

30.42 66.6667

30.43 66.6667

30.44 166.667

30.45 100

30.46 66.6667

30.47 75

30.48 125

30.49 141.667

30.5 75

30.51 125

30.52 141.667

30.53 83.3333

30.54 108.333

30.55 116.667

30.56 75

30.57 100

30.58 83.3333

30.59 116.667

30.6 91.6667

30.61 150

30.62 125

30.63 100

30.64 166.667

30.65 141.667

30.66 100

30.67 83.3333

30.68 100

30.69 100

30.7 83.3333

30.71 91.6667

30.72 158.333

30.73 91.6667

30.74 100

30.75 125

30.76 150

30.77 100

30.78 158.333

30.79 75

30.8 166.667

30.81 75

30.82 183.333

30.83 125

30.84 108.333

30.85 100

30.86 83.3333

30.87 83.3333

30.88 58.3333

30.89 133.333

30.9 75

30.91 66.6667

30.92 125

30.93 50

30.94 125

30.95 41.6667

30.96 66.6667

30.97 75

30.98 83.3333

30.99 125

31 75

31.01 75

31.02 91.6667

31.03 133.333

31.04 91.6667

31.05 91.6667

31.06 91.6667

31.07 100

31.08 116.667

31.09 75

31.1 41.6667

31.11 125

31.12 108.333

31.13 150

31.14 133.333

31.15 75

31.16 83.3333

31.17 91.6667

31.18 125

31.19 91.6667

31.2 91.6667

31.21 133.333

31.22 83.3333

31.23 83.3333

31.24 183.333

31.25 83.3333

31.26 100

31.27 116.667

31.28 83.3333

31.29 66.6667

31.3 83.3333

31.31 83.3333

31.32 75

31.33 66.6667

31.34 158.333

31.35 66.6667

31.36 116.667

31.37 100

31.38 58.3333

31.39 75

31.4 50

31.41 58.3333

31.42 75

31.43 83.3333

31.44 100

31.45 116.667

31.46 91.6667

31.47 75

31.48 108.333

31.49 75

31.5 83.3333

31.51 83.3333

31.52 91.6667

31.53 108.333

31.54 75

31.55 83.3333

31.56 66.6667

31.57 125

31.58 83.3333

31.59 108.333

31.6 150

31.61 133.333

31.62 125

31.63 83.3333

31.64 91.6667

31.65 116.667

31.66 75

31.67 133.333

31.68 75

31.69 66.6667

31.7 108.333

31.71 116.667

31.72 133.333

31.73 75

31.74 150

31.75 100

31.76 91.6667

31.77 66.6667

31.78 125

31.79 108.333

31.8 133.333

31.81 83.3333

31.82 66.6667

31.83 66.6667

31.84 66.6667

31.85 150

31.86 108.333

31.87 125

31.88 83.3333

31.89 75

31.9 116.667

31.91 133.333

31.92 91.6667

31.93 125

31.94 125

31.95 66.6667

31.96 75

31.97 166.667

31.98 50

31.99 133.333

32 91.6667

32.01 66.6667

32.02 91.6667

32.03 141.667

32.04 75

32.05 66.6667

32.06 100

32.07 125

32.08 91.6667

32.09 83.3333

32.1 108.333

32.11 100

32.12 66.6667

32.13 66.6667

32.14 133.333

32.15 125

32.16 125

32.17 100

32.18 108.333

32.19 66.6667

32.2 100

32.21 83.3333

32.22 33.3333

32.23 66.6667

32.24 91.6667

32.25 75

32.26 75

32.27 75

32.28 83.3333

32.29 58.3333

32.3 66.6667

32.31 116.667

32.32 100

32.33 100

32.34 66.6667

32.35 75

32.36 91.6667

32.37 58.3333

32.38 133.333

32.39 58.3333

32.4 91.6667

32.41 91.6667

32.42 133.333

32.43 50

32.44 91.6667

32.45 141.667

32.46 50

32.47 58.3333

32.48 58.3333

32.49 50

32.5 116.667

32.51 41.6667

32.52 116.667

32.53 83.3333

32.54 58.3333

32.55 50

32.56 50

32.57 75

32.58 125

32.59 83.3333

32.6 116.667

32.61 91.6667

32.62 75

32.63 150

32.64 50

32.65 50

32.66 58.3333

32.67 83.3333

32.68 150

32.69 100

32.7 125

32.71 116.667

32.72 83.3333

32.73 41.6667

32.74 75

32.75 91.6667

32.76 75

32.77 41.6667

32.78 58.3333

32.79 75

32.8 75

32.81 83.3333

32.82 66.6667

32.83 33.3333

32.84 83.3333

32.85 100

32.86 75

32.87 100

32.88 66.6667

32.89 91.6667

32.9 100

32.91 66.6667

32.92 58.3333

32.93 158.333

32.94 108.333

32.95 66.6667

32.96 75

32.97 116.667

32.98 66.6667

32.99 100

33 58.3333

33.01 100

33.02 91.6667

33.03 108.333

33.04 108.333

33.05 50

33.06 75

33.07 75

33.08 83.3333

33.09 58.3333

33.1 75

33.11 83.3333

33.12 100

33.13 58.3333

33.14 75

33.15 66.6667

33.16 108.333

33.17 58.3333

33.18 91.6667

33.19 91.6667

33.2 50

33.21 108.333

33.22 66.6667

33.23 100

33.24 83.3333

33.25 58.3333

33.26 83.3333

33.27 50

33.28 100

33.29 41.6667

33.3 66.6667

33.31 91.6667

33.32 58.3333

33.33 83.3333

33.34 50

33.35 41.6667

33.36 83.3333

33.37 91.6667

33.38 83.3333

33.39 75

33.4 50

33.41 91.6667

33.42 91.6667

33.43 133.333

33.44 91.6667

33.45 58.3333

33.46 66.6667

33.47 108.333

33.48 50

33.49 58.3333

33.5 58.3333

33.51 100

33.52 100

33.53 75

33.54 83.3333

33.55 158.333

33.56 58.3333

33.57 100

33.58 150

33.59 91.6667

33.6 91.6667

33.61 75

33.62 58.3333

33.63 108.333

33.64 66.6667

33.65 58.3333

33.66 66.6667

33.67 75

33.68 66.6667

33.69 33.3333

33.7 33.3333

33.71 83.3333

33.72 83.3333

33.73 66.6667

33.74 91.6667

33.75 58.3333

33.76 83.3333

33.77 75

33.78 83.3333

33.79 91.6667

33.8 100

33.81 100

33.82 50

33.83 108.333

33.84 125

33.85 133.333

33.86 91.6667

33.87 116.667

33.88 91.6667

33.89 91.6667

33.9 100

33.91 66.6667

33.92 50

33.93 125

33.94 41.6667

33.95 75

33.96 66.6667

33.97 100

33.98 66.6667

33.99 108.333

34 75

34.01 75

34.02 108.333

34.03 58.3333

34.04 108.333

34.05 58.3333

34.06 66.6667

34.07 83.3333

34.08 108.333

34.09 33.3333

34.1 100

34.11 58.3333

34.12 41.6667

34.13 125

34.14 158.333

34.15 66.6667

34.16 66.6667

34.17 133.333

34.18 100

34.19 66.6667

34.2 50

34.21 116.667

34.22 83.3333

34.23 33.3333

34.24 58.3333

34.25 83.3333

34.26 75

34.27 108.333

34.28 50

34.29 66.6667

34.3 83.3333

34.31 58.3333

34.32 116.667

34.33 58.3333

34.34 66.6667

34.35 75

34.36 91.6667

34.37 133.333

34.38 66.6667

34.39 91.6667

34.4 83.3333

34.41 58.3333

34.42 25

34.43 50

34.44 66.6667

34.45 50

34.46 75

34.47 83.3333

34.48 75

34.49 108.333

34.5 108.333

34.51 100

34.52 58.3333

34.53 83.3333

34.54 83.3333

34.55 66.6667

34.56 75

34.57 41.6667

34.58 58.3333

34.59 75

34.6 75

34.61 75

34.62 50

34.63 83.3333

34.64 50

34.65 66.6667

34.66 133.333

34.67 58.3333

34.68 75

34.69 58.3333

34.7 33.3333

34.71 83.3333

34.72 41.6667

34.73 83.3333

34.74 83.3333

34.75 66.6667

34.76 83.3333

34.77 75

34.78 158.333

34.79 66.6667

34.8 116.667

34.81 91.6667

34.82 100

34.83 91.6667

34.84 100

34.85 91.6667

34.86 100

34.87 50

34.88 58.3333

34.89 91.6667

34.9 91.6667

34.91 75

34.92 83.3333

34.93 41.6667

34.94 91.6667

34.95 83.3333

34.96 91.6667

34.97 91.6667

34.98 100

34.99 83.3333

35 58.3333

35.01 83.3333

35.02 83.3333

35.03 50

35.04 50

35.05 108.333

35.06 41.6667

35.07 58.3333

35.08 83.3333

35.09 50

35.1 100

35.11 58.3333

35.12 58.3333

35.13 66.6667

35.14 58.3333

35.15 91.6667

35.16 83.3333

35.17 41.6667

35.18 41.6667

35.19 58.3333

35.2 75

35.21 75

35.22 91.6667

35.23 91.6667

35.24 83.3333

35.25 83.3333

35.26 66.6667

35.27 66.6667

35.28 83.3333

35.29 83.3333

35.3 116.667

35.31 133.333

35.32 58.3333

35.33 58.3333

35.34 108.333

35.35 75

35.36 50

35.37 75

35.38 33.3333

35.39 100

35.4 50

35.41 58.3333

35.42 58.3333

35.43 91.6667

35.44 50

35.45 66.6667

35.46 91.6667

35.47 75

35.48 41.6667

35.49 100

35.5 66.6667

35.51 75

35.52 91.6667

35.53 66.6667

35.54 50

35.55 58.3333

35.56 75

35.57 133.333

35.58 116.667

35.59 100

35.6 116.667

35.61 58.3333

35.62 75

35.63 75

35.64 41.6667

35.65 25

35.66 75

35.67 100

35.68 66.6667

35.69 66.6667

35.7 66.6667

35.71 66.6667

35.72 75

35.73 58.3333

35.74 66.6667

35.75 91.6667

35.76 66.6667

35.77 66.6667

35.78 75

35.79 33.3333

35.8 91.6667

35.81 25

35.82 91.6667

35.83 91.6667

35.84 33.3333

35.85 33.3333

35.86 41.6667

35.87 108.333

35.88 125

35.89 66.6667

35.9 108.333

35.91 116.667

35.92 75

35.93 75

35.94 66.6667

35.95 58.3333

35.96 108.333

35.97 100

35.98 41.6667

35.99 83.3333

36 108.333

36.01 66.6667

36.02 58.3333

36.03 41.6667

36.04 75

36.05 66.6667

36.06 116.667

36.07 41.6667

36.08 50

36.09 50

36.1 83.3333

36.11 41.6667

36.12 33.3333

36.13 100

36.14 50

36.15 75

36.16 58.3333

36.17 75

36.18 58.3333

36.19 8.33333

36.2 58.3333

36.21 58.3333

36.22 50

36.23 116.667

36.24 108.333

36.25 66.6667

36.26 83.3333

36.27 41.6667

36.28 50

36.29 108.333

36.3 83.3333

36.31 91.6667

36.32 83.3333

36.33 50

36.34 66.6667

36.35 75

36.36 58.3333

36.37 66.6667

36.38 50

36.39 41.6667

36.4 66.6667

36.41 91.6667

36.42 83.3333

36.43 108.333

36.44 83.3333

36.45 75

36.46 41.6667

36.47 75

36.48 41.6667

36.49 75

36.5 66.6667

36.51 100

36.52 116.667

36.53 83.3333

36.54 66.6667

36.55 66.6667

36.56 50

36.57 33.3333

36.58 91.6667

36.59 58.3333

36.6 66.6667

36.61 50

36.62 50

36.63 100

36.64 66.6667

36.65 33.3333

36.66 66.6667

36.67 25

36.68 50

36.69 75

36.7 108.333

36.71 66.6667

36.72 25

36.73 50

36.74 66.6667

36.75 75

36.76 41.6667

36.77 58.3333

36.78 41.6667

36.79 108.333

36.8 25

36.81 16.6667

36.82 75

36.83 50

36.84 66.6667

36.85 108.333

36.86 91.6667

36.87 50

36.88 75

36.89 8.33333

36.9 66.6667

36.91 66.6667

36.92 25

36.93 50

36.94 50

36.95 41.6667

36.96 66.6667

36.97 50

36.98 83.3333

36.99 66.6667

37 25

37.01 58.3333

37.02 50

37.03 83.3333

37.04 25

37.05 50

37.06 66.6667

37.07 58.3333

37.08 66.6667

37.09 41.6667

37.1 58.3333

37.11 8.33333

37.12 41.6667

37.13 83.3333

37.14 108.333

37.15 66.6667

37.16 58.3333

37.17 25

37.18 33.3333

37.19 58.3333

37.2 58.3333

37.21 50

37.22 75

37.23 75

37.24 66.6667

37.25 75

37.26 108.333

37.27 83.3333

37.28 108.333

37.29 41.6667

37.3 16.6667

37.31 75

37.32 83.3333

37.33 108.333

37.34 50

37.35 33.3333

37.36 116.667

37.37 75

37.38 91.6667

37.39 41.6667

37.4 33.3333

37.41 50

37.42 58.3333

37.43 83.3333

37.44 41.6667

37.45 33.3333

37.46 58.3333

37.47 33.3333

37.48 33.3333

37.49 41.6667

37.5 41.6667

37.51 83.3333

37.52 83.3333

37.53 66.6667

37.54 66.6667

37.55 50

37.56 83.3333

37.57 58.3333

37.58 75

37.59 41.6667

37.6 58.3333

37.61 58.3333

37.62 66.6667

37.63 75

37.64 50

37.65 58.3333

37.66 75

37.67 58.3333

37.68 83.3333

37.69 58.3333

37.7 50

37.71 66.6667

37.72 58.3333

37.73 66.6667

37.74 66.6667

37.75 66.6667

37.76 50

37.77 75

37.78 66.6667

37.79 100

37.8 33.3333

37.81 58.3333

37.82 75

37.83 66.6667

37.84 50

37.85 100

37.86 41.6667

37.87 116.667

37.88 33.3333

37.89 58.3333

37.9 50

37.91 41.6667

37.92 50

37.93 75

37.94 25

37.95 75

37.96 33.3333

37.97 33.3333

37.98 50

37.99 66.6667

38 58.3333

38.01 58.3333

38.02 41.6667

38.03 33.3333

38.04 66.6667

38.05 66.6667

38.06 66.6667

38.07 83.3333

38.08 58.3333

38.09 141.667

38.1 50

38.11 41.6667

38.12 41.6667

38.13 75

38.14 75

38.15 66.6667

38.16 58.3333

38.17 83.3333

38.18 75

38.19 25

38.2 50

38.21 58.3333

38.22 66.6667

38.23 75

38.24 75

38.25 58.3333

38.26 58.3333

38.27 25

38.28 50

38.29 25

38.3 75

38.31 83.3333

38.32 58.3333

38.33 50

38.34 75

38.35 33.3333

38.36 58.3333

38.37 83.3333

38.38 75

38.39 33.3333

38.4 83.3333

38.41 33.3333

38.42 83.3333

38.43 33.3333

38.44 83.3333

38.45 41.6667

38.46 75

38.47 58.3333

38.48 66.6667

38.49 108.333

38.5 66.6667

38.51 100

38.52 100

38.53 33.3333

38.54 75

38.55 33.3333

38.56 75

38.57 75

38.58 58.3333

38.59 58.3333

38.6 75

38.61 33.3333

38.62 75

38.63 83.3333

38.64 75

38.65 41.6667

38.66 83.3333

38.67 58.3333

38.68 16.6667

38.69 50

38.7 83.3333

38.71 50

38.72 58.3333

38.73 41.6667

38.74 33.3333

38.75 25

38.76 83.3333

38.77 66.6667

38.78 83.3333

38.79 91.6667

38.8 75

38.81 50

38.82 75

38.83 16.6667

38.84 75

38.85 41.6667

38.86 25

38.87 83.3333

38.88 41.6667

38.89 33.3333

38.9 75

38.91 75

38.92 75

38.93 50

38.94 58.3333

38.95 41.6667

38.96 58.3333

38.97 58.3333

38.98 33.3333

38.99 58.3333

39 66.6667

39.01 25

39.02 33.3333

39.03 33.3333

39.04 41.6667

39.05 58.3333

39.06 16.6667

39.07 50

39.08 41.6667

39.09 33.3333

39.1 50

39.11 33.3333

39.12 33.3333

39.13 58.3333

39.14 75

39.15 16.6667

39.16 66.6667

39.17 25

39.18 58.3333

39.19 16.6667

39.2 75

39.21 41.6667

39.22 50

39.23 33.3333

39.24 75

39.25 83.3333

39.26 16.6667

39.27 50

39.28 16.6667

39.29 50

39.3 91.6667

39.31 50

39.32 25

39.33 16.6667

39.34 83.3333

39.35 50

39.36 75

39.37 50

39.38 41.6667

39.39 50

39.4 83.3333

39.41 66.6667

39.42 100

39.43 41.6667

39.44 66.6667

39.45 50

39.46 50

39.47 41.6667

39.48 41.6667

39.49 41.6667

39.5 33.3333

39.51 83.3333

39.52 75

39.53 33.3333

39.54 33.3333

39.55 108.333

39.56 83.3333

39.57 41.6667

39.58 50

39.59 83.3333

39.6 33.3333

39.61 33.3333

39.62 91.6667

39.63 83.3333

39.64 58.3333

39.65 25

39.66 50

39.67 100

39.68 25

39.69 16.6667

39.7 75

39.71 66.6667

39.72 50

39.73 66.6667

39.74 58.3333

39.75 41.6667

39.76 41.6667

39.77 75

39.78 58.3333

39.79 66.6667

39.8 100

39.81 66.6667

39.82 58.3333

39.83 100

39.84 25

39.85 41.6667

39.86 16.6667

39.87 33.3333

39.88 50

39.89 50

39.9 66.6667

39.91 58.3333

39.92 41.6667

39.93 58.3333

39.94 83.3333

39.95 41.6667

39.96 58.3333

39.97 58.3333

39.98 58.3333

39.99 41.6667

40 66.6667

40.01 58.3333

40.02 16.6667

40.03 50

40.04 41.6667

40.05 25

40.06 41.6667

40.07 33.3333

40.08 58.3333

40.09 50

40.1 100

40.11 83.3333

40.12 33.3333

40.13 66.6667

40.14 41.6667

40.15 41.6667

40.16 41.6667

40.17 25

40.18 50

40.19 41.6667

40.2 83.3333

40.21 66.6667

40.22 83.3333

40.23 50

40.24 91.6667

40.25 91.6667

40.26 50

40.27 75

40.28 58.3333

40.29 58.3333

40.3 58.3333

40.31 75

40.32 58.3333

40.33 50

40.34 66.6667

40.35 16.6667

40.36 16.6667

40.37 66.6667

40.38 25

40.39 25

40.4 50

40.41 58.3333

40.42 33.3333

40.43 25

40.44 58.3333

40.45 75

40.46 75

40.47 25

40.48 50

40.49 66.6667

40.5 25

40.51 16.6667

40.52 66.6667

40.53 41.6667

40.54 58.3333

40.55 41.6667

40.56 58.3333

40.57 8.33333

40.58 50

40.59 41.6667

40.6 25

40.61 58.3333

40.62 50

40.63 50

40.64 50

40.65 58.3333

40.66 50

40.67 50

40.68 33.3333

40.69 33.3333

40.7 41.6667

40.71 75

40.72 41.6667

40.73 41.6667

40.74 100

40.75 66.6667

40.76 50

40.77 33.3333

40.78 58.3333

40.79 33.3333

40.8 50

40.81 75

40.82 75

40.83 50

40.84 58.3333

40.85 16.6667

40.86 25

40.87 50

40.88 41.6667

40.89 50

40.9 66.6667

40.91 41.6667

40.92 83.3333

40.93 58.3333

40.94 8.33333

40.95 25

40.96 58.3333

40.97 91.6667

40.98 58.3333

40.99 25

41 16.6667

41.01 41.6667

41.02 66.6667

41.03 66.6667

41.04 58.3333

41.05 58.3333

41.06 66.6667

41.07 16.6667

41.08 41.6667

41.09 58.3333

41.1 41.6667

41.11 41.6667

41.12 50

41.13 50

41.14 66.6667

41.15 41.6667

41.16 25

41.17 25

41.18 41.6667

41.19 33.3333

41.2 33.3333

41.21 41.6667

41.22 50

41.23 50

41.24 41.6667

41.25 125

41.26 41.6667

41.27 33.3333

41.28 66.6667

41.29 50

41.3 75

41.31 25

41.32 16.6667

41.33 66.6667

41.34 25

41.35 58.3333

41.36 33.3333

41.37 41.6667

41.38 58.3333

41.39 41.6667

41.4 25

41.41 33.3333

41.42 66.6667

41.43 25

41.44 41.6667

41.45 50

41.46 66.6667

41.47 66.6667

41.48 41.6667

41.49 58.3333

41.5 33.3333

41.51 75

41.52 41.6667

41.53 41.6667

41.54 75

41.55 41.6667

41.56 50

41.57 16.6667

41.58 58.3333

41.59 58.3333

41.6 41.6667

41.61 66.6667

41.62 33.3333

41.63 50

41.64 41.6667

41.65 75

41.66 33.3333

41.67 25

41.68 33.3333

41.69 91.6667

41.7 91.6667

41.71 25

41.72 58.3333

41.73 33.3333

41.74 83.3333

41.75 16.6667

41.76 33.3333

41.77 100

41.78 33.3333

41.79 50

41.8 83.3333

41.81 41.6667

41.82 75

41.83 66.6667

41.84 50

41.85 66.6667

41.86 58.3333

41.87 75

41.88 66.6667

41.89 50

41.9 58.3333

41.91 50

41.92 50

41.93 75

41.94 41.6667

41.95 50

41.96 41.6667

41.97 16.6667

41.98 50

41.99 58.3333

42 58.3333

42.01 50

42.02 25

42.03 58.3333

42.04 66.6667

42.05 16.6667

42.06 25

42.07 50

42.08 75

42.09 75

42.1 58.3333

42.11 25

42.12 66.6667

42.13 41.6667

42.14 58.3333

42.15 41.6667

42.16 50

42.17 58.3333

42.18 50

42.19 91.6667

42.2 41.6667

42.21 25

42.22 41.6667

42.23 58.3333

42.24 41.6667

42.25 66.6667

42.26 100

42.27 33.3333

42.28 58.3333

42.29 50

42.3 50

42.31 50

42.32 41.6667

42.33 33.3333

42.34 50

42.35 33.3333

42.36 75

42.37 75

42.38 58.3333

42.39 33.3333

42.4 41.6667

42.41 41.6667

42.42 50

42.43 41.6667

42.44 108.333

42.45 58.3333

42.46 58.3333

42.47 41.6667

42.48 50

42.49 66.6667

42.5 58.3333

42.51 33.3333

42.52 50

42.53 41.6667

42.54 66.6667

42.55 41.6667

42.56 66.6667

42.57 75

42.58 16.6667

42.59 41.6667

42.6 66.6667

42.61 41.6667

42.62 50

42.63 58.3333

42.64 75

42.65 83.3333

42.66 8.33333

42.67 50

42.68 25

42.69 41.6667

42.7 91.6667

42.71 41.6667

42.72 41.6667

42.73 25

42.74 50

42.75 8.33333

42.76 58.3333

42.77 50

42.78 25

42.79 66.6667

42.8 33.3333

42.81 25

42.82 41.6667

42.83 50

42.84 66.6667

42.85 83.3333

42.86 66.6667

42.87 41.6667

42.88 50

42.89 25

42.9 66.6667

42.91 33.3333

42.92 50

42.93 58.3333

42.94 50

42.95 75

42.96 41.6667

42.97 75

42.98 83.3333

42.99 33.3333

43 33.3333

43.01 50

43.02 16.6667

43.03 41.6667

43.04 58.3333

43.05 41.6667

43.06 41.6667

43.07 58.3333

43.08 50

43.09 33.3333

43.1 50

43.11 58.3333

43.12 66.6667

43.13 58.3333

43.14 83.3333

43.15 66.6667

43.16 33.3333

43.17 16.6667

43.18 25

43.19 41.6667

43.2 75

43.21 58.3333

43.22 50

43.23 33.3333

43.24 83.3333

43.25 50

43.26 100

43.27 58.3333

43.28 66.6667

43.29 25

43.3 58.3333

43.31 91.6667

43.32 50

43.33 50

43.34 50

43.35 58.3333

43.36 58.3333

43.37 50

43.38 75

43.39 50

43.4 58.3333

43.41 83.3333

43.42 58.3333

43.43 75

43.44 50

43.45 50

43.46 41.6667

43.47 116.667

43.48 100

43.49 66.6667

43.5 91.6667

43.51 108.333

43.52 108.333

43.53 41.6667

43.54 83.3333

43.55 58.3333

43.56 108.333

43.57 58.3333

43.58 50

43.59 41.6667

43.6 50

43.61 75

43.62 58.3333

43.63 108.333

43.64 75

43.65 91.6667

43.66 50

43.67 66.6667

43.68 108.333

43.69 83.3333

43.7 58.3333

43.71 91.6667

43.72 83.3333

43.73 58.3333

43.74 75

43.75 58.3333

43.76 66.6667

43.77 66.6667

43.78 66.6667

43.79 75

43.8 116.667

43.81 66.6667

43.82 41.6667

43.83 33.3333

43.84 83.3333

43.85 116.667

43.86 33.3333

43.87 108.333

43.88 66.6667

43.89 58.3333

43.9 50

43.91 116.667

43.92 66.6667

43.93 100

43.94 83.3333

43.95 66.6667

43.96 100

43.97 100

43.98 91.6667

43.99 58.3333

44 66.6667

44.01 66.6667

44.02 25

44.03 66.6667

44.04 58.3333

44.05 83.3333

44.06 108.333

44.07 108.333

44.08 58.3333

44.09 91.6667

44.1 91.6667

44.11 58.3333

44.12 100

44.13 66.6667

44.14 58.3333

44.15 91.6667

44.16 125

44.17 108.333

44.18 50

44.19 33.3333

44.2 133.333

44.21 58.3333

44.22 133.333

44.23 150

44.24 58.3333

44.25 83.3333

44.26 66.6667

44.27 75

44.28 83.3333

44.29 75

44.3 83.3333

44.31 83.3333

44.32 91.6667

44.33 116.667

44.34 108.333

44.35 100

44.36 116.667

44.37 83.3333

44.38 91.6667

44.39 125

44.4 100

44.41 141.667

44.42 125

44.43 158.333

44.44 225

44.45 158.333

44.46 150

44.47 158.333

44.48 216.667

44.49 266.667

44.5 266.667

44.51 275

44.52 300

44.53 258.333

44.54 366.667

44.55 383.333

44.56 458.333

44.57 566.667

44.58 608.333

44.59 758.333

44.6 950

44.61 1308.33

44.62 1641.67

44.63 2175

44.64 2516.67

44.65 3166.67

44.66 3983.33

44.67 4458.33

44.68 4425

44.69 4466.67

44.7 4166.67

44.71 3866.67

44.72 3100

44.73 2650

44.74 2008.33

44.75 1816.67

44.76 1758.33

44.77 1766.67

44.78 1816.67

44.79 1966.67

44.8 2175

44.81 2125

44.82 1841.67

44.83 1525

44.84 1141.67

44.85 1091.67

44.86 766.667

44.87 500

44.88 358.333

44.89 300

44.9 250

44.91 166.667

44.92 175

44.93 200

44.94 108.333

44.95 108.333

44.96 108.333

44.97 150

44.98 116.667

44.99 183.333

45 91.6667

45.01 108.333

45.02 108.333

45.03 91.6667

45.04 83.3333

45.05 108.333

45.06 116.667

45.07 108.333

45.08 16.6667

45.09 50

45.1 83.3333

45.11 91.6667

45.12 75

45.13 91.6667

45.14 91.6667

45.15 75

45.16 83.3333

45.17 75

45.18 50

45.19 75

45.2 83.3333

45.21 75

45.22 41.6667

45.23 33.3333

45.24 41.6667

45.25 41.6667

45.26 66.6667

45.27 33.3333

45.28 58.3333

45.29 50

45.3 91.6667

45.31 58.3333

45.32 41.6667

45.33 50

45.34 25

45.35 50

45.36 58.3333

45.37 100

45.38 41.6667

45.39 50

45.4 33.3333

45.41 58.3333

45.42 66.6667

45.43 83.3333

45.44 33.3333

45.45 58.3333

45.46 66.6667

45.47 50

45.48 41.6667

45.49 50

45.5 83.3333

45.51 75

45.52 33.3333

45.53 33.3333

45.54 41.6667

45.55 16.6667

45.56 66.6667

45.57 16.6667

45.58 91.6667

45.59 25

45.6 50

45.61 25

45.62 33.3333

45.63 58.3333

45.64 50

45.65 66.6667

45.66 66.6667

45.67 66.6667

45.68 25

45.69 33.3333

45.7 66.6667

45.71 41.6667

45.72 16.6667

45.73 66.6667

45.74 58.3333

45.75 58.3333

45.76 33.3333

45.77 58.3333

45.78 66.6667

45.79 16.6667

45.8 33.3333

45.81 58.3333

45.82 50

45.83 8.33333

45.84 25

45.85 58.3333

45.86 16.6667

45.87 58.3333

45.88 33.3333

45.89 58.3333

45.9 58.3333

45.91 33.3333

45.92 75

45.93 58.3333

45.94 41.6667

45.95 83.3333

45.96 41.6667

45.97 50

45.98 83.3333

45.99 33.3333

46 41.6667

46.01 100

46.02 41.6667

46.03 75

46.04 25

46.05 25

46.06 58.3333

46.07 33.3333

46.08 25

46.09 41.6667

46.1 58.3333

46.11 33.3333

46.12 33.3333

46.13 91.6667

46.14 83.3333

46.15 16.6667

46.16 50

46.17 50

46.18 58.3333

46.19 50

46.2 41.6667

46.21 58.3333

46.22 83.3333

46.23 33.3333

46.24 33.3333

46.25 50

46.26 75

46.27 66.6667

46.28 41.6667

46.29 25

46.3 41.6667

46.31 50

46.32 83.3333

46.33 33.3333

46.34 50

46.35 41.6667

46.36 83.3333

46.37 50

46.38 41.6667

46.39 50

46.4 100

46.41 75

46.42 66.6667

46.43 50

46.44 75

46.45 50

46.46 66.6667

46.47 33.3333

46.48 16.6667

46.49 41.6667

46.5 66.6667

46.51 33.3333

46.52 58.3333

46.53 66.6667

46.54 50

46.55 33.3333

46.56 33.3333

46.57 58.3333

46.58 41.6667

46.59 25

46.6 50

46.61 58.3333

46.62 50

46.63 25

46.64 25

46.65 50

46.66 33.3333

46.67 41.6667

46.68 41.6667

46.69 50

46.7 66.6667

46.71 16.6667

46.72 0

46.73 50

46.74 83.3333

46.75 66.6667

46.76 50

46.77 33.3333

46.78 91.6667

46.79 66.6667

46.8 50

46.81 41.6667

46.82 50

46.83 33.3333

46.84 58.3333

46.85 8.33333

46.86 50

46.87 75

46.88 58.3333

46.89 41.6667

46.9 66.6667

46.91 33.3333

46.92 50

46.93 33.3333

46.94 66.6667

46.95 16.6667

46.96 50

46.97 41.6667

46.98 33.3333

46.99 58.3333

47 33.3333

47.01 41.6667

47.02 33.3333

47.03 50

47.04 33.3333

47.05 25

47.06 41.6667

47.07 66.6667

47.08 25

47.09 33.3333

47.1 25

47.11 8.33333

47.12 58.3333

47.13 66.6667

47.14 75

47.15 33.3333

47.16 41.6667

47.17 91.6667

47.18 58.3333

47.19 33.3333

47.2 33.3333

47.21 25

47.22 33.3333

47.23 41.6667

47.24 33.3333

47.25 50

47.26 25

47.27 66.6667

47.28 16.6667

47.29 58.3333

47.3 33.3333

47.31 41.6667

47.32 50

47.33 41.6667

47.34 0

47.35 91.6667

47.36 33.3333

47.37 66.6667

47.38 50

47.39 91.6667

47.4 25

47.41 75

47.42 41.6667

47.43 75

47.44 83.3333

47.45 25

47.46 16.6667

47.47 25

47.48 75

47.49 58.3333

47.5 50

47.51 41.6667

47.52 75

47.53 50

47.54 50

47.55 25

47.56 25

47.57 16.6667

47.58 58.3333

47.59 33.3333

47.6 33.3333

47.61 58.3333

47.62 33.3333

47.63 58.3333

47.64 58.3333

47.65 50

47.66 33.3333

47.67 50

47.68 33.3333

47.69 66.6667

47.7 41.6667

47.71 41.6667

47.72 83.3333

47.73 33.3333

47.74 41.6667

47.75 33.3333

47.76 58.3333

47.77 33.3333

47.78 66.6667

47.79 75

47.8 41.6667

47.81 91.6667

47.82 41.6667

47.83 58.3333

47.84 75

47.85 58.3333

47.86 25

47.87 75

47.88 33.3333

47.89 50

47.9 58.3333

47.91 66.6667

47.92 66.6667

47.93 66.6667

47.94 33.3333

47.95 50

47.96 50

47.97 50

47.98 33.3333

47.99 66.6667

48 33.3333

48.01 58.3333

48.02 50

48.03 25

48.04 33.3333

48.05 75

48.06 41.6667

48.07 50

48.08 66.6667

48.09 75

48.1 66.6667

48.11 16.6667

48.12 50

48.13 25

48.14 41.6667

48.15 91.6667

48.16 58.3333

48.17 41.6667

48.18 50

48.19 50

48.2 8.33333

48.21 41.6667

48.22 58.3333

48.23 50

48.24 41.6667

48.25 50

48.26 41.6667

48.27 16.6667

48.28 50

48.29 50

48.3 75

48.31 41.6667

48.32 58.3333

48.33 41.6667

48.34 58.3333

48.35 41.6667

48.36 66.6667

48.37 50

48.38 50

48.39 66.6667

48.4 66.6667

48.41 33.3333

48.42 66.6667

48.43 75

48.44 58.3333

48.45 50

48.46 100

48.47 33.3333

48.48 50

48.49 33.3333

48.5 41.6667

48.51 75

48.52 25

48.53 41.6667

48.54 33.3333

48.55 50

48.56 41.6667

48.57 41.6667

48.58 41.6667

48.59 25

48.6 16.6667

48.61 33.3333

48.62 33.3333

48.63 50

48.64 8.33333

48.65 25

48.66 33.3333

48.67 75

48.68 75

48.69 16.6667

48.7 50

48.71 41.6667

48.72 41.6667

48.73 50

48.74 66.6667

48.75 50

48.76 25

48.77 41.6667

48.78 50

48.79 50

48.8 25

48.81 41.6667

48.82 25

48.83 25

48.84 33.3333

48.85 50

48.86 91.6667

48.87 33.3333

48.88 25

48.89 25

48.9 33.3333

48.91 116.667

48.92 33.3333

48.93 41.6667

48.94 33.3333

48.95 16.6667

48.96 33.3333

48.97 33.3333

48.98 41.6667

48.99 66.6667

49 41.6667

49.01 41.6667

49.02 41.6667

49.03 50

49.04 58.3333

49.05 50

49.06 8.33333

49.07 41.6667

49.08 41.6667

49.09 50

49.1 33.3333

49.11 41.6667

49.12 41.6667

49.13 66.6667

49.14 25

49.15 75

49.16 33.3333

49.17 41.6667

49.18 25

49.19 58.3333

49.2 25

49.21 25

49.22 50

49.23 50

49.24 33.3333

49.25 33.3333

49.26 33.3333

49.27 25

49.28 41.6667

49.29 50

49.3 16.6667

49.31 41.6667

49.32 33.3333

49.33 41.6667

49.34 41.6667

49.35 58.3333

49.36 0

49.37 58.3333

49.38 25

49.39 50

49.4 33.3333

49.41 25

49.42 58.3333

49.43 33.3333

49.44 108.333

49.45 16.6667

49.46 41.6667

49.47 75

49.48 50

49.49 25

49.5 41.6667

49.51 66.6667

49.52 66.6667

49.53 33.3333

49.54 50

49.55 50

49.56 66.6667

49.57 16.6667

49.58 50

49.59 50

49.6 83.3333

49.61 41.6667

49.62 50

49.63 91.6667

49.64 66.6667

49.65 25

49.66 66.6667

49.67 50

49.68 41.6667

49.69 58.3333

49.7 108.333

49.71 41.6667

49.72 50

49.73 41.6667

49.74 83.3333

49.75 66.6667

49.76 16.6667

49.77 41.6667

49.78 108.333

49.79 41.6667

49.8 66.6667

49.81 25

49.82 66.6667

49.83 50

49.84 41.6667

49.85 33.3333

49.86 33.3333

49.87 58.3333

49.88 50

49.89 25

49.9 50

49.91 25

49.92 50

49.93 33.3333

49.94 66.6667

49.95 50

49.96 33.3333

49.97 33.3333

49.98 75

49.99 33.3333

50 33.3333

50.01 50

50.02 66.6667

50.03 75

50.04 50

50.05 58.3333

50.06 58.3333

50.07 25

50.08 58.3333

50.09 33.3333

50.1 50

50.11 33.3333

50.12 25

50.13 50

50.14 25

50.15 50

50.16 66.6667

50.17 66.6667

50.18 33.3333

50.19 50

50.2 33.3333

50.21 33.3333

50.22 25

50.23 50

50.24 58.3333

50.25 0

50.26 41.6667

50.27 58.3333

50.28 33.3333

50.29 25

50.3 66.6667

50.31 50

50.32 100

50.33 50

50.34 41.6667

50.35 50

50.36 41.6667

50.37 33.3333

50.38 33.3333

50.39 66.6667

50.4 25

50.41 25

50.42 108.333

50.43 8.33333

50.44 33.3333

50.45 91.6667

50.46 25

50.47 25

50.48 33.3333

50.49 16.6667

50.5 41.6667

50.51 33.3333

50.52 66.6667

50.53 50

50.54 41.6667

50.55 66.6667

50.56 41.6667

50.57 83.3333

50.58 25

50.59 75

50.6 16.6667

50.61 33.3333

50.62 41.6667

50.63 75

50.64 50

50.65 50

50.66 16.6667

50.67 25

50.68 50

50.69 25

50.7 33.3333

50.71 16.6667

50.72 25

50.73 41.6667

50.74 58.3333

50.75 50

50.76 75

50.77 50

50.78 33.3333

50.79 50

50.8 41.6667

50.81 41.6667

50.82 25

50.83 41.6667

50.84 41.6667

50.85 16.6667

50.86 41.6667

50.87 41.6667

50.88 91.6667

50.89 33.3333

50.9 25

50.91 50

50.92 41.6667

50.93 33.3333

50.94 66.6667

50.95 75

50.96 58.3333

50.97 16.6667

50.98 50

50.99 41.6667

51 33.3333

51.01 91.6667

51.02 75

51.03 25

51.04 100

51.05 33.3333

51.06 50

51.07 41.6667

51.08 25

51.09 58.3333

51.1 16.6667

51.11 58.3333

51.12 25

51.13 50

51.14 41.6667

51.15 25

51.16 66.6667

51.17 58.3333

51.18 41.6667

51.19 66.6667

51.2 25

51.21 16.6667

51.22 25

51.23 50

51.24 66.6667

51.25 25

51.26 58.3333

51.27 66.6667

51.28 50

51.29 50

51.3 41.6667

51.31 16.6667

51.32 75

51.33 41.6667

51.34 16.6667

51.35 33.3333

51.36 33.3333

51.37 50

51.38 25

51.39 8.33333

51.4 83.3333

51.41 58.3333

51.42 50

51.43 33.3333

51.44 25

51.45 41.6667

51.46 58.3333

51.47 75

51.48 66.6667

51.49 50

51.5 41.6667

51.51 33.3333

51.52 25

51.53 25

51.54 33.3333

51.55 16.6667

51.56 33.3333

51.57 25

51.58 58.3333

51.59 50

51.6 58.3333

51.61 58.3333

51.62 33.3333

51.63 83.3333

51.64 50

51.65 33.3333

51.66 33.3333

51.67 16.6667

51.68 50

51.69 58.3333

51.7 33.3333

51.71 33.3333

51.72 16.6667

51.73 25

51.74 33.3333

51.75 50

51.76 33.3333

51.77 58.3333

51.78 41.6667

51.79 33.3333

51.8 8.33333

51.81 41.6667

51.82 25

51.83 58.3333

51.84 25

51.85 50

51.86 50

51.87 50

51.88 83.3333

51.89 41.6667

51.9 58.3333

51.91 66.6667

51.92 41.6667

51.93 33.3333

51.94 41.6667

51.95 33.3333

51.96 25

51.97 50

51.98 33.3333

51.99 33.3333

52 25

52.01 66.6667

52.02 25

52.03 41.6667

52.04 58.3333

52.05 66.6667

52.06 50

52.07 8.33333

52.08 16.6667

52.09 58.3333

52.1 25

52.11 75

52.12 41.6667

52.13 41.6667

52.14 41.6667

52.15 33.3333

52.16 16.6667

52.17 16.6667

52.18 16.6667

52.19 25

52.2 58.3333

52.21 83.3333

52.22 41.6667

52.23 41.6667

52.24 41.6667

52.25 58.3333

52.26 8.33333

52.27 50

52.28 58.3333

52.29 41.6667

52.3 41.6667

52.31 33.3333

52.32 41.6667

52.33 33.3333

52.34 16.6667

52.35 50

52.36 50

52.37 66.6667

52.38 25

52.39 41.6667

52.4 75

52.41 25

52.42 41.6667

52.43 66.6667

52.44 58.3333

52.45 58.3333

52.46 25

52.47 58.3333

52.48 66.6667

52.49 25

52.5 50

52.51 58.3333

52.52 41.6667

52.53 41.6667

52.54 50

52.55 16.6667

52.56 33.3333

52.57 8.33333

52.58 41.6667

52.59 41.6667

52.6 16.6667

52.61 41.6667

52.62 58.3333

52.63 75

52.64 58.3333

52.65 16.6667

52.66 58.3333

52.67 50

52.68 66.6667

52.69 16.6667

52.7 33.3333

52.71 25

52.72 0

52.73 41.6667

52.74 25

52.75 41.6667

52.76 41.6667

52.77 33.3333

52.78 50

52.79 41.6667

52.8 50

52.81 25

52.82 58.3333

52.83 25

52.84 25

52.85 66.6667

52.86 75

52.87 8.33333

52.88 58.3333

52.89 58.3333

52.9 25

52.91 41.6667

52.92 16.6667

52.93 50

52.94 33.3333

52.95 25

52.96 58.3333

52.97 41.6667

52.98 33.3333

52.99 33.3333

53 58.3333

53.01 41.6667

53.02 33.3333

53.03 58.3333

53.04 41.6667

53.05 33.3333

53.06 33.3333

53.07 16.6667

53.08 25

53.09 75

53.1 58.3333

53.11 50

53.12 50

53.13 33.3333

53.14 50

53.15 33.3333

53.16 16.6667

53.17 41.6667

53.18 25

53.19 50

53.2 25

53.21 16.6667

53.22 66.6667

53.23 16.6667

53.24 58.3333

53.25 125

53.26 33.3333

53.27 41.6667

53.28 50

53.29 58.3333

53.3 50

53.31 50

53.32 41.6667

53.33 58.3333

53.34 33.3333

53.35 16.6667

53.36 33.3333

53.37 50

53.38 41.6667

53.39 66.6667

53.4 58.3333

53.41 41.6667

53.42 33.3333

53.43 25

53.44 41.6667

53.45 50

53.46 33.3333

53.47 16.6667

53.48 33.3333

53.49 41.6667

53.5 83.3333

53.51 66.6667

53.52 41.6667

53.53 33.3333

53.54 41.6667

53.55 50

53.56 8.33333

53.57 33.3333

53.58 41.6667

53.59 33.3333

53.6 58.3333

53.61 41.6667

53.62 25

53.63 16.6667

53.64 58.3333

53.65 58.3333

53.66 41.6667

53.67 41.6667

53.68 50

53.69 8.33333

53.7 33.3333

53.71 50

53.72 41.6667

53.73 33.3333

53.74 41.6667

53.75 41.6667

53.76 58.3333

53.77 83.3333

53.78 50

53.79 41.6667

53.8 25

53.81 66.6667

53.82 75

53.83 33.3333

53.84 16.6667

53.85 66.6667

53.86 58.3333

53.87 25

53.88 33.3333

53.89 33.3333

53.9 58.3333

53.91 50

53.92 16.6667

53.93 83.3333

53.94 16.6667

53.95 58.3333

53.96 75

53.97 50

53.98 25

53.99 25

54 16.6667

54.01 58.3333

54.02 58.3333

54.03 25

54.04 25

54.05 58.3333

54.06 25

54.07 41.6667

54.08 41.6667

54.09 50

54.1 25

54.11 33.3333

54.12 58.3333

54.13 33.3333

54.14 16.6667

54.15 25

54.16 41.6667

54.17 33.3333

54.18 33.3333

54.19 16.6667

54.2 58.3333

54.21 58.3333

54.22 75

54.23 16.6667

54.24 41.6667

54.25 33.3333

54.26 41.6667

54.27 66.6667

54.28 50

54.29 83.3333

54.3 25

54.31 33.3333

54.32 41.6667

54.33 58.3333

54.34 16.6667

54.35 50

54.36 41.6667

54.37 25

54.38 41.6667

54.39 50

54.4 50

54.41 58.3333

54.42 50

54.43 16.6667

54.44 41.6667

54.45 33.3333

54.46 58.3333

54.47 33.3333

54.48 16.6667

54.49 8.33333

54.5 66.6667

54.51 33.3333

54.52 58.3333

54.53 58.3333

54.54 75

54.55 33.3333

54.56 50

54.57 75

54.58 25

54.59 66.6667

54.6 66.6667

54.61 8.33333

54.62 41.6667

54.63 50

54.64 33.3333

54.65 33.3333

54.66 41.6667

54.67 33.3333

54.68 58.3333

54.69 41.6667

54.7 41.6667

54.71 41.6667

54.72 33.3333

54.73 83.3333

54.74 33.3333

54.75 91.6667

54.76 41.6667

54.77 8.33333

54.78 50

54.79 25

54.8 41.6667

54.81 25

54.82 58.3333

54.83 41.6667

54.84 0

54.85 41.6667

54.86 16.6667

54.87 50

54.88 25

54.89 50

54.9 41.6667

54.91 66.6667

54.92 83.3333

54.93 50

54.94 58.3333

54.95 33.3333

54.96 25

54.97 41.6667

54.98 50

54.99 58.3333

55 25

55.01 91.6667

55.02 50

55.03 58.3333

55.04 50

55.05 75

55.06 75

55.07 41.6667

55.08 75

55.09 58.3333

55.1 16.6667

55.11 50

55.12 75

55.13 16.6667

55.14 33.3333

55.15 83.3333

55.16 58.3333

55.17 25

55.18 58.3333

55.19 58.3333

55.2 58.3333

55.21 25

55.22 41.6667

55.23 33.3333

55.24 41.6667

55.25 33.3333

55.26 33.3333

55.27 41.6667

55.28 41.6667

55.29 41.6667

55.3 41.6667

55.31 41.6667

55.32 33.3333

55.33 41.6667

55.34 41.6667

55.35 41.6667

55.36 33.3333

55.37 66.6667

55.38 33.3333

55.39 25

55.4 41.6667

55.41 33.3333

55.42 66.6667

55.43 16.6667

55.44 33.3333

55.45 75

55.46 16.6667

55.47 66.6667

55.48 33.3333

55.49 50

55.5 41.6667

55.51 25

55.52 50

55.53 58.3333

55.54 25

55.55 66.6667

55.56 41.6667

55.57 25

55.58 33.3333

55.59 33.3333

55.6 50

55.61 25

55.62 58.3333

55.63 50

55.64 25

55.65 33.3333

55.66 25

55.67 16.6667

55.68 25

55.69 66.6667

55.7 58.3333

55.71 25

55.72 41.6667

55.73 25

55.74 41.6667

55.75 41.6667

55.76 25

55.77 16.6667

55.78 91.6667

55.79 66.6667

55.8 41.6667

55.81 58.3333

55.82 50

55.83 58.3333

55.84 41.6667

55.85 25

55.86 41.6667

55.87 25

55.88 50

55.89 41.6667

55.9 33.3333

55.91 50

55.92 25

55.93 8.33333

55.94 16.6667

55.95 58.3333

55.96 25

55.97 0

55.98 33.3333

55.99 25

56 58.3333

56.01 16.6667

56.02 75

56.03 25

56.04 50

56.05 33.3333

56.06 58.3333

56.07 58.3333

56.08 50

56.09 16.6667

56.1 41.6667

56.11 16.6667

56.12 8.33333

56.13 33.3333

56.14 58.3333

56.15 58.3333

56.16 33.3333

56.17 41.6667

56.18 25

56.19 0

56.2 108.333

56.21 50

56.22 41.6667

56.23 41.6667

56.24 83.3333

56.25 25

56.26 16.6667

56.27 50

56.28 33.3333

56.29 33.3333

56.3 50

56.31 25

56.32 75

56.33 41.6667

56.34 33.3333

56.35 58.3333

56.36 41.6667

56.37 50

56.38 25

56.39 75

56.4 66.6667

56.41 66.6667

56.42 50

56.43 33.3333

56.44 41.6667

56.45 25

56.46 8.33333

56.47 66.6667

56.48 58.3333

56.49 41.6667

56.5 33.3333

56.51 33.3333

56.52 50

56.53 41.6667

56.54 33.3333

56.55 33.3333

56.56 33.3333

56.57 33.3333

56.58 16.6667

56.59 100

56.6 50

56.61 41.6667

56.62 16.6667

56.63 41.6667

56.64 50

56.65 58.3333

56.66 83.3333

56.67 41.6667

56.68 33.3333

56.69 25

56.7 33.3333

56.71 66.6667

56.72 83.3333

56.73 58.3333

56.74 33.3333

56.75 41.6667

56.76 25

56.77 41.6667

56.78 50

56.79 33.3333

56.8 83.3333

56.81 58.3333

56.82 25

56.83 33.3333

56.84 58.3333

56.85 50

56.86 50

56.87 8.33333

56.88 25

56.89 25

56.9 25

56.91 33.3333

56.92 16.6667

56.93 50

56.94 25

56.95 58.3333

56.96 25

56.97 33.3333

56.98 8.33333

56.99 16.6667

57 16.6667

57.01 41.6667

57.02 50

57.03 8.33333

57.04 50

57.05 50

57.06 50

57.07 58.3333

57.08 50

57.09 75

57.1 16.6667

57.11 41.6667

57.12 16.6667

57.13 25

57.14 58.3333

57.15 33.3333

57.16 41.6667

57.17 33.3333

57.18 58.3333

57.19 33.3333

57.2 91.6667

57.21 58.3333

57.22 58.3333

57.23 58.3333

57.24 16.6667

57.25 33.3333

57.26 16.6667

57.27 50

57.28 91.6667

57.29 8.33333

57.3 41.6667

57.31 25

57.32 8.33333

57.33 16.6667

57.34 33.3333

57.35 50

57.36 8.33333

57.37 41.6667

57.38 25

57.39 50

57.4 33.3333

57.41 50

57.42 25

57.43 66.6667

57.44 58.3333

57.45 25

57.46 16.6667

57.47 16.6667

57.48 75

57.49 41.6667

57.5 75

57.51 33.3333

57.52 41.6667

57.53 50

57.54 33.3333

57.55 83.3333

57.56 50

57.57 58.3333

57.58 16.6667

57.59 41.6667

57.6 75

57.61 41.6667

57.62 50

57.63 33.3333

57.64 50

57.65 25

57.66 25

57.67 41.6667

57.68 58.3333

57.69 16.6667

57.7 41.6667

57.71 83.3333

57.72 8.33333

57.73 75

57.74 33.3333

57.75 33.3333

57.76 16.6667

57.77 41.6667

57.78 75

57.79 66.6667

57.8 66.6667

57.81 33.3333

57.82 41.6667

57.83 108.333

57.84 75

57.85 58.3333

57.86 33.3333

57.87 41.6667

57.88 50

57.89 58.3333

57.9 58.3333

57.91 58.3333

57.92 83.3333

57.93 33.3333

57.94 41.6667

57.95 33.3333

57.96 33.3333

57.97 33.3333

57.98 0

57.99 50

58 58.3333

58.01 16.6667

58.02 83.3333

58.03 33.3333

58.04 33.3333

58.05 16.6667

58.06 58.3333

58.07 41.6667

58.08 66.6667

58.09 41.6667

58.1 25

58.11 50

58.12 50

58.13 50

58.14 58.3333

58.15 33.3333

58.16 41.6667

58.17 58.3333

58.18 25

58.19 50

58.2 50

58.21 41.6667

58.22 41.6667

58.23 58.3333

58.24 66.6667

58.25 41.6667

58.26 41.6667

58.27 75

58.28 66.6667

58.29 33.3333

58.3 50

58.31 25

58.32 50

58.33 66.6667

58.34 66.6667

58.35 41.6667

58.36 8.33333

58.37 25

58.38 25

58.39 25

58.4 25

58.41 25

58.42 8.33333

58.43 16.6667

58.44 33.3333

58.45 41.6667

58.46 33.3333

58.47 58.3333

58.48 16.6667

58.49 33.3333

58.5 58.3333

58.51 41.6667

58.52 33.3333

58.53 33.3333

58.54 58.3333

58.55 58.3333

58.56 58.3333

58.57 16.6667

58.58 50

58.59 75

58.6 25

58.61 58.3333

58.62 33.3333

58.63 33.3333

58.64 41.6667

58.65 8.33333

58.66 16.6667

58.67 25

58.68 33.3333

58.69 41.6667

58.7 66.6667

58.71 16.6667

58.72 50

58.73 41.6667

58.74 8.33333

58.75 41.6667

58.76 91.6667

58.77 33.3333

58.78 50

58.79 16.6667

58.8 25

58.81 41.6667

58.82 33.3333

58.83 50

58.84 25

58.85 66.6667

58.86 33.3333

58.87 41.6667

58.88 58.3333

58.89 41.6667

58.9 50

58.91 75

58.92 16.6667

58.93 8.33333

58.94 33.3333

58.95 50

58.96 83.3333

58.97 41.6667

58.98 75

58.99 8.33333

59 33.3333

59.01 33.3333

59.02 50

59.03 50

59.04 83.3333

59.05 58.3333

59.06 16.6667

59.07 33.3333

59.08 66.6667

59.09 33.3333

59.1 25

59.11 25

59.12 33.3333

59.13 41.6667

59.14 25

59.15 16.6667

59.16 25

59.17 91.6667

59.18 50

59.19 41.6667

59.2 41.6667

59.21 33.3333

59.22 33.3333

59.23 66.6667

59.24 50

59.25 66.6667

59.26 50

59.27 33.3333

59.28 25

59.29 16.6667

59.3 41.6667

59.31 16.6667

59.32 41.6667

59.33 66.6667

59.34 41.6667

59.35 75

59.36 50

59.37 33.3333

59.38 16.6667

59.39 66.6667

59.4 33.3333

59.41 58.3333

59.42 33.3333

59.43 58.3333

59.44 66.6667

59.45 16.6667

59.46 25

59.47 33.3333

59.48 58.3333

59.49 41.6667

59.5 58.3333

59.51 33.3333

59.52 75

59.53 41.6667

59.54 75

59.55 58.3333

59.56 41.6667

59.57 66.6667

59.58 33.3333

59.59 33.3333

59.6 58.3333

59.61 58.3333

59.62 33.3333

59.63 33.3333

59.64 33.3333

59.65 16.6667

59.66 50

59.67 33.3333

59.68 16.6667

59.69 25

59.7 58.3333

59.71 33.3333

59.72 33.3333

59.73 33.3333

59.74 50

59.75 50

59.76 50

59.77 83.3333

59.78 58.3333

59.79 50

59.8 25

59.81 33.3333

59.82 58.3333

59.83 41.6667

59.84 33.3333

59.85 83.3333

59.86 50

59.87 50

59.88 33.3333

59.89 16.6667

59.9 75

59.91 83.3333

59.92 50

59.93 58.3333

59.94 50

59.95 0

59.96 41.6667

59.97 58.3333

59.98 8.33333

59.99 41.6667

60 66.6667

60.01 83.3333

60.02 25

60.03 50

60.04 50

60.05 33.3333

60.06 41.6667

60.07 66.6667

60.08 33.3333

60.09 66.6667

60.1 16.6667

60.11 25

60.12 33.3333

60.13 75

60.14 25

60.15 33.3333

60.16 33.3333

60.17 58.3333

60.18 25

60.19 66.6667

60.2 50

60.21 108.333

60.22 16.6667

60.23 50

60.24 58.3333

60.25 50

60.26 41.6667

60.27 50

60.28 33.3333

60.29 50

60.3 83.3333

60.31 58.3333

60.32 16.6667

60.33 58.3333

60.34 50

60.35 8.33333

60.36 33.3333

60.37 50

60.38 66.6667

60.39 33.3333

60.4 16.6667

60.41 50

60.42 25

60.43 25

60.44 25

60.45 16.6667

60.46 41.6667

60.47 58.3333

60.48 75

60.49 50

60.5 25

60.51 50

60.52 75

60.53 33.3333

60.54 41.6667

60.55 41.6667

60.56 50

60.57 41.6667

60.58 33.3333

60.59 41.6667

60.6 58.3333

60.61 33.3333

60.62 50

60.63 58.3333

60.64 33.3333

60.65 83.3333

60.66 41.6667

60.67 58.3333

60.68 83.3333

60.69 58.3333

60.7 41.6667

60.71 50

60.72 41.6667

60.73 8.33333

60.74 33.3333

60.75 58.3333

60.76 25

60.77 33.3333

60.78 8.33333

60.79 50

60.8 66.6667

60.81 50

60.82 50

60.83 25

60.84 25

60.85 16.6667

60.86 66.6667

60.87 16.6667

60.88 16.6667

60.89 16.6667

60.9 41.6667

60.91 66.6667

60.92 41.6667

60.93 16.6667

60.94 41.6667

60.95 58.3333

60.96 33.3333

60.97 50

60.98 58.3333

60.99 58.3333

61 58.3333

61.01 25

61.02 75

61.03 25

61.04 50

61.05 50

61.06 0

61.07 83.3333

61.08 50

61.09 50

61.1 75

61.11 8.33333

61.12 75

61.13 33.3333

61.14 25

61.15 33.3333

61.16 58.3333

61.17 75

61.18 91.6667

61.19 66.6667

61.2 50

61.21 50

61.22 33.3333

61.23 50

61.24 91.6667

61.25 33.3333

61.26 58.3333

61.27 41.6667

61.28 41.6667

61.29 41.6667

61.3 58.3333

61.31 66.6667

61.32 8.33333

61.33 8.33333

61.34 75

61.35 25

61.36 75

61.37 33.3333

61.38 83.3333

61.39 50

61.4 16.6667

61.41 58.3333

61.42 66.6667

61.43 41.6667

61.44 58.3333

61.45 41.6667

61.46 41.6667

61.47 16.6667

61.48 33.3333

61.49 25

61.5 41.6667

61.51 58.3333

61.52 50

61.53 33.3333

61.54 16.6667

61.55 25

61.56 33.3333

61.57 41.6667

61.58 25

61.59 41.6667

61.6 25

61.61 25

61.62 50

61.63 25

61.64 41.6667

61.65 75

61.66 25

61.67 33.3333

61.68 25

61.69 41.6667

61.7 33.3333

61.71 58.3333

61.72 33.3333

61.73 16.6667

61.74 33.3333

61.75 58.3333

61.76 50

61.77 75

61.78 33.3333

61.79 33.3333

61.8 66.6667

61.81 108.333

61.82 58.3333

61.83 66.6667

61.84 50

61.85 33.3333

61.86 50

61.87 33.3333

61.88 41.6667

61.89 66.6667

61.9 50

61.91 41.6667

61.92 16.6667

61.93 75

61.94 33.3333

61.95 50

61.96 41.6667

61.97 33.3333

61.98 66.6667

61.99 50

62 83.3333

62.01 75

62.02 41.6667

62.03 33.3333

62.04 58.3333

62.05 8.33333

62.06 33.3333

62.07 33.3333

62.08 33.3333

62.09 75

62.1 58.3333

62.11 41.6667

62.12 25

62.13 25

62.14 50

62.15 33.3333

62.16 66.6667

62.17 50

62.18 66.6667

62.19 50

62.2 50

62.21 41.6667

62.22 41.6667

62.23 58.3333

62.24 33.3333

62.25 25

62.26 25

62.27 83.3333

62.28 50

62.29 33.3333

62.3 91.6667

62.31 41.6667

62.32 58.3333

62.33 41.6667

62.34 50

62.35 58.3333

62.36 75

62.37 25

62.38 41.6667

62.39 66.6667

62.4 75

62.41 33.3333

62.42 66.6667

62.43 25

62.44 33.3333

62.45 58.3333

62.46 58.3333

62.47 66.6667

62.48 8.33333

62.49 25

62.5 58.3333

62.51 50

62.52 75

62.53 41.6667

62.54 58.3333

62.55 75

62.56 41.6667

62.57 66.6667

62.58 33.3333

62.59 66.6667

62.6 100

62.61 66.6667

62.62 25

62.63 41.6667

62.64 25

62.65 41.6667

62.66 8.33333

62.67 50

62.68 16.6667

62.69 141.667

62.7 58.3333

62.71 41.6667

62.72 16.6667

62.73 33.3333

62.74 91.6667

62.75 66.6667

62.76 50

62.77 58.3333

62.78 25

62.79 50

62.8 41.6667

62.81 41.6667

62.82 50

62.83 33.3333

62.84 25

62.85 50

62.86 33.3333

62.87 50

62.88 75

62.89 50

62.9 25

62.91 41.6667

62.92 58.3333

62.93 83.3333

62.94 25

62.95 33.3333

62.96 66.6667

62.97 25

62.98 33.3333

62.99 41.6667

63 41.6667

63.01 16.6667

63.02 91.6667

63.03 58.3333

63.04 58.3333

63.05 50

63.06 25

63.07 25

63.08 66.6667

63.09 41.6667

63.1 58.3333

63.11 58.3333

63.12 50

63.13 25

63.14 58.3333

63.15 25

63.16 50

63.17 25

63.18 50

63.19 58.3333

63.2 50

63.21 58.3333

63.22 66.6667

63.23 83.3333

63.24 83.3333

63.25 83.3333

63.26 83.3333

63.27 58.3333

63.28 33.3333

63.29 33.3333

63.3 33.3333

63.31 16.6667

63.32 16.6667

63.33 33.3333

63.34 41.6667

63.35 58.3333

63.36 75

63.37 25

63.38 50

63.39 25

63.4 16.6667

63.41 66.6667

63.42 58.3333

63.43 75

63.44 58.3333

63.45 25

63.46 50

63.47 41.6667

63.48 50

63.49 50

63.5 83.3333

63.51 25

63.52 50

63.53 58.3333

63.54 50

63.55 25

63.56 25

63.57 33.3333

63.58 50

63.59 41.6667

63.6 50

63.61 50

63.62 75

63.63 25

63.64 25

63.65 66.6667

63.66 50

63.67 50

63.68 83.3333

63.69 25

63.7 8.33333

63.71 16.6667

63.72 41.6667

63.73 91.6667

63.74 33.3333

63.75 25

63.76 50

63.77 41.6667

63.78 33.3333

63.79 50

63.8 50

63.81 16.6667

63.82 41.6667

63.83 41.6667

63.84 75

63.85 33.3333

63.86 83.3333

63.87 108.333

63.88 33.3333

63.89 58.3333

63.9 33.3333

63.91 33.3333

63.92 66.6667

63.93 41.6667

63.94 100

63.95 41.6667

63.96 33.3333

63.97 50

63.98 16.6667

63.99 50

64 50

64.01 58.3333

64.02 41.6667

64.03 50

64.04 58.3333

64.05 33.3333

64.06 66.6667

64.07 25

64.08 58.3333

64.09 41.6667

64.1 50

64.11 108.333

64.12 50

64.13 33.3333

64.14 16.6667

64.15 50

64.16 75

64.17 58.3333

64.18 25

64.19 16.6667

64.2 8.33333

64.21 66.6667

64.22 58.3333

64.23 25

64.24 25

64.25 41.6667

64.26 83.3333

64.27 16.6667

64.28 25

64.29 75

64.3 66.6667

64.31 66.6667

64.32 50

64.33 33.3333

64.34 41.6667

64.35 41.6667

64.36 41.6667

64.37 16.6667

64.38 41.6667

64.39 41.6667

64.4 66.6667

64.41 58.3333

64.42 33.3333

64.43 50

64.44 50

64.45 41.6667

64.46 83.3333

64.47 41.6667

64.48 50

64.49 83.3333

64.5 58.3333

64.51 75

64.52 41.6667

64.53 66.6667

64.54 16.6667

64.55 41.6667

64.56 41.6667

64.57 41.6667

64.58 58.3333

64.59 75

64.6 66.6667

64.61 58.3333

64.62 50

64.63 66.6667

64.64 66.6667

64.65 66.6667

64.66 50

64.67 66.6667

64.68 25

64.69 50

64.7 50

64.71 41.6667

64.72 33.3333

64.73 66.6667

64.74 33.3333

64.75 75

64.76 50

64.77 58.3333

64.78 50

64.79 91.6667

64.8 58.3333

64.81 66.6667

64.82 100

64.83 58.3333

64.84 75

64.85 25

64.86 83.3333

64.87 75

64.88 83.3333

64.89 75

64.9 116.667

64.91 191.667

64.92 125

64.93 125

64.94 191.667

64.95 216.667

64.96 166.667

64.97 333.333

64.98 358.333

64.99 366.667

65 533.333

65.01 583.333

65.02 650

65.03 641.667

65.04 608.333

65.05 650

65.06 516.667

65.07 575

65.08 416.667

65.09 366.667

65.1 391.667

65.11 325

65.12 141.667

65.13 200

65.14 183.333

65.15 241.667

65.16 216.667

65.17 191.667

65.18 200

65.19 250

65.2 333.333

65.21 275

65.22 375

65.23 200

65.24 241.667

65.25 241.667

65.26 233.333

65.27 183.333

65.28 125

65.29 133.333

65.3 125

65.31 75

65.32 58.3333

65.33 66.6667

65.34 83.3333

65.35 66.6667

65.36 100

65.37 58.3333

65.38 58.3333

65.39 116.667

65.4 66.6667

65.41 75

65.42 41.6667

65.43 66.6667

65.44 41.6667

65.45 25

65.46 33.3333

65.47 41.6667

65.48 75

65.49 50

65.5 41.6667

65.51 83.3333

65.52 58.3333

65.53 16.6667

65.54 75

65.55 16.6667

65.56 66.6667

65.57 83.3333

65.58 16.6667

65.59 75

65.6 58.3333

65.61 33.3333

65.62 33.3333

65.63 25

65.64 41.6667

65.65 25

65.66 41.6667

65.67 33.3333

65.68 58.3333

65.69 58.3333

65.7 91.6667

65.71 33.3333

65.72 75

65.73 66.6667

65.74 75

65.75 50

65.76 25

65.77 58.3333

65.78 66.6667

65.79 83.3333

65.8 75

65.81 66.6667

65.82 58.3333

65.83 16.6667

65.84 50

65.85 75

65.86 41.6667

65.87 50

65.88 58.3333

65.89 33.3333

65.9 58.3333

65.91 100

65.92 41.6667

65.93 16.6667

65.94 33.3333

65.95 58.3333

65.96 16.6667

65.97 58.3333

65.98 33.3333

65.99 33.3333

66 33.3333

66.01 41.6667

66.02 16.6667

66.03 50

66.04 25

66.05 66.6667

66.06 41.6667

66.07 58.3333

66.08 41.6667

66.09 25

66.1 41.6667

66.11 50

66.12 33.3333

66.13 58.3333

66.14 58.3333

66.15 25

66.16 33.3333

66.17 58.3333

66.18 33.3333

66.19 41.6667

66.2 33.3333

66.21 33.3333

66.22 75

66.23 33.3333

66.24 25

66.25 33.3333

66.26 58.3333

66.27 33.3333

66.28 33.3333

66.29 108.333

66.3 33.3333

66.31 41.6667

66.32 58.3333

66.33 83.3333

66.34 16.6667

66.35 25

66.36 50

66.37 50

66.38 75

66.39 41.6667

66.4 16.6667

66.41 33.3333

66.42 41.6667

66.43 66.6667

66.44 33.3333

66.45 33.3333

66.46 41.6667

66.47 33.3333

66.48 58.3333

66.49 41.6667

66.5 50

66.51 75

66.52 58.3333

66.53 50

66.54 66.6667

66.55 66.6667

66.56 66.6667

66.57 66.6667

66.58 33.3333

66.59 41.6667

66.6 41.6667

66.61 50

66.62 41.6667

66.63 75

66.64 75

66.65 66.6667

66.66 50

66.67 33.3333

66.68 41.6667

66.69 58.3333

66.7 75

66.71 91.6667

66.72 66.6667

66.73 50

66.74 25

66.75 41.6667

66.76 66.6667

66.77 41.6667

66.78 41.6667

66.79 33.3333

66.8 66.6667

66.81 41.6667

66.82 33.3333

66.83 50

66.84 58.3333

66.85 8.33333

66.86 58.3333

66.87 41.6667

66.88 16.6667

66.89 58.3333

66.9 58.3333

66.91 75

66.92 25

66.93 75

66.94 75

66.95 41.6667

66.96 33.3333

66.97 66.6667

66.98 58.3333

66.99 58.3333

67 75

67.01 83.3333

67.02 83.3333

67.03 41.6667

67.04 33.3333

67.05 16.6667

67.06 41.6667

67.07 66.6667

67.08 58.3333

67.09 58.3333

67.1 41.6667

67.11 50

67.12 25

67.13 50

67.14 8.33333

67.15 33.3333

67.16 83.3333

67.17 25

67.18 50

67.19 25

67.2 50

67.21 58.3333

67.22 50

67.23 50

67.24 66.6667

67.25 16.6667

67.26 41.6667

67.27 75

67.28 41.6667

67.29 8.33333

67.3 50

67.31 58.3333

67.32 16.6667

67.33 25

67.34 66.6667

67.35 33.3333

67.36 16.6667

67.37 25

67.38 33.3333

67.39 58.3333

67.4 50

67.41 75

67.42 58.3333

67.43 75

67.44 41.6667

67.45 33.3333

67.46 75

67.47 50

67.48 66.6667

67.49 33.3333

67.5 25

67.51 58.3333

67.52 41.6667

67.53 33.3333

67.54 33.3333

67.55 58.3333

67.56 83.3333

67.57 33.3333

67.58 41.6667

67.59 33.3333

67.6 58.3333

67.61 41.6667

67.62 33.3333

67.63 58.3333

67.64 41.6667

67.65 58.3333

67.66 41.6667

67.67 50

67.68 50

67.69 83.3333

67.7 58.3333

67.71 75

67.72 50

67.73 58.3333

67.74 58.3333

67.75 16.6667

67.76 50

67.77 25

67.78 58.3333

67.79 100

67.8 33.3333

67.81 16.6667

67.82 66.6667

67.83 66.6667

67.84 41.6667

67.85 75

67.86 25

67.87 50

67.88 25

67.89 25

67.9 91.6667

67.91 25

67.92 25

67.93 25

67.94 58.3333

67.95 41.6667

67.96 16.6667

67.97 25

67.98 50

67.99 50

68 25

68.01 8.33333

68.02 33.3333

68.03 25

68.04 8.33333

68.05 33.3333

68.06 16.6667

68.07 58.3333

68.08 50

68.09 41.6667

68.1 16.6667

68.11 66.6667

68.12 41.6667

68.13 41.6667

68.14 58.3333

68.15 50

68.16 91.6667

68.17 25

68.18 66.6667

68.19 75

68.2 75

68.21 41.6667

68.22 50

68.23 58.3333

68.24 25

68.25 66.6667

68.26 33.3333

68.27 33.3333

68.28 100

68.29 33.3333

68.3 0

68.31 16.6667

68.32 41.6667

68.33 50

68.34 25

68.35 50

68.36 33.3333

68.37 50

68.38 50

68.39 25

68.4 41.6667

68.41 50

68.42 33.3333

68.43 33.3333

68.44 25

68.45 41.6667

68.46 33.3333

68.47 58.3333

68.48 0

68.49 66.6667

68.5 25

68.51 50

68.52 58.3333

68.53 50

68.54 50

68.55 58.3333

68.56 25

68.57 25

68.58 25

68.59 50

68.6 58.3333

68.61 16.6667

68.62 75

68.63 66.6667

68.64 50

68.65 66.6667

68.66 41.6667

68.67 8.33333

68.68 50

68.69 41.6667

68.7 25

68.71 25

68.72 58.3333

68.73 33.3333

68.74 41.6667

68.75 25

68.76 58.3333

68.77 41.6667

68.78 41.6667

68.79 33.3333

68.8 33.3333

68.81 33.3333

68.82 58.3333

68.83 58.3333

68.84 33.3333

68.85 41.6667

68.86 66.6667

68.87 41.6667

68.88 41.6667

68.89 16.6667

68.9 41.6667

68.91 41.6667

68.92 25

68.93 25

68.94 41.6667

68.95 16.6667

68.96 33.3333

68.97 83.3333

68.98 25

68.99 33.3333

69 50

69.01 16.6667

69.02 25

69.03 58.3333

69.04 58.3333

69.05 50

69.06 58.3333

69.07 50

69.08 25

69.09 41.6667

69.1 50

69.11 25

69.12 58.3333

69.13 41.6667

69.14 25

69.15 33.3333

69.16 50

69.17 33.3333

69.18 41.6667

69.19 41.6667

69.2 66.6667

69.21 50

69.22 58.3333

69.23 66.6667

69.24 8.33333

69.25 33.3333

69.26 25

69.27 8.33333

69.28 25

69.29 33.3333

69.3 25

69.31 66.6667

69.32 16.6667

69.33 58.3333

69.34 50

69.35 41.6667

69.36 25

69.37 25

69.38 33.3333

69.39 58.3333

69.4 83.3333

69.41 50

69.42 16.6667

69.43 33.3333

69.44 41.6667

69.45 41.6667

69.46 16.6667

69.47 33.3333

69.48 25

69.49 58.3333

69.5 16.6667

69.51 58.3333

69.52 41.6667

69.53 58.3333

69.54 41.6667

69.55 66.6667

69.56 58.3333

69.57 16.6667

69.58 50

69.59 41.6667

69.6 16.6667

69.61 66.6667

69.62 25

69.63 58.3333

69.64 25

69.65 25

69.66 91.6667

69.67 8.33333

69.68 25

69.69 50

69.7 8.33333

69.71 58.3333

69.72 50

69.73 50

69.74 33.3333

69.75 66.6667

69.76 41.6667

69.77 33.3333

69.78 41.6667

69.79 33.3333

69.8 33.3333

69.81 33.3333

69.82 25

69.83 33.3333

69.84 41.6667

69.85 41.6667

69.86 66.6667

69.87 41.6667

69.88 50

69.89 33.3333

69.9 50

69.91 25

69.92 25

69.93 50

69.94 66.6667

69.95 33.3333

69.96 25

69.97 50

69.98 66.6667

69.99 25

70 41.6667

**Raw data 2**. XRD raw data of the powder obtained after heat-treatment of as-sprayed powders prepared from the solution with citric acid at 450 ℃.

Goniometer RINT2000 vertical goniometer

Attachment Auto sample changer type B(6 samples)

Monochromater Fixed Monochromator

ScanningMode 2Theta/Theta

ScanningType Continuos Scanning

X-Ray 40kV/100mA

DivSlit 1 deg.

DivH.L.Slit 10mm

SctSlit 1 deg.

RecSlit 0.15mm

Monochro RS No Use

K-beta filter

Start 20

Stop 70

Step 0.01

20 308.333

20.01 300

20.02 241.667

20.03 208.333

20.04 358.333

20.05 325

20.06 391.667

20.07 308.333

20.08 408.333

20.09 383.333

20.1 308.333

20.11 308.333

20.12 291.667

20.13 300

20.14 341.667

20.15 283.333

20.16 266.667

20.17 308.333

20.18 308.333

20.19 283.333

20.2 225

20.21 266.667

20.22 241.667

20.23 316.667

20.24 266.667

20.25 441.667

20.26 283.333

20.27 291.667

20.28 225

20.29 258.333

20.3 316.667

20.31 341.667

20.32 325

20.33 266.667

20.34 358.333

20.35 300

20.36 191.667

20.37 333.333

20.38 383.333

20.39 283.333

20.4 250

20.41 358.333

20.42 316.667

20.43 350

20.44 266.667

20.45 408.333

20.46 333.333

20.47 325

20.48 225

20.49 300

20.5 316.667

20.51 291.667

20.52 275

20.53 408.333

20.54 383.333

20.55 283.333

20.56 241.667

20.57 333.333

20.58 358.333

20.59 316.667

20.6 266.667

20.61 341.667

20.62 358.333

20.63 325

20.64 325

20.65 266.667

20.66 333.333

20.67 300

20.68 291.667

20.69 291.667

20.7 358.333

20.71 391.667

20.72 300

20.73 341.667

20.74 300

20.75 333.333

20.76 358.333

20.77 325

20.78 291.667

20.79 366.667

20.8 375

20.81 283.333

20.82 450

20.83 258.333

20.84 333.333

20.85 291.667

20.86 400

20.87 466.667

20.88 308.333

20.89 266.667

20.9 333.333

20.91 350

20.92 375

20.93 333.333

20.94 416.667

20.95 333.333

20.96 358.333

20.97 250

20.98 366.667

20.99 308.333

21 400

21.01 366.667

21.02 291.667

21.03 266.667

21.04 308.333

21.05 308.333

21.06 275

21.07 241.667

21.08 316.667

21.09 341.667

21.1 316.667

21.11 375

21.12 316.667

21.13 291.667

21.14 283.333

21.15 258.333

21.16 291.667

21.17 200

21.18 300

21.19 416.667

21.2 358.333

21.21 283.333

21.22 333.333

21.23 308.333

21.24 233.333

21.25 241.667

21.26 350

21.27 400

21.28 283.333

21.29 416.667

21.3 316.667

21.31 358.333

21.32 250

21.33 283.333

21.34 316.667

21.35 283.333

21.36 366.667

21.37 341.667

21.38 275

21.39 366.667

21.4 375

21.41 283.333

21.42 391.667

21.43 233.333

21.44 316.667

21.45 283.333

21.46 316.667

21.47 233.333

21.48 416.667

21.49 225

21.5 258.333

21.51 266.667

21.52 291.667

21.53 300

21.54 333.333

21.55 225

21.56 275

21.57 275

21.58 283.333

21.59 366.667

21.6 275

21.61 233.333

21.62 316.667

21.63 350

21.64 333.333

21.65 283.333

21.66 258.333

21.67 316.667

21.68 258.333

21.69 383.333

21.7 375

21.71 358.333

21.72 375

21.73 208.333

21.74 283.333

21.75 391.667

21.76 266.667

21.77 341.667

21.78 325

21.79 225

21.8 425

21.81 258.333

21.82 250

21.83 291.667

21.84 283.333

21.85 250

21.86 191.667

21.87 358.333

21.88 258.333

21.89 291.667

21.9 316.667

21.91 341.667

21.92 308.333

21.93 250

21.94 366.667

21.95 308.333

21.96 200

21.97 341.667

21.98 283.333

21.99 308.333

22 283.333

22.01 316.667

22.02 258.333

22.03 283.333

22.04 308.333

22.05 308.333

22.06 333.333

22.07 191.667

22.08 283.333

22.09 408.333

22.1 341.667

22.11 358.333

22.12 283.333

22.13 291.667

22.14 266.667

22.15 150

22.16 300

22.17 291.667

22.18 266.667

22.19 325

22.2 291.667

22.21 325

22.22 191.667

22.23 225

22.24 358.333

22.25 291.667

22.26 333.333

22.27 325

22.28 275

22.29 266.667

22.3 300

22.31 325

22.32 308.333

22.33 233.333

22.34 266.667

22.35 308.333

22.36 291.667

22.37 325

22.38 350

22.39 233.333

22.4 316.667

22.41 308.333

22.42 366.667

22.43 250

22.44 308.333

22.45 275

22.46 208.333

22.47 283.333

22.48 266.667

22.49 266.667

22.5 233.333

22.51 158.333

22.52 308.333

22.53 341.667

22.54 308.333

22.55 233.333

22.56 316.667

22.57 266.667

22.58 241.667

22.59 283.333

22.6 300

22.61 258.333

22.62 200

22.63 291.667

22.64 208.333

22.65 241.667

22.66 283.333

22.67 316.667

22.68 325

22.69 183.333

22.7 233.333

22.71 300

22.72 350

22.73 241.667

22.74 291.667

22.75 383.333

22.76 266.667

22.77 291.667

22.78 275

22.79 241.667

22.8 358.333

22.81 300

22.82 350

22.83 300

22.84 291.667

22.85 300

22.86 200

22.87 258.333

22.88 341.667

22.89 166.667

22.9 200

22.91 350

22.92 183.333

22.93 225

22.94 233.333

22.95 258.333

22.96 225

22.97 333.333

22.98 366.667

22.99 216.667

23 258.333

23.01 250

23.02 366.667

23.03 325

23.04 166.667

23.05 325

23.06 258.333

23.07 325

23.08 241.667

23.09 200

23.1 233.333

23.11 266.667

23.12 266.667

23.13 241.667

23.14 333.333

23.15 258.333

23.16 216.667

23.17 241.667

23.18 291.667

23.19 158.333

23.2 250

23.21 375

23.22 233.333

23.23 283.333

23.24 300

23.25 216.667

23.26 300

23.27 333.333

23.28 291.667

23.29 191.667

23.3 266.667

23.31 316.667

23.32 283.333

23.33 258.333

23.34 275

23.35 191.667

23.36 191.667

23.37 241.667

23.38 225

23.39 275

23.4 175

23.41 258.333

23.42 175

23.43 250

23.44 225

23.45 250

23.46 275

23.47 216.667

23.48 225

23.49 283.333

23.5 300

23.51 241.667

23.52 266.667

23.53 175

23.54 241.667

23.55 200

23.56 191.667

23.57 258.333

23.58 225

23.59 266.667

23.6 258.333

23.61 233.333

23.62 175

23.63 283.333

23.64 258.333

23.65 341.667

23.66 258.333

23.67 166.667

23.68 308.333

23.69 191.667

23.7 283.333

23.71 258.333

23.72 250

23.73 275

23.74 266.667

23.75 258.333

23.76 241.667

23.77 233.333

23.78 208.333

23.79 166.667

23.8 225

23.81 266.667

23.82 183.333

23.83 166.667

23.84 241.667

23.85 225

23.86 141.667

23.87 183.333

23.88 233.333

23.89 291.667

23.9 225

23.91 241.667

23.92 250

23.93 150

23.94 225

23.95 200

23.96 266.667

23.97 191.667

23.98 150

23.99 175

24 191.667

24.01 225

24.02 250

24.03 133.333

24.04 258.333

24.05 250

24.06 275

24.07 225

24.08 258.333

24.09 191.667

24.1 250

24.11 266.667

24.12 300

24.13 166.667

24.14 225

24.15 216.667

24.16 216.667

24.17 241.667

24.18 283.333

24.19 200

24.2 233.333

24.21 141.667

24.22 216.667

24.23 283.333

24.24 216.667

24.25 283.333

24.26 216.667

24.27 241.667

24.28 183.333

24.29 325

24.3 250

24.31 208.333

24.32 158.333

24.33 175

24.34 233.333

24.35 250

24.36 241.667

24.37 200

24.38 216.667

24.39 250

24.4 241.667

24.41 183.333

24.42 216.667

24.43 241.667

24.44 175

24.45 258.333

24.46 216.667

24.47 183.333

24.48 308.333

24.49 241.667

24.5 200

24.51 133.333

24.52 191.667

24.53 133.333

24.54 241.667

24.55 225

24.56 150

24.57 200

24.58 216.667

24.59 191.667

24.6 191.667

24.61 241.667

24.62 183.333

24.63 241.667

24.64 191.667

24.65 208.333

24.66 125

24.67 225

24.68 258.333

24.69 166.667

24.7 225

24.71 208.333

24.72 200

24.73 300

24.74 191.667

24.75 233.333

24.76 166.667

24.77 208.333

24.78 216.667

24.79 175

24.8 208.333

24.81 216.667

24.82 258.333

24.83 241.667

24.84 216.667

24.85 183.333

24.86 175

24.87 166.667

24.88 216.667

24.89 233.333

24.9 175

24.91 166.667

24.92 166.667

24.93 216.667

24.94 166.667

24.95 200

24.96 241.667

24.97 166.667

24.98 133.333

24.99 175

25 225

25.01 125

25.02 166.667

25.03 158.333

25.04 216.667

25.05 191.667

25.06 266.667

25.07 200

25.08 175

25.09 150

25.1 250

25.11 191.667

25.12 216.667

25.13 175

25.14 116.667

25.15 233.333

25.16 208.333

25.17 125

25.18 133.333

25.19 175

25.2 200

25.21 216.667

25.22 241.667

25.23 175

25.24 175

25.25 225

25.26 100

25.27 166.667

25.28 158.333

25.29 150

25.3 241.667

25.31 183.333

25.32 191.667

25.33 125

25.34 166.667

25.35 150

25.36 141.667

25.37 216.667

25.38 191.667

25.39 141.667

25.4 150

25.41 175

25.42 208.333

25.43 208.333

25.44 141.667

25.45 191.667

25.46 183.333

25.47 125

25.48 150

25.49 258.333

25.5 166.667

25.51 208.333

25.52 141.667

25.53 183.333

25.54 191.667

25.55 141.667

25.56 200

25.57 225

25.58 166.667

25.59 158.333

25.6 175

25.61 258.333

25.62 183.333

25.63 208.333

25.64 150

25.65 266.667

25.66 183.333

25.67 116.667

25.68 208.333

25.69 158.333

25.7 225

25.71 150

25.72 75

25.73 133.333

25.74 183.333

25.75 175

25.76 166.667

25.77 166.667

25.78 125

25.79 166.667

25.8 150

25.81 91.6667

25.82 183.333

25.83 158.333

25.84 175

25.85 175

25.86 158.333

25.87 150

25.88 175

25.89 166.667

25.9 175

25.91 125

25.92 225

25.93 183.333

25.94 191.667

25.95 141.667

25.96 208.333

25.97 125

25.98 175

25.99 241.667

26 200

26.01 183.333

26.02 141.667

26.03 150

26.04 100

26.05 241.667

26.06 83.3333

26.07 150

26.08 225

26.09 191.667

26.1 158.333

26.11 91.6667

26.12 166.667

26.13 150

26.14 183.333

26.15 183.333

26.16 158.333

26.17 125

26.18 125

26.19 116.667

26.2 133.333

26.21 183.333

26.22 150

26.23 183.333

26.24 133.333

26.25 125

26.26 75

26.27 200

26.28 208.333

26.29 166.667

26.3 125

26.31 191.667

26.32 208.333

26.33 166.667

26.34 116.667

26.35 183.333

26.36 150

26.37 183.333

26.38 100

26.39 158.333

26.4 91.6667

26.41 133.333

26.42 233.333

26.43 200

26.44 175

26.45 116.667

26.46 175

26.47 141.667

26.48 175

26.49 125

26.5 125

26.51 125

26.52 133.333

26.53 158.333

26.54 158.333

26.55 166.667

26.56 141.667

26.57 166.667

26.58 125

26.59 191.667

26.6 191.667

26.61 116.667

26.62 175

26.63 83.3333

26.64 200

26.65 125

26.66 141.667

26.67 158.333

26.68 150

26.69 216.667

26.7 133.333

26.71 183.333

26.72 225

26.73 150

26.74 100

26.75 133.333

26.76 158.333

26.77 150

26.78 125

26.79 100

26.8 158.333

26.81 150

26.82 125

26.83 125

26.84 175

26.85 150

26.86 191.667

26.87 166.667

26.88 125

26.89 141.667

26.9 200

26.91 183.333

26.92 216.667

26.93 133.333

26.94 200

26.95 150

26.96 133.333

26.97 83.3333

26.98 150

26.99 116.667

27 133.333

27.01 141.667

27.02 183.333

27.03 200

27.04 200

27.05 125

27.06 166.667

27.07 108.333

27.08 108.333

27.09 141.667

27.1 150

27.11 150

27.12 91.6667

27.13 66.6667

27.14 133.333

27.15 83.3333

27.16 141.667

27.17 166.667

27.18 158.333

27.19 108.333

27.2 158.333

27.21 150

27.22 100

27.23 125

27.24 91.6667

27.25 75

27.26 75

27.27 133.333

27.28 116.667

27.29 166.667

27.3 116.667

27.31 100

27.32 175

27.33 141.667

27.34 141.667

27.35 166.667

27.36 200

27.37 100

27.38 133.333

27.39 141.667

27.4 191.667

27.41 150

27.42 216.667

27.43 183.333

27.44 150

27.45 150

27.46 141.667

27.47 158.333

27.48 191.667

27.49 108.333

27.5 150

27.51 108.333

27.52 183.333

27.53 100

27.54 116.667

27.55 158.333

27.56 50

27.57 125

27.58 100

27.59 141.667

27.6 100

27.61 166.667

27.62 125

27.63 158.333

27.64 116.667

27.65 175

27.66 141.667

27.67 150

27.68 191.667

27.69 166.667

27.7 158.333

27.71 216.667

27.72 108.333

27.73 141.667

27.74 125

27.75 108.333

27.76 116.667

27.77 141.667

27.78 150

27.79 108.333

27.8 116.667

27.81 133.333

27.82 141.667

27.83 133.333

27.84 116.667

27.85 141.667

27.86 133.333

27.87 166.667

27.88 200

27.89 141.667

27.9 108.333

27.91 175

27.92 100

27.93 166.667

27.94 108.333

27.95 108.333

27.96 133.333

27.97 116.667

27.98 100

27.99 175

28 116.667

28.01 108.333

28.02 166.667

28.03 116.667

28.04 133.333

28.05 133.333

28.06 125

28.07 166.667

28.08 100

28.09 108.333

28.1 141.667

28.11 58.3333

28.12 100

28.13 75

28.14 166.667

28.15 141.667

28.16 150

28.17 125

28.18 141.667

28.19 125

28.2 125

28.21 141.667

28.22 141.667

28.23 100

28.24 125

28.25 100

28.26 183.333

28.27 166.667

28.28 116.667

28.29 75

28.3 141.667

28.31 141.667

28.32 141.667

28.33 150

28.34 100

28.35 100

28.36 175

28.37 150

28.38 158.333

28.39 108.333

28.4 108.333

28.41 141.667

28.42 108.333

28.43 100

28.44 100

28.45 125

28.46 133.333

28.47 141.667

28.48 133.333

28.49 141.667

28.5 133.333

28.51 141.667

28.52 133.333

28.53 125

28.54 83.3333

28.55 166.667

28.56 116.667

28.57 150

28.58 108.333

28.59 200

28.6 100

28.61 116.667

28.62 116.667

28.63 166.667

28.64 116.667

28.65 58.3333

28.66 183.333

28.67 200

28.68 133.333

28.69 158.333

28.7 91.6667

28.71 150

28.72 75

28.73 91.6667

28.74 158.333

28.75 150

28.76 116.667

28.77 100

28.78 66.6667

28.79 100

28.8 208.333

28.81 83.3333

28.82 150

28.83 158.333

28.84 91.6667

28.85 125

28.86 116.667

28.87 133.333

28.88 133.333

28.89 150

28.9 141.667

28.91 141.667

28.92 83.3333

28.93 83.3333

28.94 100

28.95 116.667

28.96 141.667

28.97 125

28.98 158.333

28.99 150

29 141.667

29.01 141.667

29.02 141.667

29.03 108.333

29.04 183.333

29.05 133.333

29.06 91.6667

29.07 175

29.08 166.667

29.09 191.667

29.1 91.6667

29.11 158.333

29.12 91.6667

29.13 141.667

29.14 158.333

29.15 100

29.16 125

29.17 125

29.18 141.667

29.19 100

29.2 116.667

29.21 141.667

29.22 116.667

29.23 108.333

29.24 183.333

29.25 83.3333

29.26 175

29.27 141.667

29.28 125

29.29 166.667

29.3 141.667

29.31 200

29.32 91.6667

29.33 83.3333

29.34 116.667

29.35 133.333

29.36 175

29.37 158.333

29.38 116.667

29.39 66.6667

29.4 125

29.41 200

29.42 125

29.43 91.6667

29.44 100

29.45 133.333

29.46 166.667

29.47 100

29.48 66.6667

29.49 83.3333

29.5 150

29.51 141.667

29.52 108.333

29.53 75

29.54 158.333

29.55 150

29.56 108.333

29.57 133.333

29.58 116.667

29.59 83.3333

29.6 100

29.61 116.667

29.62 108.333

29.63 150

29.64 133.333

29.65 83.3333

29.66 125

29.67 66.6667

29.68 116.667

29.69 125

29.7 83.3333

29.71 83.3333

29.72 91.6667

29.73 200

29.74 116.667

29.75 116.667

29.76 141.667

29.77 150

29.78 100

29.79 133.333

29.8 100

29.81 133.333

29.82 83.3333

29.83 125

29.84 116.667

29.85 100

29.86 116.667

29.87 158.333

29.88 108.333

29.89 116.667

29.9 150

29.91 150

29.92 116.667

29.93 116.667

29.94 158.333

29.95 91.6667

29.96 125

29.97 150

29.98 91.6667

29.99 166.667

30 116.667

30.01 191.667

30.02 125

30.03 83.3333

30.04 108.333

30.05 141.667

30.06 141.667

30.07 166.667

30.08 141.667

30.09 58.3333

30.1 175

30.11 150

30.12 66.6667

30.13 83.3333

30.14 175

30.15 166.667

30.16 75

30.17 75

30.18 133.333

30.19 133.333

30.2 150

30.21 183.333

30.22 133.333

30.23 158.333

30.24 75

30.25 100

30.26 125

30.27 175

30.28 116.667

30.29 91.6667

30.3 91.6667

30.31 100

30.32 125

30.33 116.667

30.34 125

30.35 83.3333

30.36 100

30.37 133.333

30.38 166.667

30.39 83.3333

30.4 116.667

30.41 158.333

30.42 158.333

30.43 125

30.44 141.667

30.45 116.667

30.46 191.667

30.47 91.6667

30.48 125

30.49 83.3333

30.5 116.667

30.51 108.333

30.52 75

30.53 75

30.54 83.3333

30.55 125

30.56 91.6667

30.57 108.333

30.58 208.333

30.59 141.667

30.6 100

30.61 166.667

30.62 158.333

30.63 83.3333

30.64 91.6667

30.65 166.667

30.66 83.3333

30.67 66.6667

30.68 108.333

30.69 133.333

30.7 125

30.71 141.667

30.72 250

30.73 125

30.74 116.667

30.75 183.333

30.76 58.3333

30.77 100

30.78 175

30.79 58.3333

30.8 116.667

30.81 125

30.82 108.333

30.83 150

30.84 108.333

30.85 91.6667

30.86 125

30.87 150

30.88 108.333

30.89 108.333

30.9 108.333

30.91 175

30.92 125

30.93 66.6667

30.94 66.6667

30.95 116.667

30.96 108.333

30.97 91.6667

30.98 100

30.99 166.667

31 150

31.01 116.667

31.02 108.333

31.03 100

31.04 133.333

31.05 83.3333

31.06 75

31.07 91.6667

31.08 133.333

31.09 108.333

31.1 116.667

31.11 91.6667

31.12 116.667

31.13 125

31.14 166.667

31.15 108.333

31.16 100

31.17 150

31.18 125

31.19 108.333

31.2 108.333

31.21 91.6667

31.22 125

31.23 66.6667

31.24 108.333

31.25 75

31.26 125

31.27 150

31.28 158.333

31.29 150

31.3 100

31.31 116.667

31.32 133.333

31.33 75

31.34 66.6667

31.35 216.667

31.36 133.333

31.37 108.333

31.38 100

31.39 150

31.4 108.333

31.41 108.333

31.42 158.333

31.43 116.667

31.44 75

31.45 108.333

31.46 100

31.47 116.667

31.48 166.667

31.49 108.333

31.5 58.3333

31.51 100

31.52 133.333

31.53 66.6667

31.54 83.3333

31.55 141.667

31.56 125

31.57 66.6667

31.58 100

31.59 91.6667

31.6 116.667

31.61 58.3333

31.62 83.3333

31.63 100

31.64 108.333

31.65 150

31.66 175

31.67 66.6667

31.68 91.6667

31.69 91.6667

31.7 108.333

31.71 108.333

31.72 83.3333

31.73 108.333

31.74 83.3333

31.75 66.6667

31.76 100

31.77 100

31.78 108.333

31.79 125

31.8 125

31.81 75

31.82 108.333

31.83 141.667

31.84 166.667

31.85 166.667

31.86 125

31.87 116.667

31.88 125

31.89 116.667

31.9 91.6667

31.91 116.667

31.92 108.333

31.93 125

31.94 50

31.95 66.6667

31.96 58.3333

31.97 91.6667

31.98 100

31.99 133.333

32 100

32.01 116.667

32.02 108.333

32.03 108.333

32.04 108.333

32.05 91.6667

32.06 125

32.07 75

32.08 58.3333

32.09 141.667

32.1 100

32.11 150

32.12 116.667

32.13 166.667

32.14 50

32.15 175

32.16 100

32.17 158.333

32.18 75

32.19 83.3333

32.2 150

32.21 141.667

32.22 100

32.23 66.6667

32.24 41.6667

32.25 91.6667

32.26 91.6667

32.27 141.667

32.28 33.3333

32.29 75

32.3 150

32.31 125

32.32 83.3333

32.33 108.333

32.34 108.333

32.35 66.6667

32.36 108.333

32.37 100

32.38 83.3333

32.39 108.333

32.4 125

32.41 75

32.42 58.3333

32.43 116.667

32.44 175

32.45 125

32.46 133.333

32.47 75

32.48 125

32.49 58.3333

32.5 75

32.51 75

32.52 116.667

32.53 100

32.54 75

32.55 108.333

32.56 141.667

32.57 91.6667

32.58 125

32.59 66.6667

32.6 66.6667

32.61 83.3333

32.62 100

32.63 133.333

32.64 125

32.65 133.333

32.66 83.3333

32.67 50

32.68 108.333

32.69 108.333

32.7 100

32.71 91.6667

32.72 100

32.73 75

32.74 166.667

32.75 66.6667

32.76 166.667

32.77 116.667

32.78 83.3333

32.79 66.6667

32.8 75

32.81 50

32.82 133.333

32.83 116.667

32.84 33.3333

32.85 100

32.86 50

32.87 58.3333

32.88 66.6667

32.89 91.6667

32.9 75

32.91 66.6667

32.92 116.667

32.93 116.667

32.94 66.6667

32.95 91.6667

32.96 58.3333

32.97 150

32.98 125

32.99 100

33 100

33.01 108.333

33.02 108.333

33.03 75

33.04 108.333

33.05 91.6667

33.06 91.6667

33.07 91.6667

33.08 91.6667

33.09 100

33.1 66.6667

33.11 116.667

33.12 108.333

33.13 133.333

33.14 66.6667

33.15 108.333

33.16 58.3333

33.17 125

33.18 83.3333

33.19 66.6667

33.2 91.6667

33.21 91.6667

33.22 66.6667

33.23 116.667

33.24 91.6667

33.25 83.3333

33.26 83.3333

33.27 133.333

33.28 150

33.29 91.6667

33.3 116.667

33.31 150

33.32 133.333

33.33 75

33.34 108.333

33.35 100

33.36 125

33.37 91.6667

33.38 100

33.39 116.667

33.4 116.667

33.41 116.667

33.42 100

33.43 75

33.44 158.333

33.45 41.6667

33.46 66.6667

33.47 108.333

33.48 125

33.49 100

33.5 125

33.51 141.667

33.52 50

33.53 150

33.54 116.667

33.55 100

33.56 75

33.57 100

33.58 100

33.59 116.667

33.6 141.667

33.61 116.667

33.62 100

33.63 108.333

33.64 108.333

33.65 83.3333

33.66 150

33.67 125

33.68 108.333

33.69 116.667

33.7 133.333

33.71 83.3333

33.72 100

33.73 158.333

33.74 133.333

33.75 75

33.76 108.333

33.77 83.3333

33.78 108.333

33.79 91.6667

33.8 158.333

33.81 58.3333

33.82 150

33.83 91.6667

33.84 83.3333

33.85 58.3333

33.86 133.333

33.87 125

33.88 150

33.89 108.333

33.9 91.6667

33.91 83.3333

33.92 83.3333

33.93 108.333

33.94 108.333

33.95 133.333

33.96 91.6667

33.97 83.3333

33.98 166.667

33.99 91.6667

34 66.6667

34.01 75

34.02 91.6667

34.03 91.6667

34.04 100

34.05 83.3333

34.06 100

34.07 91.6667

34.08 75

34.09 83.3333

34.1 33.3333

34.11 91.6667

34.12 141.667

34.13 100

34.14 91.6667

34.15 100

34.16 91.6667

34.17 166.667

34.18 133.333

34.19 125

34.2 133.333

34.21 75

34.22 125

34.23 83.3333

34.24 133.333

34.25 141.667

34.26 116.667

34.27 133.333

34.28 100

34.29 133.333

34.3 83.3333

34.31 100

34.32 133.333

34.33 141.667

34.34 133.333

34.35 200

34.36 166.667

34.37 116.667

34.38 133.333

34.39 150

34.4 100

34.41 125

34.42 133.333

34.43 108.333

34.44 125

34.45 125

34.46 58.3333

34.47 100

34.48 125

34.49 50

34.5 75

34.51 91.6667

34.52 83.3333

34.53 83.3333

34.54 175

34.55 116.667

34.56 150

34.57 108.333

34.58 108.333

34.59 125

34.6 108.333

34.61 91.6667

34.62 50

34.63 141.667

34.64 125

34.65 166.667

34.66 108.333

34.67 91.6667

34.68 108.333

34.69 58.3333

34.7 133.333

34.71 133.333

34.72 116.667

34.73 125

34.74 91.6667

34.75 125

34.76 183.333

34.77 141.667

34.78 141.667

34.79 175

34.8 183.333

34.81 125

34.82 200

34.83 158.333

34.84 83.3333

34.85 225

34.86 141.667

34.87 158.333

34.88 141.667

34.89 108.333

34.9 141.667

34.91 150

34.92 133.333

34.93 91.6667

34.94 150

34.95 158.333

34.96 150

34.97 191.667

34.98 133.333

34.99 183.333

35 183.333

35.01 158.333

35.02 125

35.03 166.667

35.04 116.667

35.05 100

35.06 158.333

35.07 66.6667

35.08 125

35.09 133.333

35.1 166.667

35.11 166.667

35.12 150

35.13 141.667

35.14 108.333

35.15 150

35.16 150

35.17 108.333

35.18 108.333

35.19 125

35.2 216.667

35.21 108.333

35.22 141.667

35.23 200

35.24 150

35.25 166.667

35.26 91.6667

35.27 208.333

35.28 158.333

35.29 158.333

35.3 183.333

35.31 158.333

35.32 141.667

35.33 150

35.34 116.667

35.35 191.667

35.36 141.667

35.37 133.333

35.38 133.333

35.39 225

35.4 233.333

35.41 125

35.42 150

35.43 150

35.44 150

35.45 166.667

35.46 75

35.47 166.667

35.48 175

35.49 191.667

35.5 241.667

35.51 158.333

35.52 150

35.53 141.667

35.54 166.667

35.55 158.333

35.56 133.333

35.57 175

35.58 183.333

35.59 175

35.6 116.667

35.61 191.667

35.62 150

35.63 125

35.64 108.333

35.65 133.333

35.66 233.333

35.67 150

35.68 233.333

35.69 125

35.7 225

35.71 183.333

35.72 108.333

35.73 158.333

35.74 208.333

35.75 150

35.76 133.333

35.77 141.667

35.78 100

35.79 150

35.8 166.667

35.81 150

35.82 158.333

35.83 108.333

35.84 125

35.85 133.333

35.86 191.667

35.87 108.333

35.88 116.667

35.89 133.333

35.9 141.667

35.91 158.333

35.92 75

35.93 108.333

35.94 100

35.95 83.3333

35.96 158.333

35.97 125

35.98 175

35.99 108.333

36 58.3333

36.01 125

36.02 141.667

36.03 133.333

36.04 150

36.05 125

36.06 83.3333

36.07 100

36.08 141.667

36.09 108.333

36.1 125

36.11 108.333

36.12 116.667

36.13 116.667

36.14 125

36.15 100

36.16 125

36.17 100

36.18 150

36.19 150

36.2 83.3333

36.21 100

36.22 58.3333

36.23 200

36.24 91.6667

36.25 100

36.26 108.333

36.27 108.333

36.28 75

36.29 100

36.3 66.6667

36.31 58.3333

36.32 100

36.33 141.667

36.34 116.667

36.35 116.667

36.36 75

36.37 141.667

36.38 133.333

36.39 175

36.4 100

36.41 116.667

36.42 108.333

36.43 100

36.44 133.333

36.45 91.6667

36.46 66.6667

36.47 100

36.48 83.3333

36.49 125

36.5 100

36.51 91.6667

36.52 91.6667

36.53 91.6667

36.54 141.667

36.55 108.333

36.56 83.3333

36.57 150

36.58 83.3333

36.59 125

36.6 116.667

36.61 100

36.62 91.6667

36.63 108.333

36.64 83.3333

36.65 66.6667

36.66 100

36.67 83.3333

36.68 100

36.69 91.6667

36.7 141.667

36.71 100

36.72 75

36.73 75

36.74 91.6667

36.75 166.667

36.76 100

36.77 141.667

36.78 75

36.79 100

36.8 66.6667

36.81 41.6667

36.82 141.667

36.83 108.333

36.84 100

36.85 66.6667

36.86 58.3333

36.87 150

36.88 133.333

36.89 150

36.9 66.6667

36.91 83.3333

36.92 75

36.93 91.6667

36.94 116.667

36.95 108.333

36.96 91.6667

36.97 75

36.98 108.333

36.99 116.667

37 91.6667

37.01 83.3333

37.02 33.3333

37.03 116.667

37.04 41.6667

37.05 91.6667

37.06 91.6667

37.07 116.667

37.08 75

37.09 100

37.1 91.6667

37.11 58.3333

37.12 83.3333

37.13 91.6667

37.14 75

37.15 125

37.16 83.3333

37.17 66.6667

37.18 58.3333

37.19 83.3333

37.2 83.3333

37.21 100

37.22 150

37.23 91.6667

37.24 83.3333

37.25 91.6667

37.26 100

37.27 108.333

37.28 100

37.29 91.6667

37.3 75

37.31 108.333

37.32 50

37.33 91.6667

37.34 75

37.35 91.6667

37.36 50

37.37 125

37.38 91.6667

37.39 100

37.4 41.6667

37.41 83.3333

37.42 125

37.43 66.6667

37.44 58.3333

37.45 58.3333

37.46 91.6667

37.47 141.667

37.48 58.3333

37.49 41.6667

37.5 58.3333

37.51 83.3333

37.52 108.333

37.53 91.6667

37.54 83.3333

37.55 100

37.56 66.6667

37.57 100

37.58 66.6667

37.59 83.3333

37.6 75

37.61 91.6667

37.62 33.3333

37.63 125

37.64 91.6667

37.65 83.3333

37.66 83.3333

37.67 58.3333

37.68 66.6667

37.69 33.3333

37.7 100

37.71 50

37.72 66.6667

37.73 75

37.74 66.6667

37.75 33.3333

37.76 125

37.77 16.6667

37.78 58.3333

37.79 83.3333

37.8 83.3333

37.81 100

37.82 83.3333

37.83 66.6667

37.84 116.667

37.85 100

37.86 83.3333

37.87 116.667

37.88 83.3333

37.89 75

37.9 75

37.91 66.6667

37.92 50

37.93 50

37.94 75

37.95 50

37.96 83.3333

37.97 83.3333

37.98 116.667

37.99 91.6667

38 100

38.01 58.3333

38.02 100

38.03 58.3333

38.04 133.333

38.05 75

38.06 66.6667

38.07 16.6667

38.08 91.6667

38.09 108.333

38.1 50

38.11 50

38.12 58.3333

38.13 50

38.14 50

38.15 100

38.16 83.3333

38.17 83.3333

38.18 58.3333

38.19 83.3333

38.2 83.3333

38.21 66.6667

38.22 41.6667

38.23 58.3333

38.24 50

38.25 66.6667

38.26 91.6667

38.27 66.6667

38.28 133.333

38.29 58.3333

38.3 41.6667

38.31 50

38.32 83.3333

38.33 50

38.34 50

38.35 50

38.36 75

38.37 75

38.38 41.6667

38.39 58.3333

38.4 50

38.41 108.333

38.42 100

38.43 50

38.44 83.3333

38.45 66.6667

38.46 66.6667

38.47 108.333

38.48 50

38.49 58.3333

38.5 50

38.51 91.6667

38.52 83.3333

38.53 83.3333

38.54 33.3333

38.55 75

38.56 100

38.57 116.667

38.58 100

38.59 16.6667

38.6 75

38.61 83.3333

38.62 66.6667

38.63 41.6667

38.64 91.6667

38.65 66.6667

38.66 75

38.67 75

38.68 58.3333

38.69 75

38.7 58.3333

38.71 41.6667

38.72 41.6667

38.73 50

38.74 50

38.75 66.6667

38.76 75

38.77 75

38.78 75

38.79 41.6667

38.8 66.6667

38.81 75

38.82 83.3333

38.83 58.3333

38.84 83.3333

38.85 91.6667

38.86 133.333

38.87 58.3333

38.88 58.3333

38.89 25

38.9 58.3333

38.91 75

38.92 75

38.93 58.3333

38.94 83.3333

38.95 66.6667

38.96 41.6667

38.97 58.3333

38.98 25

38.99 33.3333

39 66.6667

39.01 50

39.02 41.6667

39.03 66.6667

39.04 116.667

39.05 50

39.06 33.3333

39.07 83.3333

39.08 66.6667

39.09 58.3333

39.1 58.3333

39.11 33.3333

39.12 75

39.13 58.3333

39.14 66.6667

39.15 75

39.16 66.6667

39.17 41.6667

39.18 66.6667

39.19 58.3333

39.2 75

39.21 33.3333

39.22 58.3333

39.23 91.6667

39.24 58.3333

39.25 41.6667

39.26 91.6667

39.27 50

39.28 75

39.29 58.3333

39.3 66.6667

39.31 66.6667

39.32 91.6667

39.33 83.3333

39.34 33.3333

39.35 75

39.36 75

39.37 58.3333

39.38 33.3333

39.39 75

39.4 50

39.41 33.3333

39.42 66.6667

39.43 50

39.44 50

39.45 91.6667

39.46 25

39.47 50

39.48 58.3333

39.49 8.33333

39.5 83.3333

39.51 50

39.52 75

39.53 83.3333

39.54 58.3333

39.55 58.3333

39.56 58.3333

39.57 50

39.58 66.6667

39.59 50

39.6 41.6667

39.61 58.3333

39.62 83.3333

39.63 91.6667

39.64 58.3333

39.65 41.6667

39.66 125

39.67 25

39.68 41.6667

39.69 66.6667

39.7 41.6667

39.71 58.3333

39.72 83.3333

39.73 41.6667

39.74 50

39.75 58.3333

39.76 25

39.77 50

39.78 33.3333

39.79 58.3333

39.8 83.3333

39.81 33.3333

39.82 100

39.83 91.6667

39.84 50

39.85 91.6667

39.86 75

39.87 41.6667

39.88 41.6667

39.89 50

39.9 66.6667

39.91 50

39.92 50

39.93 83.3333

39.94 50

39.95 66.6667

39.96 58.3333

39.97 25

39.98 41.6667

39.99 75

40 58.3333

40.01 33.3333

40.02 58.3333

40.03 58.3333

40.04 41.6667

40.05 41.6667

40.06 50

40.07 108.333

40.08 33.3333

40.09 50

40.1 75

40.11 75

40.12 66.6667

40.13 66.6667

40.14 91.6667

40.15 75

40.16 83.3333

40.17 91.6667

40.18 83.3333

40.19 50

40.2 41.6667

40.21 58.3333

40.22 41.6667

40.23 33.3333

40.24 58.3333

40.25 16.6667

40.26 58.3333

40.27 83.3333

40.28 50

40.29 41.6667

40.3 83.3333

40.31 50

40.32 33.3333

40.33 33.3333

40.34 58.3333

40.35 50

40.36 75

40.37 41.6667

40.38 91.6667

40.39 83.3333

40.4 58.3333

40.41 58.3333

40.42 16.6667

40.43 25

40.44 75

40.45 33.3333

40.46 58.3333

40.47 66.6667

40.48 58.3333

40.49 50

40.5 25

40.51 66.6667

40.52 33.3333

40.53 58.3333

40.54 50

40.55 25

40.56 91.6667

40.57 50

40.58 83.3333

40.59 75

40.6 75

40.61 58.3333

40.62 66.6667

40.63 50

40.64 91.6667

40.65 41.6667

40.66 75

40.67 58.3333

40.68 58.3333

40.69 41.6667

40.7 33.3333

40.71 66.6667

40.72 91.6667

40.73 50

40.74 41.6667

40.75 75

40.76 41.6667

40.77 41.6667

40.78 58.3333

40.79 75

40.8 50

40.81 58.3333

40.82 33.3333

40.83 25

40.84 50

40.85 66.6667

40.86 58.3333

40.87 66.6667

40.88 50

40.89 50

40.9 16.6667

40.91 91.6667

40.92 25

40.93 58.3333

40.94 91.6667

40.95 50

40.96 50

40.97 25

40.98 91.6667

40.99 75

41 50

41.01 50

41.02 58.3333

41.03 75

41.04 58.3333

41.05 66.6667

41.06 50

41.07 91.6667

41.08 66.6667

41.09 50

41.1 108.333

41.11 41.6667

41.12 41.6667

41.13 58.3333

41.14 8.33333

41.15 50

41.16 58.3333

41.17 58.3333

41.18 50

41.19 41.6667

41.2 25

41.21 41.6667

41.22 66.6667

41.23 50

41.24 116.667

41.25 41.6667

41.26 33.3333

41.27 83.3333

41.28 33.3333

41.29 83.3333

41.3 91.6667

41.31 50

41.32 41.6667

41.33 33.3333

41.34 75

41.35 50

41.36 33.3333

41.37 83.3333

41.38 50

41.39 83.3333

41.4 33.3333

41.41 33.3333

41.42 33.3333

41.43 50

41.44 50

41.45 58.3333

41.46 66.6667

41.47 58.3333

41.48 100

41.49 50

41.5 83.3333

41.51 58.3333

41.52 33.3333

41.53 25

41.54 50

41.55 41.6667

41.56 66.6667

41.57 33.3333

41.58 16.6667

41.59 41.6667

41.6 75

41.61 41.6667

41.62 33.3333

41.63 41.6667

41.64 25

41.65 50

41.66 50

41.67 75

41.68 58.3333

41.69 25

41.7 58.3333

41.71 33.3333

41.72 50

41.73 58.3333

41.74 33.3333

41.75 50

41.76 41.6667

41.77 66.6667

41.78 58.3333

41.79 58.3333

41.8 41.6667

41.81 83.3333

41.82 58.3333

41.83 83.3333

41.84 25

41.85 66.6667

41.86 33.3333

41.87 66.6667

41.88 75

41.89 66.6667

41.9 50

41.91 66.6667

41.92 25

41.93 58.3333

41.94 16.6667

41.95 100

41.96 50

41.97 58.3333

41.98 91.6667

41.99 50

42 58.3333

42.01 83.3333

42.02 66.6667

42.03 75

42.04 83.3333

42.05 75

42.06 108.333

42.07 58.3333

42.08 58.3333

42.09 58.3333

42.1 50

42.11 50

42.12 50

42.13 133.333

42.14 66.6667

42.15 25

42.16 91.6667

42.17 41.6667

42.18 75

42.19 83.3333

42.2 91.6667

42.21 50

42.22 58.3333

42.23 91.6667

42.24 41.6667

42.25 50

42.26 41.6667

42.27 66.6667

42.28 83.3333

42.29 25

42.3 75

42.31 91.6667

42.32 91.6667

42.33 58.3333

42.34 75

42.35 58.3333

42.36 75

42.37 33.3333

42.38 83.3333

42.39 58.3333

42.4 66.6667

42.41 91.6667

42.42 75

42.43 58.3333

42.44 83.3333

42.45 66.6667

42.46 58.3333

42.47 58.3333

42.48 83.3333

42.49 108.333

42.5 75

42.51 66.6667

42.52 83.3333

42.53 91.6667

42.54 50

42.55 66.6667

42.56 66.6667

42.57 83.3333

42.58 50

42.59 75

42.6 91.6667

42.61 91.6667

42.62 108.333

42.63 83.3333

42.64 75

42.65 66.6667

42.66 66.6667

42.67 50

42.68 133.333

42.69 75

42.7 83.3333

42.71 41.6667

42.72 50

42.73 41.6667

42.74 75

42.75 75

42.76 58.3333

42.77 41.6667

42.78 108.333

42.79 116.667

42.8 75

42.81 58.3333

42.82 33.3333

42.83 100

42.84 75

42.85 83.3333

42.86 133.333

42.87 83.3333

42.88 100

42.89 125

42.9 125

42.91 108.333

42.92 83.3333

42.93 108.333

42.94 83.3333

42.95 83.3333

42.96 91.6667

42.97 91.6667

42.98 100

42.99 133.333

43 83.3333

43.01 83.3333

43.02 50

43.03 66.6667

43.04 83.3333

43.05 75

43.06 66.6667

43.07 100

43.08 91.6667

43.09 91.6667

43.1 50

43.11 100

43.12 100

43.13 75

43.14 66.6667

43.15 100

43.16 116.667

43.17 83.3333

43.18 75

43.19 141.667

43.2 116.667

43.21 75

43.22 75

43.23 83.3333

43.24 50

43.25 75

43.26 116.667

43.27 116.667

43.28 125

43.29 108.333

43.3 100

43.31 66.6667

43.32 50

43.33 91.6667

43.34 100

43.35 100

43.36 108.333

43.37 116.667

43.38 91.6667

43.39 100

43.4 133.333

43.41 83.3333

43.42 83.3333

43.43 58.3333

43.44 116.667

43.45 33.3333

43.46 108.333

43.47 66.6667

43.48 75

43.49 91.6667

43.5 58.3333

43.51 75

43.52 83.3333

43.53 91.6667

43.54 108.333

43.55 33.3333

43.56 83.3333

43.57 83.3333

43.58 83.3333

43.59 83.3333

43.6 75

43.61 100

43.62 75

43.63 100

43.64 108.333

43.65 91.6667

43.66 125

43.67 83.3333

43.68 108.333

43.69 141.667

43.7 83.3333

43.71 125

43.72 66.6667

43.73 75

43.74 100

43.75 75

43.76 91.6667

43.77 50

43.78 58.3333

43.79 66.6667

43.8 83.3333

43.81 100

43.82 91.6667

43.83 116.667

43.84 100

43.85 100

43.86 66.6667

43.87 25

43.88 83.3333

43.89 66.6667

43.9 116.667

43.91 133.333

43.92 116.667

43.93 108.333

43.94 66.6667

43.95 66.6667

43.96 50

43.97 100

43.98 75

43.99 75

44 116.667

44.01 66.6667

44.02 66.6667

44.03 108.333

44.04 66.6667

44.05 133.333

44.06 58.3333

44.07 125

44.08 25

44.09 75

44.1 66.6667

44.11 100

44.12 75

44.13 108.333

44.14 100

44.15 100

44.16 116.667

44.17 91.6667

44.18 100

44.19 100

44.2 41.6667

44.21 58.3333

44.22 150

44.23 116.667

44.24 125

44.25 116.667

44.26 133.333

44.27 175

44.28 116.667

44.29 166.667

44.3 58.3333

44.31 83.3333

44.32 108.333

44.33 208.333

44.34 200

44.35 208.333

44.36 175

44.37 200

44.38 150

44.39 175

44.4 233.333

44.41 258.333

44.42 291.667

44.43 208.333

44.44 250

44.45 266.667

44.46 325

44.47 258.333

44.48 325

44.49 400

44.5 333.333

44.51 383.333

44.52 366.667

44.53 608.333

44.54 750

44.55 566.667

44.56 750

44.57 716.667

44.58 741.667

44.59 808.333

44.6 1058.33

44.61 866.667

44.62 1000

44.63 1291.67

44.64 1091.67

44.65 1208.33

44.66 983.333

44.67 933.333

44.68 800

44.69 975

44.7 716.667

44.71 991.667

44.72 841.667

44.73 783.333

44.74 850

44.75 858.333

44.76 625

44.77 591.667

44.78 616.667

44.79 583.333

44.8 433.333

44.81 458.333

44.82 400

44.83 383.333

44.84 400

44.85 366.667

44.86 341.667

44.87 216.667

44.88 241.667

44.89 225

44.9 250

44.91 150

44.92 183.333

44.93 141.667

44.94 158.333

44.95 158.333

44.96 125

44.97 150

44.98 91.6667

44.99 141.667

45 83.3333

45.01 91.6667

45.02 108.333

45.03 83.3333

45.04 125

45.05 108.333

45.06 116.667

45.07 133.333

45.08 91.6667

45.09 58.3333

45.1 100

45.11 91.6667

45.12 116.667

45.13 75

45.14 66.6667

45.15 91.6667

45.16 75

45.17 100

45.18 108.333

45.19 83.3333

45.2 41.6667

45.21 58.3333

45.22 75

45.23 125

45.24 141.667

45.25 33.3333

45.26 100

45.27 50

45.28 83.3333

45.29 75

45.3 58.3333

45.31 41.6667

45.32 83.3333

45.33 83.3333

45.34 66.6667

45.35 75

45.36 50

45.37 66.6667

45.38 41.6667

45.39 25

45.4 66.6667

45.41 41.6667

45.42 91.6667

45.43 25

45.44 91.6667

45.45 33.3333

45.46 33.3333

45.47 41.6667

45.48 75

45.49 108.333

45.5 66.6667

45.51 58.3333

45.52 50

45.53 50

45.54 58.3333

45.55 58.3333

45.56 66.6667

45.57 8.33333

45.58 83.3333

45.59 83.3333

45.6 75

45.61 33.3333

45.62 25

45.63 41.6667

45.64 58.3333

45.65 16.6667

45.66 50

45.67 100

45.68 75

45.69 75

45.7 50

45.71 58.3333

45.72 50

45.73 58.3333

45.74 33.3333

45.75 33.3333

45.76 58.3333

45.77 50

45.78 41.6667

45.79 50

45.8 33.3333

45.81 66.6667

45.82 83.3333

45.83 58.3333

45.84 50

45.85 75

45.86 91.6667

45.87 25

45.88 41.6667

45.89 66.6667

45.9 83.3333

45.91 8.33333

45.92 75

45.93 58.3333

45.94 58.3333

45.95 41.6667

45.96 75

45.97 25

45.98 66.6667

45.99 75

46 58.3333

46.01 33.3333

46.02 33.3333

46.03 58.3333

46.04 66.6667

46.05 58.3333

46.06 41.6667

46.07 33.3333

46.08 58.3333

46.09 58.3333

46.1 50

46.11 41.6667

46.12 50

46.13 66.6667

46.14 50

46.15 66.6667

46.16 25

46.17 25

46.18 108.333

46.19 66.6667

46.2 33.3333

46.21 50

46.22 33.3333

46.23 50

46.24 25

46.25 50

46.26 41.6667

46.27 116.667

46.28 50

46.29 50

46.3 25

46.31 75

46.32 25

46.33 50

46.34 58.3333

46.35 58.3333

46.36 50

46.37 50

46.38 41.6667

46.39 58.3333

46.4 41.6667

46.41 58.3333

46.42 41.6667

46.43 75

46.44 41.6667

46.45 50

46.46 33.3333

46.47 66.6667

46.48 25

46.49 50

46.5 50

46.51 41.6667

46.52 50

46.53 50

46.54 41.6667

46.55 66.6667

46.56 25

46.57 58.3333

46.58 58.3333

46.59 41.6667

46.6 41.6667

46.61 58.3333

46.62 66.6667

46.63 25

46.64 58.3333

46.65 33.3333

46.66 100

46.67 75

46.68 75

46.69 83.3333

46.7 58.3333

46.71 33.3333

46.72 50

46.73 41.6667

46.74 25

46.75 58.3333

46.76 50

46.77 33.3333

46.78 50

46.79 58.3333

46.8 58.3333

46.81 75

46.82 41.6667

46.83 33.3333

46.84 75

46.85 75

46.86 25

46.87 50

46.88 100

46.89 33.3333

46.9 8.33333

46.91 33.3333

46.92 66.6667

46.93 41.6667

46.94 41.6667

46.95 50

46.96 91.6667

46.97 33.3333

46.98 41.6667

46.99 50

47 58.3333

47.01 33.3333

47.02 58.3333

47.03 33.3333

47.04 41.6667

47.05 58.3333

47.06 41.6667

47.07 33.3333

47.08 75

47.09 66.6667

47.1 66.6667

47.11 66.6667

47.12 25

47.13 33.3333

47.14 50

47.15 25

47.16 25

47.17 33.3333

47.18 41.6667

47.19 58.3333

47.2 33.3333

47.21 33.3333

47.22 116.667

47.23 25

47.24 100

47.25 75

47.26 66.6667

47.27 50

47.28 16.6667

47.29 66.6667

47.3 50

47.31 50

47.32 50

47.33 50

47.34 58.3333

47.35 8.33333

47.36 58.3333

47.37 50

47.38 58.3333

47.39 125

47.4 66.6667

47.41 66.6667

47.42 50

47.43 91.6667

47.44 66.6667

47.45 41.6667

47.46 75

47.47 58.3333

47.48 100

47.49 8.33333

47.5 50

47.51 41.6667

47.52 25

47.53 50

47.54 41.6667

47.55 58.3333

47.56 25

47.57 41.6667

47.58 75

47.59 33.3333

47.6 41.6667

47.61 25

47.62 66.6667

47.63 100

47.64 58.3333

47.65 33.3333

47.66 25

47.67 58.3333

47.68 58.3333

47.69 25

47.7 33.3333

47.71 75

47.72 75

47.73 50

47.74 66.6667

47.75 125

47.76 33.3333

47.77 66.6667

47.78 58.3333

47.79 58.3333

47.8 58.3333

47.81 83.3333

47.82 66.6667

47.83 41.6667

47.84 25

47.85 91.6667

47.86 66.6667

47.87 75

47.88 16.6667

47.89 50

47.9 0

47.91 66.6667

47.92 58.3333

47.93 75

47.94 66.6667

47.95 41.6667

47.96 16.6667

47.97 50

47.98 83.3333

47.99 41.6667

48 75

48.01 50

48.02 50

48.03 83.3333

48.04 33.3333

48.05 41.6667

48.06 83.3333

48.07 83.3333

48.08 33.3333

48.09 75

48.1 41.6667

48.11 33.3333

48.12 75

48.13 33.3333

48.14 33.3333

48.15 50

48.16 33.3333

48.17 41.6667

48.18 41.6667

48.19 16.6667

48.2 66.6667

48.21 41.6667

48.22 75

48.23 41.6667

48.24 66.6667

48.25 41.6667

48.26 50

48.27 41.6667

48.28 50

48.29 25

48.3 58.3333

48.31 66.6667

48.32 50

48.33 75

48.34 58.3333

48.35 66.6667

48.36 75

48.37 91.6667

48.38 50

48.39 66.6667

48.4 58.3333

48.41 25

48.42 33.3333

48.43 41.6667

48.44 16.6667

48.45 83.3333

48.46 58.3333

48.47 41.6667

48.48 16.6667

48.49 33.3333

48.5 100

48.51 41.6667

48.52 33.3333

48.53 50

48.54 58.3333

48.55 50

48.56 33.3333

48.57 50

48.58 41.6667

48.59 66.6667

48.6 58.3333

48.61 58.3333

48.62 58.3333

48.63 25

48.64 75

48.65 16.6667

48.66 91.6667

48.67 33.3333

48.68 25

48.69 50

48.7 0

48.71 25

48.72 50

48.73 50

48.74 33.3333

48.75 41.6667

48.76 75

48.77 50

48.78 58.3333

48.79 33.3333

48.8 91.6667

48.81 91.6667

48.82 75

48.83 66.6667

48.84 75

48.85 66.6667

48.86 41.6667

48.87 66.6667

48.88 41.6667

48.89 91.6667

48.9 66.6667

48.91 33.3333

48.92 58.3333

48.93 33.3333

48.94 16.6667

48.95 33.3333

48.96 50

48.97 66.6667

48.98 83.3333

48.99 33.3333

49 25

49.01 41.6667

49.02 91.6667

49.03 16.6667

49.04 58.3333

49.05 66.6667

49.06 41.6667

49.07 66.6667

49.08 66.6667

49.09 41.6667

49.1 58.3333

49.11 58.3333

49.12 66.6667

49.13 41.6667

49.14 50

49.15 25

49.16 58.3333

49.17 75

49.18 50

49.19 41.6667

49.2 75

49.21 50

49.22 50

49.23 50

49.24 58.3333

49.25 91.6667

49.26 75

49.27 50

49.28 50

49.29 50

49.3 50

49.31 66.6667

49.32 83.3333

49.33 58.3333

49.34 50

49.35 50

49.36 66.6667

49.37 66.6667

49.38 50

49.39 66.6667

49.4 66.6667

49.41 75

49.42 41.6667

49.43 91.6667

49.44 25

49.45 83.3333

49.46 83.3333

49.47 25

49.48 41.6667

49.49 66.6667

49.5 41.6667

49.51 25

49.52 41.6667

49.53 25

49.54 91.6667

49.55 58.3333

49.56 50

49.57 41.6667

49.58 41.6667

49.59 58.3333

49.6 50

49.61 33.3333

49.62 66.6667

49.63 41.6667

49.64 66.6667

49.65 75

49.66 58.3333

49.67 50

49.68 41.6667

49.69 58.3333

49.7 83.3333

49.71 58.3333

49.72 100

49.73 75

49.74 66.6667

49.75 16.6667

49.76 41.6667

49.77 33.3333

49.78 33.3333

49.79 41.6667

49.8 66.6667

49.81 91.6667

49.82 41.6667

49.83 25

49.84 41.6667

49.85 100

49.86 58.3333

49.87 75

49.88 75

49.89 50

49.9 25

49.91 50

49.92 50

49.93 58.3333

49.94 33.3333

49.95 41.6667

49.96 58.3333

49.97 91.6667

49.98 16.6667

49.99 41.6667

50 83.3333

50.01 58.3333

50.02 16.6667

50.03 75

50.04 41.6667

50.05 41.6667

50.06 33.3333

50.07 58.3333

50.08 50

50.09 75

50.1 33.3333

50.11 25

50.12 50

50.13 16.6667

50.14 25

50.15 66.6667

50.16 66.6667

50.17 50

50.18 66.6667

50.19 25

50.2 25

50.21 41.6667

50.22 50

50.23 41.6667

50.24 75

50.25 50

50.26 66.6667

50.27 16.6667

50.28 41.6667

50.29 50

50.3 83.3333

50.31 41.6667

50.32 83.3333

50.33 66.6667

50.34 58.3333

50.35 41.6667

50.36 58.3333

50.37 41.6667

50.38 75

50.39 66.6667

50.4 66.6667

50.41 58.3333

50.42 41.6667

50.43 16.6667

50.44 66.6667

50.45 66.6667

50.46 83.3333

50.47 100

50.48 50

50.49 33.3333

50.5 25

50.51 33.3333

50.52 41.6667

50.53 58.3333

50.54 50

50.55 33.3333

50.56 16.6667

50.57 41.6667

50.58 33.3333

50.59 50

50.6 41.6667

50.61 33.3333

50.62 91.6667

50.63 41.6667

50.64 75

50.65 91.6667

50.66 25

50.67 50

50.68 66.6667

50.69 41.6667

50.7 41.6667

50.71 58.3333

50.72 58.3333

50.73 66.6667

50.74 25

50.75 58.3333

50.76 50

50.77 25

50.78 75

50.79 50

50.8 33.3333

50.81 41.6667

50.82 75

50.83 116.667

50.84 66.6667

50.85 58.3333

50.86 16.6667

50.87 41.6667

50.88 91.6667

50.89 66.6667

50.9 41.6667

50.91 16.6667

50.92 58.3333

50.93 33.3333

50.94 41.6667

50.95 50

50.96 58.3333

50.97 50

50.98 66.6667

50.99 8.33333

51 83.3333

51.01 8.33333

51.02 33.3333

51.03 41.6667

51.04 50

51.05 16.6667

51.06 75

51.07 33.3333

51.08 58.3333

51.09 75

51.1 50

51.11 41.6667

51.12 50

51.13 33.3333

51.14 75

51.15 41.6667

51.16 25

51.17 50

51.18 25

51.19 75

51.2 66.6667

51.21 33.3333

51.22 66.6667

51.23 66.6667

51.24 33.3333

51.25 41.6667

51.26 83.3333

51.27 16.6667

51.28 58.3333

51.29 41.6667

51.3 66.6667

51.31 66.6667

51.32 58.3333

51.33 58.3333

51.34 58.3333

51.35 25

51.36 25

51.37 33.3333

51.38 50

51.39 66.6667

51.4 33.3333

51.41 33.3333

51.42 41.6667

51.43 41.6667

51.44 33.3333

51.45 50

51.46 16.6667

51.47 91.6667

51.48 75

51.49 66.6667

51.5 66.6667

51.51 41.6667

51.52 25

51.53 33.3333

51.54 33.3333

51.55 33.3333

51.56 16.6667

51.57 33.3333

51.58 58.3333

51.59 75

51.6 41.6667

51.61 66.6667

51.62 108.333

51.63 75

51.64 41.6667

51.65 41.6667

51.66 58.3333

51.67 75

51.68 8.33333

51.69 33.3333

51.7 16.6667

51.71 58.3333

51.72 75

51.73 16.6667

51.74 58.3333

51.75 58.3333

51.76 16.6667

51.77 66.6667

51.78 66.6667

51.79 66.6667

51.8 25

51.81 66.6667

51.82 91.6667

51.83 91.6667

51.84 50

51.85 25

51.86 50

51.87 33.3333

51.88 83.3333

51.89 16.6667

51.9 50

51.91 33.3333

51.92 41.6667

51.93 33.3333

51.94 66.6667

51.95 25

51.96 75

51.97 83.3333

51.98 75

51.99 16.6667

52 66.6667

52.01 75

52.02 58.3333

52.03 66.6667

52.04 41.6667

52.05 50

52.06 58.3333

52.07 41.6667

52.08 66.6667

52.09 50

52.1 75

52.11 58.3333

52.12 25

52.13 58.3333

52.14 58.3333

52.15 25

52.16 58.3333

52.17 66.6667

52.18 58.3333

52.19 41.6667

52.2 50

52.21 8.33333

52.22 66.6667

52.23 16.6667

52.24 8.33333

52.25 25

52.26 41.6667

52.27 58.3333

52.28 58.3333

52.29 16.6667

52.3 58.3333

52.31 50

52.32 100

52.33 33.3333

52.34 25

52.35 33.3333

52.36 58.3333

52.37 41.6667

52.38 58.3333

52.39 33.3333

52.4 58.3333

52.41 50

52.42 50

52.43 83.3333

52.44 75

52.45 50

52.46 41.6667

52.47 33.3333

52.48 41.6667

52.49 133.333

52.5 66.6667

52.51 50

52.52 58.3333

52.53 58.3333

52.54 25

52.55 66.6667

52.56 50

52.57 75

52.58 66.6667

52.59 50

52.6 25

52.61 66.6667

52.62 66.6667

52.63 25

52.64 41.6667

52.65 50

52.66 66.6667

52.67 66.6667

52.68 41.6667

52.69 66.6667

52.7 8.33333

52.71 100

52.72 41.6667

52.73 25

52.74 58.3333

52.75 58.3333

52.76 58.3333

52.77 33.3333

52.78 41.6667

52.79 50

52.8 41.6667

52.81 50

52.82 58.3333

52.83 33.3333

52.84 41.6667

52.85 75

52.86 66.6667

52.87 66.6667

52.88 50

52.89 50

52.9 41.6667

52.91 33.3333

52.92 16.6667

52.93 75

52.94 58.3333

52.95 58.3333

52.96 58.3333

52.97 41.6667

52.98 41.6667

52.99 41.6667

53 83.3333

53.01 16.6667

53.02 108.333

53.03 41.6667

53.04 25

53.05 41.6667

53.06 58.3333

53.07 58.3333

53.08 25

53.09 83.3333

53.1 41.6667

53.11 50

53.12 41.6667

53.13 66.6667

53.14 75

53.15 50

53.16 41.6667

53.17 50

53.18 75

53.19 58.3333

53.2 33.3333

53.21 58.3333

53.22 66.6667

53.23 41.6667

53.24 41.6667

53.25 58.3333

53.26 33.3333

53.27 108.333

53.28 58.3333

53.29 66.6667

53.3 91.6667

53.31 41.6667

53.32 50

53.33 25

53.34 50

53.35 0

53.36 50

53.37 66.6667

53.38 41.6667

53.39 58.3333

53.4 66.6667

53.41 33.3333

53.42 41.6667

53.43 50

53.44 83.3333

53.45 16.6667

53.46 50

53.47 66.6667

53.48 25

53.49 83.3333

53.5 33.3333

53.51 83.3333

53.52 50

53.53 50

53.54 50

53.55 58.3333

53.56 50

53.57 58.3333

53.58 66.6667

53.59 33.3333

53.6 41.6667

53.61 58.3333

53.62 75

53.63 50

53.64 33.3333

53.65 50

53.66 33.3333

53.67 66.6667

53.68 50

53.69 41.6667

53.7 41.6667

53.71 66.6667

53.72 25

53.73 83.3333

53.74 33.3333

53.75 41.6667

53.76 50

53.77 58.3333

53.78 41.6667

53.79 41.6667

53.8 58.3333

53.81 66.6667

53.82 83.3333

53.83 83.3333

53.84 66.6667

53.85 91.6667

53.86 33.3333

53.87 33.3333

53.88 66.6667

53.89 33.3333

53.9 75

53.91 50

53.92 41.6667

53.93 25

53.94 41.6667

53.95 75

53.96 41.6667

53.97 41.6667

53.98 91.6667

53.99 83.3333

54 16.6667

54.01 58.3333

54.02 50

54.03 75

54.04 50

54.05 41.6667

54.06 50

54.07 58.3333

54.08 50

54.09 41.6667

54.1 75

54.11 50

54.12 41.6667

54.13 33.3333

54.14 75

54.15 100

54.16 58.3333

54.17 58.3333

54.18 50

54.19 66.6667

54.2 66.6667

54.21 58.3333

54.22 83.3333

54.23 66.6667

54.24 58.3333

54.25 83.3333

54.26 75

54.27 91.6667

54.28 41.6667

54.29 25

54.3 16.6667

54.31 16.6667

54.32 50

54.33 41.6667

54.34 58.3333

54.35 75

54.36 50

54.37 25

54.38 58.3333

54.39 75

54.4 50

54.41 25

54.42 33.3333

54.43 58.3333

54.44 50

54.45 83.3333

54.46 75

54.47 58.3333

54.48 50

54.49 33.3333

54.5 41.6667

54.51 100

54.52 75

54.53 16.6667

54.54 75

54.55 75

54.56 66.6667

54.57 66.6667

54.58 91.6667

54.59 83.3333

54.6 83.3333

54.61 50

54.62 83.3333

54.63 8.33333

54.64 58.3333

54.65 50

54.66 33.3333

54.67 25

54.68 75

54.69 50

54.7 91.6667

54.71 58.3333

54.72 75

54.73 25

54.74 83.3333

54.75 33.3333

54.76 41.6667

54.77 66.6667

54.78 83.3333

54.79 50

54.8 50

54.81 66.6667

54.82 66.6667

54.83 25

54.84 41.6667

54.85 41.6667

54.86 50

54.87 75

54.88 66.6667

54.89 58.3333

54.9 58.3333

54.91 41.6667

54.92 16.6667

54.93 58.3333

54.94 33.3333

54.95 58.3333

54.96 33.3333

54.97 41.6667

54.98 33.3333

54.99 83.3333

55 58.3333

55.01 25

55.02 33.3333

55.03 50

55.04 16.6667

55.05 66.6667

55.06 83.3333

55.07 50

55.08 83.3333

55.09 50

55.1 58.3333

55.11 33.3333

55.12 50

55.13 41.6667

55.14 33.3333

55.15 50

55.16 25

55.17 91.6667

55.18 91.6667

55.19 58.3333

55.2 83.3333

55.21 25

55.22 91.6667

55.23 50

55.24 58.3333

55.25 58.3333

55.26 16.6667

55.27 58.3333

55.28 75

55.29 25

55.3 75

55.31 83.3333

55.32 83.3333

55.33 91.6667

55.34 50

55.35 16.6667

55.36 66.6667

55.37 33.3333

55.38 83.3333

55.39 58.3333

55.4 50

55.41 58.3333

55.42 50

55.43 50

55.44 108.333

55.45 50

55.46 25

55.47 83.3333

55.48 100

55.49 33.3333

55.5 83.3333

55.51 25

55.52 41.6667

55.53 16.6667

55.54 33.3333

55.55 58.3333

55.56 25

55.57 33.3333

55.58 58.3333

55.59 91.6667

55.6 66.6667

55.61 50

55.62 50

55.63 91.6667

55.64 25

55.65 50

55.66 100

55.67 58.3333

55.68 33.3333

55.69 25

55.7 58.3333

55.71 41.6667

55.72 33.3333

55.73 50

55.74 66.6667

55.75 75

55.76 58.3333

55.77 50

55.78 58.3333

55.79 75

55.8 66.6667

55.81 83.3333

55.82 66.6667

55.83 58.3333

55.84 100

55.85 58.3333

55.86 33.3333

55.87 58.3333

55.88 58.3333

55.89 91.6667

55.9 75

55.91 50

55.92 75

55.93 83.3333

55.94 75

55.95 66.6667

55.96 33.3333

55.97 66.6667

55.98 58.3333

55.99 75

56 50

56.01 58.3333

56.02 25

56.03 58.3333

56.04 83.3333

56.05 41.6667

56.06 50

56.07 50

56.08 33.3333

56.09 50

56.1 41.6667

56.11 50

56.12 91.6667

56.13 75

56.14 100

56.15 41.6667

56.16 58.3333

56.17 75

56.18 58.3333

56.19 75

56.2 75

56.21 50

56.22 41.6667

56.23 33.3333

56.24 33.3333

56.25 58.3333

56.26 50

56.27 58.3333

56.28 91.6667

56.29 33.3333

56.3 83.3333

56.31 58.3333

56.32 100

56.33 91.6667

56.34 66.6667

56.35 50

56.36 75

56.37 100

56.38 100

56.39 58.3333

56.4 25

56.41 100

56.42 66.6667

56.43 50

56.44 58.3333

56.45 50

56.46 75

56.47 125

56.48 83.3333

56.49 50

56.5 91.6667

56.51 75

56.52 66.6667

56.53 33.3333

56.54 66.6667

56.55 50

56.56 100

56.57 66.6667

56.58 75

56.59 41.6667

56.6 58.3333

56.61 33.3333

56.62 83.3333

56.63 75

56.64 41.6667

56.65 58.3333

56.66 50

56.67 66.6667

56.68 91.6667

56.69 83.3333

56.7 58.3333

56.71 91.6667

56.72 75

56.73 75

56.74 50

56.75 50

56.76 41.6667

56.77 58.3333

56.78 50

56.79 16.6667

56.8 83.3333

56.81 66.6667

56.82 75

56.83 58.3333

56.84 58.3333

56.85 83.3333

56.86 33.3333

56.87 83.3333

56.88 75

56.89 75

56.9 75

56.91 58.3333

56.92 58.3333

56.93 58.3333

56.94 66.6667

56.95 91.6667

56.96 75

56.97 58.3333

56.98 66.6667

56.99 100

57 66.6667

57.01 58.3333

57.02 58.3333

57.03 41.6667

57.04 125

57.05 75

57.06 41.6667

57.07 66.6667

57.08 66.6667

57.09 108.333

57.1 116.667

57.11 25

57.12 66.6667

57.13 133.333

57.14 83.3333

57.15 75

57.16 108.333

57.17 66.6667

57.18 75

57.19 100

57.2 41.6667

57.21 100

57.22 108.333

57.23 66.6667

57.24 83.3333

57.25 75

57.26 66.6667

57.27 33.3333

57.28 108.333

57.29 58.3333

57.3 66.6667

57.31 66.6667

57.32 83.3333

57.33 75

57.34 58.3333

57.35 83.3333

57.36 58.3333

57.37 66.6667

57.38 25

57.39 108.333

57.4 58.3333

57.41 66.6667

57.42 66.6667

57.43 66.6667

57.44 50

57.45 58.3333

57.46 66.6667

57.47 100

57.48 100

57.49 58.3333

57.5 66.6667

57.51 33.3333

57.52 83.3333

57.53 83.3333

57.54 50

57.55 83.3333

57.56 66.6667

57.57 108.333

57.58 100

57.59 83.3333

57.6 58.3333

57.61 108.333

57.62 75

57.63 83.3333

57.64 75

57.65 50

57.66 58.3333

57.67 41.6667

57.68 58.3333

57.69 58.3333

57.7 66.6667

57.71 50

57.72 91.6667

57.73 100

57.74 91.6667

57.75 66.6667

57.76 91.6667

57.77 33.3333

57.78 50

57.79 33.3333

57.8 75

57.81 83.3333

57.82 50

57.83 33.3333

57.84 25

57.85 41.6667

57.86 50

57.87 50

57.88 41.6667

57.89 50

57.9 75

57.91 41.6667

57.92 83.3333

57.93 33.3333

57.94 58.3333

57.95 83.3333

57.96 66.6667

57.97 66.6667

57.98 50

57.99 41.6667

58 75

58.01 75

58.02 33.3333

58.03 58.3333

58.04 33.3333

58.05 58.3333

58.06 58.3333

58.07 41.6667

58.08 33.3333

58.09 58.3333

58.1 125

58.11 41.6667

58.12 91.6667

58.13 50

58.14 33.3333

58.15 75

58.16 33.3333

58.17 50

58.18 75

58.19 66.6667

58.2 91.6667

58.21 91.6667

58.22 41.6667

58.23 50

58.24 58.3333

58.25 75

58.26 33.3333

58.27 41.6667

58.28 108.333

58.29 41.6667

58.3 25

58.31 75

58.32 33.3333

58.33 66.6667

58.34 50

58.35 41.6667

58.36 58.3333

58.37 75

58.38 41.6667

58.39 50

58.4 25

58.41 16.6667

58.42 91.6667

58.43 25

58.44 100

58.45 83.3333

58.46 50

58.47 58.3333

58.48 41.6667

58.49 41.6667

58.5 66.6667

58.51 33.3333

58.52 75

58.53 75

58.54 33.3333

58.55 50

58.56 33.3333

58.57 50

58.58 58.3333

58.59 83.3333

58.6 33.3333

58.61 16.6667

58.62 66.6667

58.63 108.333

58.64 50

58.65 66.6667

58.66 83.3333

58.67 58.3333

58.68 33.3333

58.69 33.3333

58.7 50

58.71 66.6667

58.72 91.6667

58.73 50

58.74 25

58.75 75

58.76 58.3333

58.77 66.6667

58.78 50

58.79 100

58.8 50

58.81 33.3333

58.82 41.6667

58.83 41.6667

58.84 75

58.85 41.6667

58.86 108.333

58.87 41.6667

58.88 58.3333

58.89 83.3333

58.9 50

58.91 58.3333

58.92 91.6667

58.93 50

58.94 75

58.95 50

58.96 66.6667

58.97 83.3333

58.98 33.3333

58.99 50

59 91.6667

59.01 33.3333

59.02 50

59.03 41.6667

59.04 16.6667

59.05 100

59.06 66.6667

59.07 100

59.08 83.3333

59.09 50

59.1 25

59.11 83.3333

59.12 16.6667

59.13 66.6667

59.14 33.3333

59.15 41.6667

59.16 91.6667

59.17 75

59.18 33.3333

59.19 41.6667

59.2 50

59.21 91.6667

59.22 58.3333

59.23 58.3333

59.24 50

59.25 25

59.26 16.6667

59.27 66.6667

59.28 41.6667

59.29 25

59.3 58.3333

59.31 33.3333

59.32 75

59.33 141.667

59.34 58.3333

59.35 75

59.36 50

59.37 50

59.38 50

59.39 50

59.4 50

59.41 91.6667

59.42 50

59.43 58.3333

59.44 25

59.45 66.6667

59.46 58.3333

59.47 58.3333

59.48 66.6667

59.49 50

59.5 41.6667

59.51 50

59.52 100

59.53 33.3333

59.54 91.6667

59.55 33.3333

59.56 33.3333

59.57 58.3333

59.58 33.3333

59.59 41.6667

59.6 8.33333

59.61 75

59.62 75

59.63 50

59.64 50

59.65 66.6667

59.66 116.667

59.67 50

59.68 41.6667

59.69 75

59.7 25

59.71 83.3333

59.72 58.3333

59.73 33.3333

59.74 58.3333

59.75 41.6667

59.76 50

59.77 25

59.78 66.6667

59.79 41.6667

59.8 50

59.81 125

59.82 75

59.83 16.6667

59.84 75

59.85 58.3333

59.86 41.6667

59.87 50

59.88 50

59.89 75

59.9 50

59.91 41.6667

59.92 33.3333

59.93 50

59.94 66.6667

59.95 58.3333

59.96 50

59.97 50

59.98 100

59.99 25

60 58.3333

60.01 41.6667

60.02 41.6667

60.03 50

60.04 91.6667

60.05 50

60.06 91.6667

60.07 25

60.08 83.3333

60.09 50

60.1 58.3333

60.11 58.3333

60.12 33.3333

60.13 33.3333

60.14 25

60.15 41.6667

60.16 75

60.17 66.6667

60.18 66.6667

60.19 75

60.2 75

60.21 41.6667

60.22 91.6667

60.23 50

60.24 58.3333

60.25 25

60.26 41.6667

60.27 66.6667

60.28 108.333

60.29 75

60.3 58.3333

60.31 75

60.32 91.6667

60.33 100

60.34 66.6667

60.35 75

60.36 58.3333

60.37 66.6667

60.38 41.6667

60.39 50

60.4 33.3333

60.41 33.3333

60.42 41.6667

60.43 16.6667

60.44 58.3333

60.45 75

60.46 66.6667

60.47 58.3333

60.48 58.3333

60.49 33.3333

60.5 75

60.51 25

60.52 75

60.53 41.6667

60.54 8.33333

60.55 33.3333

60.56 58.3333

60.57 58.3333

60.58 66.6667

60.59 33.3333

60.6 83.3333

60.61 41.6667

60.62 50

60.63 41.6667

60.64 58.3333

60.65 66.6667

60.66 33.3333

60.67 83.3333

60.68 75

60.69 83.3333

60.7 25

60.71 58.3333

60.72 41.6667

60.73 66.6667

60.74 75

60.75 16.6667

60.76 75

60.77 91.6667

60.78 91.6667

60.79 33.3333

60.8 33.3333

60.81 33.3333

60.82 66.6667

60.83 25

60.84 66.6667

60.85 83.3333

60.86 33.3333

60.87 25

60.88 133.333

60.89 33.3333

60.9 58.3333

60.91 66.6667

60.92 75

60.93 33.3333

60.94 41.6667

60.95 41.6667

60.96 66.6667

60.97 66.6667

60.98 33.3333

60.99 58.3333

61 66.6667

61.01 83.3333

61.02 50

61.03 25

61.04 50

61.05 58.3333

61.06 50

61.07 41.6667

61.08 83.3333

61.09 58.3333

61.1 75

61.11 66.6667

61.12 33.3333

61.13 25

61.14 75

61.15 58.3333

61.16 41.6667

61.17 83.3333

61.18 50

61.19 50

61.2 33.3333

61.21 58.3333

61.22 66.6667

61.23 83.3333

61.24 83.3333

61.25 75

61.26 50

61.27 108.333

61.28 75

61.29 33.3333

61.3 25

61.31 83.3333

61.32 91.6667

61.33 100

61.34 91.6667

61.35 83.3333

61.36 50

61.37 66.6667

61.38 100

61.39 83.3333

61.4 66.6667

61.41 66.6667

61.42 83.3333

61.43 83.3333

61.44 41.6667

61.45 58.3333

61.46 75

61.47 50

61.48 8.33333

61.49 91.6667

61.5 83.3333

61.51 75

61.52 66.6667

61.53 58.3333

61.54 91.6667

61.55 83.3333

61.56 41.6667

61.57 66.6667

61.58 91.6667

61.59 75

61.6 108.333

61.61 83.3333

61.62 91.6667

61.63 91.6667

61.64 66.6667

61.65 58.3333

61.66 91.6667

61.67 75

61.68 41.6667

61.69 16.6667

61.7 58.3333

61.71 100

61.72 33.3333

61.73 50

61.74 50

61.75 75

61.76 58.3333

61.77 83.3333

61.78 100

61.79 66.6667

61.8 66.6667

61.81 141.667

61.82 83.3333

61.83 116.667

61.84 83.3333

61.85 83.3333

61.86 91.6667

61.87 91.6667

61.88 83.3333

61.89 41.6667

61.9 116.667

61.91 83.3333

61.92 91.6667

61.93 108.333

61.94 75

61.95 58.3333

61.96 83.3333

61.97 66.6667

61.98 91.6667

61.99 83.3333

62 66.6667

62.01 33.3333

62.02 125

62.03 66.6667

62.04 58.3333

62.05 75

62.06 108.333

62.07 75

62.08 83.3333

62.09 75

62.1 91.6667

62.11 66.6667

62.12 83.3333

62.13 58.3333

62.14 100

62.15 116.667

62.16 75

62.17 116.667

62.18 75

62.19 75

62.2 75

62.21 125

62.22 75

62.23 91.6667

62.24 116.667

62.25 91.6667

62.26 75

62.27 108.333

62.28 108.333

62.29 83.3333

62.3 83.3333

62.31 75

62.32 58.3333

62.33 83.3333

62.34 116.667

62.35 66.6667

62.36 116.667

62.37 150

62.38 33.3333

62.39 158.333

62.4 108.333

62.41 66.6667

62.42 58.3333

62.43 91.6667

62.44 108.333

62.45 108.333

62.46 50

62.47 125

62.48 108.333

62.49 91.6667

62.5 91.6667

62.51 108.333

62.52 66.6667

62.53 108.333

62.54 83.3333

62.55 83.3333

62.56 125

62.57 133.333

62.58 66.6667

62.59 108.333

62.6 108.333

62.61 75

62.62 66.6667

62.63 125

62.64 91.6667

62.65 133.333

62.66 83.3333

62.67 100

62.68 75

62.69 66.6667

62.7 108.333

62.71 108.333

62.72 100

62.73 100

62.74 91.6667

62.75 66.6667

62.76 66.6667

62.77 83.3333

62.78 58.3333

62.79 125

62.8 58.3333

62.81 108.333

62.82 75

62.83 183.333

62.84 125

62.85 83.3333

62.86 83.3333

62.87 83.3333

62.88 66.6667

62.89 133.333

62.9 83.3333

62.91 100

62.92 116.667

62.93 83.3333

62.94 125

62.95 100

62.96 100

62.97 83.3333

62.98 91.6667

62.99 58.3333

63 58.3333

63.01 75

63.02 41.6667

63.03 66.6667

63.04 158.333

63.05 50

63.06 50

63.07 125

63.08 91.6667

63.09 108.333

63.1 58.3333

63.11 108.333

63.12 116.667

63.13 66.6667

63.14 83.3333

63.15 108.333

63.16 108.333

63.17 58.3333

63.18 75

63.19 75

63.2 83.3333

63.21 108.333

63.22 75

63.23 141.667

63.24 58.3333

63.25 83.3333

63.26 50

63.27 83.3333

63.28 41.6667

63.29 58.3333

63.3 50

63.31 75

63.32 116.667

63.33 58.3333

63.34 116.667

63.35 83.3333

63.36 108.333

63.37 108.333

63.38 58.3333

63.39 108.333

63.4 83.3333

63.41 50

63.42 75

63.43 41.6667

63.44 91.6667

63.45 75

63.46 50

63.47 83.3333

63.48 91.6667

63.49 75

63.5 50

63.51 75

63.52 133.333

63.53 91.6667

63.54 58.3333

63.55 116.667

63.56 50

63.57 66.6667

63.58 75

63.59 66.6667

63.6 66.6667

63.61 83.3333

63.62 66.6667

63.63 75

63.64 41.6667

63.65 58.3333

63.66 83.3333

63.67 50

63.68 91.6667

63.69 58.3333

63.7 25

63.71 58.3333

63.72 83.3333

63.73 108.333

63.74 58.3333

63.75 83.3333

63.76 41.6667

63.77 41.6667

63.78 91.6667

63.79 50

63.8 41.6667

63.81 75

63.82 66.6667

63.83 66.6667

63.84 83.3333

63.85 58.3333

63.86 16.6667

63.87 50

63.88 66.6667

63.89 50

63.9 66.6667

63.91 83.3333

63.92 91.6667

63.93 116.667

63.94 75

63.95 100

63.96 58.3333

63.97 83.3333

63.98 83.3333

63.99 83.3333

64 100

64.01 91.6667

64.02 75

64.03 25

64.04 66.6667

64.05 91.6667

64.06 50

64.07 50

64.08 66.6667

64.09 91.6667

64.1 58.3333

64.11 50

64.12 83.3333

64.13 75

64.14 58.3333

64.15 83.3333

64.16 83.3333

64.17 91.6667

64.18 91.6667

64.19 33.3333

64.2 75

64.21 41.6667

64.22 50

64.23 50

64.24 75

64.25 66.6667

64.26 66.6667

64.27 58.3333

64.28 50

64.29 83.3333

64.3 75

64.31 75

64.32 66.6667

64.33 41.6667

64.34 58.3333

64.35 50

64.36 50

64.37 16.6667

64.38 83.3333

64.39 75

64.4 83.3333

64.41 58.3333

64.42 66.6667

64.43 100

64.44 58.3333

64.45 16.6667

64.46 33.3333

64.47 108.333

64.48 58.3333

64.49 108.333

64.5 75

64.51 83.3333

64.52 66.6667

64.53 83.3333

64.54 58.3333

64.55 108.333

64.56 58.3333

64.57 83.3333

64.58 83.3333

64.59 50

64.6 75

64.61 83.3333

64.62 66.6667

64.63 25

64.64 50

64.65 41.6667

64.66 58.3333

64.67 83.3333

64.68 58.3333

64.69 66.6667

64.7 50

64.71 41.6667

64.72 58.3333

64.73 66.6667

64.74 100

64.75 91.6667

64.76 133.333

64.77 66.6667

64.78 66.6667

64.79 75

64.8 116.667

64.81 108.333

64.82 58.3333

64.83 58.3333

64.84 58.3333

64.85 75

64.86 83.3333

64.87 83.3333

64.88 33.3333

64.89 100

64.9 133.333

64.91 91.6667

64.92 150

64.93 66.6667

64.94 83.3333

64.95 108.333

64.96 166.667

64.97 208.333

64.98 200

64.99 175

65 150

65.01 183.333

65.02 158.333

65.03 191.667

65.04 183.333

65.05 100

65.06 208.333

65.07 108.333

65.08 175

65.09 125

65.1 133.333

65.11 116.667

65.12 133.333

65.13 141.667

65.14 125

65.15 225

65.16 158.333

65.17 75

65.18 183.333

65.19 91.6667

65.2 183.333

65.21 108.333

65.22 116.667

65.23 108.333

65.24 125

65.25 100

65.26 108.333

65.27 75

65.28 91.6667

65.29 75

65.3 41.6667

65.31 66.6667

65.32 75

65.33 75

65.34 75

65.35 58.3333

65.36 33.3333

65.37 58.3333

65.38 100

65.39 41.6667

65.4 33.3333

65.41 133.333

65.42 50

65.43 50

65.44 75

65.45 33.3333

65.46 58.3333

65.47 50

65.48 33.3333

65.49 108.333

65.5 66.6667

65.51 91.6667

65.52 41.6667

65.53 75

65.54 0

65.55 83.3333

65.56 66.6667

65.57 33.3333

65.58 58.3333

65.59 75

65.6 66.6667

65.61 41.6667

65.62 50

65.63 58.3333

65.64 25

65.65 50

65.66 50

65.67 50

65.68 25

65.69 83.3333

65.7 16.6667

65.71 66.6667

65.72 16.6667

65.73 75

65.74 16.6667

65.75 16.6667

65.76 58.3333

65.77 33.3333

65.78 58.3333

65.79 41.6667

65.8 58.3333

65.81 58.3333

65.82 66.6667

65.83 41.6667

65.84 50

65.85 75

65.86 91.6667

65.87 58.3333

65.88 50

65.89 58.3333

65.9 75

65.91 16.6667

65.92 25

65.93 66.6667

65.94 75

65.95 58.3333

65.96 83.3333

65.97 33.3333

65.98 83.3333

65.99 58.3333

66 50

66.01 41.6667

66.02 41.6667

66.03 33.3333

66.04 66.6667

66.05 91.6667

66.06 58.3333

66.07 25

66.08 50

66.09 25

66.1 58.3333

66.11 58.3333

66.12 66.6667

66.13 108.333

66.14 83.3333

66.15 66.6667

66.16 50

66.17 91.6667

66.18 66.6667

66.19 58.3333

66.2 58.3333

66.21 33.3333

66.22 33.3333

66.23 41.6667

66.24 50

66.25 58.3333

66.26 83.3333

66.27 33.3333

66.28 50

66.29 33.3333

66.3 91.6667

66.31 83.3333

66.32 58.3333

66.33 50

66.34 66.6667

66.35 41.6667

66.36 41.6667

66.37 41.6667

66.38 41.6667

66.39 75

66.4 50

66.41 33.3333

66.42 33.3333

66.43 50

66.44 58.3333

66.45 58.3333

66.46 91.6667

66.47 58.3333

66.48 66.6667

66.49 50

66.5 75

66.51 41.6667

66.52 75

66.53 41.6667

66.54 66.6667

66.55 58.3333

66.56 50

66.57 41.6667

66.58 66.6667

66.59 25

66.6 16.6667

66.61 66.6667

66.62 58.3333

66.63 50

66.64 66.6667

66.65 58.3333

66.66 33.3333

66.67 25

66.68 58.3333

66.69 58.3333

66.7 50

66.71 41.6667

66.72 25

66.73 50

66.74 75

66.75 8.33333

66.76 75

66.77 75

66.78 50

66.79 41.6667

66.8 58.3333

66.81 25

66.82 83.3333

66.83 75

66.84 58.3333

66.85 58.3333

66.86 41.6667

66.87 66.6667

66.88 58.3333

66.89 41.6667

66.9 33.3333

66.91 16.6667

66.92 33.3333

66.93 66.6667

66.94 33.3333

66.95 25

66.96 25

66.97 66.6667

66.98 25

66.99 8.33333

67 50

67.01 58.3333

67.02 50

67.03 75

67.04 33.3333

67.05 66.6667

67.06 33.3333

67.07 58.3333

67.08 16.6667

67.09 33.3333

67.1 41.6667

67.11 50

67.12 58.3333

67.13 75

67.14 33.3333

67.15 33.3333

67.16 108.333

67.17 41.6667

67.18 50

67.19 41.6667

67.2 16.6667

67.21 8.33333

67.22 58.3333

67.23 50

67.24 50

67.25 50

67.26 33.3333

67.27 50

67.28 58.3333

67.29 41.6667

67.3 66.6667

67.31 50

67.32 83.3333

67.33 41.6667

67.34 41.6667

67.35 58.3333

67.36 83.3333

67.37 33.3333

67.38 33.3333

67.39 75

67.4 66.6667

67.41 33.3333

67.42 91.6667

67.43 41.6667

67.44 25

67.45 91.6667

67.46 66.6667

67.47 100

67.48 33.3333

67.49 50

67.5 33.3333

67.51 83.3333

67.52 33.3333

67.53 41.6667

67.54 33.3333

67.55 8.33333

67.56 58.3333

67.57 58.3333

67.58 8.33333

67.59 75

67.6 50

67.61 75

67.62 58.3333

67.63 58.3333

67.64 50

67.65 91.6667

67.66 75

67.67 58.3333

67.68 41.6667

67.69 58.3333

67.7 50

67.71 58.3333

67.72 66.6667

67.73 25

67.74 75

67.75 41.6667

67.76 50

67.77 25

67.78 33.3333

67.79 41.6667

67.8 25

67.81 33.3333

67.82 108.333

67.83 66.6667

67.84 50

67.85 83.3333

67.86 58.3333

67.87 83.3333

67.88 58.3333

67.89 66.6667

67.9 33.3333

67.91 50

67.92 41.6667

67.93 25

67.94 50

67.95 66.6667

67.96 50

67.97 58.3333

67.98 41.6667

67.99 75

68 58.3333

68.01 50

68.02 66.6667

68.03 41.6667

68.04 58.3333

68.05 58.3333

68.06 41.6667

68.07 91.6667

68.08 50

68.09 41.6667

68.1 50

68.11 41.6667

68.12 0

68.13 33.3333

68.14 58.3333

68.15 25

68.16 41.6667

68.17 50

68.18 50

68.19 33.3333

68.2 41.6667

68.21 75

68.22 50

68.23 75

68.24 83.3333

68.25 91.6667

68.26 33.3333

68.27 41.6667

68.28 58.3333

68.29 58.3333

68.3 33.3333

68.31 41.6667

68.32 41.6667

68.33 41.6667

68.34 50

68.35 83.3333

68.36 41.6667

68.37 66.6667

68.38 25

68.39 58.3333

68.4 41.6667

68.41 33.3333

68.42 41.6667

68.43 41.6667

68.44 66.6667

68.45 83.3333

68.46 58.3333

68.47 66.6667

68.48 58.3333

68.49 83.3333

68.5 8.33333

68.51 50

68.52 25

68.53 50

68.54 58.3333

68.55 33.3333

68.56 33.3333

68.57 41.6667

68.58 75

68.59 66.6667

68.6 41.6667

68.61 41.6667

68.62 75

68.63 33.3333

68.64 41.6667

68.65 66.6667

68.66 16.6667

68.67 75

68.68 58.3333

68.69 83.3333

68.7 41.6667

68.71 41.6667

68.72 66.6667

68.73 41.6667

68.74 25

68.75 33.3333

68.76 33.3333

68.77 66.6667

68.78 33.3333

68.79 75

68.8 41.6667

68.81 25

68.82 66.6667

68.83 33.3333

68.84 33.3333

68.85 41.6667

68.86 91.6667

68.87 33.3333

68.88 91.6667

68.89 91.6667

68.9 58.3333

68.91 41.6667

68.92 16.6667

68.93 83.3333

68.94 41.6667

68.95 75

68.96 108.333

68.97 50

68.98 41.6667

68.99 75

69 33.3333

69.01 91.6667

69.02 50

69.03 33.3333

69.04 50

69.05 41.6667

69.06 41.6667

69.07 41.6667

69.08 58.3333

69.09 58.3333

69.1 75

69.11 50

69.12 41.6667

69.13 75

69.14 58.3333

69.15 75

69.16 41.6667

69.17 33.3333

69.18 66.6667

69.19 41.6667

69.2 8.33333

69.21 41.6667

69.22 41.6667

69.23 33.3333

69.24 75

69.25 41.6667

69.26 75

69.27 41.6667

69.28 50

69.29 50

69.3 41.6667

69.31 83.3333

69.32 8.33333

69.33 41.6667

69.34 25

69.35 75

69.36 33.3333

69.37 50

69.38 50

69.39 25

69.4 50

69.41 33.3333

69.42 41.6667

69.43 66.6667

69.44 83.3333

69.45 33.3333

69.46 50

69.47 83.3333

69.48 75

69.49 16.6667

69.5 41.6667

69.51 50

69.52 58.3333

69.53 58.3333

69.54 33.3333

69.55 50

69.56 66.6667

69.57 33.3333

69.58 83.3333

69.59 41.6667

69.6 50

69.61 41.6667

69.62 50

69.63 75

69.64 66.6667

69.65 58.3333

69.66 91.6667

69.67 58.3333

69.68 66.6667

69.69 50

69.7 41.6667

69.71 16.6667

69.72 58.3333

69.73 25

69.74 25

69.75 41.6667

69.76 41.6667

69.77 25

69.78 58.3333

69.79 50

69.8 41.6667

69.81 25

69.82 16.6667

69.83 41.6667

69.84 25

69.85 41.6667

69.86 50

69.87 75

69.88 66.6667

69.89 41.6667

69.9 66.6667

69.91 41.6667

69.92 50

69.93 50

69.94 41.6667

69.95 33.3333

69.96 33.3333

69.97 66.6667

69.98 41.6667

69.99 16.6667

70 75

**Raw data 3**. XRD raw data of the powder obtained after heat-treatment of as-sprayed powders prepared from the solution with sucrose at 450 ℃.

Goniometer RINT2000 vertical goniometer

Attachment Auto sample changer type B(6 samples)

Monochromater Fixed Monochromator

ScanningMode 2Theta/Theta

ScanningType Continuos Scanning

X-Ray 40kV/100mA

DivSlit 1 deg.

DivH.L.Slit 10mm

SctSlit 1 deg.

RecSlit 0.15mm

Monochro RS No Use

K-beta filter

Start 20

Stop 70

Step 0.01

20 391.667

20.01 583.333

20.02 508.333

20.03 600

20.04 541.667

20.05 475

20.06 591.667

20.07 541.667

20.08 525

20.09 516.667

20.1 608.333

20.11 683.333

20.12 458.333

20.13 566.667

20.14 541.667

20.15 525

20.16 566.667

20.17 683.333

20.18 750

20.19 533.333

20.2 591.667

20.21 516.667

20.22 600

20.23 583.333

20.24 525

20.25 566.667

20.26 658.333

20.27 708.333

20.28 575

20.29 525

20.3 650

20.31 566.667

20.32 641.667

20.33 558.333

20.34 575

20.35 575

20.36 583.333

20.37 608.333

20.38 533.333

20.39 666.667

20.4 508.333

20.41 700

20.42 625

20.43 666.667

20.44 608.333

20.45 575

20.46 583.333

20.47 558.333

20.48 575

20.49 641.667

20.5 533.333

20.51 491.667

20.52 575

20.53 591.667

20.54 566.667

20.55 733.333

20.56 558.333

20.57 508.333

20.58 650

20.59 533.333

20.6 583.333

20.61 458.333

20.62 516.667

20.63 533.333

20.64 566.667

20.65 616.667

20.66 641.667

20.67 491.667

20.68 650

20.69 591.667

20.7 533.333

20.71 566.667

20.72 616.667

20.73 583.333

20.74 591.667

20.75 591.667

20.76 558.333

20.77 600

20.78 783.333

20.79 591.667

20.8 650

20.81 691.667

20.82 558.333

20.83 591.667

20.84 583.333

20.85 675

20.86 508.333

20.87 641.667

20.88 525

20.89 450

20.9 616.667

20.91 558.333

20.92 491.667

20.93 650

20.94 558.333

20.95 500

20.96 491.667

20.97 541.667

20.98 666.667

20.99 491.667

21 691.667

21.01 508.333

21.02 550

21.03 550

21.04 466.667

21.05 658.333

21.06 616.667

21.07 675

21.08 666.667

21.09 516.667

21.1 433.333

21.11 541.667

21.12 600

21.13 508.333

21.14 566.667

21.15 616.667

21.16 650

21.17 558.333

21.18 591.667

21.19 466.667

21.2 591.667

21.21 508.333

21.22 625

21.23 600

21.24 633.333

21.25 625

21.26 691.667

21.27 666.667

21.28 566.667

21.29 600

21.3 525

21.31 583.333

21.32 641.667

21.33 600

21.34 700

21.35 608.333

21.36 708.333

21.37 675

21.38 558.333

21.39 475

21.4 550

21.41 475

21.42 750

21.43 675

21.44 575

21.45 633.333

21.46 658.333

21.47 566.667

21.48 533.333

21.49 625

21.5 566.667

21.51 575

21.52 616.667

21.53 516.667

21.54 675

21.55 550

21.56 625

21.57 641.667

21.58 766.667

21.59 625

21.6 591.667

21.61 633.333

21.62 591.667

21.63 558.333

21.64 566.667

21.65 466.667

21.66 525

21.67 691.667

21.68 516.667

21.69 625

21.7 416.667

21.71 566.667

21.72 591.667

21.73 650

21.74 625

21.75 641.667

21.76 600

21.77 641.667

21.78 700

21.79 650

21.8 650

21.81 683.333

21.82 625

21.83 591.667

21.84 641.667

21.85 633.333

21.86 600

21.87 591.667

21.88 558.333

21.89 575

21.9 625

21.91 616.667

21.92 566.667

21.93 591.667

21.94 516.667

21.95 700

21.96 650

21.97 491.667

21.98 558.333

21.99 533.333

22 575

22.01 616.667

22.02 633.333

22.03 558.333

22.04 508.333

22.05 458.333

22.06 700

22.07 608.333

22.08 566.667

22.09 550

22.1 600

22.11 500

22.12 633.333

22.13 625

22.14 600

22.15 583.333

22.16 483.333

22.17 441.667

22.18 541.667

22.19 550

22.2 491.667

22.21 525

22.22 633.333

22.23 633.333

22.24 558.333

22.25 758.333

22.26 625

22.27 458.333

22.28 508.333

22.29 458.333

22.3 508.333

22.31 625

22.32 433.333

22.33 608.333

22.34 525

22.35 550

22.36 558.333

22.37 500

22.38 600

22.39 458.333

22.4 491.667

22.41 616.667

22.42 525

22.43 441.667

22.44 550

22.45 591.667

22.46 525

22.47 566.667

22.48 525

22.49 458.333

22.5 566.667

22.51 550

22.52 541.667

22.53 658.333

22.54 566.667

22.55 508.333

22.56 566.667

22.57 691.667

22.58 508.333

22.59 433.333

22.6 416.667

22.61 575

22.62 433.333

22.63 516.667

22.64 483.333

22.65 391.667

22.66 533.333

22.67 541.667

22.68 466.667

22.69 641.667

22.7 616.667

22.71 508.333

22.72 433.333

22.73 466.667

22.74 475

22.75 575

22.76 600

22.77 400

22.78 491.667

22.79 483.333

22.8 475

22.81 633.333

22.82 391.667

22.83 400

22.84 566.667

22.85 483.333

22.86 608.333

22.87 575

22.88 641.667

22.89 558.333

22.9 516.667

22.91 575

22.92 458.333

22.93 466.667

22.94 400

22.95 741.667

22.96 416.667

22.97 575

22.98 541.667

22.99 516.667

23 500

23.01 600

23.02 466.667

23.03 666.667

23.04 566.667

23.05 491.667

23.06 416.667

23.07 550

23.08 475

23.09 458.333

23.1 408.333

23.11 491.667

23.12 500

23.13 433.333

23.14 500

23.15 416.667

23.16 358.333

23.17 441.667

23.18 400

23.19 583.333

23.2 508.333

23.21 516.667

23.22 516.667

23.23 441.667

23.24 491.667

23.25 566.667

23.26 500

23.27 558.333

23.28 458.333

23.29 450

23.3 458.333

23.31 508.333

23.32 408.333

23.33 525

23.34 508.333

23.35 533.333

23.36 508.333

23.37 450

23.38 466.667

23.39 500

23.4 508.333

23.41 458.333

23.42 416.667

23.43 508.333

23.44 508.333

23.45 533.333

23.46 391.667

23.47 641.667

23.48 416.667

23.49 491.667

23.5 458.333

23.51 416.667

23.52 475

23.53 483.333

23.54 508.333

23.55 466.667

23.56 541.667

23.57 408.333

23.58 558.333

23.59 508.333

23.6 391.667

23.61 400

23.62 458.333

23.63 383.333

23.64 366.667

23.65 425

23.66 608.333

23.67 491.667

23.68 533.333

23.69 516.667

23.7 466.667

23.71 483.333

23.72 375

23.73 466.667

23.74 491.667

23.75 425

23.76 400

23.77 450

23.78 500

23.79 383.333

23.8 475

23.81 458.333

23.82 466.667

23.83 441.667

23.84 475

23.85 375

23.86 375

23.87 341.667

23.88 491.667

23.89 433.333

23.9 441.667

23.91 391.667

23.92 325

23.93 416.667

23.94 325

23.95 433.333

23.96 541.667

23.97 466.667

23.98 350

23.99 558.333

24 408.333

24.01 483.333

24.02 391.667

24.03 483.333

24.04 341.667

24.05 341.667

24.06 416.667

24.07 341.667

24.08 500

24.09 433.333

24.1 450

24.11 525

24.12 550

24.13 441.667

24.14 466.667

24.15 475

24.16 433.333

24.17 358.333

24.18 366.667

24.19 425

24.2 366.667

24.21 375

24.22 358.333

24.23 408.333

24.24 416.667

24.25 466.667

24.26 483.333

24.27 316.667

24.28 341.667

24.29 491.667

24.3 433.333

24.31 425

24.32 458.333

24.33 400

24.34 383.333

24.35 325

24.36 341.667

24.37 425

24.38 508.333

24.39 450

24.4 433.333

24.41 350

24.42 350

24.43 283.333

24.44 366.667

24.45 358.333

24.46 383.333

24.47 308.333

24.48 383.333

24.49 525

24.5 400

24.51 458.333

24.52 375

24.53 441.667

24.54 375

24.55 458.333

24.56 425

24.57 458.333

24.58 450

24.59 491.667

24.6 508.333

24.61 333.333

24.62 416.667

24.63 350

24.64 458.333

24.65 383.333

24.66 333.333

24.67 550

24.68 291.667

24.69 450

24.7 325

24.71 358.333

24.72 358.333

24.73 366.667

24.74 433.333

24.75 425

24.76 433.333

24.77 366.667

24.78 383.333

24.79 283.333

24.8 358.333

24.81 466.667

24.82 350

24.83 458.333

24.84 375

24.85 308.333

24.86 375

24.87 300

24.88 350

24.89 366.667

24.9 333.333

24.91 316.667

24.92 458.333

24.93 433.333

24.94 391.667

24.95 425

24.96 308.333

24.97 508.333

24.98 308.333

24.99 425

25 391.667

25.01 433.333

25.02 341.667

25.03 408.333

25.04 308.333

25.05 391.667

25.06 350

25.07 350

25.08 333.333

25.09 316.667

25.1 333.333

25.11 408.333

25.12 408.333

25.13 408.333

25.14 316.667

25.15 350

25.16 375

25.17 450

25.18 366.667

25.19 283.333

25.2 433.333

25.21 441.667

25.22 350

25.23 391.667

25.24 358.333

25.25 316.667

25.26 283.333

25.27 308.333

25.28 241.667

25.29 258.333

25.3 308.333

25.31 358.333

25.32 358.333

25.33 375

25.34 341.667

25.35 283.333

25.36 383.333

25.37 366.667

25.38 266.667

25.39 375

25.4 458.333

25.41 358.333

25.42 316.667

25.43 366.667

25.44 316.667

25.45 350

25.46 308.333

25.47 391.667

25.48 383.333

25.49 391.667

25.5 283.333

25.51 441.667

25.52 233.333

25.53 241.667

25.54 433.333

25.55 316.667

25.56 283.333

25.57 391.667

25.58 300

25.59 333.333

25.6 316.667

25.61 416.667

25.62 275

25.63 350

25.64 266.667

25.65 283.333

25.66 258.333

25.67 341.667

25.68 350

25.69 291.667

25.7 258.333

25.71 258.333

25.72 375

25.73 433.333

25.74 283.333

25.75 250

25.76 275

25.77 266.667

25.78 283.333

25.79 325

25.8 391.667

25.81 350

25.82 308.333

25.83 358.333

25.84 408.333

25.85 333.333

25.86 383.333

25.87 458.333

25.88 416.667

25.89 283.333

25.9 216.667

25.91 316.667

25.92 350

25.93 316.667

25.94 325

25.95 366.667

25.96 316.667

25.97 333.333

25.98 275

25.99 250

26 266.667

26.01 341.667

26.02 275

26.03 316.667

26.04 175

26.05 283.333

26.06 300

26.07 333.333

26.08 275

26.09 391.667

26.1 325

26.11 283.333

26.12 275

26.13 333.333

26.14 291.667

26.15 291.667

26.16 225

26.17 391.667

26.18 291.667

26.19 358.333

26.2 341.667

26.21 316.667

26.22 258.333

26.23 258.333

26.24 408.333

26.25 275

26.26 200

26.27 325

26.28 300

26.29 383.333

26.3 325

26.31 216.667

26.32 316.667

26.33 241.667

26.34 250

26.35 333.333

26.36 316.667

26.37 333.333

26.38 250

26.39 258.333

26.4 325

26.41 325

26.42 358.333

26.43 291.667

26.44 250

26.45 258.333

26.46 291.667

26.47 358.333

26.48 300

26.49 283.333

26.5 283.333

26.51 341.667

26.52 208.333

26.53 341.667

26.54 291.667

26.55 383.333

26.56 400

26.57 283.333

26.58 391.667

26.59 375

26.6 300

26.61 308.333

26.62 308.333

26.63 325

26.64 266.667

26.65 241.667

26.66 308.333

26.67 325

26.68 250

26.69 275

26.7 316.667

26.71 283.333

26.72 358.333

26.73 300

26.74 275

26.75 308.333

26.76 250

26.77 241.667

26.78 366.667

26.79 308.333

26.8 316.667

26.81 291.667

26.82 341.667

26.83 233.333

26.84 300

26.85 266.667

26.86 291.667

26.87 300

26.88 266.667

26.89 266.667

26.9 366.667

26.91 300

26.92 200

26.93 233.333

26.94 275

26.95 341.667

26.96 308.333

26.97 308.333

26.98 291.667

26.99 300

27 341.667

27.01 258.333

27.02 283.333

27.03 275

27.04 200

27.05 183.333

27.06 225

27.07 300

27.08 250

27.09 266.667

27.1 250

27.11 233.333

27.12 233.333

27.13 250

27.14 225

27.15 233.333

27.16 241.667

27.17 283.333

27.18 308.333

27.19 325

27.2 241.667

27.21 283.333

27.22 225

27.23 141.667

27.24 233.333

27.25 175

27.26 283.333

27.27 225

27.28 266.667

27.29 250

27.3 266.667

27.31 200

27.32 208.333

27.33 266.667

27.34 316.667

27.35 200

27.36 233.333

27.37 200

27.38 241.667

27.39 216.667

27.4 200

27.41 300

27.42 233.333

27.43 191.667

27.44 200

27.45 241.667

27.46 175

27.47 275

27.48 216.667

27.49 291.667

27.5 241.667

27.51 208.333

27.52 258.333

27.53 241.667

27.54 208.333

27.55 208.333

27.56 225

27.57 200

27.58 341.667

27.59 283.333

27.6 283.333

27.61 225

27.62 233.333

27.63 241.667

27.64 291.667

27.65 208.333

27.66 258.333

27.67 225

27.68 208.333

27.69 233.333

27.7 300

27.71 233.333

27.72 275

27.73 341.667

27.74 258.333

27.75 266.667

27.76 225

27.77 200

27.78 225

27.79 216.667

27.8 175

27.81 191.667

27.82 275

27.83 216.667

27.84 275

27.85 275

27.86 191.667

27.87 241.667

27.88 216.667

27.89 241.667

27.9 283.333

27.91 275

27.92 258.333

27.93 308.333

27.94 308.333

27.95 241.667

27.96 233.333

27.97 300

27.98 216.667

27.99 233.333

28 233.333

28.01 233.333

28.02 233.333

28.03 266.667

28.04 341.667

28.05 325

28.06 183.333

28.07 200

28.08 258.333

28.09 258.333

28.1 383.333

28.11 208.333

28.12 291.667

28.13 216.667

28.14 191.667

28.15 166.667

28.16 166.667

28.17 266.667

28.18 325

28.19 158.333

28.2 275

28.21 200

28.22 208.333

28.23 300

28.24 250

28.25 275

28.26 216.667

28.27 316.667

28.28 225

28.29 225

28.3 275

28.31 241.667

28.32 216.667

28.33 250

28.34 200

28.35 191.667

28.36 191.667

28.37 208.333

28.38 183.333

28.39 200

28.4 325

28.41 216.667

28.42 200

28.43 266.667

28.44 233.333

28.45 250

28.46 233.333

28.47 208.333

28.48 250

28.49 250

28.5 183.333

28.51 200

28.52 141.667

28.53 191.667

28.54 250

28.55 233.333

28.56 250

28.57 208.333

28.58 166.667

28.59 208.333

28.6 175

28.61 233.333

28.62 241.667

28.63 208.333

28.64 241.667

28.65 200

28.66 175

28.67 183.333

28.68 225

28.69 366.667

28.7 191.667

28.71 141.667

28.72 208.333

28.73 233.333

28.74 258.333

28.75 250

28.76 308.333

28.77 241.667

28.78 208.333

28.79 158.333

28.8 200

28.81 241.667

28.82 175

28.83 250

28.84 200

28.85 208.333

28.86 283.333

28.87 183.333

28.88 183.333

28.89 183.333

28.9 191.667

28.91 250

28.92 225

28.93 225

28.94 175

28.95 216.667

28.96 300

28.97 291.667

28.98 300

28.99 208.333

29 258.333

29.01 150

29.02 275

29.03 208.333

29.04 175

29.05 233.333

29.06 83.3333

29.07 200

29.08 183.333

29.09 166.667

29.1 300

29.11 258.333

29.12 200

29.13 250

29.14 175

29.15 275

29.16 200

29.17 291.667

29.18 316.667

29.19 183.333

29.2 241.667

29.21 216.667

29.22 266.667

29.23 166.667

29.24 233.333

29.25 191.667

29.26 366.667

29.27 166.667

29.28 250

29.29 275

29.3 208.333

29.31 283.333

29.32 275

29.33 191.667

29.34 183.333

29.35 208.333

29.36 183.333

29.37 241.667

29.38 191.667

29.39 175

29.4 191.667

29.41 191.667

29.42 141.667

29.43 183.333

29.44 250

29.45 191.667

29.46 225

29.47 191.667

29.48 250

29.49 175

29.5 216.667

29.51 266.667

29.52 183.333

29.53 166.667

29.54 150

29.55 208.333

29.56 175

29.57 233.333

29.58 200

29.59 250

29.6 275

29.61 208.333

29.62 250

29.63 233.333

29.64 175

29.65 208.333

29.66 175

29.67 191.667

29.68 225

29.69 250

29.7 191.667

29.71 258.333

29.72 225

29.73 250

29.74 250

29.75 191.667

29.76 175

29.77 241.667

29.78 225

29.79 208.333

29.8 216.667

29.81 191.667

29.82 225

29.83 208.333

29.84 200

29.85 283.333

29.86 175

29.87 150

29.88 191.667

29.89 183.333

29.9 183.333

29.91 208.333

29.92 241.667

29.93 275

29.94 166.667

29.95 158.333

29.96 133.333

29.97 125

29.98 183.333

29.99 241.667

30 175

30.01 200

30.02 225

30.03 316.667

30.04 233.333

30.05 183.333

30.06 241.667

30.07 116.667

30.08 200

30.09 191.667

30.1 216.667

30.11 208.333

30.12 225

30.13 125

30.14 200

30.15 150

30.16 208.333

30.17 200

30.18 175

30.19 150

30.2 158.333

30.21 258.333

30.22 241.667

30.23 200

30.24 183.333

30.25 183.333

30.26 191.667

30.27 158.333

30.28 200

30.29 283.333

30.3 191.667

30.31 216.667

30.32 208.333

30.33 216.667

30.34 233.333

30.35 233.333

30.36 266.667

30.37 175

30.38 141.667

30.39 250

30.4 208.333

30.41 225

30.42 191.667

30.43 233.333

30.44 191.667

30.45 225

30.46 183.333

30.47 216.667

30.48 233.333

30.49 241.667

30.5 175

30.51 208.333

30.52 158.333

30.53 283.333

30.54 241.667

30.55 183.333

30.56 166.667

30.57 208.333

30.58 183.333

30.59 241.667

30.6 166.667

30.61 150

30.62 208.333

30.63 200

30.64 183.333

30.65 275

30.66 183.333

30.67 175

30.68 175

30.69 150

30.7 250

30.71 183.333

30.72 175

30.73 175

30.74 233.333

30.75 233.333

30.76 241.667

30.77 166.667

30.78 216.667

30.79 225

30.8 200

30.81 216.667

30.82 166.667

30.83 141.667

30.84 183.333

30.85 183.333

30.86 183.333

30.87 191.667

30.88 200

30.89 216.667

30.9 241.667

30.91 166.667

30.92 191.667

30.93 250

30.94 166.667

30.95 183.333

30.96 158.333

30.97 250

30.98 166.667

30.99 158.333

31 250

31.01 150

31.02 175

31.03 175

31.04 175

31.05 233.333

31.06 175

31.07 208.333

31.08 191.667

31.09 225

31.1 183.333

31.11 191.667

31.12 158.333

31.13 200

31.14 125

31.15 150

31.16 175

31.17 150

31.18 125

31.19 208.333

31.2 100

31.21 116.667

31.22 250

31.23 250

31.24 191.667

31.25 166.667

31.26 158.333

31.27 116.667

31.28 175

31.29 183.333

31.3 200

31.31 166.667

31.32 100

31.33 183.333

31.34 216.667

31.35 325

31.36 150

31.37 183.333

31.38 166.667

31.39 250

31.4 208.333

31.41 225

31.42 241.667

31.43 208.333

31.44 208.333

31.45 266.667

31.46 175

31.47 250

31.48 183.333

31.49 208.333

31.5 183.333

31.51 141.667

31.52 108.333

31.53 250

31.54 175

31.55 175

31.56 191.667

31.57 191.667

31.58 241.667

31.59 158.333

31.6 166.667

31.61 233.333

31.62 233.333

31.63 158.333

31.64 150

31.65 150

31.66 183.333

31.67 166.667

31.68 191.667

31.69 208.333

31.7 216.667

31.71 150

31.72 141.667

31.73 225

31.74 141.667

31.75 150

31.76 200

31.77 233.333

31.78 233.333

31.79 183.333

31.8 225

31.81 166.667

31.82 241.667

31.83 191.667

31.84 175

31.85 100

31.86 225

31.87 133.333

31.88 166.667

31.89 166.667

31.9 166.667

31.91 150

31.92 225

31.93 150

31.94 133.333

31.95 250

31.96 183.333

31.97 233.333

31.98 241.667

31.99 175

32 158.333

32.01 233.333

32.02 141.667

32.03 133.333

32.04 175

32.05 200

32.06 208.333

32.07 208.333

32.08 208.333

32.09 225

32.1 183.333

32.11 233.333

32.12 108.333

32.13 158.333

32.14 166.667

32.15 200

32.16 191.667

32.17 250

32.18 191.667

32.19 308.333

32.2 133.333

32.21 166.667

32.22 175

32.23 150

32.24 175

32.25 158.333

32.26 191.667

32.27 250

32.28 141.667

32.29 216.667

32.3 158.333

32.31 191.667

32.32 166.667

32.33 208.333

32.34 108.333

32.35 150

32.36 216.667

32.37 158.333

32.38 141.667

32.39 175

32.4 150

32.41 208.333

32.42 158.333

32.43 133.333

32.44 116.667

32.45 133.333

32.46 158.333

32.47 200

32.48 183.333

32.49 150

32.5 233.333

32.51 216.667

32.52 233.333

32.53 200

32.54 150

32.55 200

32.56 200

32.57 125

32.58 191.667

32.59 133.333

32.6 133.333

32.61 200

32.62 166.667

32.63 208.333

32.64 200

32.65 200

32.66 158.333

32.67 175

32.68 158.333

32.69 191.667

32.7 150

32.71 200

32.72 158.333

32.73 208.333

32.74 200

32.75 191.667

32.76 191.667

32.77 141.667

32.78 141.667

32.79 125

32.8 216.667

32.81 250

32.82 175

32.83 241.667

32.84 216.667

32.85 216.667

32.86 225

32.87 158.333

32.88 150

32.89 150

32.9 175

32.91 175

32.92 133.333

32.93 166.667

32.94 216.667

32.95 166.667

32.96 125

32.97 183.333

32.98 175

32.99 191.667

33 141.667

33.01 125

33.02 150

33.03 225

33.04 216.667

33.05 125

33.06 200

33.07 175

33.08 158.333

33.09 200

33.1 141.667

33.11 241.667

33.12 116.667

33.13 150

33.14 216.667

33.15 183.333

33.16 141.667

33.17 208.333

33.18 241.667

33.19 216.667

33.2 166.667

33.21 225

33.22 158.333

33.23 166.667

33.24 141.667

33.25 166.667

33.26 133.333

33.27 133.333

33.28 183.333

33.29 141.667

33.3 200

33.31 216.667

33.32 191.667

33.33 166.667

33.34 166.667

33.35 183.333

33.36 133.333

33.37 175

33.38 125

33.39 208.333

33.4 175

33.41 191.667

33.42 116.667

33.43 200

33.44 225

33.45 133.333

33.46 191.667

33.47 158.333

33.48 233.333

33.49 191.667

33.5 158.333

33.51 183.333

33.52 283.333

33.53 158.333

33.54 183.333

33.55 166.667

33.56 175

33.57 158.333

33.58 233.333

33.59 133.333

33.6 175

33.61 233.333

33.62 175

33.63 133.333

33.64 141.667

33.65 200

33.66 183.333

33.67 183.333

33.68 125

33.69 191.667

33.7 125

33.71 225

33.72 108.333

33.73 175

33.74 225

33.75 216.667

33.76 191.667

33.77 191.667

33.78 225

33.79 200

33.8 175

33.81 158.333

33.82 200

33.83 183.333

33.84 183.333

33.85 200

33.86 91.6667

33.87 133.333

33.88 100

33.89 250

33.9 200

33.91 141.667

33.92 208.333

33.93 200

33.94 166.667

33.95 191.667

33.96 158.333

33.97 133.333

33.98 166.667

33.99 191.667

34 200

34.01 91.6667

34.02 141.667

34.03 166.667

34.04 133.333

34.05 183.333

34.06 200

34.07 166.667

34.08 200

34.09 141.667

34.1 200

34.11 308.333

34.12 241.667

34.13 150

34.14 225

34.15 191.667

34.16 125

34.17 150

34.18 200

34.19 183.333

34.2 233.333

34.21 133.333

34.22 158.333

34.23 175

34.24 183.333

34.25 158.333

34.26 233.333

34.27 150

34.28 166.667

34.29 150

34.3 183.333

34.31 208.333

34.32 200

34.33 141.667

34.34 166.667

34.35 150

34.36 150

34.37 166.667

34.38 133.333

34.39 200

34.4 225

34.41 191.667

34.42 158.333

34.43 158.333

34.44 108.333

34.45 275

34.46 225

34.47 200

34.48 116.667

34.49 200

34.5 191.667

34.51 150

34.52 175

34.53 183.333

34.54 283.333

34.55 133.333

34.56 158.333

34.57 191.667

34.58 141.667

34.59 200

34.6 208.333

34.61 116.667

34.62 175

34.63 150

34.64 133.333

34.65 183.333

34.66 125

34.67 125

34.68 125

34.69 125

34.7 150

34.71 191.667

34.72 158.333

34.73 200

34.74 208.333

34.75 166.667

34.76 150

34.77 191.667

34.78 150

34.79 191.667

34.8 208.333

34.81 150

34.82 150

34.83 183.333

34.84 116.667

34.85 141.667

34.86 225

34.87 133.333

34.88 200

34.89 166.667

34.9 175

34.91 150

34.92 175

34.93 250

34.94 116.667

34.95 125

34.96 125

34.97 250

34.98 183.333

34.99 150

35 108.333

35.01 183.333

35.02 133.333

35.03 200

35.04 150

35.05 175

35.06 183.333

35.07 116.667

35.08 150

35.09 158.333

35.1 158.333

35.11 133.333

35.12 200

35.13 175

35.14 200

35.15 125

35.16 225

35.17 133.333

35.18 133.333

35.19 225

35.2 200

35.21 200

35.22 150

35.23 158.333

35.24 166.667

35.25 108.333

35.26 216.667

35.27 150

35.28 233.333

35.29 141.667

35.3 133.333

35.31 175

35.32 116.667

35.33 200

35.34 250

35.35 141.667

35.36 116.667

35.37 233.333

35.38 150

35.39 150

35.4 125

35.41 191.667

35.42 91.6667

35.43 133.333

35.44 200

35.45 258.333

35.46 158.333

35.47 225

35.48 200

35.49 150

35.5 191.667

35.51 183.333

35.52 158.333

35.53 125

35.54 183.333

35.55 225

35.56 166.667

35.57 200

35.58 158.333

35.59 166.667

35.6 166.667

35.61 183.333

35.62 183.333

35.63 150

35.64 166.667

35.65 241.667

35.66 158.333

35.67 191.667

35.68 191.667

35.69 166.667

35.7 175

35.71 116.667

35.72 166.667

35.73 150

35.74 141.667

35.75 183.333

35.76 233.333

35.77 91.6667

35.78 200

35.79 108.333

35.8 158.333

35.81 158.333

35.82 158.333

35.83 158.333

35.84 116.667

35.85 158.333

35.86 150

35.87 133.333

35.88 166.667

35.89 133.333

35.9 191.667

35.91 183.333

35.92 208.333

35.93 175

35.94 150

35.95 150

35.96 91.6667

35.97 141.667

35.98 191.667

35.99 158.333

36 175

36.01 208.333

36.02 175

36.03 150

36.04 208.333

36.05 150

36.06 158.333

36.07 141.667

36.08 150

36.09 150

36.1 158.333

36.11 183.333

36.12 216.667

36.13 183.333

36.14 175

36.15 150

36.16 125

36.17 150

36.18 208.333

36.19 116.667

36.2 141.667

36.21 200

36.22 125

36.23 233.333

36.24 175

36.25 191.667

36.26 133.333

36.27 158.333

36.28 158.333

36.29 200

36.3 150

36.31 116.667

36.32 50

36.33 100

36.34 150

36.35 116.667

36.36 116.667

36.37 183.333

36.38 141.667

36.39 175

36.4 83.3333

36.41 191.667

36.42 175

36.43 166.667

36.44 125

36.45 141.667

36.46 183.333

36.47 166.667

36.48 91.6667

36.49 141.667

36.5 250

36.51 108.333

36.52 150

36.53 141.667

36.54 125

36.55 250

36.56 166.667

36.57 175

36.58 133.333

36.59 141.667

36.6 158.333

36.61 158.333

36.62 116.667

36.63 116.667

36.64 158.333

36.65 233.333

36.66 183.333

36.67 166.667

36.68 83.3333

36.69 175

36.7 175

36.71 125

36.72 191.667

36.73 133.333

36.74 141.667

36.75 191.667

36.76 158.333

36.77 166.667

36.78 175

36.79 175

36.8 125

36.81 108.333

36.82 183.333

36.83 116.667

36.84 175

36.85 150

36.86 166.667

36.87 125

36.88 191.667

36.89 141.667

36.9 125

36.91 125

36.92 175

36.93 150

36.94 150

36.95 158.333

36.96 175

36.97 158.333

36.98 191.667

36.99 116.667

37 116.667

37.01 183.333

37.02 83.3333

37.03 91.6667

37.04 125

37.05 141.667

37.06 166.667

37.07 133.333

37.08 166.667

37.09 175

37.1 125

37.11 125

37.12 166.667

37.13 133.333

37.14 166.667

37.15 150

37.16 141.667

37.17 166.667

37.18 108.333

37.19 108.333

37.2 108.333

37.21 125

37.22 125

37.23 116.667

37.24 133.333

37.25 108.333

37.26 100

37.27 158.333

37.28 141.667

37.29 100

37.3 116.667

37.31 116.667

37.32 125

37.33 116.667

37.34 216.667

37.35 241.667

37.36 108.333

37.37 125

37.38 66.6667

37.39 108.333

37.4 166.667

37.41 108.333

37.42 125

37.43 125

37.44 183.333

37.45 108.333

37.46 133.333

37.47 158.333

37.48 75

37.49 108.333

37.5 133.333

37.51 108.333

37.52 125

37.53 158.333

37.54 108.333

37.55 183.333

37.56 108.333

37.57 141.667

37.58 91.6667

37.59 75

37.6 108.333

37.61 225

37.62 108.333

37.63 125

37.64 108.333

37.65 125

37.66 91.6667

37.67 58.3333

37.68 133.333

37.69 58.3333

37.7 116.667

37.71 200

37.72 133.333

37.73 91.6667

37.74 116.667

37.75 208.333

37.76 125

37.77 125

37.78 125

37.79 141.667

37.8 125

37.81 83.3333

37.82 158.333

37.83 141.667

37.84 75

37.85 150

37.86 116.667

37.87 150

37.88 116.667

37.89 141.667

37.9 133.333

37.91 208.333

37.92 133.333

37.93 175

37.94 116.667

37.95 133.333

37.96 150

37.97 100

37.98 133.333

37.99 108.333

38 183.333

38.01 116.667

38.02 133.333

38.03 125

38.04 83.3333

38.05 158.333

38.06 166.667

38.07 58.3333

38.08 91.6667

38.09 175

38.1 91.6667

38.11 158.333

38.12 100

38.13 158.333

38.14 133.333

38.15 116.667

38.16 116.667

38.17 83.3333

38.18 100

38.19 100

38.2 83.3333

38.21 108.333

38.22 141.667

38.23 116.667

38.24 108.333

38.25 158.333

38.26 133.333

38.27 91.6667

38.28 141.667

38.29 100

38.3 108.333

38.31 75

38.32 116.667

38.33 66.6667

38.34 166.667

38.35 100

38.36 100

38.37 66.6667

38.38 108.333

38.39 116.667

38.4 150

38.41 133.333

38.42 83.3333

38.43 58.3333

38.44 166.667

38.45 150

38.46 141.667

38.47 116.667

38.48 150

38.49 75

38.5 133.333

38.51 175

38.52 58.3333

38.53 100

38.54 108.333

38.55 58.3333

38.56 150

38.57 75

38.58 100

38.59 150

38.6 125

38.61 100

38.62 108.333

38.63 83.3333

38.64 108.333

38.65 83.3333

38.66 108.333

38.67 150

38.68 150

38.69 116.667

38.7 158.333

38.71 175

38.72 83.3333

38.73 125

38.74 133.333

38.75 100

38.76 108.333

38.77 125

38.78 133.333

38.79 183.333

38.8 91.6667

38.81 100

38.82 150

38.83 83.3333

38.84 125

38.85 183.333

38.86 133.333

38.87 158.333

38.88 75

38.89 150

38.9 83.3333

38.91 150

38.92 125

38.93 75

38.94 83.3333

38.95 83.3333

38.96 133.333

38.97 125

38.98 83.3333

38.99 125

39 91.6667

39.01 100

39.02 150

39.03 141.667

39.04 66.6667

39.05 133.333

39.06 125

39.07 100

39.08 100

39.09 158.333

39.1 91.6667

39.11 150

39.12 116.667

39.13 100

39.14 83.3333

39.15 141.667

39.16 133.333

39.17 75

39.18 91.6667

39.19 141.667

39.2 166.667

39.21 125

39.22 91.6667

39.23 66.6667

39.24 125

39.25 108.333

39.26 91.6667

39.27 133.333

39.28 133.333

39.29 108.333

39.3 100

39.31 125

39.32 100

39.33 141.667

39.34 108.333

39.35 125

39.36 66.6667

39.37 116.667

39.38 83.3333

39.39 141.667

39.4 125

39.41 91.6667

39.42 75

39.43 125

39.44 108.333

39.45 50

39.46 116.667

39.47 83.3333

39.48 108.333

39.49 91.6667

39.5 116.667

39.51 50

39.52 108.333

39.53 33.3333

39.54 133.333

39.55 125

39.56 83.3333

39.57 125

39.58 66.6667

39.59 75

39.6 100

39.61 125

39.62 33.3333

39.63 91.6667

39.64 108.333

39.65 125

39.66 166.667

39.67 66.6667

39.68 125

39.69 91.6667

39.7 133.333

39.71 75

39.72 116.667

39.73 58.3333

39.74 108.333

39.75 108.333

39.76 141.667

39.77 141.667

39.78 50

39.79 108.333

39.8 141.667

39.81 125

39.82 133.333

39.83 108.333

39.84 116.667

39.85 116.667

39.86 116.667

39.87 116.667

39.88 108.333

39.89 133.333

39.9 83.3333

39.91 66.6667

39.92 108.333

39.93 108.333

39.94 75

39.95 58.3333

39.96 141.667

39.97 166.667

39.98 116.667

39.99 100

40 141.667

40.01 108.333

40.02 75

40.03 125

40.04 66.6667

40.05 108.333

40.06 75

40.07 58.3333

40.08 75

40.09 75

40.1 125

40.11 91.6667

40.12 125

40.13 83.3333

40.14 91.6667

40.15 133.333

40.16 108.333

40.17 133.333

40.18 66.6667

40.19 108.333

40.2 83.3333

40.21 108.333

40.22 91.6667

40.23 50

40.24 125

40.25 116.667

40.26 108.333

40.27 50

40.28 125

40.29 166.667

40.3 66.6667

40.31 116.667

40.32 91.6667

40.33 75

40.34 116.667

40.35 183.333

40.36 141.667

40.37 125

40.38 83.3333

40.39 58.3333

40.4 100

40.41 133.333

40.42 141.667

40.43 150

40.44 108.333

40.45 58.3333

40.46 75

40.47 83.3333

40.48 116.667

40.49 175

40.5 100

40.51 141.667

40.52 91.6667

40.53 125

40.54 133.333

40.55 91.6667

40.56 108.333

40.57 125

40.58 108.333

40.59 58.3333

40.6 125

40.61 141.667

40.62 133.333

40.63 83.3333

40.64 150

40.65 183.333

40.66 158.333

40.67 58.3333

40.68 75

40.69 141.667

40.7 125

40.71 83.3333

40.72 91.6667

40.73 75

40.74 150

40.75 83.3333

40.76 133.333

40.77 83.3333

40.78 66.6667

40.79 100

40.8 108.333

40.81 150

40.82 75

40.83 75

40.84 108.333

40.85 75

40.86 91.6667

40.87 75

40.88 75

40.89 116.667

40.9 100

40.91 83.3333

40.92 125

40.93 141.667

40.94 125

40.95 141.667

40.96 83.3333

40.97 75

40.98 91.6667

40.99 50

41 83.3333

41.01 141.667

41.02 66.6667

41.03 125

41.04 108.333

41.05 58.3333

41.06 83.3333

41.07 75

41.08 91.6667

41.09 66.6667

41.1 116.667

41.11 108.333

41.12 100

41.13 125

41.14 108.333

41.15 133.333

41.16 58.3333

41.17 91.6667

41.18 158.333

41.19 91.6667

41.2 158.333

41.21 108.333

41.22 133.333

41.23 125

41.24 75

41.25 50

41.26 91.6667

41.27 133.333

41.28 100

41.29 108.333

41.3 141.667

41.31 100

41.32 50

41.33 91.6667

41.34 75

41.35 108.333

41.36 91.6667

41.37 100

41.38 91.6667

41.39 100

41.4 108.333

41.41 91.6667

41.42 125

41.43 83.3333

41.44 100

41.45 116.667

41.46 133.333

41.47 83.3333

41.48 175

41.49 141.667

41.5 50

41.51 125

41.52 83.3333

41.53 83.3333

41.54 66.6667

41.55 41.6667

41.56 100

41.57 133.333

41.58 75

41.59 166.667

41.6 108.333

41.61 125

41.62 125

41.63 58.3333

41.64 75

41.65 108.333

41.66 141.667

41.67 75

41.68 50

41.69 58.3333

41.7 141.667

41.71 116.667

41.72 133.333

41.73 91.6667

41.74 83.3333

41.75 91.6667

41.76 133.333

41.77 191.667

41.78 75

41.79 83.3333

41.8 58.3333

41.81 91.6667

41.82 66.6667

41.83 150

41.84 91.6667

41.85 158.333

41.86 100

41.87 91.6667

41.88 116.667

41.89 100

41.9 75

41.91 91.6667

41.92 116.667

41.93 141.667

41.94 75

41.95 125

41.96 91.6667

41.97 125

41.98 91.6667

41.99 116.667

42 116.667

42.01 91.6667

42.02 108.333

42.03 166.667

42.04 75

42.05 116.667

42.06 66.6667

42.07 150

42.08 158.333

42.09 141.667

42.1 100

42.11 175

42.12 108.333

42.13 91.6667

42.14 75

42.15 83.3333

42.16 58.3333

42.17 91.6667

42.18 175

42.19 125

42.2 108.333

42.21 66.6667

42.22 125

42.23 133.333

42.24 58.3333

42.25 91.6667

42.26 100

42.27 100

42.28 158.333

42.29 125

42.3 175

42.31 116.667

42.32 116.667

42.33 116.667

42.34 83.3333

42.35 116.667

42.36 141.667

42.37 133.333

42.38 100

42.39 150

42.4 141.667

42.41 158.333

42.42 125

42.43 83.3333

42.44 83.3333

42.45 108.333

42.46 108.333

42.47 150

42.48 91.6667

42.49 166.667

42.5 100

42.51 166.667

42.52 133.333

42.53 50

42.54 100

42.55 83.3333

42.56 150

42.57 108.333

42.58 66.6667

42.59 66.6667

42.6 166.667

42.61 91.6667

42.62 91.6667

42.63 116.667

42.64 116.667

42.65 83.3333

42.66 158.333

42.67 158.333

42.68 108.333

42.69 83.3333

42.7 108.333

42.71 116.667

42.72 75

42.73 116.667

42.74 150

42.75 83.3333

42.76 108.333

42.77 100

42.78 158.333

42.79 150

42.8 75

42.81 125

42.82 66.6667

42.83 91.6667

42.84 150

42.85 125

42.86 50

42.87 100

42.88 125

42.89 100

42.9 158.333

42.91 125

42.92 150

42.93 66.6667

42.94 141.667

42.95 141.667

42.96 66.6667

42.97 158.333

42.98 66.6667

42.99 83.3333

43 41.6667

43.01 141.667

43.02 100

43.03 108.333

43.04 100

43.05 150

43.06 125

43.07 116.667

43.08 158.333

43.09 66.6667

43.1 83.3333

43.11 133.333

43.12 141.667

43.13 83.3333

43.14 141.667

43.15 41.6667

43.16 100

43.17 116.667

43.18 116.667

43.19 83.3333

43.2 141.667

43.21 116.667

43.22 175

43.23 91.6667

43.24 83.3333

43.25 141.667

43.26 100

43.27 133.333

43.28 116.667

43.29 116.667

43.3 116.667

43.31 91.6667

43.32 75

43.33 125

43.34 141.667

43.35 166.667

43.36 83.3333

43.37 116.667

43.38 66.6667

43.39 41.6667

43.4 125

43.41 158.333

43.42 66.6667

43.43 125

43.44 100

43.45 75

43.46 75

43.47 158.333

43.48 133.333

43.49 116.667

43.5 91.6667

43.51 75

43.52 75

43.53 125

43.54 175

43.55 133.333

43.56 100

43.57 116.667

43.58 108.333

43.59 116.667

43.6 141.667

43.61 108.333

43.62 125

43.63 166.667

43.64 91.6667

43.65 150

43.66 75

43.67 141.667

43.68 108.333

43.69 125

43.7 108.333

43.71 166.667

43.72 150

43.73 183.333

43.74 166.667

43.75 83.3333

43.76 141.667

43.77 100

43.78 158.333

43.79 125

43.8 116.667

43.81 100

43.82 116.667

43.83 58.3333

43.84 150

43.85 133.333

43.86 116.667

43.87 100

43.88 100

43.89 116.667

43.9 125

43.91 125

43.92 125

43.93 108.333

43.94 116.667

43.95 116.667

43.96 150

43.97 158.333

43.98 116.667

43.99 150

44 116.667

44.01 150

44.02 83.3333

44.03 133.333

44.04 83.3333

44.05 83.3333

44.06 116.667

44.07 75

44.08 83.3333

44.09 116.667

44.1 108.333

44.11 133.333

44.12 133.333

44.13 116.667

44.14 58.3333

44.15 83.3333

44.16 100

44.17 91.6667

44.18 133.333

44.19 150

44.2 141.667

44.21 83.3333

44.22 100

44.23 158.333

44.24 133.333

44.25 141.667

44.26 125

44.27 108.333

44.28 158.333

44.29 116.667

44.3 125

44.31 125

44.32 133.333

44.33 141.667

44.34 116.667

44.35 191.667

44.36 91.6667

44.37 166.667

44.38 200

44.39 91.6667

44.4 133.333

44.41 108.333

44.42 175

44.43 116.667

44.44 175

44.45 100

44.46 158.333

44.47 158.333

44.48 125

44.49 141.667

44.5 175

44.51 125

44.52 241.667

44.53 191.667

44.54 191.667

44.55 183.333

44.56 108.333

44.57 141.667

44.58 158.333

44.59 250

44.6 216.667

44.61 208.333

44.62 191.667

44.63 291.667

44.64 225

44.65 233.333

44.66 166.667

44.67 175

44.68 241.667

44.69 225

44.7 283.333

44.71 291.667

44.72 191.667

44.73 250

44.74 300

44.75 183.333

44.76 250

44.77 275

44.78 133.333

44.79 208.333

44.8 191.667

44.81 200

44.82 216.667

44.83 183.333

44.84 208.333

44.85 125

44.86 116.667

44.87 208.333

44.88 141.667

44.89 225

44.9 133.333

44.91 133.333

44.92 175

44.93 91.6667

44.94 100

44.95 191.667

44.96 150

44.97 83.3333

44.98 100

44.99 166.667

45 200

45.01 108.333

45.02 133.333

45.03 108.333

45.04 91.6667

45.05 141.667

45.06 133.333

45.07 116.667

45.08 150

45.09 108.333

45.1 83.3333

45.11 108.333

45.12 75

45.13 116.667

45.14 116.667

45.15 133.333

45.16 158.333

45.17 116.667

45.18 66.6667

45.19 41.6667

45.2 91.6667

45.21 91.6667

45.22 116.667

45.23 100

45.24 100

45.25 175

45.26 108.333

45.27 116.667

45.28 116.667

45.29 116.667

45.3 116.667

45.31 83.3333

45.32 91.6667

45.33 91.6667

45.34 125

45.35 83.3333

45.36 166.667

45.37 133.333

45.38 100

45.39 125

45.4 75

45.41 141.667

45.42 116.667

45.43 116.667

45.44 133.333

45.45 141.667

45.46 108.333

45.47 116.667

45.48 125

45.49 91.6667

45.5 83.3333

45.51 150

45.52 100

45.53 66.6667

45.54 133.333

45.55 75

45.56 58.3333

45.57 125

45.58 75

45.59 100

45.6 133.333

45.61 83.3333

45.62 91.6667

45.63 66.6667

45.64 41.6667

45.65 116.667

45.66 108.333

45.67 125

45.68 108.333

45.69 75

45.7 91.6667

45.71 133.333

45.72 91.6667

45.73 83.3333

45.74 100

45.75 75

45.76 83.3333

45.77 75

45.78 100

45.79 66.6667

45.8 75

45.81 83.3333

45.82 100

45.83 133.333

45.84 200

45.85 75

45.86 116.667

45.87 66.6667

45.88 158.333

45.89 83.3333

45.9 91.6667

45.91 91.6667

45.92 66.6667

45.93 75

45.94 133.333

45.95 100

45.96 116.667

45.97 108.333

45.98 100

45.99 141.667

46 91.6667

46.01 108.333

46.02 100

46.03 83.3333

46.04 91.6667

46.05 91.6667

46.06 125

46.07 116.667

46.08 125

46.09 50

46.1 83.3333

46.11 91.6667

46.12 91.6667

46.13 66.6667

46.14 100

46.15 66.6667

46.16 100

46.17 66.6667

46.18 66.6667

46.19 116.667

46.2 158.333

46.21 116.667

46.22 83.3333

46.23 141.667

46.24 41.6667

46.25 66.6667

46.26 58.3333

46.27 58.3333

46.28 83.3333

46.29 75

46.3 125

46.31 133.333

46.32 83.3333

46.33 75

46.34 125

46.35 91.6667

46.36 58.3333

46.37 108.333

46.38 125

46.39 116.667

46.4 133.333

46.41 66.6667

46.42 91.6667

46.43 83.3333

46.44 58.3333

46.45 116.667

46.46 141.667

46.47 125

46.48 75

46.49 108.333

46.5 108.333

46.51 150

46.52 83.3333

46.53 83.3333

46.54 141.667

46.55 91.6667

46.56 50

46.57 125

46.58 83.3333

46.59 100

46.6 66.6667

46.61 125

46.62 150

46.63 100

46.64 25

46.65 91.6667

46.66 166.667

46.67 100

46.68 141.667

46.69 108.333

46.7 75

46.71 83.3333

46.72 125

46.73 100

46.74 116.667

46.75 91.6667

46.76 50

46.77 133.333

46.78 83.3333

46.79 125

46.8 75

46.81 100

46.82 33.3333

46.83 83.3333

46.84 166.667

46.85 66.6667

46.86 141.667

46.87 66.6667

46.88 41.6667

46.89 83.3333

46.9 150

46.91 91.6667

46.92 158.333

46.93 75

46.94 141.667

46.95 83.3333

46.96 108.333

46.97 83.3333

46.98 91.6667

46.99 125

47 83.3333

47.01 133.333

47.02 58.3333

47.03 141.667

47.04 50

47.05 108.333

47.06 41.6667

47.07 91.6667

47.08 100

47.09 125

47.1 100

47.11 108.333

47.12 75

47.13 108.333

47.14 125

47.15 75

47.16 125

47.17 116.667

47.18 116.667

47.19 150

47.2 75

47.21 125

47.22 116.667

47.23 108.333

47.24 83.3333

47.25 175

47.26 83.3333

47.27 116.667

47.28 158.333

47.29 75

47.3 58.3333

47.31 116.667

47.32 133.333

47.33 116.667

47.34 91.6667

47.35 91.6667

47.36 108.333

47.37 133.333

47.38 83.3333

47.39 125

47.4 91.6667

47.41 116.667

47.42 58.3333

47.43 66.6667

47.44 58.3333

47.45 133.333

47.46 58.3333

47.47 83.3333

47.48 100

47.49 108.333

47.5 66.6667

47.51 66.6667

47.52 116.667

47.53 66.6667

47.54 91.6667

47.55 100

47.56 58.3333

47.57 66.6667

47.58 58.3333

47.59 125

47.6 75

47.61 58.3333

47.62 100

47.63 116.667

47.64 91.6667

47.65 41.6667

47.66 83.3333

47.67 150

47.68 116.667

47.69 108.333

47.7 83.3333

47.71 75

47.72 91.6667

47.73 141.667

47.74 75

47.75 125

47.76 108.333

47.77 75

47.78 91.6667

47.79 66.6667

47.8 125

47.81 158.333

47.82 116.667

47.83 125

47.84 100

47.85 83.3333

47.86 83.3333

47.87 100

47.88 116.667

47.89 100

47.9 91.6667

47.91 91.6667

47.92 100

47.93 125

47.94 91.6667

47.95 108.333

47.96 116.667

47.97 108.333

47.98 83.3333

47.99 83.3333

48 133.333

48.01 91.6667

48.02 41.6667

48.03 91.6667

48.04 50

48.05 108.333

48.06 75

48.07 83.3333

48.08 108.333

48.09 100

48.1 116.667

48.11 75

48.12 108.333

48.13 133.333

48.14 125

48.15 125

48.16 58.3333

48.17 41.6667

48.18 150

48.19 108.333

48.2 83.3333

48.21 158.333

48.22 91.6667

48.23 108.333

48.24 116.667

48.25 91.6667

48.26 125

48.27 41.6667

48.28 91.6667

48.29 91.6667

48.3 66.6667

48.31 66.6667

48.32 100

48.33 100

48.34 91.6667

48.35 75

48.36 83.3333

48.37 75

48.38 41.6667

48.39 141.667

48.4 133.333

48.41 66.6667

48.42 91.6667

48.43 108.333

48.44 133.333

48.45 133.333

48.46 108.333

48.47 58.3333

48.48 58.3333

48.49 100

48.5 83.3333

48.51 66.6667

48.52 133.333

48.53 133.333

48.54 125

48.55 66.6667

48.56 91.6667

48.57 125

48.58 83.3333

48.59 75

48.6 141.667

48.61 83.3333

48.62 83.3333

48.63 141.667

48.64 41.6667

48.65 83.3333

48.66 91.6667

48.67 116.667

48.68 91.6667

48.69 108.333

48.7 75

48.71 116.667

48.72 83.3333

48.73 33.3333

48.74 75

48.75 66.6667

48.76 125

48.77 75

48.78 83.3333

48.79 83.3333

48.8 125

48.81 91.6667

48.82 91.6667

48.83 75

48.84 100

48.85 116.667

48.86 91.6667

48.87 33.3333

48.88 58.3333

48.89 100

48.9 33.3333

48.91 116.667

48.92 83.3333

48.93 83.3333

48.94 58.3333

48.95 75

48.96 58.3333

48.97 66.6667

48.98 91.6667

48.99 108.333

49 108.333

49.01 133.333

49.02 58.3333

49.03 58.3333

49.04 50

49.05 141.667

49.06 116.667

49.07 125

49.08 66.6667

49.09 83.3333

49.1 141.667

49.11 133.333

49.12 83.3333

49.13 41.6667

49.14 100

49.15 141.667

49.16 100

49.17 75

49.18 108.333

49.19 100

49.2 141.667

49.21 100

49.22 116.667

49.23 108.333

49.24 75

49.25 75

49.26 150

49.27 91.6667

49.28 108.333

49.29 50

49.3 158.333

49.31 83.3333

49.32 108.333

49.33 108.333

49.34 66.6667

49.35 83.3333

49.36 100

49.37 91.6667

49.38 83.3333

49.39 100

49.4 125

49.41 116.667

49.42 75

49.43 75

49.44 66.6667

49.45 125

49.46 50

49.47 116.667

49.48 141.667

49.49 33.3333

49.5 83.3333

49.51 100

49.52 83.3333

49.53 50

49.54 100

49.55 83.3333

49.56 116.667

49.57 83.3333

49.58 41.6667

49.59 83.3333

49.6 133.333

49.61 141.667

49.62 75

49.63 91.6667

49.64 133.333

49.65 91.6667

49.66 83.3333

49.67 125

49.68 91.6667

49.69 150

49.7 100

49.71 100

49.72 75

49.73 33.3333

49.74 125

49.75 83.3333

49.76 58.3333

49.77 108.333

49.78 116.667

49.79 91.6667

49.8 100

49.81 116.667

49.82 66.6667

49.83 83.3333

49.84 66.6667

49.85 91.6667

49.86 83.3333

49.87 91.6667

49.88 75

49.89 158.333

49.9 91.6667

49.91 125

49.92 108.333

49.93 133.333

49.94 75

49.95 91.6667

49.96 91.6667

49.97 58.3333

49.98 75

49.99 116.667

50 50

50.01 75

50.02 108.333

50.03 158.333

50.04 125

50.05 175

50.06 83.3333

50.07 75

50.08 133.333

50.09 116.667

50.1 141.667

50.11 83.3333

50.12 100

50.13 91.6667

50.14 83.3333

50.15 75

50.16 75

50.17 125

50.18 50

50.19 91.6667

50.2 116.667

50.21 83.3333

50.22 108.333

50.23 141.667

50.24 58.3333

50.25 125

50.26 83.3333

50.27 83.3333

50.28 83.3333

50.29 108.333

50.3 83.3333

50.31 125

50.32 91.6667

50.33 91.6667

50.34 58.3333

50.35 75

50.36 125

50.37 83.3333

50.38 50

50.39 75

50.4 83.3333

50.41 58.3333

50.42 91.6667

50.43 50

50.44 116.667

50.45 66.6667

50.46 75

50.47 91.6667

50.48 66.6667

50.49 91.6667

50.5 66.6667

50.51 50

50.52 50

50.53 100

50.54 83.3333

50.55 91.6667

50.56 125

50.57 83.3333

50.58 83.3333

50.59 108.333

50.6 91.6667

50.61 116.667

50.62 116.667

50.63 125

50.64 100

50.65 158.333

50.66 125

50.67 133.333

50.68 50

50.69 83.3333

50.7 116.667

50.71 41.6667

50.72 58.3333

50.73 66.6667

50.74 100

50.75 58.3333

50.76 83.3333

50.77 100

50.78 66.6667

50.79 50

50.8 41.6667

50.81 91.6667

50.82 133.333

50.83 108.333

50.84 108.333

50.85 83.3333

50.86 83.3333

50.87 58.3333

50.88 100

50.89 83.3333

50.9 108.333

50.91 125

50.92 58.3333

50.93 125

50.94 83.3333

50.95 116.667

50.96 108.333

50.97 125

50.98 100

50.99 83.3333

51 83.3333

51.01 91.6667

51.02 83.3333

51.03 100

51.04 50

51.05 166.667

51.06 108.333

51.07 91.6667

51.08 133.333

51.09 91.6667

51.1 58.3333

51.11 41.6667

51.12 91.6667

51.13 116.667

51.14 116.667

51.15 116.667

51.16 75

51.17 116.667

51.18 116.667

51.19 83.3333

51.2 116.667

51.21 116.667

51.22 66.6667

51.23 91.6667

51.24 116.667

51.25 83.3333

51.26 125

51.27 100

51.28 116.667

51.29 91.6667

51.3 133.333

51.31 125

51.32 108.333

51.33 108.333

51.34 141.667

51.35 58.3333

51.36 83.3333

51.37 91.6667

51.38 125

51.39 41.6667

51.4 108.333

51.41 100

51.42 66.6667

51.43 50

51.44 116.667

51.45 91.6667

51.46 83.3333

51.47 116.667

51.48 33.3333

51.49 83.3333

51.5 66.6667

51.51 58.3333

51.52 83.3333

51.53 91.6667

51.54 125

51.55 75

51.56 116.667

51.57 91.6667

51.58 150

51.59 66.6667

51.6 100

51.61 116.667

51.62 108.333

51.63 83.3333

51.64 83.3333

51.65 125

51.66 41.6667

51.67 166.667

51.68 66.6667

51.69 116.667

51.7 133.333

51.71 75

51.72 100

51.73 58.3333

51.74 66.6667

51.75 58.3333

51.76 91.6667

51.77 58.3333

51.78 75

51.79 100

51.8 100

51.81 100

51.82 83.3333

51.83 125

51.84 141.667

51.85 58.3333

51.86 125

51.87 100

51.88 100

51.89 133.333

51.9 133.333

51.91 75

51.92 91.6667

51.93 100

51.94 116.667

51.95 116.667

51.96 83.3333

51.97 100

51.98 125

51.99 66.6667

52 108.333

52.01 66.6667

52.02 158.333

52.03 133.333

52.04 116.667

52.05 108.333

52.06 58.3333

52.07 108.333

52.08 100

52.09 58.3333

52.1 83.3333

52.11 66.6667

52.12 75

52.13 91.6667

52.14 66.6667

52.15 116.667

52.16 91.6667

52.17 66.6667

52.18 100

52.19 100

52.2 100

52.21 83.3333

52.22 83.3333

52.23 58.3333

52.24 83.3333

52.25 50

52.26 83.3333

52.27 108.333

52.28 66.6667

52.29 83.3333

52.3 33.3333

52.31 125

52.32 83.3333

52.33 58.3333

52.34 83.3333

52.35 58.3333

52.36 91.6667

52.37 100

52.38 66.6667

52.39 58.3333

52.4 75

52.41 58.3333

52.42 125

52.43 108.333

52.44 100

52.45 116.667

52.46 116.667

52.47 66.6667

52.48 100

52.49 50

52.5 91.6667

52.51 91.6667

52.52 108.333

52.53 66.6667

52.54 100

52.55 41.6667

52.56 75

52.57 108.333

52.58 91.6667

52.59 116.667

52.6 75

52.61 91.6667

52.62 41.6667

52.63 41.6667

52.64 125

52.65 141.667

52.66 83.3333

52.67 141.667

52.68 100

52.69 108.333

52.7 100

52.71 125

52.72 133.333

52.73 75

52.74 91.6667

52.75 108.333

52.76 91.6667

52.77 58.3333

52.78 108.333

52.79 50

52.8 75

52.81 100

52.82 100

52.83 58.3333

52.84 83.3333

52.85 116.667

52.86 108.333

52.87 83.3333

52.88 125

52.89 58.3333

52.9 66.6667

52.91 66.6667

52.92 116.667

52.93 133.333

52.94 100

52.95 66.6667

52.96 100

52.97 75

52.98 108.333

52.99 108.333

53 75

53.01 41.6667

53.02 83.3333

53.03 141.667

53.04 100

53.05 66.6667

53.06 83.3333

53.07 108.333

53.08 41.6667

53.09 75

53.1 166.667

53.11 75

53.12 75

53.13 91.6667

53.14 108.333

53.15 116.667

53.16 100

53.17 108.333

53.18 91.6667

53.19 91.6667

53.2 175

53.21 108.333

53.22 133.333

53.23 91.6667

53.24 83.3333

53.25 50

53.26 116.667

53.27 33.3333

53.28 91.6667

53.29 133.333

53.3 116.667

53.31 91.6667

53.32 108.333

53.33 91.6667

53.34 100

53.35 100

53.36 58.3333

53.37 83.3333

53.38 116.667

53.39 91.6667

53.4 100

53.41 116.667

53.42 100

53.43 91.6667

53.44 108.333

53.45 66.6667

53.46 116.667

53.47 75

53.48 66.6667

53.49 125

53.5 75

53.51 58.3333

53.52 116.667

53.53 58.3333

53.54 91.6667

53.55 100

53.56 125

53.57 91.6667

53.58 125

53.59 100

53.6 66.6667

53.61 83.3333

53.62 116.667

53.63 150

53.64 100

53.65 66.6667

53.66 75

53.67 150

53.68 100

53.69 58.3333

53.7 58.3333

53.71 100

53.72 108.333

53.73 75

53.74 58.3333

53.75 75

53.76 100

53.77 141.667

53.78 91.6667

53.79 150

53.8 66.6667

53.81 91.6667

53.82 100

53.83 66.6667

53.84 125

53.85 116.667

53.86 150

53.87 91.6667

53.88 75

53.89 91.6667

53.9 133.333

53.91 133.333

53.92 158.333

53.93 108.333

53.94 83.3333

53.95 116.667

53.96 91.6667

53.97 83.3333

53.98 108.333

53.99 125

54 41.6667

54.01 66.6667

54.02 100

54.03 108.333

54.04 83.3333

54.05 83.3333

54.06 158.333

54.07 41.6667

54.08 108.333

54.09 141.667

54.1 83.3333

54.11 141.667

54.12 91.6667

54.13 66.6667

54.14 75

54.15 125

54.16 141.667

54.17 75

54.18 83.3333

54.19 100

54.2 75

54.21 150

54.22 91.6667

54.23 83.3333

54.24 83.3333

54.25 91.6667

54.26 116.667

54.27 116.667

54.28 58.3333

54.29 83.3333

54.3 150

54.31 125

54.32 58.3333

54.33 66.6667

54.34 41.6667

54.35 91.6667

54.36 133.333

54.37 75

54.38 75

54.39 100

54.4 125

54.41 66.6667

54.42 66.6667

54.43 108.333

54.44 91.6667

54.45 91.6667

54.46 116.667

54.47 100

54.48 100

54.49 133.333

54.5 141.667

54.51 116.667

54.52 116.667

54.53 91.6667

54.54 83.3333

54.55 75

54.56 33.3333

54.57 33.3333

54.58 75

54.59 150

54.6 133.333

54.61 75

54.62 75

54.63 100

54.64 91.6667

54.65 83.3333

54.66 100

54.67 108.333

54.68 83.3333

54.69 108.333

54.7 125

54.71 66.6667

54.72 83.3333

54.73 100

54.74 75

54.75 58.3333

54.76 66.6667

54.77 83.3333

54.78 75

54.79 100

54.8 91.6667

54.81 41.6667

54.82 91.6667

54.83 125

54.84 108.333

54.85 108.333

54.86 100

54.87 66.6667

54.88 75

54.89 75

54.9 58.3333

54.91 150

54.92 166.667

54.93 58.3333

54.94 133.333

54.95 116.667

54.96 83.3333

54.97 125

54.98 75

54.99 116.667

55 133.333

55.01 116.667

55.02 116.667

55.03 108.333

55.04 150

55.05 66.6667

55.06 91.6667

55.07 141.667

55.08 66.6667

55.09 50

55.1 133.333

55.11 108.333

55.12 50

55.13 116.667

55.14 83.3333

55.15 50

55.16 166.667

55.17 108.333

55.18 100

55.19 91.6667

55.2 83.3333

55.21 150

55.22 141.667

55.23 108.333

55.24 91.6667

55.25 83.3333

55.26 91.6667

55.27 150

55.28 91.6667

55.29 66.6667

55.3 100

55.31 75

55.32 108.333

55.33 83.3333

55.34 58.3333

55.35 108.333

55.36 116.667

55.37 58.3333

55.38 83.3333

55.39 133.333

55.4 91.6667

55.41 141.667

55.42 125

55.43 116.667

55.44 91.6667

55.45 66.6667

55.46 116.667

55.47 116.667

55.48 108.333

55.49 75

55.5 75

55.51 83.3333

55.52 125

55.53 83.3333

55.54 108.333

55.55 133.333

55.56 66.6667

55.57 100

55.58 83.3333

55.59 83.3333

55.6 133.333

55.61 91.6667

55.62 108.333

55.63 125

55.64 41.6667

55.65 41.6667

55.66 41.6667

55.67 66.6667

55.68 66.6667

55.69 75

55.7 83.3333

55.71 91.6667

55.72 66.6667

55.73 50

55.74 108.333

55.75 58.3333

55.76 83.3333

55.77 41.6667

55.78 66.6667

55.79 133.333

55.8 133.333

55.81 100

55.82 150

55.83 41.6667

55.84 66.6667

55.85 83.3333

55.86 66.6667

55.87 91.6667

55.88 150

55.89 125

55.9 100

55.91 116.667

55.92 75

55.93 91.6667

55.94 91.6667

55.95 108.333

55.96 100

55.97 108.333

55.98 83.3333

55.99 125

56 108.333

56.01 100

56.02 125

56.03 91.6667

56.04 141.667

56.05 125

56.06 75

56.07 116.667

56.08 66.6667

56.09 116.667

56.1 91.6667

56.11 91.6667

56.12 66.6667

56.13 83.3333

56.14 125

56.15 100

56.16 91.6667

56.17 75

56.18 116.667

56.19 100

56.2 83.3333

56.21 75

56.22 91.6667

56.23 91.6667

56.24 91.6667

56.25 108.333

56.26 83.3333

56.27 75

56.28 125

56.29 100

56.3 83.3333

56.31 66.6667

56.32 141.667

56.33 83.3333

56.34 108.333

56.35 100

56.36 108.333

56.37 91.6667

56.38 75

56.39 125

56.4 91.6667

56.41 91.6667

56.42 166.667

56.43 75

56.44 41.6667

56.45 116.667

56.46 158.333

56.47 75

56.48 91.6667

56.49 91.6667

56.5 108.333

56.51 66.6667

56.52 66.6667

56.53 166.667

56.54 125

56.55 100

56.56 83.3333

56.57 50

56.58 133.333

56.59 108.333

56.6 66.6667

56.61 100

56.62 58.3333

56.63 141.667

56.64 91.6667

56.65 91.6667

56.66 75

56.67 100

56.68 100

56.69 66.6667

56.7 75

56.71 133.333

56.72 116.667

56.73 100

56.74 66.6667

56.75 183.333

56.76 125

56.77 116.667

56.78 133.333

56.79 66.6667

56.8 133.333

56.81 66.6667

56.82 50

56.83 108.333

56.84 91.6667

56.85 100

56.86 108.333

56.87 50

56.88 108.333

56.89 75

56.9 125

56.91 91.6667

56.92 75

56.93 116.667

56.94 133.333

56.95 116.667

56.96 75

56.97 183.333

56.98 75

56.99 58.3333

57 116.667

57.01 108.333

57.02 83.3333

57.03 83.3333

57.04 100

57.05 41.6667

57.06 75

57.07 83.3333

57.08 83.3333

57.09 58.3333

57.1 133.333

57.11 133.333

57.12 150

57.13 100

57.14 50

57.15 91.6667

57.16 133.333

57.17 116.667

57.18 83.3333

57.19 133.333

57.2 125

57.21 33.3333

57.22 125

57.23 166.667

57.24 116.667

57.25 83.3333

57.26 100

57.27 133.333

57.28 91.6667

57.29 100

57.3 116.667

57.31 83.3333

57.32 100

57.33 41.6667

57.34 58.3333

57.35 116.667

57.36 100

57.37 91.6667

57.38 75

57.39 83.3333

57.4 58.3333

57.41 108.333

57.42 33.3333

57.43 50

57.44 83.3333

57.45 116.667

57.46 75

57.47 66.6667

57.48 100

57.49 100

57.5 133.333

57.51 66.6667

57.52 58.3333

57.53 100

57.54 108.333

57.55 125

57.56 58.3333

57.57 141.667

57.58 83.3333

57.59 108.333

57.6 100

57.61 58.3333

57.62 100

57.63 100

57.64 91.6667

57.65 166.667

57.66 58.3333

57.67 108.333

57.68 108.333

57.69 125

57.7 83.3333

57.71 75

57.72 83.3333

57.73 66.6667

57.74 150

57.75 116.667

57.76 91.6667

57.77 125

57.78 100

57.79 141.667

57.8 75

57.81 108.333

57.82 91.6667

57.83 133.333

57.84 91.6667

57.85 116.667

57.86 66.6667

57.87 75

57.88 100

57.89 83.3333

57.9 108.333

57.91 133.333

57.92 66.6667

57.93 133.333

57.94 141.667

57.95 91.6667

57.96 100

57.97 75

57.98 175

57.99 83.3333

58 100

58.01 83.3333

58.02 91.6667

58.03 141.667

58.04 66.6667

58.05 133.333

58.06 175

58.07 108.333

58.08 125

58.09 83.3333

58.1 75

58.11 58.3333

58.12 150

58.13 100

58.14 66.6667

58.15 133.333

58.16 66.6667

58.17 108.333

58.18 66.6667

58.19 116.667

58.2 150

58.21 125

58.22 125

58.23 125

58.24 116.667

58.25 108.333

58.26 58.3333

58.27 116.667

58.28 108.333

58.29 25

58.3 100

58.31 83.3333

58.32 75

58.33 75

58.34 91.6667

58.35 125

58.36 58.3333

58.37 108.333

58.38 116.667

58.39 133.333

58.4 116.667

58.41 91.6667

58.42 75

58.43 75

58.44 100

58.45 100

58.46 108.333

58.47 100

58.48 133.333

58.49 66.6667

58.5 133.333

58.51 116.667

58.52 141.667

58.53 108.333

58.54 75

58.55 58.3333

58.56 91.6667

58.57 141.667

58.58 141.667

58.59 83.3333

58.6 75

58.61 75

58.62 100

58.63 100

58.64 83.3333

58.65 100

58.66 91.6667

58.67 133.333

58.68 66.6667

58.69 75

58.7 108.333

58.71 83.3333

58.72 108.333

58.73 83.3333

58.74 91.6667

58.75 58.3333

58.76 108.333

58.77 133.333

58.78 100

58.79 58.3333

58.8 150

58.81 91.6667

58.82 50

58.83 75

58.84 91.6667

58.85 116.667

58.86 191.667

58.87 150

58.88 141.667

58.89 100

58.9 108.333

58.91 91.6667

58.92 83.3333

58.93 116.667

58.94 75

58.95 83.3333

58.96 66.6667

58.97 116.667

58.98 66.6667

58.99 116.667

59 133.333

59.01 75

59.02 141.667

59.03 91.6667

59.04 100

59.05 125

59.06 50

59.07 91.6667

59.08 75

59.09 100

59.1 100

59.11 58.3333

59.12 50

59.13 75

59.14 100

59.15 108.333

59.16 116.667

59.17 100

59.18 108.333

59.19 83.3333

59.2 83.3333

59.21 83.3333

59.22 66.6667

59.23 108.333

59.24 83.3333

59.25 133.333

59.26 108.333

59.27 150

59.28 83.3333

59.29 100

59.3 108.333

59.31 75

59.32 175

59.33 100

59.34 125

59.35 141.667

59.36 75

59.37 100

59.38 100

59.39 125

59.4 58.3333

59.41 91.6667

59.42 75

59.43 83.3333

59.44 58.3333

59.45 50

59.46 58.3333

59.47 108.333

59.48 133.333

59.49 125

59.5 108.333

59.51 133.333

59.52 75

59.53 83.3333

59.54 125

59.55 141.667

59.56 108.333

59.57 116.667

59.58 150

59.59 125

59.6 166.667

59.61 108.333

59.62 108.333

59.63 108.333

59.64 66.6667

59.65 125

59.66 141.667

59.67 125

59.68 108.333

59.69 83.3333

59.7 91.6667

59.71 158.333

59.72 125

59.73 133.333

59.74 58.3333

59.75 58.3333

59.76 150

59.77 100

59.78 58.3333

59.79 83.3333

59.8 108.333

59.81 100

59.82 125

59.83 125

59.84 100

59.85 91.6667

59.86 41.6667

59.87 66.6667

59.88 66.6667

59.89 50

59.9 108.333

59.91 141.667

59.92 108.333

59.93 108.333

59.94 116.667

59.95 100

59.96 116.667

59.97 75

59.98 125

59.99 116.667

60 116.667

60.01 100

60.02 125

60.03 116.667

60.04 91.6667

60.05 91.6667

60.06 125

60.07 100

60.08 108.333

60.09 58.3333

60.1 58.3333

60.11 91.6667

60.12 116.667

60.13 116.667

60.14 58.3333

60.15 33.3333

60.16 66.6667

60.17 108.333

60.18 66.6667

60.19 133.333

60.2 133.333

60.21 75

60.22 108.333

60.23 66.6667

60.24 83.3333

60.25 125

60.26 91.6667

60.27 91.6667

60.28 116.667

60.29 58.3333

60.3 116.667

60.31 50

60.32 183.333

60.33 83.3333

60.34 66.6667

60.35 91.6667

60.36 116.667

60.37 75

60.38 75

60.39 66.6667

60.4 125

60.41 116.667

60.42 66.6667

60.43 83.3333

60.44 116.667

60.45 83.3333

60.46 50

60.47 166.667

60.48 133.333

60.49 108.333

60.5 91.6667

60.51 100

60.52 116.667

60.53 108.333

60.54 83.3333

60.55 100

60.56 116.667

60.57 133.333

60.58 100

60.59 100

60.6 183.333

60.61 141.667

60.62 75

60.63 125

60.64 108.333

60.65 83.3333

60.66 83.3333

60.67 91.6667

60.68 108.333

60.69 166.667

60.7 158.333

60.71 83.3333

60.72 100

60.73 141.667

60.74 91.6667

60.75 116.667

60.76 100

60.77 125

60.78 108.333

60.79 75

60.8 75

60.81 116.667

60.82 100

60.83 91.6667

60.84 50

60.85 125

60.86 83.3333

60.87 108.333

60.88 83.3333

60.89 91.6667

60.9 100

60.91 75

60.92 125

60.93 108.333

60.94 100

60.95 116.667

60.96 66.6667

60.97 108.333

60.98 100

60.99 100

61 66.6667

61.01 116.667

61.02 150

61.03 133.333

61.04 66.6667

61.05 125

61.06 191.667

61.07 125

61.08 75

61.09 75

61.1 133.333

61.11 58.3333

61.12 100

61.13 75

61.14 83.3333

61.15 125

61.16 100

61.17 133.333

61.18 91.6667

61.19 108.333

61.2 133.333

61.21 75

61.22 141.667

61.23 100

61.24 116.667

61.25 91.6667

61.26 125

61.27 150

61.28 116.667

61.29 158.333

61.3 108.333

61.31 175

61.32 100

61.33 108.333

61.34 108.333

61.35 116.667

61.36 91.6667

61.37 150

61.38 108.333

61.39 100

61.4 116.667

61.41 108.333

61.42 116.667

61.43 75

61.44 116.667

61.45 41.6667

61.46 133.333

61.47 108.333

61.48 166.667

61.49 100

61.5 108.333

61.51 83.3333

61.52 108.333

61.53 75

61.54 108.333

61.55 100

61.56 108.333

61.57 91.6667

61.58 91.6667

61.59 91.6667

61.6 83.3333

61.61 75

61.62 83.3333

61.63 108.333

61.64 116.667

61.65 83.3333

61.66 150

61.67 100

61.68 133.333

61.69 133.333

61.7 100

61.71 108.333

61.72 108.333

61.73 75

61.74 91.6667

61.75 125

61.76 125

61.77 75

61.78 150

61.79 150

61.8 66.6667

61.81 83.3333

61.82 75

61.83 133.333

61.84 108.333

61.85 183.333

61.86 133.333

61.87 75

61.88 133.333

61.89 141.667

61.9 108.333

61.91 100

61.92 133.333

61.93 116.667

61.94 125

61.95 91.6667

61.96 108.333

61.97 100

61.98 125

61.99 100

62 116.667

62.01 100

62.02 50

62.03 75

62.04 66.6667

62.05 91.6667

62.06 125

62.07 116.667

62.08 150

62.09 91.6667

62.1 41.6667

62.11 108.333

62.12 150

62.13 108.333

62.14 108.333

62.15 100

62.16 91.6667

62.17 58.3333

62.18 91.6667

62.19 83.3333

62.2 191.667

62.21 100

62.22 91.6667

62.23 66.6667

62.24 133.333

62.25 116.667

62.26 91.6667

62.27 125

62.28 91.6667

62.29 66.6667

62.3 150

62.31 58.3333

62.32 141.667

62.33 125

62.34 91.6667

62.35 116.667

62.36 100

62.37 116.667

62.38 125

62.39 108.333

62.4 91.6667

62.41 91.6667

62.42 75

62.43 75

62.44 158.333

62.45 108.333

62.46 150

62.47 91.6667

62.48 100

62.49 83.3333

62.5 116.667

62.51 141.667

62.52 141.667

62.53 141.667

62.54 91.6667

62.55 100

62.56 166.667

62.57 158.333

62.58 83.3333

62.59 116.667

62.6 108.333

62.61 83.3333

62.62 58.3333

62.63 141.667

62.64 125

62.65 125

62.66 133.333

62.67 125

62.68 150

62.69 158.333

62.7 83.3333

62.71 108.333

62.72 158.333

62.73 66.6667

62.74 158.333

62.75 83.3333

62.76 83.3333

62.77 125

62.78 116.667

62.79 116.667

62.8 133.333

62.81 133.333

62.82 100

62.83 116.667

62.84 83.3333

62.85 75

62.86 83.3333

62.87 125

62.88 108.333

62.89 108.333

62.9 133.333

62.91 91.6667

62.92 91.6667

62.93 108.333

62.94 100

62.95 100

62.96 150

62.97 116.667

62.98 100

62.99 100

63 141.667

63.01 116.667

63.02 91.6667

63.03 116.667

63.04 91.6667

63.05 100

63.06 141.667

63.07 125

63.08 100

63.09 75

63.1 75

63.11 66.6667

63.12 141.667

63.13 100

63.14 141.667

63.15 133.333

63.16 91.6667

63.17 108.333

63.18 58.3333

63.19 125

63.2 116.667

63.21 125

63.22 125

63.23 108.333

63.24 108.333

63.25 108.333

63.26 116.667

63.27 116.667

63.28 141.667

63.29 141.667

63.3 150

63.31 100

63.32 91.6667

63.33 66.6667

63.34 100

63.35 83.3333

63.36 100

63.37 83.3333

63.38 108.333

63.39 108.333

63.4 91.6667

63.41 141.667

63.42 133.333

63.43 133.333

63.44 83.3333

63.45 166.667

63.46 83.3333

63.47 100

63.48 116.667

63.49 33.3333

63.5 175

63.51 116.667

63.52 133.333

63.53 100

63.54 108.333

63.55 108.333

63.56 75

63.57 108.333

63.58 116.667

63.59 175

63.6 91.6667

63.61 150

63.62 100

63.63 108.333

63.64 125

63.65 41.6667

63.66 50

63.67 116.667

63.68 83.3333

63.69 108.333

63.7 116.667

63.71 91.6667

63.72 100

63.73 91.6667

63.74 108.333

63.75 125

63.76 100

63.77 100

63.78 125

63.79 150

63.8 150

63.81 133.333

63.82 116.667

63.83 83.3333

63.84 141.667

63.85 125

63.86 125

63.87 58.3333

63.88 91.6667

63.89 108.333

63.9 125

63.91 91.6667

63.92 116.667

63.93 108.333

63.94 108.333

63.95 83.3333

63.96 125

63.97 91.6667

63.98 66.6667

63.99 166.667

64 66.6667

64.01 141.667

64.02 83.3333

64.03 83.3333

64.04 141.667

64.05 133.333

64.06 83.3333

64.07 75

64.08 158.333

64.09 108.333

64.1 150

64.11 125

64.12 125

64.13 83.3333

64.14 133.333

64.15 75

64.16 108.333

64.17 66.6667

64.18 133.333

64.19 125

64.2 66.6667

64.21 133.333

64.22 158.333

64.23 133.333

64.24 100

64.25 91.6667

64.26 108.333

64.27 133.333

64.28 91.6667

64.29 58.3333

64.3 91.6667

64.31 58.3333

64.32 141.667

64.33 91.6667

64.34 141.667

64.35 108.333

64.36 75

64.37 133.333

64.38 116.667

64.39 91.6667

64.4 75

64.41 91.6667

64.42 150

64.43 75

64.44 141.667

64.45 108.333

64.46 125

64.47 116.667

64.48 116.667

64.49 83.3333

64.5 116.667

64.51 141.667

64.52 150

64.53 58.3333

64.54 50

64.55 100

64.56 133.333

64.57 141.667

64.58 125

64.59 75

64.6 91.6667

64.61 133.333

64.62 108.333

64.63 83.3333

64.64 91.6667

64.65 100

64.66 91.6667

64.67 58.3333

64.68 116.667

64.69 83.3333

64.7 83.3333

64.71 100

64.72 108.333

64.73 66.6667

64.74 75

64.75 108.333

64.76 125

64.77 116.667

64.78 116.667

64.79 83.3333

64.8 91.6667

64.81 91.6667

64.82 91.6667

64.83 91.6667

64.84 91.6667

64.85 133.333

64.86 116.667

64.87 100

64.88 125

64.89 133.333

64.9 141.667

64.91 158.333

64.92 91.6667

64.93 100

64.94 100

64.95 100

64.96 108.333

64.97 141.667

64.98 116.667

64.99 125

65 108.333

65.01 116.667

65.02 83.3333

65.03 50

65.04 108.333

65.05 150

65.06 125

65.07 100

65.08 91.6667

65.09 133.333

65.1 125

65.11 100

65.12 141.667

65.13 125

65.14 133.333

65.15 100

65.16 91.6667

65.17 91.6667

65.18 83.3333

65.19 108.333

65.2 100

65.21 183.333

65.22 125

65.23 141.667

65.24 116.667

65.25 133.333

65.26 75

65.27 175

65.28 158.333

65.29 91.6667

65.3 133.333

65.31 83.3333

65.32 108.333

65.33 100

65.34 116.667

65.35 100

65.36 100

65.37 66.6667

65.38 83.3333

65.39 83.3333

65.4 91.6667

65.41 66.6667

65.42 108.333

65.43 166.667

65.44 116.667

65.45 66.6667

65.46 108.333

65.47 83.3333

65.48 66.6667

65.49 116.667

65.5 91.6667

65.51 125

65.52 66.6667

65.53 133.333

65.54 100

65.55 150

65.56 116.667

65.57 91.6667

65.58 166.667

65.59 100

65.6 50

65.61 141.667

65.62 116.667

65.63 116.667

65.64 91.6667

65.65 108.333

65.66 91.6667

65.67 116.667

65.68 91.6667

65.69 100

65.7 100

65.71 125

65.72 116.667

65.73 91.6667

65.74 116.667

65.75 133.333

65.76 141.667

65.77 91.6667

65.78 108.333

65.79 100

65.8 150

65.81 100

65.82 175

65.83 133.333

65.84 141.667

65.85 66.6667

65.86 75

65.87 91.6667

65.88 58.3333

65.89 66.6667

65.9 75

65.91 100

65.92 133.333

65.93 91.6667

65.94 91.6667

65.95 166.667

65.96 75

65.97 133.333

65.98 91.6667

65.99 125

66 91.6667

66.01 100

66.02 116.667

66.03 158.333

66.04 108.333

66.05 100

66.06 100

66.07 133.333

66.08 100

66.09 66.6667

66.1 91.6667

66.11 175

66.12 133.333

66.13 50

66.14 83.3333

66.15 91.6667

66.16 91.6667

66.17 158.333

66.18 91.6667

66.19 100

66.2 100

66.21 75

66.22 125

66.23 91.6667

66.24 141.667

66.25 91.6667

66.26 108.333

66.27 83.3333

66.28 133.333

66.29 108.333

66.3 91.6667

66.31 91.6667

66.32 91.6667

66.33 83.3333

66.34 125

66.35 75

66.36 100

66.37 125

66.38 125

66.39 75

66.4 75

66.41 91.6667

66.42 150

66.43 83.3333

66.44 133.333

66.45 150

66.46 83.3333

66.47 100

66.48 116.667

66.49 125

66.5 141.667

66.51 158.333

66.52 91.6667

66.53 125

66.54 100

66.55 50

66.56 100

66.57 91.6667

66.58 108.333

66.59 100

66.6 141.667

66.61 100

66.62 125

66.63 116.667

66.64 58.3333

66.65 100

66.66 108.333

66.67 125

66.68 75

66.69 116.667

66.7 75

66.71 141.667

66.72 125

66.73 100

66.74 50

66.75 91.6667

66.76 58.3333

66.77 133.333

66.78 58.3333

66.79 91.6667

66.8 125

66.81 108.333

66.82 91.6667

66.83 58.3333

66.84 150

66.85 41.6667

66.86 125

66.87 58.3333

66.88 116.667

66.89 66.6667

66.9 100

66.91 91.6667

66.92 125

66.93 100

66.94 116.667

66.95 41.6667

66.96 91.6667

66.97 75

66.98 100

66.99 91.6667

67 100

67.01 108.333

67.02 83.3333

67.03 133.333

67.04 100

67.05 158.333

67.06 108.333

67.07 125

67.08 141.667

67.09 75

67.1 91.6667

67.11 108.333

67.12 100

67.13 83.3333

67.14 66.6667

67.15 108.333

67.16 83.3333

67.17 108.333

67.18 66.6667

67.19 91.6667

67.2 116.667

67.21 166.667

67.22 141.667

67.23 41.6667

67.24 75

67.25 83.3333

67.26 100

67.27 83.3333

67.28 66.6667

67.29 100

67.3 108.333

67.31 91.6667

67.32 83.3333

67.33 91.6667

67.34 75

67.35 116.667

67.36 66.6667

67.37 91.6667

67.38 91.6667

67.39 66.6667

67.4 83.3333

67.41 100

67.42 116.667

67.43 125

67.44 125

67.45 116.667

67.46 125

67.47 91.6667

67.48 83.3333

67.49 66.6667

67.5 108.333

67.51 116.667

67.52 58.3333

67.53 100

67.54 91.6667

67.55 125

67.56 150

67.57 66.6667

67.58 75

67.59 108.333

67.6 75

67.61 125

67.62 75

67.63 91.6667

67.64 33.3333

67.65 175

67.66 108.333

67.67 91.6667

67.68 108.333

67.69 83.3333

67.7 141.667

67.71 100

67.72 100

67.73 75

67.74 108.333

67.75 75

67.76 116.667

67.77 83.3333

67.78 116.667

67.79 133.333

67.8 91.6667

67.81 100

67.82 108.333

67.83 50

67.84 150

67.85 50

67.86 83.3333

67.87 58.3333

67.88 83.3333

67.89 141.667

67.9 75

67.91 100

67.92 116.667

67.93 108.333

67.94 66.6667

67.95 100

67.96 108.333

67.97 125

67.98 66.6667

67.99 100

68 150

68.01 41.6667

68.02 133.333

68.03 66.6667

68.04 116.667

68.05 91.6667

68.06 75

68.07 100

68.08 75

68.09 75

68.1 125

68.11 108.333

68.12 66.6667

68.13 116.667

68.14 100

68.15 75

68.16 75

68.17 108.333

68.18 83.3333

68.19 91.6667

68.2 141.667

68.21 125

68.22 75

68.23 41.6667

68.24 91.6667

68.25 75

68.26 108.333

68.27 116.667

68.28 50

68.29 158.333

68.3 141.667

68.31 75

68.32 83.3333

68.33 66.6667

68.34 125

68.35 75

68.36 133.333

68.37 141.667

68.38 116.667

68.39 75

68.4 100

68.41 108.333

68.42 108.333

68.43 66.6667

68.44 100

68.45 83.3333

68.46 133.333

68.47 91.6667

68.48 116.667

68.49 83.3333

68.5 150

68.51 75

68.52 66.6667

68.53 108.333

68.54 66.6667

68.55 100

68.56 133.333

68.57 100

68.58 133.333

68.59 75

68.6 133.333

68.61 125

68.62 125

68.63 66.6667

68.64 116.667

68.65 141.667

68.66 75

68.67 75

68.68 116.667

68.69 66.6667

68.7 116.667

68.71 50

68.72 91.6667

68.73 66.6667

68.74 50

68.75 83.3333

68.76 91.6667

68.77 75

68.78 58.3333

68.79 75

68.8 58.3333

68.81 100

68.82 58.3333

68.83 125

68.84 66.6667

68.85 75

68.86 100

68.87 91.6667

68.88 58.3333

68.89 166.667

68.9 100

68.91 91.6667

68.92 116.667

68.93 100

68.94 116.667

68.95 41.6667

68.96 108.333

68.97 108.333

68.98 75

68.99 125

69 100

69.01 108.333

69.02 133.333

69.03 116.667

69.04 125

69.05 108.333

69.06 141.667

69.07 58.3333

69.08 108.333

69.09 100

69.1 116.667

69.11 133.333

69.12 133.333

69.13 50

69.14 66.6667

69.15 75

69.16 66.6667

69.17 50

69.18 91.6667

69.19 58.3333

69.2 58.3333

69.21 75

69.22 83.3333

69.23 100

69.24 75

69.25 141.667

69.26 108.333

69.27 100

69.28 100

69.29 66.6667

69.3 108.333

69.31 91.6667

69.32 50

69.33 83.3333

69.34 116.667

69.35 83.3333

69.36 83.3333

69.37 108.333

69.38 91.6667

69.39 58.3333

69.4 83.3333

69.41 133.333

69.42 125

69.43 125

69.44 75

69.45 133.333

69.46 66.6667

69.47 58.3333

69.48 108.333

69.49 91.6667

69.5 58.3333

69.51 83.3333

69.52 91.6667

69.53 100

69.54 108.333

69.55 75

69.56 125

69.57 83.3333

69.58 75

69.59 91.6667

69.6 83.3333

69.61 125

69.62 83.3333

69.63 58.3333

69.64 108.333

69.65 83.3333

69.66 66.6667

69.67 91.6667

69.68 41.6667

69.69 83.3333

69.7 75

69.71 141.667

69.72 91.6667

69.73 100

69.74 150

69.75 108.333

69.76 116.667

69.77 133.333

69.78 66.6667

69.79 100

69.8 91.6667

69.81 100

69.82 50

69.83 83.3333

69.84 141.667

69.85 66.6667

69.86 125

69.87 108.333

69.88 91.6667

69.89 133.333

69.9 133.333

69.91 58.3333

69.92 108.333

69.93 58.3333

69.94 116.667

69.95 83.3333

69.96 50

69.97 58.3333

69.98 108.333

69.99 91.6667

70 150

**Raw data 4**. XRD raw data of the powder obtained after heat-treatment of as-sprayed powders prepared from the solution with dextrin precursors at 450 ℃.

Goniometer RINT2000 vertical goniometer

Attachment Auto sample changer type B(6 samples)

Monochromater Fixed Monochromator

ScanningMode 2Theta/Theta

ScanningType Continuos Scanning

X-Ray 40kV/100mA

DivSlit 1 deg.

DivH.L.Slit 10mm

SctSlit 1 deg.

RecSlit 0.15mm

Monochro RS No Use

K-beta filter

Start 20

Stop 70

Step 0.01

20 666.667

20.01 425

20.02 583.333

20.03 516.667

20.04 491.667

20.05 475

20.06 441.667

20.07 608.333

20.08 658.333

20.09 566.667

20.1 408.333

20.11 566.667

20.12 525

20.13 700

20.14 466.667

20.15 600

20.16 658.333

20.17 433.333

20.18 491.667

20.19 525

20.2 650

20.21 575

20.22 600

20.23 550

20.24 600

20.25 466.667

20.26 800

20.27 450

20.28 550

20.29 558.333

20.3 575

20.31 566.667

20.32 633.333

20.33 550

20.34 475

20.35 558.333

20.36 633.333

20.37 616.667

20.38 633.333

20.39 533.333

20.4 575

20.41 625

20.42 533.333

20.43 533.333

20.44 566.667

20.45 508.333

20.46 600

20.47 541.667

20.48 600

20.49 616.667

20.5 591.667

20.51 641.667

20.52 458.333

20.53 491.667

20.54 541.667

20.55 600

20.56 575

20.57 525

20.58 591.667

20.59 583.333

20.6 541.667

20.61 683.333

20.62 558.333

20.63 675

20.64 633.333

20.65 608.333

20.66 625

20.67 566.667

20.68 716.667

20.69 575

20.7 425

20.71 566.667

20.72 550

20.73 608.333

20.74 641.667

20.75 658.333

20.76 675

20.77 575

20.78 475

20.79 550

20.8 550

20.81 658.333

20.82 491.667

20.83 683.333

20.84 525

20.85 733.333

20.86 666.667

20.87 425

20.88 691.667

20.89 591.667

20.9 575

20.91 533.333

20.92 566.667

20.93 608.333

20.94 633.333

20.95 591.667

20.96 591.667

20.97 466.667

20.98 516.667

20.99 550

21 608.333

21.01 616.667

21.02 558.333

21.03 466.667

21.04 658.333

21.05 575

21.06 558.333

21.07 733.333

21.08 691.667

21.09 533.333

21.1 775

21.11 666.667

21.12 616.667

21.13 725

21.14 575

21.15 541.667

21.16 608.333

21.17 541.667

21.18 691.667

21.19 541.667

21.2 575

21.21 508.333

21.22 558.333

21.23 550

21.24 583.333

21.25 575

21.26 641.667

21.27 525

21.28 641.667

21.29 675

21.3 525

21.31 666.667

21.32 608.333

21.33 491.667

21.34 558.333

21.35 700

21.36 675

21.37 525

21.38 650

21.39 633.333

21.4 625

21.41 666.667

21.42 516.667

21.43 441.667

21.44 566.667

21.45 483.333

21.46 533.333

21.47 666.667

21.48 675

21.49 641.667

21.5 666.667

21.51 508.333

21.52 608.333

21.53 516.667

21.54 525

21.55 641.667

21.56 708.333

21.57 733.333

21.58 541.667

21.59 591.667

21.6 600

21.61 533.333

21.62 558.333

21.63 450

21.64 658.333

21.65 508.333

21.66 508.333

21.67 483.333

21.68 566.667

21.69 541.667

21.7 566.667

21.71 666.667

21.72 641.667

21.73 500

21.74 658.333

21.75 616.667

21.76 475

21.77 641.667

21.78 641.667

21.79 533.333

21.8 600

21.81 566.667

21.82 666.667

21.83 575

21.84 550

21.85 491.667

21.86 525

21.87 508.333

21.88 516.667

21.89 591.667

21.9 541.667

21.91 558.333

21.92 616.667

21.93 508.333

21.94 633.333

21.95 666.667

21.96 483.333

21.97 658.333

21.98 533.333

21.99 533.333

22 541.667

22.01 566.667

22.02 533.333

22.03 625

22.04 575

22.05 516.667

22.06 608.333

22.07 500

22.08 516.667

22.09 566.667

22.1 491.667

22.11 566.667

22.12 433.333

22.13 608.333

22.14 525

22.15 683.333

22.16 566.667

22.17 541.667

22.18 508.333

22.19 466.667

22.2 491.667

22.21 550

22.22 691.667

22.23 466.667

22.24 491.667

22.25 575

22.26 575

22.27 625

22.28 441.667

22.29 591.667

22.3 433.333

22.31 583.333

22.32 516.667

22.33 408.333

22.34 633.333

22.35 608.333

22.36 475

22.37 600

22.38 633.333

22.39 491.667

22.4 483.333

22.41 608.333

22.42 658.333

22.43 575

22.44 533.333

22.45 516.667

22.46 558.333

22.47 558.333

22.48 583.333

22.49 633.333

22.5 633.333

22.51 583.333

22.52 575

22.53 450

22.54 475

22.55 525

22.56 466.667

22.57 583.333

22.58 433.333

22.59 466.667

22.6 491.667

22.61 550

22.62 533.333

22.63 516.667

22.64 450

22.65 458.333

22.66 550

22.67 483.333

22.68 541.667

22.69 541.667

22.7 466.667

22.71 558.333

22.72 500

22.73 516.667

22.74 458.333

22.75 658.333

22.76 525

22.77 600

22.78 516.667

22.79 658.333

22.8 666.667

22.81 458.333

22.82 416.667

22.83 508.333

22.84 516.667

22.85 583.333

22.86 508.333

22.87 491.667

22.88 466.667

22.89 525

22.9 491.667

22.91 450

22.92 491.667

22.93 583.333

22.94 558.333

22.95 416.667

22.96 491.667

22.97 491.667

22.98 400

22.99 466.667

23 458.333

23.01 383.333

23.02 441.667

23.03 575

23.04 516.667

23.05 425

23.06 441.667

23.07 458.333

23.08 441.667

23.09 491.667

23.1 466.667

23.11 591.667

23.12 491.667

23.13 400

23.14 541.667

23.15 508.333

23.16 375

23.17 575

23.18 433.333

23.19 483.333

23.2 450

23.21 491.667

23.22 516.667

23.23 575

23.24 458.333

23.25 550

23.26 633.333

23.27 416.667

23.28 441.667

23.29 491.667

23.3 500

23.31 450

23.32 550

23.33 408.333

23.34 500

23.35 325

23.36 383.333

23.37 400

23.38 350

23.39 450

23.4 375

23.41 408.333

23.42 416.667

23.43 383.333

23.44 425

23.45 508.333

23.46 550

23.47 466.667

23.48 458.333

23.49 408.333

23.5 491.667

23.51 491.667

23.52 425

23.53 491.667

23.54 408.333

23.55 341.667

23.56 341.667

23.57 483.333

23.58 500

23.59 383.333

23.6 383.333

23.61 416.667

23.62 500

23.63 325

23.64 425

23.65 383.333

23.66 308.333

23.67 366.667

23.68 341.667

23.69 416.667

23.7 516.667

23.71 441.667

23.72 483.333

23.73 408.333

23.74 325

23.75 375

23.76 458.333

23.77 333.333

23.78 341.667

23.79 450

23.8 441.667

23.81 366.667

23.82 341.667

23.83 341.667

23.84 583.333

23.85 433.333

23.86 483.333

23.87 350

23.88 291.667

23.89 383.333

23.9 508.333

23.91 425

23.92 391.667

23.93 433.333

23.94 433.333

23.95 333.333

23.96 475

23.97 358.333

23.98 300

23.99 475

24 391.667

24.01 383.333

24.02 350

24.03 383.333

24.04 383.333

24.05 316.667

24.06 483.333

24.07 425

24.08 383.333

24.09 475

24.1 308.333

24.11 550

24.12 450

24.13 550

24.14 491.667

24.15 291.667

24.16 516.667

24.17 491.667

24.18 366.667

24.19 450

24.2 525

24.21 375

24.22 408.333

24.23 366.667

24.24 341.667

24.25 466.667

24.26 541.667

24.27 383.333

24.28 408.333

24.29 391.667

24.3 383.333

24.31 308.333

24.32 416.667

24.33 425

24.34 383.333

24.35 308.333

24.36 425

24.37 341.667

24.38 408.333

24.39 475

24.4 508.333

24.41 400

24.42 441.667

24.43 375

24.44 416.667

24.45 391.667

24.46 333.333

24.47 383.333

24.48 341.667

24.49 366.667

24.5 441.667

24.51 458.333

24.52 450

24.53 266.667

24.54 400

24.55 350

24.56 341.667

24.57 358.333

24.58 358.333

24.59 458.333

24.6 341.667

24.61 375

24.62 433.333

24.63 375

24.64 416.667

24.65 350

24.66 416.667

24.67 391.667

24.68 416.667

24.69 408.333

24.7 400

24.71 266.667

24.72 433.333

24.73 341.667

24.74 366.667

24.75 400

24.76 358.333

24.77 441.667

24.78 391.667

24.79 333.333

24.8 491.667

24.81 416.667

24.82 375

24.83 391.667

24.84 308.333

24.85 375

24.86 325

24.87 250

24.88 475

24.89 300

24.9 325

24.91 341.667

24.92 358.333

24.93 283.333

24.94 425

24.95 300

24.96 425

24.97 441.667

24.98 325

24.99 325

25 341.667

25.01 366.667

25.02 425

25.03 341.667

25.04 341.667

25.05 433.333

25.06 358.333

25.07 325

25.08 466.667

25.09 308.333

25.1 358.333

25.11 383.333

25.12 325

25.13 325

25.14 375

25.15 441.667

25.16 266.667

25.17 300

25.18 291.667

25.19 358.333

25.2 450

25.21 391.667

25.22 333.333

25.23 441.667

25.24 416.667

25.25 341.667

25.26 325

25.27 300

25.28 375

25.29 316.667

25.3 433.333

25.31 308.333

25.32 300

25.33 425

25.34 341.667

25.35 341.667

25.36 300

25.37 375

25.38 391.667

25.39 333.333

25.4 333.333

25.41 325

25.42 291.667

25.43 333.333

25.44 300

25.45 316.667

25.46 358.333

25.47 291.667

25.48 275

25.49 350

25.5 316.667

25.51 258.333

25.52 233.333

25.53 316.667

25.54 308.333

25.55 325

25.56 366.667

25.57 275

25.58 275

25.59 341.667

25.6 200

25.61 266.667

25.62 266.667

25.63 191.667

25.64 258.333

25.65 316.667

25.66 350

25.67 325

25.68 308.333

25.69 275

25.7 350

25.71 358.333

25.72 391.667

25.73 300

25.74 258.333

25.75 283.333

25.76 333.333

25.77 316.667

25.78 341.667

25.79 333.333

25.8 241.667

25.81 333.333

25.82 400

25.83 283.333

25.84 316.667

25.85 350

25.86 325

25.87 208.333

25.88 316.667

25.89 341.667

25.9 300

25.91 341.667

25.92 233.333

25.93 266.667

25.94 316.667

25.95 316.667

25.96 308.333

25.97 333.333

25.98 400

25.99 375

26 283.333

26.01 308.333

26.02 300

26.03 283.333

26.04 216.667

26.05 350

26.06 275

26.07 325

26.08 208.333

26.09 325

26.1 266.667

26.11 375

26.12 325

26.13 250

26.14 300

26.15 275

26.16 291.667

26.17 325

26.18 275

26.19 325

26.2 308.333

26.21 258.333

26.22 291.667

26.23 316.667

26.24 308.333

26.25 325

26.26 250

26.27 291.667

26.28 266.667

26.29 258.333

26.3 266.667

26.31 333.333

26.32 275

26.33 241.667

26.34 291.667

26.35 358.333

26.36 250

26.37 258.333

26.38 216.667

26.39 233.333

26.4 250

26.41 283.333

26.42 350

26.43 325

26.44 283.333

26.45 333.333

26.46 333.333

26.47 266.667

26.48 208.333

26.49 283.333

26.5 333.333

26.51 275

26.52 250

26.53 258.333

26.54 258.333

26.55 333.333

26.56 308.333

26.57 275

26.58 225

26.59 241.667

26.6 291.667

26.61 266.667

26.62 225

26.63 258.333

26.64 341.667

26.65 300

26.66 258.333

26.67 241.667

26.68 258.333

26.69 333.333

26.7 233.333

26.71 233.333

26.72 291.667

26.73 275

26.74 200

26.75 333.333

26.76 241.667

26.77 300

26.78 241.667

26.79 241.667

26.8 266.667

26.81 266.667

26.82 200

26.83 166.667

26.84 241.667

26.85 291.667

26.86 208.333

26.87 275

26.88 300

26.89 358.333

26.9 216.667

26.91 250

26.92 258.333

26.93 275

26.94 291.667

26.95 283.333

26.96 183.333

26.97 275

26.98 250

26.99 300

27 241.667

27.01 333.333

27.02 233.333

27.03 333.333

27.04 200

27.05 275

27.06 233.333

27.07 233.333

27.08 241.667

27.09 300

27.1 233.333

27.11 258.333

27.12 275

27.13 216.667

27.14 250

27.15 225

27.16 300

27.17 225

27.18 200

27.19 283.333

27.2 183.333

27.21 233.333

27.22 233.333

27.23 241.667

27.24 158.333

27.25 225

27.26 300

27.27 250

27.28 275

27.29 283.333

27.3 275

27.31 175

27.32 316.667

27.33 308.333

27.34 241.667

27.35 241.667

27.36 241.667

27.37 241.667

27.38 350

27.39 258.333

27.4 216.667

27.41 208.333

27.42 216.667

27.43 150

27.44 250

27.45 166.667

27.46 291.667

27.47 183.333

27.48 275

27.49 266.667

27.5 266.667

27.51 225

27.52 225

27.53 250

27.54 258.333

27.55 291.667

27.56 208.333

27.57 233.333

27.58 250

27.59 325

27.6 233.333

27.61 250

27.62 225

27.63 241.667

27.64 216.667

27.65 291.667

27.66 316.667

27.67 250

27.68 191.667

27.69 275

27.7 200

27.71 225

27.72 166.667

27.73 166.667

27.74 166.667

27.75 266.667

27.76 191.667

27.77 233.333

27.78 250

27.79 233.333

27.8 275

27.81 258.333

27.82 208.333

27.83 283.333

27.84 241.667

27.85 208.333

27.86 250

27.87 291.667

27.88 158.333

27.89 241.667

27.9 266.667

27.91 266.667

27.92 266.667

27.93 225

27.94 191.667

27.95 200

27.96 291.667

27.97 250

27.98 116.667

27.99 200

28 191.667

28.01 200

28.02 250

28.03 283.333

28.04 250

28.05 166.667

28.06 208.333

28.07 158.333

28.08 275

28.09 258.333

28.1 258.333

28.11 216.667

28.12 166.667

28.13 250

28.14 241.667

28.15 216.667

28.16 233.333

28.17 166.667

28.18 316.667

28.19 250

28.2 291.667

28.21 266.667

28.22 258.333

28.23 200

28.24 316.667

28.25 241.667

28.26 266.667

28.27 241.667

28.28 241.667

28.29 233.333

28.3 216.667

28.31 233.333

28.32 200

28.33 241.667

28.34 233.333

28.35 291.667

28.36 150

28.37 191.667

28.38 233.333

28.39 208.333

28.4 175

28.41 208.333

28.42 183.333

28.43 200

28.44 175

28.45 233.333

28.46 208.333

28.47 283.333

28.48 208.333

28.49 233.333

28.5 258.333

28.51 166.667

28.52 208.333

28.53 241.667

28.54 266.667

28.55 275

28.56 216.667

28.57 250

28.58 191.667

28.59 191.667

28.6 191.667

28.61 208.333

28.62 183.333

28.63 191.667

28.64 208.333

28.65 266.667

28.66 258.333

28.67 258.333

28.68 233.333

28.69 291.667

28.7 216.667

28.71 225

28.72 183.333

28.73 158.333

28.74 225

28.75 216.667

28.76 308.333

28.77 175

28.78 158.333

28.79 200

28.8 266.667

28.81 200

28.82 200

28.83 150

28.84 216.667

28.85 250

28.86 283.333

28.87 241.667

28.88 241.667

28.89 216.667

28.9 183.333

28.91 275

28.92 183.333

28.93 141.667

28.94 233.333

28.95 241.667

28.96 208.333

28.97 216.667

28.98 225

28.99 233.333

29 216.667

29.01 216.667

29.02 233.333

29.03 225

29.04 225

29.05 258.333

29.06 241.667

29.07 266.667

29.08 183.333

29.09 258.333

29.1 191.667

29.11 216.667

29.12 225

29.13 233.333

29.14 200

29.15 200

29.16 283.333

29.17 158.333

29.18 291.667

29.19 200

29.2 166.667

29.21 166.667

29.22 166.667

29.23 258.333

29.24 233.333

29.25 225

29.26 141.667

29.27 233.333

29.28 183.333

29.29 183.333

29.3 208.333

29.31 216.667

29.32 258.333

29.33 208.333

29.34 175

29.35 200

29.36 183.333

29.37 291.667

29.38 141.667

29.39 225

29.4 258.333

29.41 225

29.42 166.667

29.43 225

29.44 191.667

29.45 275

29.46 175

29.47 166.667

29.48 191.667

29.49 175

29.5 233.333

29.51 241.667

29.52 258.333

29.53 241.667

29.54 350

29.55 183.333

29.56 258.333

29.57 225

29.58 191.667

29.59 258.333

29.6 141.667

29.61 200

29.62 175

29.63 258.333

29.64 108.333

29.65 250

29.66 225

29.67 225

29.68 133.333

29.69 258.333

29.7 216.667

29.71 216.667

29.72 258.333

29.73 191.667

29.74 216.667

29.75 141.667

29.76 108.333

29.77 225

29.78 191.667

29.79 191.667

29.8 108.333

29.81 175

29.82 233.333

29.83 258.333

29.84 200

29.85 200

29.86 266.667

29.87 175

29.88 191.667

29.89 225

29.9 200

29.91 191.667

29.92 200

29.93 183.333

29.94 258.333

29.95 250

29.96 216.667

29.97 241.667

29.98 208.333

29.99 216.667

30 216.667

30.01 191.667

30.02 208.333

30.03 208.333

30.04 183.333

30.05 200

30.06 250

30.07 208.333

30.08 183.333

30.09 200

30.1 191.667

30.11 200

30.12 166.667

30.13 200

30.14 175

30.15 116.667

30.16 200

30.17 183.333

30.18 216.667

30.19 200

30.2 175

30.21 175

30.22 241.667

30.23 150

30.24 233.333

30.25 166.667

30.26 133.333

30.27 183.333

30.28 200

30.29 216.667

30.3 191.667

30.31 225

30.32 200

30.33 150

30.34 200

30.35 225

30.36 166.667

30.37 183.333

30.38 225

30.39 216.667

30.4 175

30.41 166.667

30.42 150

30.43 275

30.44 183.333

30.45 141.667

30.46 150

30.47 166.667

30.48 133.333

30.49 175

30.5 208.333

30.51 133.333

30.52 125

30.53 208.333

30.54 175

30.55 200

30.56 100

30.57 233.333

30.58 275

30.59 241.667

30.6 191.667

30.61 125

30.62 125

30.63 150

30.64 166.667

30.65 191.667

30.66 200

30.67 175

30.68 191.667

30.69 233.333

30.7 258.333

30.71 141.667

30.72 216.667

30.73 175

30.74 200

30.75 183.333

30.76 250

30.77 158.333

30.78 166.667

30.79 216.667

30.8 141.667

30.81 191.667

30.82 175

30.83 275

30.84 175

30.85 283.333

30.86 150

30.87 158.333

30.88 191.667

30.89 225

30.9 125

30.91 200

30.92 166.667

30.93 216.667

30.94 183.333

30.95 191.667

30.96 166.667

30.97 208.333

30.98 225

30.99 175

31 183.333

31.01 233.333

31.02 116.667

31.03 233.333

31.04 175

31.05 183.333

31.06 183.333

31.07 175

31.08 183.333

31.09 200

31.1 208.333

31.11 191.667

31.12 233.333

31.13 208.333

31.14 175

31.15 116.667

31.16 158.333

31.17 150

31.18 158.333

31.19 216.667

31.2 216.667

31.21 241.667

31.22 158.333

31.23 150

31.24 208.333

31.25 191.667

31.26 158.333

31.27 191.667

31.28 191.667

31.29 133.333

31.3 208.333

31.31 183.333

31.32 200

31.33 125

31.34 166.667

31.35 158.333

31.36 183.333

31.37 158.333

31.38 191.667

31.39 166.667

31.4 225

31.41 133.333

31.42 150

31.43 133.333

31.44 116.667

31.45 225

31.46 241.667

31.47 241.667

31.48 183.333

31.49 133.333

31.5 58.3333

31.51 125

31.52 150

31.53 141.667

31.54 200

31.55 150

31.56 183.333

31.57 150

31.58 208.333

31.59 183.333

31.6 175

31.61 158.333

31.62 100

31.63 183.333

31.64 200

31.65 233.333

31.66 200

31.67 166.667

31.68 150

31.69 175

31.7 141.667

31.71 166.667

31.72 175

31.73 208.333

31.74 116.667

31.75 183.333

31.76 166.667

31.77 133.333

31.78 191.667

31.79 250

31.8 125

31.81 200

31.82 166.667

31.83 200

31.84 175

31.85 241.667

31.86 175

31.87 166.667

31.88 208.333

31.89 175

31.9 183.333

31.91 141.667

31.92 216.667

31.93 91.6667

31.94 200

31.95 158.333

31.96 208.333

31.97 166.667

31.98 225

31.99 208.333

32 175

32.01 158.333

32.02 266.667

32.03 166.667

32.04 216.667

32.05 141.667

32.06 150

32.07 166.667

32.08 191.667

32.09 216.667

32.1 183.333

32.11 150

32.12 125

32.13 166.667

32.14 225

32.15 200

32.16 183.333

32.17 166.667

32.18 208.333

32.19 141.667

32.2 158.333

32.21 258.333

32.22 208.333

32.23 200

32.24 150

32.25 133.333

32.26 225

32.27 183.333

32.28 225

32.29 166.667

32.3 125

32.31 133.333

32.32 175

32.33 183.333

32.34 191.667

32.35 108.333

32.36 150

32.37 108.333

32.38 141.667

32.39 200

32.4 141.667

32.41 233.333

32.42 141.667

32.43 141.667

32.44 166.667

32.45 166.667

32.46 200

32.47 150

32.48 158.333

32.49 133.333

32.5 125

32.51 141.667

32.52 125

32.53 258.333

32.54 191.667

32.55 191.667

32.56 158.333

32.57 100

32.58 158.333

32.59 158.333

32.6 208.333

32.61 66.6667

32.62 183.333

32.63 166.667

32.64 200

32.65 166.667

32.66 183.333

32.67 100

32.68 183.333

32.69 108.333

32.7 233.333

32.71 125

32.72 175

32.73 83.3333

32.74 216.667

32.75 191.667

32.76 108.333

32.77 150

32.78 133.333

32.79 183.333

32.8 175

32.81 166.667

32.82 166.667

32.83 158.333

32.84 158.333

32.85 150

32.86 183.333

32.87 141.667

32.88 158.333

32.89 166.667

32.9 183.333

32.91 200

32.92 216.667

32.93 166.667

32.94 100

32.95 158.333

32.96 175

32.97 141.667

32.98 150

32.99 191.667

33 200

33.01 191.667

33.02 191.667

33.03 166.667

33.04 141.667

33.05 191.667

33.06 183.333

33.07 208.333

33.08 150

33.09 150

33.1 158.333

33.11 175

33.12 175

33.13 116.667

33.14 116.667

33.15 116.667

33.16 83.3333

33.17 91.6667

33.18 150

33.19 116.667

33.2 158.333

33.21 150

33.22 183.333

33.23 225

33.24 191.667

33.25 175

33.26 125

33.27 183.333

33.28 200

33.29 175

33.3 141.667

33.31 141.667

33.32 175

33.33 150

33.34 183.333

33.35 116.667

33.36 191.667

33.37 200

33.38 150

33.39 116.667

33.4 150

33.41 183.333

33.42 116.667

33.43 125

33.44 158.333

33.45 166.667

33.46 125

33.47 158.333

33.48 175

33.49 175

33.5 125

33.51 100

33.52 225

33.53 158.333

33.54 200

33.55 133.333

33.56 183.333

33.57 200

33.58 216.667

33.59 116.667

33.6 216.667

33.61 233.333

33.62 200

33.63 141.667

33.64 100

33.65 175

33.66 191.667

33.67 191.667

33.68 158.333

33.69 158.333

33.7 175

33.71 150

33.72 216.667

33.73 216.667

33.74 166.667

33.75 200

33.76 150

33.77 116.667

33.78 166.667

33.79 208.333

33.8 91.6667

33.81 191.667

33.82 191.667

33.83 191.667

33.84 166.667

33.85 183.333

33.86 191.667

33.87 175

33.88 233.333

33.89 141.667

33.9 125

33.91 191.667

33.92 225

33.93 175

33.94 208.333

33.95 108.333

33.96 216.667

33.97 141.667

33.98 158.333

33.99 175

34 141.667

34.01 116.667

34.02 175

34.03 150

34.04 166.667

34.05 141.667

34.06 283.333

34.07 125

34.08 183.333

34.09 166.667

34.1 166.667

34.11 150

34.12 158.333

34.13 125

34.14 158.333

34.15 108.333

34.16 175

34.17 216.667

34.18 108.333

34.19 108.333

34.2 141.667

34.21 216.667

34.22 150

34.23 266.667

34.24 158.333

34.25 158.333

34.26 183.333

34.27 158.333

34.28 133.333

34.29 100

34.3 166.667

34.31 116.667

34.32 133.333

34.33 158.333

34.34 158.333

34.35 175

34.36 141.667

34.37 133.333

34.38 166.667

34.39 133.333

34.4 158.333

34.41 133.333

34.42 133.333

34.43 150

34.44 225

34.45 183.333

34.46 108.333

34.47 133.333

34.48 133.333

34.49 100

34.5 125

34.51 108.333

34.52 216.667

34.53 158.333

34.54 141.667

34.55 216.667

34.56 150

34.57 133.333

34.58 200

34.59 150

34.6 175

34.61 141.667

34.62 175

34.63 141.667

34.64 166.667

34.65 216.667

34.66 166.667

34.67 216.667

34.68 158.333

34.69 258.333

34.7 183.333

34.71 108.333

34.72 183.333

34.73 191.667

34.74 208.333

34.75 191.667

34.76 191.667

34.77 183.333

34.78 216.667

34.79 141.667

34.8 191.667

34.81 175

34.82 200

34.83 83.3333

34.84 225

34.85 216.667

34.86 233.333

34.87 191.667

34.88 150

34.89 200

34.9 141.667

34.91 183.333

34.92 150

34.93 183.333

34.94 175

34.95 200

34.96 125

34.97 150

34.98 166.667

34.99 141.667

35 125

35.01 208.333

35.02 191.667

35.03 200

35.04 183.333

35.05 158.333

35.06 250

35.07 216.667

35.08 233.333

35.09 183.333

35.1 200

35.11 175

35.12 141.667

35.13 133.333

35.14 150

35.15 116.667

35.16 175

35.17 141.667

35.18 208.333

35.19 208.333

35.2 191.667

35.21 233.333

35.22 225

35.23 200

35.24 166.667

35.25 183.333

35.26 191.667

35.27 225

35.28 175

35.29 133.333

35.3 266.667

35.31 225

35.32 150

35.33 141.667

35.34 200

35.35 133.333

35.36 150

35.37 133.333

35.38 150

35.39 125

35.4 200

35.41 141.667

35.42 208.333

35.43 141.667

35.44 225

35.45 141.667

35.46 200

35.47 233.333

35.48 100

35.49 191.667

35.5 191.667

35.51 175

35.52 100

35.53 150

35.54 158.333

35.55 158.333

35.56 216.667

35.57 200

35.58 208.333

35.59 233.333

35.6 133.333

35.61 125

35.62 166.667

35.63 158.333

35.64 166.667

35.65 141.667

35.66 166.667

35.67 141.667

35.68 216.667

35.69 200

35.7 208.333

35.71 208.333

35.72 175

35.73 133.333

35.74 191.667

35.75 200

35.76 191.667

35.77 191.667

35.78 175

35.79 150

35.8 116.667

35.81 200

35.82 150

35.83 225

35.84 116.667

35.85 91.6667

35.86 125

35.87 125

35.88 183.333

35.89 158.333

35.9 133.333

35.91 175

35.92 266.667

35.93 175

35.94 150

35.95 108.333

35.96 133.333

35.97 258.333

35.98 166.667

35.99 150

36 158.333

36.01 208.333

36.02 191.667

36.03 125

36.04 183.333

36.05 141.667

36.06 158.333

36.07 141.667

36.08 150

36.09 125

36.1 208.333

36.11 225

36.12 141.667

36.13 208.333

36.14 216.667

36.15 208.333

36.16 166.667

36.17 141.667

36.18 141.667

36.19 116.667

36.2 175

36.21 100

36.22 183.333

36.23 108.333

36.24 191.667

36.25 158.333

36.26 141.667

36.27 125

36.28 108.333

36.29 191.667

36.3 150

36.31 175

36.32 175

36.33 166.667

36.34 166.667

36.35 158.333

36.36 150

36.37 158.333

36.38 158.333

36.39 125

36.4 166.667

36.41 166.667

36.42 150

36.43 158.333

36.44 166.667

36.45 116.667

36.46 66.6667

36.47 141.667

36.48 191.667

36.49 133.333

36.5 158.333

36.51 175

36.52 216.667

36.53 175

36.54 191.667

36.55 216.667

36.56 141.667

36.57 150

36.58 208.333

36.59 116.667

36.6 166.667

36.61 150

36.62 158.333

36.63 91.6667

36.64 108.333

36.65 150

36.66 91.6667

36.67 158.333

36.68 100

36.69 216.667

36.7 150

36.71 100

36.72 241.667

36.73 125

36.74 133.333

36.75 108.333

36.76 133.333

36.77 91.6667

36.78 116.667

36.79 216.667

36.8 166.667

36.81 166.667

36.82 100

36.83 150

36.84 100

36.85 158.333

36.86 116.667

36.87 166.667

36.88 141.667

36.89 150

36.9 141.667

36.91 125

36.92 175

36.93 116.667

36.94 166.667

36.95 108.333

36.96 133.333

36.97 108.333

36.98 200

36.99 150

37 116.667

37.01 166.667

37.02 100

37.03 108.333

37.04 125

37.05 141.667

37.06 125

37.07 116.667

37.08 133.333

37.09 133.333

37.1 125

37.11 75

37.12 166.667

37.13 108.333

37.14 108.333

37.15 66.6667

37.16 200

37.17 150

37.18 100

37.19 83.3333

37.2 141.667

37.21 175

37.22 125

37.23 116.667

37.24 150

37.25 108.333

37.26 91.6667

37.27 108.333

37.28 166.667

37.29 141.667

37.3 133.333

37.31 166.667

37.32 100

37.33 141.667

37.34 133.333

37.35 175

37.36 108.333

37.37 41.6667

37.38 91.6667

37.39 125

37.4 158.333

37.41 150

37.42 116.667

37.43 100

37.44 200

37.45 125

37.46 133.333

37.47 116.667

37.48 116.667

37.49 125

37.5 100

37.51 100

37.52 175

37.53 133.333

37.54 108.333

37.55 116.667

37.56 116.667

37.57 75

37.58 150

37.59 175

37.6 158.333

37.61 158.333

37.62 66.6667

37.63 150

37.64 141.667

37.65 83.3333

37.66 125

37.67 100

37.68 116.667

37.69 100

37.7 108.333

37.71 141.667

37.72 200

37.73 141.667

37.74 75

37.75 133.333

37.76 116.667

37.77 150

37.78 116.667

37.79 125

37.8 166.667

37.81 125

37.82 75

37.83 108.333

37.84 116.667

37.85 141.667

37.86 141.667

37.87 158.333

37.88 133.333

37.89 108.333

37.9 191.667

37.91 58.3333

37.92 100

37.93 150

37.94 141.667

37.95 125

37.96 125

37.97 125

37.98 133.333

37.99 141.667

38 108.333

38.01 100

38.02 75

38.03 100

38.04 191.667

38.05 108.333

38.06 141.667

38.07 150

38.08 75

38.09 75

38.1 125

38.11 133.333

38.12 116.667

38.13 75

38.14 125

38.15 116.667

38.16 83.3333

38.17 150

38.18 100

38.19 166.667

38.2 158.333

38.21 41.6667

38.22 116.667

38.23 133.333

38.24 150

38.25 100

38.26 91.6667

38.27 108.333

38.28 108.333

38.29 133.333

38.3 100

38.31 183.333

38.32 166.667

38.33 133.333

38.34 50

38.35 116.667

38.36 150

38.37 58.3333

38.38 125

38.39 91.6667

38.4 91.6667

38.41 108.333

38.42 116.667

38.43 108.333

38.44 150

38.45 175

38.46 133.333

38.47 83.3333

38.48 41.6667

38.49 166.667

38.5 75

38.51 116.667

38.52 66.6667

38.53 150

38.54 83.3333

38.55 116.667

38.56 125

38.57 108.333

38.58 108.333

38.59 150

38.6 75

38.61 58.3333

38.62 100

38.63 191.667

38.64 116.667

38.65 125

38.66 125

38.67 116.667

38.68 150

38.69 66.6667

38.7 158.333

38.71 158.333

38.72 83.3333

38.73 200

38.74 116.667

38.75 158.333

38.76 133.333

38.77 91.6667

38.78 108.333

38.79 50

38.8 83.3333

38.81 133.333

38.82 108.333

38.83 150

38.84 108.333

38.85 116.667

38.86 175

38.87 83.3333

38.88 141.667

38.89 125

38.9 100

38.91 100

38.92 91.6667

38.93 100

38.94 166.667

38.95 100

38.96 133.333

38.97 83.3333

38.98 108.333

38.99 150

39 116.667

39.01 100

39.02 125

39.03 75

39.04 108.333

39.05 91.6667

39.06 141.667

39.07 75

39.08 91.6667

39.09 108.333

39.1 91.6667

39.11 175

39.12 100

39.13 91.6667

39.14 108.333

39.15 75

39.16 58.3333

39.17 116.667

39.18 91.6667

39.19 108.333

39.2 116.667

39.21 150

39.22 150

39.23 100

39.24 166.667

39.25 83.3333

39.26 133.333

39.27 166.667

39.28 150

39.29 75

39.3 75

39.31 125

39.32 100

39.33 125

39.34 91.6667

39.35 100

39.36 100

39.37 50

39.38 133.333

39.39 125

39.4 133.333

39.41 150

39.42 50

39.43 125

39.44 91.6667

39.45 100

39.46 66.6667

39.47 108.333

39.48 75

39.49 133.333

39.5 116.667

39.51 150

39.52 58.3333

39.53 100

39.54 83.3333

39.55 133.333

39.56 125

39.57 100

39.58 108.333

39.59 108.333

39.6 108.333

39.61 141.667

39.62 108.333

39.63 83.3333

39.64 58.3333

39.65 133.333

39.66 158.333

39.67 33.3333

39.68 83.3333

39.69 83.3333

39.7 100

39.71 50

39.72 75

39.73 166.667

39.74 125

39.75 108.333

39.76 116.667

39.77 91.6667

39.78 91.6667

39.79 116.667

39.8 133.333

39.81 91.6667

39.82 66.6667

39.83 100

39.84 66.6667

39.85 50

39.86 83.3333

39.87 100

39.88 150

39.89 75

39.9 91.6667

39.91 100

39.92 141.667

39.93 91.6667

39.94 100

39.95 125

39.96 83.3333

39.97 116.667

39.98 100

39.99 66.6667

40 133.333

40.01 75

40.02 116.667

40.03 125

40.04 108.333

40.05 91.6667

40.06 75

40.07 150

40.08 116.667

40.09 50

40.1 116.667

40.11 58.3333

40.12 83.3333

40.13 91.6667

40.14 75

40.15 50

40.16 108.333

40.17 116.667

40.18 108.333

40.19 133.333

40.2 100

40.21 91.6667

40.22 75

40.23 108.333

40.24 125

40.25 133.333

40.26 108.333

40.27 100

40.28 41.6667

40.29 83.3333

40.3 83.3333

40.31 108.333

40.32 108.333

40.33 58.3333

40.34 108.333

40.35 108.333

40.36 150

40.37 50

40.38 158.333

40.39 83.3333

40.4 66.6667

40.41 108.333

40.42 108.333

40.43 100

40.44 41.6667

40.45 125

40.46 158.333

40.47 125

40.48 66.6667

40.49 91.6667

40.5 91.6667

40.51 58.3333

40.52 66.6667

40.53 75

40.54 83.3333

40.55 91.6667

40.56 100

40.57 166.667

40.58 91.6667

40.59 58.3333

40.6 108.333

40.61 158.333

40.62 75

40.63 83.3333

40.64 100

40.65 141.667

40.66 91.6667

40.67 133.333

40.68 41.6667

40.69 133.333

40.7 141.667

40.71 158.333

40.72 75

40.73 58.3333

40.74 116.667

40.75 91.6667

40.76 91.6667

40.77 75

40.78 58.3333

40.79 50

40.8 100

40.81 108.333

40.82 91.6667

40.83 83.3333

40.84 75

40.85 125

40.86 116.667

40.87 75

40.88 75

40.89 141.667

40.9 100

40.91 133.333

40.92 75

40.93 116.667

40.94 125

40.95 58.3333

40.96 83.3333

40.97 83.3333

40.98 100

40.99 83.3333

41 116.667

41.01 100

41.02 100

41.03 125

41.04 108.333

41.05 100

41.06 91.6667

41.07 75

41.08 100

41.09 116.667

41.1 91.6667

41.11 175

41.12 50

41.13 100

41.14 141.667

41.15 116.667

41.16 83.3333

41.17 91.6667

41.18 100

41.19 58.3333

41.2 116.667

41.21 66.6667

41.22 108.333

41.23 100

41.24 66.6667

41.25 83.3333

41.26 108.333

41.27 100

41.28 83.3333

41.29 141.667

41.3 108.333

41.31 83.3333

41.32 66.6667

41.33 75

41.34 108.333

41.35 125

41.36 66.6667

41.37 108.333

41.38 108.333

41.39 125

41.4 141.667

41.41 133.333

41.42 125

41.43 91.6667

41.44 108.333

41.45 133.333

41.46 158.333

41.47 100

41.48 116.667

41.49 100

41.5 150

41.51 108.333

41.52 108.333

41.53 100

41.54 158.333

41.55 75

41.56 141.667

41.57 100

41.58 91.6667

41.59 116.667

41.6 116.667

41.61 116.667

41.62 158.333

41.63 100

41.64 108.333

41.65 116.667

41.66 100

41.67 83.3333

41.68 108.333

41.69 100

41.7 133.333

41.71 25

41.72 133.333

41.73 116.667

41.74 91.6667

41.75 100

41.76 66.6667

41.77 83.3333

41.78 125

41.79 91.6667

41.8 133.333

41.81 133.333

41.82 125

41.83 66.6667

41.84 91.6667

41.85 66.6667

41.86 91.6667

41.87 141.667

41.88 66.6667

41.89 116.667

41.9 116.667

41.91 75

41.92 108.333

41.93 100

41.94 75

41.95 158.333

41.96 75

41.97 116.667

41.98 58.3333

41.99 75

42 116.667

42.01 125

42.02 166.667

42.03 91.6667

42.04 100

42.05 91.6667

42.06 100

42.07 108.333

42.08 141.667

42.09 91.6667

42.1 125

42.11 108.333

42.12 108.333

42.13 91.6667

42.14 100

42.15 100

42.16 100

42.17 125

42.18 58.3333

42.19 133.333

42.2 133.333

42.21 125

42.22 66.6667

42.23 133.333

42.24 108.333

42.25 66.6667

42.26 158.333

42.27 150

42.28 175

42.29 116.667

42.3 150

42.31 91.6667

42.32 108.333

42.33 141.667

42.34 66.6667

42.35 91.6667

42.36 75

42.37 100

42.38 91.6667

42.39 141.667

42.4 116.667

42.41 158.333

42.42 108.333

42.43 108.333

42.44 125

42.45 158.333

42.46 125

42.47 125

42.48 100

42.49 116.667

42.5 141.667

42.51 100

42.52 108.333

42.53 91.6667

42.54 91.6667

42.55 116.667

42.56 100

42.57 125

42.58 91.6667

42.59 141.667

42.6 66.6667

42.61 108.333

42.62 91.6667

42.63 133.333

42.64 100

42.65 133.333

42.66 100

42.67 141.667

42.68 83.3333

42.69 116.667

42.7 125

42.71 116.667

42.72 100

42.73 75

42.74 116.667

42.75 75

42.76 150

42.77 116.667

42.78 66.6667

42.79 75

42.8 91.6667

42.81 150

42.82 100

42.83 50

42.84 83.3333

42.85 66.6667

42.86 91.6667

42.87 75

42.88 108.333

42.89 100

42.9 100

42.91 83.3333

42.92 125

42.93 83.3333

42.94 25

42.95 91.6667

42.96 83.3333

42.97 91.6667

42.98 83.3333

42.99 125

43 183.333

43.01 100

43.02 100

43.03 91.6667

43.04 166.667

43.05 133.333

43.06 116.667

43.07 75

43.08 100

43.09 150

43.1 141.667

43.11 125

43.12 83.3333

43.13 83.3333

43.14 116.667

43.15 75

43.16 75

43.17 83.3333

43.18 100

43.19 141.667

43.2 83.3333

43.21 133.333

43.22 83.3333

43.23 91.6667

43.24 91.6667

43.25 141.667

43.26 150

43.27 141.667

43.28 116.667

43.29 100

43.3 116.667

43.31 125

43.32 150

43.33 150

43.34 150

43.35 75

43.36 75

43.37 125

43.38 91.6667

43.39 175

43.4 116.667

43.41 91.6667

43.42 66.6667

43.43 150

43.44 150

43.45 150

43.46 141.667

43.47 100

43.48 125

43.49 125

43.5 116.667

43.51 116.667

43.52 150

43.53 50

43.54 141.667

43.55 150

43.56 100

43.57 91.6667

43.58 125

43.59 75

43.6 100

43.61 116.667

43.62 91.6667

43.63 108.333

43.64 141.667

43.65 108.333

43.66 91.6667

43.67 133.333

43.68 125

43.69 133.333

43.7 100

43.71 133.333

43.72 91.6667

43.73 125

43.74 91.6667

43.75 41.6667

43.76 91.6667

43.77 91.6667

43.78 100

43.79 158.333

43.8 133.333

43.81 141.667

43.82 108.333

43.83 108.333

43.84 91.6667

43.85 83.3333

43.86 133.333

43.87 108.333

43.88 83.3333

43.89 125

43.9 75

43.91 100

43.92 141.667

43.93 91.6667

43.94 83.3333

43.95 83.3333

43.96 150

43.97 100

43.98 108.333

43.99 91.6667

44 91.6667

44.01 125

44.02 133.333

44.03 108.333

44.04 83.3333

44.05 100

44.06 100

44.07 108.333

44.08 100

44.09 83.3333

44.1 125

44.11 133.333

44.12 75

44.13 100

44.14 91.6667

44.15 150

44.16 141.667

44.17 108.333

44.18 125

44.19 141.667

44.2 66.6667

44.21 108.333

44.22 133.333

44.23 100

44.24 75

44.25 108.333

44.26 125

44.27 116.667

44.28 125

44.29 116.667

44.3 125

44.31 66.6667

44.32 116.667

44.33 83.3333

44.34 116.667

44.35 125

44.36 91.6667

44.37 75

44.38 133.333

44.39 100

44.4 166.667

44.41 141.667

44.42 125

44.43 75

44.44 91.6667

44.45 125

44.46 191.667

44.47 100

44.48 125

44.49 158.333

44.5 116.667

44.51 200

44.52 166.667

44.53 225

44.54 233.333

44.55 233.333

44.56 233.333

44.57 183.333

44.58 233.333

44.59 266.667

44.6 333.333

44.61 416.667

44.62 383.333

44.63 483.333

44.64 483.333

44.65 508.333

44.66 575

44.67 558.333

44.68 575

44.69 466.667

44.7 400

44.71 341.667

44.72 341.667

44.73 250

44.74 283.333

44.75 291.667

44.76 250

44.77 366.667

44.78 333.333

44.79 308.333

44.8 208.333

44.81 191.667

44.82 200

44.83 225

44.84 208.333

44.85 191.667

44.86 141.667

44.87 116.667

44.88 150

44.89 125

44.9 166.667

44.91 133.333

44.92 108.333

44.93 141.667

44.94 141.667

44.95 133.333

44.96 166.667

44.97 116.667

44.98 75

44.99 100

45 108.333

45.01 100

45.02 66.6667

45.03 125

45.04 91.6667

45.05 75

45.06 58.3333

45.07 108.333

45.08 150

45.09 150

45.1 150

45.11 100

45.12 75

45.13 150

45.14 141.667

45.15 116.667

45.16 116.667

45.17 108.333

45.18 116.667

45.19 175

45.2 91.6667

45.21 108.333

45.22 116.667

45.23 108.333

45.24 133.333

45.25 116.667

45.26 83.3333

45.27 175

45.28 100

45.29 133.333

45.3 66.6667

45.31 91.6667

45.32 66.6667

45.33 66.6667

45.34 75

45.35 116.667

45.36 50

45.37 91.6667

45.38 41.6667

45.39 100

45.4 141.667

45.41 83.3333

45.42 75

45.43 66.6667

45.44 108.333

45.45 75

45.46 116.667

45.47 108.333

45.48 41.6667

45.49 191.667

45.5 133.333

45.51 83.3333

45.52 125

45.53 83.3333

45.54 116.667

45.55 66.6667

45.56 108.333

45.57 116.667

45.58 108.333

45.59 66.6667

45.6 108.333

45.61 108.333

45.62 91.6667

45.63 125

45.64 108.333

45.65 41.6667

45.66 100

45.67 141.667

45.68 75

45.69 133.333

45.7 41.6667

45.71 125

45.72 83.3333

45.73 75

45.74 75

45.75 116.667

45.76 116.667

45.77 50

45.78 91.6667

45.79 108.333

45.8 75

45.81 150

45.82 41.6667

45.83 108.333

45.84 100

45.85 100

45.86 91.6667

45.87 75

45.88 66.6667

45.89 150

45.9 91.6667

45.91 75

45.92 66.6667

45.93 83.3333

45.94 116.667

45.95 91.6667

45.96 100

45.97 75

45.98 100

45.99 100

46 141.667

46.01 75

46.02 150

46.03 75

46.04 116.667

46.05 91.6667

46.06 125

46.07 166.667

46.08 100

46.09 58.3333

46.1 100

46.11 133.333

46.12 83.3333

46.13 108.333

46.14 133.333

46.15 91.6667

46.16 125

46.17 66.6667

46.18 100

46.19 91.6667

46.2 100

46.21 91.6667

46.22 41.6667

46.23 158.333

46.24 141.667

46.25 108.333

46.26 100

46.27 83.3333

46.28 116.667

46.29 108.333

46.3 83.3333

46.31 125

46.32 100

46.33 91.6667

46.34 75

46.35 116.667

46.36 83.3333

46.37 116.667

46.38 108.333

46.39 75

46.4 75

46.41 91.6667

46.42 116.667

46.43 100

46.44 50

46.45 100

46.46 50

46.47 150

46.48 100

46.49 58.3333

46.5 141.667

46.51 91.6667

46.52 125

46.53 108.333

46.54 66.6667

46.55 83.3333

46.56 133.333

46.57 83.3333

46.58 75

46.59 100

46.6 50

46.61 75

46.62 100

46.63 91.6667

46.64 83.3333

46.65 75

46.66 100

46.67 75

46.68 100

46.69 83.3333

46.7 116.667

46.71 75

46.72 133.333

46.73 125

46.74 141.667

46.75 108.333

46.76 66.6667

46.77 83.3333

46.78 91.6667

46.79 100

46.8 91.6667

46.81 91.6667

46.82 58.3333

46.83 166.667

46.84 125

46.85 75

46.86 100

46.87 66.6667

46.88 116.667

46.89 25

46.9 100

46.91 83.3333

46.92 58.3333

46.93 158.333

46.94 91.6667

46.95 58.3333

46.96 108.333

46.97 108.333

46.98 91.6667

46.99 125

47 108.333

47.01 58.3333

47.02 58.3333

47.03 125

47.04 125

47.05 175

47.06 83.3333

47.07 116.667

47.08 133.333

47.09 91.6667

47.1 50

47.11 83.3333

47.12 83.3333

47.13 58.3333

47.14 116.667

47.15 75

47.16 91.6667

47.17 75

47.18 100

47.19 75

47.2 125

47.21 100

47.22 133.333

47.23 91.6667

47.24 100

47.25 125

47.26 75

47.27 108.333

47.28 150

47.29 116.667

47.3 58.3333

47.31 91.6667

47.32 58.3333

47.33 91.6667

47.34 83.3333

47.35 50

47.36 83.3333

47.37 108.333

47.38 133.333

47.39 83.3333

47.4 58.3333

47.41 75

47.42 125

47.43 100

47.44 108.333

47.45 116.667

47.46 116.667

47.47 108.333

47.48 133.333

47.49 58.3333

47.5 58.3333

47.51 108.333

47.52 141.667

47.53 91.6667

47.54 100

47.55 83.3333

47.56 33.3333

47.57 116.667

47.58 125

47.59 91.6667

47.6 83.3333

47.61 91.6667

47.62 125

47.63 83.3333

47.64 108.333

47.65 141.667

47.66 91.6667

47.67 50

47.68 91.6667

47.69 58.3333

47.7 83.3333

47.71 75

47.72 83.3333

47.73 75

47.74 83.3333

47.75 83.3333

47.76 125

47.77 83.3333

47.78 108.333

47.79 91.6667

47.8 91.6667

47.81 58.3333

47.82 91.6667

47.83 91.6667

47.84 66.6667

47.85 91.6667

47.86 66.6667

47.87 125

47.88 108.333

47.89 83.3333

47.9 66.6667

47.91 91.6667

47.92 75

47.93 66.6667

47.94 58.3333

47.95 91.6667

47.96 116.667

47.97 66.6667

47.98 58.3333

47.99 41.6667

48 75

48.01 66.6667

48.02 100

48.03 108.333

48.04 83.3333

48.05 108.333

48.06 91.6667

48.07 116.667

48.08 100

48.09 58.3333

48.1 75

48.11 83.3333

48.12 58.3333

48.13 58.3333

48.14 100

48.15 100

48.16 108.333

48.17 116.667

48.18 75

48.19 116.667

48.2 100

48.21 83.3333

48.22 125

48.23 108.333

48.24 66.6667

48.25 50

48.26 100

48.27 108.333

48.28 41.6667

48.29 75

48.3 83.3333

48.31 83.3333

48.32 108.333

48.33 66.6667

48.34 75

48.35 100

48.36 116.667

48.37 75

48.38 100

48.39 91.6667

48.4 91.6667

48.41 58.3333

48.42 75

48.43 75

48.44 100

48.45 91.6667

48.46 108.333

48.47 66.6667

48.48 75

48.49 133.333

48.5 100

48.51 133.333

48.52 83.3333

48.53 91.6667

48.54 100

48.55 83.3333

48.56 66.6667

48.57 108.333

48.58 133.333

48.59 91.6667

48.6 116.667

48.61 100

48.62 116.667

48.63 58.3333

48.64 108.333

48.65 66.6667

48.66 58.3333

48.67 83.3333

48.68 75

48.69 108.333

48.7 116.667

48.71 125

48.72 83.3333

48.73 116.667

48.74 116.667

48.75 150

48.76 108.333

48.77 83.3333

48.78 108.333

48.79 100

48.8 58.3333

48.81 91.6667

48.82 75

48.83 91.6667

48.84 83.3333

48.85 66.6667

48.86 91.6667

48.87 50

48.88 66.6667

48.89 83.3333

48.9 100

48.91 50

48.92 116.667

48.93 41.6667

48.94 75

48.95 100

48.96 100

48.97 66.6667

48.98 91.6667

48.99 50

49 66.6667

49.01 58.3333

49.02 116.667

49.03 141.667

49.04 75

49.05 58.3333

49.06 91.6667

49.07 50

49.08 91.6667

49.09 50

49.1 100

49.11 125

49.12 83.3333

49.13 91.6667

49.14 83.3333

49.15 133.333

49.16 66.6667

49.17 133.333

49.18 108.333

49.19 100

49.2 91.6667

49.21 141.667

49.22 100

49.23 108.333

49.24 58.3333

49.25 75

49.26 116.667

49.27 75

49.28 50

49.29 116.667

49.3 50

49.31 75

49.32 125

49.33 108.333

49.34 91.6667

49.35 58.3333

49.36 66.6667

49.37 158.333

49.38 108.333

49.39 116.667

49.4 75

49.41 100

49.42 108.333

49.43 83.3333

49.44 100

49.45 100

49.46 91.6667

49.47 58.3333

49.48 66.6667

49.49 141.667

49.5 66.6667

49.51 100

49.52 108.333

49.53 83.3333

49.54 91.6667

49.55 108.333

49.56 66.6667

49.57 66.6667

49.58 100

49.59 108.333

49.6 83.3333

49.61 83.3333

49.62 58.3333

49.63 133.333

49.64 66.6667

49.65 75

49.66 125

49.67 133.333

49.68 91.6667

49.69 183.333

49.7 41.6667

49.71 91.6667

49.72 66.6667

49.73 141.667

49.74 58.3333

49.75 50

49.76 83.3333

49.77 83.3333

49.78 133.333

49.79 108.333

49.8 75

49.81 83.3333

49.82 83.3333

49.83 91.6667

49.84 141.667

49.85 58.3333

49.86 100

49.87 83.3333

49.88 66.6667

49.89 83.3333

49.9 58.3333

49.91 83.3333

49.92 58.3333

49.93 108.333

49.94 91.6667

49.95 91.6667

49.96 100

49.97 100

49.98 50

49.99 66.6667

50 141.667

50.01 100

50.02 91.6667

50.03 100

50.04 58.3333

50.05 41.6667

50.06 141.667

50.07 116.667

50.08 83.3333

50.09 100

50.1 116.667

50.11 116.667

50.12 125

50.13 66.6667

50.14 100

50.15 125

50.16 58.3333

50.17 75

50.18 66.6667

50.19 91.6667

50.2 133.333

50.21 41.6667

50.22 108.333

50.23 100

50.24 108.333

50.25 58.3333

50.26 91.6667

50.27 100

50.28 91.6667

50.29 100

50.3 75

50.31 133.333

50.32 83.3333

50.33 91.6667

50.34 58.3333

50.35 91.6667

50.36 108.333

50.37 141.667

50.38 83.3333

50.39 66.6667

50.4 83.3333

50.41 58.3333

50.42 75

50.43 75

50.44 83.3333

50.45 58.3333

50.46 108.333

50.47 83.3333

50.48 58.3333

50.49 50

50.5 75

50.51 116.667

50.52 100

50.53 83.3333

50.54 91.6667

50.55 133.333

50.56 75

50.57 83.3333

50.58 83.3333

50.59 50

50.6 83.3333

50.61 58.3333

50.62 108.333

50.63 158.333

50.64 58.3333

50.65 50

50.66 116.667

50.67 125

50.68 75

50.69 125

50.7 108.333

50.71 125

50.72 116.667

50.73 108.333

50.74 116.667

50.75 91.6667

50.76 91.6667

50.77 83.3333

50.78 125

50.79 100

50.8 41.6667

50.81 116.667

50.82 83.3333

50.83 66.6667

50.84 91.6667

50.85 83.3333

50.86 75

50.87 83.3333

50.88 91.6667

50.89 66.6667

50.9 108.333

50.91 100

50.92 133.333

50.93 108.333

50.94 75

50.95 66.6667

50.96 108.333

50.97 58.3333

50.98 75

50.99 108.333

51 116.667

51.01 50

51.02 116.667

51.03 108.333

51.04 58.3333

51.05 58.3333

51.06 100

51.07 91.6667

51.08 116.667

51.09 91.6667

51.1 66.6667

51.11 91.6667

51.12 83.3333

51.13 50

51.14 141.667

51.15 58.3333

51.16 83.3333

51.17 58.3333

51.18 150

51.19 108.333

51.2 75

51.21 125

51.22 75

51.23 91.6667

51.24 91.6667

51.25 41.6667

51.26 125

51.27 91.6667

51.28 116.667

51.29 108.333

51.3 91.6667

51.31 83.3333

51.32 83.3333

51.33 66.6667

51.34 50

51.35 91.6667

51.36 133.333

51.37 108.333

51.38 75

51.39 116.667

51.4 83.3333

51.41 75

51.42 100

51.43 91.6667

51.44 91.6667

51.45 91.6667

51.46 133.333

51.47 141.667

51.48 108.333

51.49 75

51.5 100

51.51 41.6667

51.52 75

51.53 116.667

51.54 91.6667

51.55 41.6667

51.56 91.6667

51.57 66.6667

51.58 66.6667

51.59 58.3333

51.6 50

51.61 125

51.62 75

51.63 141.667

51.64 125

51.65 116.667

51.66 58.3333

51.67 125

51.68 75

51.69 191.667

51.7 108.333

51.71 83.3333

51.72 83.3333

51.73 108.333

51.74 108.333

51.75 100

51.76 141.667

51.77 75

51.78 83.3333

51.79 16.6667

51.8 116.667

51.81 83.3333

51.82 100

51.83 75

51.84 58.3333

51.85 75

51.86 66.6667

51.87 166.667

51.88 75

51.89 116.667

51.9 175

51.91 100

51.92 58.3333

51.93 108.333

51.94 108.333

51.95 58.3333

51.96 50

51.97 150

51.98 83.3333

51.99 108.333

52 91.6667

52.01 100

52.02 125

52.03 83.3333

52.04 100

52.05 91.6667

52.06 50

52.07 75

52.08 116.667

52.09 75

52.1 66.6667

52.11 108.333

52.12 133.333

52.13 141.667

52.14 66.6667

52.15 108.333

52.16 83.3333

52.17 141.667

52.18 75

52.19 100

52.2 66.6667

52.21 133.333

52.22 141.667

52.23 75

52.24 100

52.25 108.333

52.26 83.3333

52.27 83.3333

52.28 75

52.29 125

52.3 91.6667

52.31 58.3333

52.32 116.667

52.33 108.333

52.34 125

52.35 108.333

52.36 50

52.37 83.3333

52.38 50

52.39 41.6667

52.4 125

52.41 108.333

52.42 83.3333

52.43 91.6667

52.44 91.6667

52.45 66.6667

52.46 50

52.47 83.3333

52.48 91.6667

52.49 75

52.5 66.6667

52.51 91.6667

52.52 100

52.53 116.667

52.54 100

52.55 75

52.56 100

52.57 100

52.58 125

52.59 150

52.6 50

52.61 91.6667

52.62 75

52.63 150

52.64 116.667

52.65 125

52.66 58.3333

52.67 83.3333

52.68 133.333

52.69 125

52.7 91.6667

52.71 108.333

52.72 108.333

52.73 91.6667

52.74 83.3333

52.75 83.3333

52.76 50

52.77 66.6667

52.78 133.333

52.79 108.333

52.8 133.333

52.81 91.6667

52.82 83.3333

52.83 91.6667

52.84 91.6667

52.85 75

52.86 58.3333

52.87 166.667

52.88 141.667

52.89 83.3333

52.9 141.667

52.91 100

52.92 75

52.93 91.6667

52.94 125

52.95 133.333

52.96 66.6667

52.97 75

52.98 108.333

52.99 125

53 83.3333

53.01 66.6667

53.02 58.3333

53.03 50

53.04 100

53.05 125

53.06 116.667

53.07 75

53.08 100

53.09 50

53.1 100

53.11 91.6667

53.12 116.667

53.13 91.6667

53.14 100

53.15 41.6667

53.16 91.6667

53.17 133.333

53.18 66.6667

53.19 91.6667

53.2 75

53.21 141.667

53.22 91.6667

53.23 108.333

53.24 58.3333

53.25 133.333

53.26 116.667

53.27 158.333

53.28 91.6667

53.29 83.3333

53.3 91.6667

53.31 91.6667

53.32 91.6667

53.33 100

53.34 116.667

53.35 58.3333

53.36 141.667

53.37 116.667

53.38 75

53.39 116.667

53.4 116.667

53.41 83.3333

53.42 116.667

53.43 83.3333

53.44 125

53.45 83.3333

53.46 41.6667

53.47 50

53.48 50

53.49 83.3333

53.5 108.333

53.51 125

53.52 41.6667

53.53 91.6667

53.54 116.667

53.55 75

53.56 108.333

53.57 83.3333

53.58 83.3333

53.59 91.6667

53.6 100

53.61 116.667

53.62 116.667

53.63 125

53.64 66.6667

53.65 100

53.66 191.667

53.67 50

53.68 133.333

53.69 66.6667

53.7 83.3333

53.71 75

53.72 125

53.73 83.3333

53.74 116.667

53.75 75

53.76 91.6667

53.77 66.6667

53.78 83.3333

53.79 50

53.8 75

53.81 100

53.82 108.333

53.83 75

53.84 100

53.85 66.6667

53.86 50

53.87 66.6667

53.88 91.6667

53.89 58.3333

53.9 41.6667

53.91 58.3333

53.92 133.333

53.93 50

53.94 100

53.95 58.3333

53.96 75

53.97 83.3333

53.98 125

53.99 100

54 83.3333

54.01 158.333

54.02 116.667

54.03 83.3333

54.04 66.6667

54.05 116.667

54.06 175

54.07 75

54.08 141.667

54.09 58.3333

54.1 100

54.11 83.3333

54.12 66.6667

54.13 91.6667

54.14 100

54.15 91.6667

54.16 116.667

54.17 58.3333

54.18 116.667

54.19 125

54.2 141.667

54.21 100

54.22 83.3333

54.23 75

54.24 83.3333

54.25 141.667

54.26 91.6667

54.27 75

54.28 91.6667

54.29 83.3333

54.3 66.6667

54.31 83.3333

54.32 116.667

54.33 91.6667

54.34 66.6667

54.35 100

54.36 108.333

54.37 91.6667

54.38 108.333

54.39 100

54.4 58.3333

54.41 66.6667

54.42 83.3333

54.43 66.6667

54.44 41.6667

54.45 116.667

54.46 91.6667

54.47 91.6667

54.48 75

54.49 108.333

54.5 116.667

54.51 91.6667

54.52 141.667

54.53 91.6667

54.54 100

54.55 175

54.56 75

54.57 100

54.58 83.3333

54.59 58.3333

54.6 116.667

54.61 33.3333

54.62 58.3333

54.63 91.6667

54.64 100

54.65 133.333

54.66 141.667

54.67 100

54.68 58.3333

54.69 58.3333

54.7 58.3333

54.71 116.667

54.72 75

54.73 91.6667

54.74 75

54.75 91.6667

54.76 158.333

54.77 75

54.78 75

54.79 100

54.8 83.3333

54.81 125

54.82 91.6667

54.83 58.3333

54.84 66.6667

54.85 100

54.86 133.333

54.87 133.333

54.88 133.333

54.89 83.3333

54.9 100

54.91 75

54.92 150

54.93 58.3333

54.94 116.667

54.95 125

54.96 83.3333

54.97 158.333

54.98 83.3333

54.99 125

55 75

55.01 58.3333

55.02 100

55.03 108.333

55.04 66.6667

55.05 91.6667

55.06 58.3333

55.07 83.3333

55.08 91.6667

55.09 100

55.1 133.333

55.11 125

55.12 100

55.13 91.6667

55.14 75

55.15 141.667

55.16 75

55.17 158.333

55.18 75

55.19 75

55.2 100

55.21 116.667

55.22 125

55.23 66.6667

55.24 125

55.25 100

55.26 100

55.27 75

55.28 108.333

55.29 100

55.3 50

55.31 33.3333

55.32 91.6667

55.33 41.6667

55.34 208.333

55.35 116.667

55.36 108.333

55.37 100

55.38 100

55.39 75

55.4 100

55.41 66.6667

55.42 108.333

55.43 66.6667

55.44 116.667

55.45 50

55.46 83.3333

55.47 58.3333

55.48 91.6667

55.49 83.3333

55.5 133.333

55.51 91.6667

55.52 83.3333

55.53 75

55.54 83.3333

55.55 133.333

55.56 83.3333

55.57 75

55.58 191.667

55.59 100

55.6 58.3333

55.61 58.3333

55.62 91.6667

55.63 50

55.64 16.6667

55.65 125

55.66 116.667

55.67 125

55.68 133.333

55.69 108.333

55.7 100

55.71 125

55.72 75

55.73 108.333

55.74 66.6667

55.75 83.3333

55.76 100

55.77 108.333

55.78 83.3333

55.79 133.333

55.8 100

55.81 133.333

55.82 108.333

55.83 116.667

55.84 58.3333

55.85 75

55.86 116.667

55.87 100

55.88 108.333

55.89 83.3333

55.9 83.3333

55.91 100

55.92 50

55.93 91.6667

55.94 100

55.95 91.6667

55.96 116.667

55.97 75

55.98 116.667

55.99 66.6667

56 66.6667

56.01 75

56.02 83.3333

56.03 75

56.04 75

56.05 75

56.06 125

56.07 116.667

56.08 75

56.09 91.6667

56.1 91.6667

56.11 116.667

56.12 91.6667

56.13 91.6667

56.14 83.3333

56.15 133.333

56.16 66.6667

56.17 100

56.18 100

56.19 83.3333

56.2 133.333

56.21 83.3333

56.22 108.333

56.23 50

56.24 141.667

56.25 66.6667

56.26 125

56.27 133.333

56.28 91.6667

56.29 91.6667

56.3 116.667

56.31 141.667

56.32 66.6667

56.33 108.333

56.34 91.6667

56.35 83.3333

56.36 108.333

56.37 133.333

56.38 108.333

56.39 100

56.4 83.3333

56.41 83.3333

56.42 108.333

56.43 116.667

56.44 83.3333

56.45 83.3333

56.46 75

56.47 100

56.48 66.6667

56.49 125

56.5 83.3333

56.51 58.3333

56.52 116.667

56.53 91.6667

56.54 83.3333

56.55 75

56.56 108.333

56.57 175

56.58 91.6667

56.59 50

56.6 108.333

56.61 116.667

56.62 91.6667

56.63 58.3333

56.64 58.3333

56.65 58.3333

56.66 141.667

56.67 75

56.68 100

56.69 100

56.7 75

56.71 66.6667

56.72 100

56.73 75

56.74 116.667

56.75 75

56.76 83.3333

56.77 133.333

56.78 108.333

56.79 108.333

56.8 108.333

56.81 133.333

56.82 66.6667

56.83 50

56.84 91.6667

56.85 66.6667

56.86 133.333

56.87 83.3333

56.88 83.3333

56.89 108.333

56.9 50

56.91 133.333

56.92 91.6667

56.93 83.3333

56.94 91.6667

56.95 75

56.96 133.333

56.97 108.333

56.98 66.6667

56.99 108.333

57 108.333

57.01 108.333

57.02 58.3333

57.03 83.3333

57.04 100

57.05 58.3333

57.06 141.667

57.07 83.3333

57.08 91.6667

57.09 116.667

57.1 133.333

57.11 116.667

57.12 58.3333

57.13 100

57.14 66.6667

57.15 75

57.16 66.6667

57.17 66.6667

57.18 66.6667

57.19 75

57.2 75

57.21 133.333

57.22 100

57.23 125

57.24 75

57.25 108.333

57.26 116.667

57.27 100

57.28 158.333

57.29 66.6667

57.3 125

57.31 133.333

57.32 83.3333

57.33 166.667

57.34 116.667

57.35 75

57.36 100

57.37 125

57.38 83.3333

57.39 125

57.4 158.333

57.41 116.667

57.42 83.3333

57.43 116.667

57.44 133.333

57.45 66.6667

57.46 75

57.47 66.6667

57.48 116.667

57.49 133.333

57.5 141.667

57.51 108.333

57.52 91.6667

57.53 83.3333

57.54 50

57.55 75

57.56 158.333

57.57 125

57.58 91.6667

57.59 83.3333

57.6 100

57.61 125

57.62 108.333

57.63 75

57.64 58.3333

57.65 108.333

57.66 100

57.67 116.667

57.68 125

57.69 91.6667

57.7 75

57.71 91.6667

57.72 116.667

57.73 108.333

57.74 125

57.75 108.333

57.76 50

57.77 91.6667

57.78 58.3333

57.79 100

57.8 116.667

57.81 83.3333

57.82 125

57.83 100

57.84 100

57.85 75

57.86 116.667

57.87 91.6667

57.88 83.3333

57.89 108.333

57.9 150

57.91 66.6667

57.92 66.6667

57.93 91.6667

57.94 75

57.95 133.333

57.96 108.333

57.97 133.333

57.98 108.333

57.99 100

58 75

58.01 66.6667

58.02 100

58.03 100

58.04 100

58.05 108.333

58.06 91.6667

58.07 100

58.08 125

58.09 100

58.1 58.3333

58.11 116.667

58.12 91.6667

58.13 100

58.14 133.333

58.15 66.6667

58.16 108.333

58.17 141.667

58.18 91.6667

58.19 83.3333

58.2 150

58.21 116.667

58.22 158.333

58.23 116.667

58.24 116.667

58.25 66.6667

58.26 66.6667

58.27 125

58.28 83.3333

58.29 91.6667

58.3 91.6667

58.31 41.6667

58.32 108.333

58.33 91.6667

58.34 108.333

58.35 75

58.36 50

58.37 91.6667

58.38 100

58.39 125

58.4 75

58.41 58.3333

58.42 116.667

58.43 100

58.44 158.333

58.45 100

58.46 116.667

58.47 116.667

58.48 100

58.49 75

58.5 100

58.51 133.333

58.52 75

58.53 100

58.54 91.6667

58.55 100

58.56 33.3333

58.57 100

58.58 125

58.59 66.6667

58.6 108.333

58.61 100

58.62 116.667

58.63 75

58.64 66.6667

58.65 91.6667

58.66 66.6667

58.67 108.333

58.68 116.667

58.69 91.6667

58.7 116.667

58.71 83.3333

58.72 83.3333

58.73 83.3333

58.74 100

58.75 100

58.76 75

58.77 75

58.78 66.6667

58.79 58.3333

58.8 75

58.81 141.667

58.82 133.333

58.83 50

58.84 116.667

58.85 108.333

58.86 100

58.87 66.6667

58.88 91.6667

58.89 116.667

58.9 116.667

58.91 58.3333

58.92 41.6667

58.93 125

58.94 100

58.95 108.333

58.96 108.333

58.97 116.667

58.98 100

58.99 116.667

59 91.6667

59.01 158.333

59.02 166.667

59.03 125

59.04 83.3333

59.05 91.6667

59.06 83.3333

59.07 108.333

59.08 100

59.09 158.333

59.1 108.333

59.11 41.6667

59.12 100

59.13 108.333

59.14 116.667

59.15 66.6667

59.16 141.667

59.17 100

59.18 66.6667

59.19 133.333

59.2 108.333

59.21 100

59.22 100

59.23 108.333

59.24 91.6667

59.25 91.6667

59.26 133.333

59.27 83.3333

59.28 100

59.29 91.6667

59.3 125

59.31 83.3333

59.32 100

59.33 108.333

59.34 108.333

59.35 100

59.36 75

59.37 50

59.38 75

59.39 116.667

59.4 66.6667

59.41 83.3333

59.42 66.6667

59.43 58.3333

59.44 141.667

59.45 150

59.46 125

59.47 66.6667

59.48 100

59.49 116.667

59.5 91.6667

59.51 75

59.52 108.333

59.53 50

59.54 91.6667

59.55 175

59.56 100

59.57 100

59.58 75

59.59 100

59.6 108.333

59.61 100

59.62 125

59.63 50

59.64 91.6667

59.65 125

59.66 133.333

59.67 58.3333

59.68 166.667

59.69 83.3333

59.7 75

59.71 91.6667

59.72 141.667

59.73 108.333

59.74 116.667

59.75 75

59.76 75

59.77 66.6667

59.78 133.333

59.79 158.333

59.8 75

59.81 141.667

59.82 108.333

59.83 50

59.84 116.667

59.85 100

59.86 125

59.87 91.6667

59.88 83.3333

59.89 108.333

59.9 83.3333

59.91 83.3333

59.92 141.667

59.93 91.6667

59.94 91.6667

59.95 75

59.96 141.667

59.97 125

59.98 116.667

59.99 100

60 83.3333

60.01 116.667

60.02 91.6667

60.03 108.333

60.04 75

60.05 116.667

60.06 58.3333

60.07 50

60.08 150

60.09 58.3333

60.1 75

60.11 83.3333

60.12 50

60.13 133.333

60.14 125

60.15 166.667

60.16 66.6667

60.17 100

60.18 66.6667

60.19 66.6667

60.2 100

60.21 100

60.22 100

60.23 66.6667

60.24 100

60.25 108.333

60.26 150

60.27 125

60.28 125

60.29 100

60.3 108.333

60.31 150

60.32 100

60.33 75

60.34 66.6667

60.35 83.3333

60.36 108.333

60.37 33.3333

60.38 133.333

60.39 116.667

60.4 100

60.41 83.3333

60.42 108.333

60.43 141.667

60.44 100

60.45 100

60.46 75

60.47 58.3333

60.48 125

60.49 66.6667

60.5 66.6667

60.51 125

60.52 58.3333

60.53 75

60.54 150

60.55 100

60.56 100

60.57 108.333

60.58 75

60.59 100

60.6 141.667

60.61 91.6667

60.62 133.333

60.63 66.6667

60.64 133.333

60.65 58.3333

60.66 75

60.67 58.3333

60.68 66.6667

60.69 116.667

60.7 75

60.71 108.333

60.72 58.3333

60.73 158.333

60.74 116.667

60.75 100

60.76 58.3333

60.77 125

60.78 58.3333

60.79 108.333

60.8 83.3333

60.81 91.6667

60.82 108.333

60.83 91.6667

60.84 75

60.85 100

60.86 91.6667

60.87 83.3333

60.88 83.3333

60.89 125

60.9 166.667

60.91 66.6667

60.92 83.3333

60.93 133.333

60.94 166.667

60.95 133.333

60.96 58.3333

60.97 125

60.98 100

60.99 83.3333

61 50

61.01 166.667

61.02 66.6667

61.03 91.6667

61.04 125

61.05 41.6667

61.06 83.3333

61.07 83.3333

61.08 125

61.09 100

61.1 125

61.11 50

61.12 83.3333

61.13 83.3333

61.14 100

61.15 83.3333

61.16 75

61.17 125

61.18 125

61.19 75

61.2 100

61.21 141.667

61.22 116.667

61.23 150

61.24 75

61.25 66.6667

61.26 150

61.27 158.333

61.28 66.6667

61.29 158.333

61.3 108.333

61.31 75

61.32 116.667

61.33 100

61.34 108.333

61.35 100

61.36 141.667

61.37 66.6667

61.38 100

61.39 191.667

61.4 83.3333

61.41 83.3333

61.42 83.3333

61.43 100

61.44 75

61.45 83.3333

61.46 125

61.47 150

61.48 100

61.49 83.3333

61.5 100

61.51 108.333

61.52 100

61.53 133.333

61.54 175

61.55 125

61.56 141.667

61.57 125

61.58 133.333

61.59 83.3333

61.6 141.667

61.61 100

61.62 83.3333

61.63 83.3333

61.64 158.333

61.65 108.333

61.66 83.3333

61.67 125

61.68 91.6667

61.69 116.667

61.7 125

61.71 100

61.72 125

61.73 91.6667

61.74 116.667

61.75 75

61.76 116.667

61.77 91.6667

61.78 100

61.79 41.6667

61.8 116.667

61.81 150

61.82 125

61.83 116.667

61.84 125

61.85 108.333

61.86 66.6667

61.87 141.667

61.88 83.3333

61.89 133.333

61.9 100

61.91 91.6667

61.92 58.3333

61.93 100

61.94 183.333

61.95 158.333

61.96 116.667

61.97 100

61.98 133.333

61.99 150

62 100

62.01 150

62.02 150

62.03 125

62.04 100

62.05 125

62.06 125

62.07 108.333

62.08 125

62.09 50

62.1 83.3333

62.11 125

62.12 116.667

62.13 116.667

62.14 83.3333

62.15 66.6667

62.16 133.333

62.17 91.6667

62.18 100

62.19 133.333

62.2 100

62.21 91.6667

62.22 150

62.23 141.667

62.24 150

62.25 116.667

62.26 108.333

62.27 183.333

62.28 133.333

62.29 116.667

62.3 125

62.31 150

62.32 141.667

62.33 116.667

62.34 125

62.35 225

62.36 100

62.37 116.667

62.38 66.6667

62.39 108.333

62.4 125

62.41 125

62.42 108.333

62.43 141.667

62.44 100

62.45 108.333

62.46 200

62.47 116.667

62.48 83.3333

62.49 166.667

62.5 83.3333

62.51 91.6667

62.52 100

62.53 108.333

62.54 158.333

62.55 116.667

62.56 100

62.57 125

62.58 75

62.59 200

62.6 116.667

62.61 116.667

62.62 158.333

62.63 108.333

62.64 125

62.65 41.6667

62.66 125

62.67 150

62.68 75

62.69 133.333

62.7 158.333

62.71 125

62.72 125

62.73 166.667

62.74 141.667

62.75 125

62.76 91.6667

62.77 91.6667

62.78 91.6667

62.79 108.333

62.8 125

62.81 75

62.82 125

62.83 141.667

62.84 83.3333

62.85 116.667

62.86 133.333

62.87 116.667

62.88 83.3333

62.89 116.667

62.9 133.333

62.91 91.6667

62.92 91.6667

62.93 125

62.94 108.333

62.95 125

62.96 133.333

62.97 125

62.98 125

62.99 183.333

63 116.667

63.01 100

63.02 100

63.03 108.333

63.04 108.333

63.05 91.6667

63.06 75

63.07 158.333

63.08 91.6667

63.09 108.333

63.1 108.333

63.11 75

63.12 108.333

63.13 91.6667

63.14 66.6667

63.15 75

63.16 116.667

63.17 133.333

63.18 133.333

63.19 141.667

63.2 108.333

63.21 150

63.22 133.333

63.23 83.3333

63.24 125

63.25 100

63.26 141.667

63.27 150

63.28 108.333

63.29 133.333

63.3 183.333

63.31 116.667

63.32 116.667

63.33 183.333

63.34 125

63.35 183.333

63.36 125

63.37 116.667

63.38 133.333

63.39 166.667

63.4 133.333

63.41 100

63.42 100

63.43 108.333

63.44 100

63.45 116.667

63.46 75

63.47 125

63.48 75

63.49 58.3333

63.5 250

63.51 91.6667

63.52 108.333

63.53 83.3333

63.54 108.333

63.55 91.6667

63.56 108.333

63.57 75

63.58 66.6667

63.59 133.333

63.6 75

63.61 116.667

63.62 108.333

63.63 66.6667

63.64 83.3333

63.65 108.333

63.66 91.6667

63.67 125

63.68 133.333

63.69 108.333

63.7 100

63.71 83.3333

63.72 141.667

63.73 108.333

63.74 158.333

63.75 116.667

63.76 133.333

63.77 116.667

63.78 133.333

63.79 116.667

63.8 83.3333

63.81 50

63.82 141.667

63.83 83.3333

63.84 116.667

63.85 91.6667

63.86 108.333

63.87 91.6667

63.88 108.333

63.89 125

63.9 108.333

63.91 133.333

63.92 133.333

63.93 50

63.94 125

63.95 91.6667

63.96 108.333

63.97 108.333

63.98 116.667

63.99 116.667

64 100

64.01 75

64.02 91.6667

64.03 133.333

64.04 141.667

64.05 133.333

64.06 125

64.07 66.6667

64.08 83.3333

64.09 125

64.1 91.6667

64.11 75

64.12 91.6667

64.13 133.333

64.14 116.667

64.15 108.333

64.16 100

64.17 91.6667

64.18 133.333

64.19 141.667

64.2 75

64.21 91.6667

64.22 66.6667

64.23 133.333

64.24 116.667

64.25 108.333

64.26 108.333

64.27 175

64.28 83.3333

64.29 141.667

64.3 183.333

64.31 116.667

64.32 108.333

64.33 116.667

64.34 100

64.35 141.667

64.36 91.6667

64.37 108.333

64.38 50

64.39 50

64.4 116.667

64.41 75

64.42 83.3333

64.43 75

64.44 83.3333

64.45 100

64.46 66.6667

64.47 91.6667

64.48 50

64.49 75

64.5 150

64.51 133.333

64.52 191.667

64.53 125

64.54 100

64.55 83.3333

64.56 108.333

64.57 116.667

64.58 91.6667

64.59 150

64.6 116.667

64.61 66.6667

64.62 75

64.63 158.333

64.64 66.6667

64.65 100

64.66 108.333

64.67 133.333

64.68 83.3333

64.69 91.6667

64.7 175

64.71 116.667

64.72 108.333

64.73 125

64.74 108.333

64.75 58.3333

64.76 108.333

64.77 150

64.78 91.6667

64.79 91.6667

64.8 108.333

64.81 116.667

64.82 83.3333

64.83 33.3333

64.84 66.6667

64.85 75

64.86 66.6667

64.87 100

64.88 141.667

64.89 91.6667

64.9 150

64.91 150

64.92 108.333

64.93 116.667

64.94 150

64.95 141.667

64.96 108.333

64.97 141.667

64.98 158.333

64.99 116.667

65 183.333

65.01 141.667

65.02 125

65.03 100

65.04 158.333

65.05 125

65.06 133.333

65.07 208.333

65.08 108.333

65.09 150

65.1 116.667

65.11 150

65.12 41.6667

65.13 150

65.14 108.333

65.15 158.333

65.16 141.667

65.17 91.6667

65.18 108.333

65.19 91.6667

65.2 108.333

65.21 116.667

65.22 66.6667

65.23 66.6667

65.24 75

65.25 108.333

65.26 108.333

65.27 125

65.28 125

65.29 50

65.3 116.667

65.31 108.333

65.32 100

65.33 75

65.34 91.6667

65.35 91.6667

65.36 100

65.37 91.6667

65.38 133.333

65.39 58.3333

65.4 166.667

65.41 116.667

65.42 100

65.43 125

65.44 100

65.45 166.667

65.46 125

65.47 116.667

65.48 91.6667

65.49 75

65.5 83.3333

65.51 91.6667

65.52 83.3333

65.53 175

65.54 125

65.55 100

65.56 108.333

65.57 125

65.58 100

65.59 125

65.6 125

65.61 183.333

65.62 83.3333

65.63 100

65.64 66.6667

65.65 150

65.66 75

65.67 108.333

65.68 125

65.69 116.667

65.7 158.333

65.71 183.333

65.72 83.3333

65.73 50

65.74 116.667

65.75 75

65.76 66.6667

65.77 91.6667

65.78 108.333

65.79 83.3333

65.8 133.333

65.81 116.667

65.82 133.333

65.83 100

65.84 75

65.85 133.333

65.86 108.333

65.87 66.6667

65.88 108.333

65.89 91.6667

65.9 125

65.91 91.6667

65.92 83.3333

65.93 108.333

65.94 75

65.95 75

65.96 116.667

65.97 125

65.98 91.6667

65.99 108.333

66 133.333

66.01 91.6667

66.02 116.667

66.03 125

66.04 83.3333

66.05 83.3333

66.06 41.6667

66.07 83.3333

66.08 83.3333

66.09 83.3333

66.1 100

66.11 75

66.12 75

66.13 116.667

66.14 100

66.15 66.6667

66.16 100

66.17 100

66.18 41.6667

66.19 66.6667

66.2 108.333

66.21 100

66.22 66.6667

66.23 91.6667

66.24 125

66.25 100

66.26 100

66.27 125

66.28 91.6667

66.29 116.667

66.3 83.3333

66.31 83.3333

66.32 125

66.33 83.3333

66.34 91.6667

66.35 116.667

66.36 108.333

66.37 133.333

66.38 100

66.39 83.3333

66.4 91.6667

66.41 83.3333

66.42 75

66.43 108.333

66.44 116.667

66.45 108.333

66.46 116.667

66.47 91.6667

66.48 125

66.49 116.667

66.5 58.3333

66.51 50

66.52 100

66.53 91.6667

66.54 66.6667

66.55 75

66.56 116.667

66.57 91.6667

66.58 83.3333

66.59 75

66.6 175

66.61 91.6667

66.62 141.667

66.63 83.3333

66.64 83.3333

66.65 141.667

66.66 141.667

66.67 75

66.68 91.6667

66.69 83.3333

66.7 108.333

66.71 100

66.72 91.6667

66.73 58.3333

66.74 91.6667

66.75 83.3333

66.76 91.6667

66.77 83.3333

66.78 75

66.79 116.667

66.8 83.3333

66.81 83.3333

66.82 66.6667

66.83 100

66.84 116.667

66.85 108.333

66.86 75

66.87 91.6667

66.88 66.6667

66.89 83.3333

66.9 141.667

66.91 125

66.92 91.6667

66.93 100

66.94 91.6667

66.95 150

66.96 108.333

66.97 83.3333

66.98 158.333

66.99 66.6667

67 58.3333

67.01 66.6667

67.02 100

67.03 66.6667

67.04 133.333

67.05 83.3333

67.06 75

67.07 91.6667

67.08 83.3333

67.09 108.333

67.1 91.6667

67.11 125

67.12 125

67.13 75

67.14 41.6667

67.15 58.3333

67.16 100

67.17 83.3333

67.18 108.333

67.19 100

67.2 83.3333

67.21 91.6667

67.22 108.333

67.23 83.3333

67.24 58.3333

67.25 133.333

67.26 58.3333

67.27 75

67.28 66.6667

67.29 91.6667

67.3 75

67.31 108.333

67.32 66.6667

67.33 133.333

67.34 100

67.35 83.3333

67.36 91.6667

67.37 50

67.38 83.3333

67.39 108.333

67.4 91.6667

67.41 83.3333

67.42 83.3333

67.43 133.333

67.44 91.6667

67.45 91.6667

67.46 116.667

67.47 75

67.48 91.6667

67.49 75

67.5 116.667

67.51 75

67.52 66.6667

67.53 100

67.54 108.333

67.55 75

67.56 75

67.57 108.333

67.58 175

67.59 100

67.6 66.6667

67.61 125

67.62 75

67.63 133.333

67.64 141.667

67.65 58.3333

67.66 116.667

67.67 75

67.68 75

67.69 83.3333

67.7 66.6667

67.71 141.667

67.72 116.667

67.73 91.6667

67.74 100

67.75 66.6667

67.76 66.6667

67.77 125

67.78 91.6667

67.79 141.667

67.8 50

67.81 66.6667

67.82 100

67.83 83.3333

67.84 100

67.85 150

67.86 66.6667

67.87 108.333

67.88 100

67.89 108.333

67.9 100

67.91 58.3333

67.92 83.3333

67.93 58.3333

67.94 66.6667

67.95 75

67.96 83.3333

67.97 66.6667

67.98 75

67.99 83.3333

68 91.6667

68.01 75

68.02 83.3333

68.03 83.3333

68.04 91.6667

68.05 91.6667

68.06 133.333

68.07 58.3333

68.08 91.6667

68.09 100

68.1 75

68.11 91.6667

68.12 83.3333

68.13 100

68.14 141.667

68.15 91.6667

68.16 100

68.17 100

68.18 183.333

68.19 125

68.2 116.667

68.21 125

68.22 108.333

68.23 125

68.24 83.3333

68.25 58.3333

68.26 108.333

68.27 125

68.28 141.667

68.29 83.3333

68.3 66.6667

68.31 108.333

68.32 91.6667

68.33 66.6667

68.34 108.333

68.35 141.667

68.36 133.333

68.37 58.3333

68.38 108.333

68.39 33.3333

68.4 108.333

68.41 141.667

68.42 75

68.43 83.3333

68.44 116.667

68.45 116.667

68.46 100

68.47 58.3333

68.48 108.333

68.49 58.3333

68.5 100

68.51 91.6667

68.52 58.3333

68.53 133.333

68.54 83.3333

68.55 75

68.56 108.333

68.57 83.3333

68.58 83.3333

68.59 83.3333

68.6 91.6667

68.61 66.6667

68.62 75

68.63 108.333

68.64 100

68.65 83.3333

68.66 83.3333

68.67 66.6667

68.68 83.3333

68.69 66.6667

68.7 100

68.71 75

68.72 100

68.73 58.3333

68.74 91.6667

68.75 66.6667

68.76 75

68.77 116.667

68.78 141.667

68.79 83.3333

68.8 83.3333

68.81 50

68.82 91.6667

68.83 108.333

68.84 125

68.85 83.3333

68.86 141.667

68.87 83.3333

68.88 116.667

68.89 91.6667

68.9 133.333

68.91 158.333

68.92 116.667

68.93 75

68.94 133.333

68.95 91.6667

68.96 141.667

68.97 125

68.98 75

68.99 100

69 100

69.01 66.6667

69.02 58.3333

69.03 125

69.04 108.333

69.05 91.6667

69.06 83.3333

69.07 75

69.08 91.6667

69.09 58.3333

69.1 108.333

69.11 150

69.12 83.3333

69.13 91.6667

69.14 158.333

69.15 91.6667

69.16 108.333

69.17 66.6667

69.18 83.3333

69.19 66.6667

69.2 108.333

69.21 58.3333

69.22 75

69.23 83.3333

69.24 133.333

69.25 125

69.26 108.333

69.27 108.333

69.28 150

69.29 58.3333

69.3 108.333

69.31 75

69.32 100

69.33 100

69.34 91.6667

69.35 75

69.36 66.6667

69.37 75

69.38 83.3333

69.39 166.667

69.4 141.667

69.41 133.333

69.42 75

69.43 100

69.44 75

69.45 125

69.46 91.6667

69.47 116.667

69.48 66.6667

69.49 100

69.5 75

69.51 91.6667

69.52 100

69.53 83.3333

69.54 133.333

69.55 116.667

69.56 58.3333

69.57 108.333

69.58 91.6667

69.59 83.3333

69.6 141.667

69.61 75

69.62 75

69.63 58.3333

69.64 150

69.65 41.6667

69.66 100

69.67 83.3333

69.68 75

69.69 108.333

69.7 66.6667

69.71 75

69.72 150

69.73 108.333

69.74 141.667

69.75 108.333

69.76 100

69.77 83.3333

69.78 133.333

69.79 58.3333

69.8 75

69.81 83.3333

69.82 91.6667

69.83 66.6667

69.84 133.333

69.85 108.333

69.86 91.6667

69.87 133.333

69.88 91.6667

69.89 125

69.9 66.6667

69.91 75

69.92 91.6667

69.93 66.6667

69.94 91.6667

69.95 100

69.96 100

69.97 150

69.98 83.3333

69.99 75

70 75

**Raw data 5**. XRD raw data of the powder obtained after heat-treatment of as-sprayed powders prepared from the solution without carbon precursors at 500 ℃.

Goniometer RINT2000 vertical goniometer

Attachment Auto sample changer type B(6 samples)

Monochromater Fixed Monochromator

ScanningMode 2Theta/Theta

ScanningType Continuos Scanning

X-Ray 40kV/100mA

DivSlit 1 deg.

DivH.L.Slit 10mm

SctSlit 1 deg.

RecSlit 0.15mm

Monochro RS No Use

K-beta filter

Start 20

Stop 70

Step 0.01

20 543.333

20.01 523.333

20.02 593.333

20.03 473.333

20.04 506.667

20.05 643.333

20.06 586.667

20.07 516.667

20.08 593.333

20.09 516.667

20.1 543.333

20.11 626.667

20.12 633.333

20.13 496.667

20.14 573.333

20.15 506.667

20.16 643.333

20.17 556.667

20.18 600

20.19 546.667

20.2 563.333

20.21 570

20.22 613.333

20.23 536.667

20.24 620

20.25 600

20.26 633.333

20.27 666.667

20.28 606.667

20.29 626.667

20.3 590

20.31 636.667

20.32 546.667

20.33 480

20.34 533.333

20.35 580

20.36 536.667

20.37 573.333

20.38 493.333

20.39 533.333

20.4 616.667

20.41 576.667

20.42 550

20.43 586.667

20.44 543.333

20.45 546.667

20.46 533.333

20.47 643.333

20.48 526.667

20.49 593.333

20.5 570

20.51 553.333

20.52 536.667

20.53 613.333

20.54 473.333

20.55 593.333

20.56 570

20.57 506.667

20.58 523.333

20.59 560

20.6 610

20.61 586.667

20.62 560

20.63 563.333

20.64 546.667

20.65 590

20.66 573.333

20.67 486.667

20.68 626.667

20.69 593.333

20.7 520

20.71 510

20.72 623.333

20.73 576.667

20.74 560

20.75 613.333

20.76 586.667

20.77 543.333

20.78 573.333

20.79 640

20.8 606.667

20.81 593.333

20.82 563.333

20.83 543.333

20.84 633.333

20.85 573.333

20.86 586.667

20.87 543.333

20.88 533.333

20.89 563.333

20.9 573.333

20.91 586.667

20.92 546.667

20.93 596.667

20.94 490

20.95 563.333

20.96 520

20.97 610

20.98 633.333

20.99 636.667

21 633.333

21.01 583.333

21.02 663.333

21.03 590

21.04 576.667

21.05 600

21.06 630

21.07 616.667

21.08 560

21.09 613.333

21.1 526.667

21.11 480

21.12 480

21.13 606.667

21.14 630

21.15 533.333

21.16 550

21.17 533.333

21.18 520

21.19 590

21.2 623.333

21.21 573.333

21.22 610

21.23 506.667

21.24 593.333

21.25 643.333

21.26 643.333

21.27 623.333

21.28 593.333

21.29 606.667

21.3 570

21.31 613.333

21.32 613.333

21.33 536.667

21.34 523.333

21.35 580

21.36 646.667

21.37 580

21.38 510

21.39 593.333

21.4 636.667

21.41 573.333

21.42 500

21.43 586.667

21.44 540

21.45 583.333

21.46 526.667

21.47 563.333

21.48 530

21.49 593.333

21.5 580

21.51 606.667

21.52 556.667

21.53 566.667

21.54 606.667

21.55 630

21.56 540

21.57 533.333

21.58 583.333

21.59 603.333

21.6 546.667

21.61 580

21.62 656.667

21.63 550

21.64 570

21.65 503.333

21.66 550

21.67 586.667

21.68 596.667

21.69 590

21.7 566.667

21.71 573.333

21.72 593.333

21.73 626.667

21.74 583.333

21.75 543.333

21.76 506.667

21.77 546.667

21.78 573.333

21.79 603.333

21.8 593.333

21.81 640

21.82 556.667

21.83 636.667

21.84 526.667

21.85 523.333

21.86 560

21.87 636.667

21.88 566.667

21.89 600

21.9 510

21.91 620

21.92 626.667

21.93 603.333

21.94 543.333

21.95 560

21.96 623.333

21.97 536.667

21.98 606.667

21.99 563.333

22 583.333

22.01 596.667

22.02 550

22.03 613.333

22.04 573.333

22.05 560

22.06 616.667

22.07 620

22.08 476.667

22.09 563.333

22.1 550

22.11 576.667

22.12 510

22.13 606.667

22.14 563.333

22.15 526.667

22.16 583.333

22.17 600

22.18 500

22.19 466.667

22.2 573.333

22.21 576.667

22.22 603.333

22.23 623.333

22.24 473.333

22.25 570

22.26 620

22.27 616.667

22.28 583.333

22.29 630

22.3 580

22.31 616.667

22.32 623.333

22.33 583.333

22.34 513.333

22.35 470

22.36 563.333

22.37 650

22.38 543.333

22.39 480

22.4 470

22.41 523.333

22.42 566.667

22.43 513.333

22.44 540

22.45 496.667

22.46 640

22.47 516.667

22.48 503.333

22.49 533.333

22.5 533.333

22.51 576.667

22.52 500

22.53 540

22.54 603.333

22.55 503.333

22.56 563.333

22.57 523.333

22.58 556.667

22.59 556.667

22.6 590

22.61 450

22.62 526.667

22.63 556.667

22.64 580

22.65 570

22.66 583.333

22.67 513.333

22.68 560

22.69 516.667

22.7 536.667

22.71 466.667

22.72 503.333

22.73 480

22.74 510

22.75 536.667

22.76 480

22.77 523.333

22.78 533.333

22.79 413.333

22.8 513.333

22.81 516.667

22.82 563.333

22.83 476.667

22.84 580

22.85 490

22.86 510

22.87 523.333

22.88 490

22.89 516.667

22.9 440

22.91 480

22.92 500

22.93 540

22.94 556.667

22.95 536.667

22.96 490

22.97 510

22.98 463.333

22.99 463.333

23 466.667

23.01 586.667

23.02 443.333

23.03 390

23.04 543.333

23.05 536.667

23.06 496.667

23.07 463.333

23.08 443.333

23.09 480

23.1 516.667

23.11 483.333

23.12 490

23.13 470

23.14 433.333

23.15 460

23.16 450

23.17 410

23.18 513.333

23.19 473.333

23.2 493.333

23.21 426.667

23.22 480

23.23 426.667

23.24 543.333

23.25 450

23.26 510

23.27 466.667

23.28 500

23.29 436.667

23.3 513.333

23.31 483.333

23.32 483.333

23.33 480

23.34 456.667

23.35 456.667

23.36 433.333

23.37 500

23.38 466.667

23.39 526.667

23.4 456.667

23.41 560

23.42 473.333

23.43 423.333

23.44 503.333

23.45 493.333

23.46 486.667

23.47 490

23.48 500

23.49 446.667

23.5 466.667

23.51 400

23.52 460

23.53 446.667

23.54 436.667

23.55 403.333

23.56 530

23.57 450

23.58 433.333

23.59 426.667

23.6 436.667

23.61 513.333

23.62 420

23.63 413.333

23.64 490

23.65 500

23.66 506.667

23.67 436.667

23.68 473.333

23.69 413.333

23.7 413.333

23.71 420

23.72 410

23.73 463.333

23.74 463.333

23.75 513.333

23.76 400

23.77 363.333

23.78 443.333

23.79 426.667

23.8 473.333

23.81 500

23.82 440

23.83 476.667

23.84 446.667

23.85 446.667

23.86 443.333

23.87 436.667

23.88 403.333

23.89 430

23.9 426.667

23.91 430

23.92 403.333

23.93 486.667

23.94 386.667

23.95 430

23.96 416.667

23.97 353.333

23.98 366.667

23.99 433.333

24 500

24.01 473.333

24.02 423.333

24.03 483.333

24.04 413.333

24.05 396.667

24.06 403.333

24.07 380

24.08 426.667

24.09 450

24.1 453.333

24.11 370

24.12 380

24.13 403.333

24.14 363.333

24.15 413.333

24.16 390

24.17 473.333

24.18 416.667

24.19 456.667

24.2 316.667

24.21 433.333

24.22 423.333

24.23 436.667

24.24 373.333

24.25 423.333

24.26 406.667

24.27 410

24.28 440

24.29 380

24.3 390

24.31 430

24.32 373.333

24.33 396.667

24.34 333.333

24.35 373.333

24.36 486.667

24.37 390

24.38 436.667

24.39 360

24.4 416.667

24.41 386.667

24.42 393.333

24.43 360

24.44 410

24.45 356.667

24.46 380

24.47 466.667

24.48 346.667

24.49 380

24.5 393.333

24.51 426.667

24.52 366.667

24.53 376.667

24.54 463.333

24.55 340

24.56 333.333

24.57 346.667

24.58 390

24.59 390

24.6 356.667

24.61 300

24.62 380

24.63 470

24.64 386.667

24.65 423.333

24.66 350

24.67 423.333

24.68 343.333

24.69 330

24.7 396.667

24.71 266.667

24.72 373.333

24.73 366.667

24.74 343.333

24.75 373.333

24.76 310

24.77 410

24.78 373.333

24.79 320

24.8 390

24.81 383.333

24.82 403.333

24.83 406.667

24.84 400

24.85 386.667

24.86 320

24.87 393.333

24.88 306.667

24.89 336.667

24.9 356.667

24.91 393.333

24.92 390

24.93 393.333

24.94 376.667

24.95 393.333

24.96 320

24.97 390

24.98 270

24.99 313.333

25 310

25.01 330

25.02 370

25.03 350

25.04 363.333

25.05 303.333

25.06 400

25.07 356.667

25.08 336.667

25.09 370

25.1 370

25.11 380

25.12 323.333

25.13 300

25.14 316.667

25.15 313.333

25.16 346.667

25.17 333.333

25.18 340

25.19 340

25.2 326.667

25.21 273.333

25.22 390

25.23 306.667

25.24 343.333

25.25 336.667

25.26 396.667

25.27 350

25.28 350

25.29 276.667

25.3 306.667

25.31 343.333

25.32 376.667

25.33 306.667

25.34 313.333

25.35 343.333

25.36 316.667

25.37 340

25.38 283.333

25.39 350

25.4 280

25.41 283.333

25.42 366.667

25.43 316.667

25.44 310

25.45 320

25.46 336.667

25.47 343.333

25.48 316.667

25.49 346.667

25.5 293.333

25.51 343.333

25.52 346.667

25.53 273.333

25.54 343.333

25.55 303.333

25.56 276.667

25.57 390

25.58 253.333

25.59 336.667

25.6 403.333

25.61 313.333

25.62 316.667

25.63 353.333

25.64 313.333

25.65 296.667

25.66 290

25.67 343.333

25.68 306.667

25.69 266.667

25.7 353.333

25.71 293.333

25.72 296.667

25.73 256.667

25.74 303.333

25.75 310

25.76 316.667

25.77 336.667

25.78 286.667

25.79 263.333

25.8 330

25.81 273.333

25.82 293.333

25.83 240

25.84 273.333

25.85 316.667

25.86 346.667

25.87 300

25.88 320

25.89 320

25.9 313.333

25.91 280

25.92 310

25.93 253.333

25.94 320

25.95 296.667

25.96 350

25.97 313.333

25.98 286.667

25.99 360

26 333.333

26.01 350

26.02 296.667

26.03 293.333

26.04 263.333

26.05 283.333

26.06 286.667

26.07 263.333

26.08 366.667

26.09 340

26.1 270

26.11 330

26.12 360

26.13 286.667

26.14 326.667

26.15 326.667

26.16 216.667

26.17 253.333

26.18 296.667

26.19 266.667

26.2 290

26.21 280

26.22 290

26.23 290

26.24 306.667

26.25 326.667

26.26 300

26.27 236.667

26.28 230

26.29 286.667

26.3 283.333

26.31 266.667

26.32 250

26.33 276.667

26.34 310

26.35 296.667

26.36 286.667

26.37 203.333

26.38 250

26.39 276.667

26.4 263.333

26.41 276.667

26.42 270

26.43 266.667

26.44 323.333

26.45 273.333

26.46 286.667

26.47 216.667

26.48 216.667

26.49 276.667

26.5 233.333

26.51 263.333

26.52 260

26.53 283.333

26.54 316.667

26.55 296.667

26.56 253.333

26.57 266.667

26.58 273.333

26.59 310

26.6 256.667

26.61 220

26.62 240

26.63 300

26.64 253.333

26.65 243.333

26.66 270

26.67 253.333

26.68 250

26.69 253.333

26.7 313.333

26.71 206.667

26.72 313.333

26.73 270

26.74 243.333

26.75 276.667

26.76 256.667

26.77 210

26.78 266.667

26.79 286.667

26.8 266.667

26.81 330

26.82 220

26.83 206.667

26.84 283.333

26.85 330

26.86 233.333

26.87 226.667

26.88 203.333

26.89 256.667

26.9 250

26.91 256.667

26.92 253.333

26.93 286.667

26.94 243.333

26.95 226.667

26.96 223.333

26.97 290

26.98 270

26.99 173.333

27 286.667

27.01 283.333

27.02 276.667

27.03 256.667

27.04 263.333

27.05 223.333

27.06 243.333

27.07 326.667

27.08 216.667

27.09 230

27.1 213.333

27.11 226.667

27.12 246.667

27.13 296.667

27.14 236.667

27.15 260

27.16 243.333

27.17 226.667

27.18 293.333

27.19 256.667

27.2 246.667

27.21 276.667

27.22 230

27.23 233.333

27.24 206.667

27.25 203.333

27.26 230

27.27 260

27.28 250

27.29 230

27.3 213.333

27.31 240

27.32 230

27.33 246.667

27.34 226.667

27.35 220

27.36 210

27.37 193.333

27.38 310

27.39 216.667

27.4 250

27.41 223.333

27.42 253.333

27.43 256.667

27.44 283.333

27.45 246.667

27.46 233.333

27.47 280

27.48 226.667

27.49 250

27.5 233.333

27.51 206.667

27.52 270

27.53 230

27.54 240

27.55 216.667

27.56 253.333

27.57 236.667

27.58 220

27.59 253.333

27.6 170

27.61 216.667

27.62 253.333

27.63 206.667

27.64 200

27.65 250

27.66 233.333

27.67 236.667

27.68 233.333

27.69 196.667

27.7 200

27.71 203.333

27.72 190

27.73 256.667

27.74 200

27.75 256.667

27.76 236.667

27.77 206.667

27.78 253.333

27.79 203.333

27.8 220

27.81 213.333

27.82 210

27.83 213.333

27.84 253.333

27.85 220

27.86 243.333

27.87 236.667

27.88 193.333

27.89 210

27.9 193.333

27.91 203.333

27.92 166.667

27.93 203.333

27.94 240

27.95 233.333

27.96 200

27.97 196.667

27.98 226.667

27.99 223.333

28 253.333

28.01 253.333

28.02 236.667

28.03 240

28.04 216.667

28.05 243.333

28.06 190

28.07 253.333

28.08 256.667

28.09 220

28.1 196.667

28.11 193.333

28.12 233.333

28.13 243.333

28.14 206.667

28.15 246.667

28.16 243.333

28.17 250

28.18 256.667

28.19 246.667

28.2 246.667

28.21 246.667

28.22 206.667

28.23 200

28.24 236.667

28.25 206.667

28.26 203.333

28.27 213.333

28.28 206.667

28.29 170

28.3 226.667

28.31 176.667

28.32 246.667

28.33 270

28.34 213.333

28.35 233.333

28.36 210

28.37 220

28.38 206.667

28.39 213.333

28.4 213.333

28.41 163.333

28.42 186.667

28.43 210

28.44 203.333

28.45 213.333

28.46 210

28.47 183.333

28.48 236.667

28.49 193.333

28.5 193.333

28.51 200

28.52 266.667

28.53 166.667

28.54 200

28.55 183.333

28.56 156.667

28.57 226.667

28.58 210

28.59 170

28.6 213.333

28.61 213.333

28.62 200

28.63 176.667

28.64 203.333

28.65 246.667

28.66 190

28.67 180

28.68 160

28.69 180

28.7 223.333

28.71 220

28.72 183.333

28.73 230

28.74 206.667

28.75 236.667

28.76 180

28.77 186.667

28.78 196.667

28.79 163.333

28.8 213.333

28.81 166.667

28.82 146.667

28.83 173.333

28.84 210

28.85 180

28.86 210

28.87 200

28.88 193.333

28.89 203.333

28.9 220

28.91 193.333

28.92 196.667

28.93 216.667

28.94 170

28.95 170

28.96 176.667

28.97 216.667

28.98 180

28.99 166.667

29 200

29.01 203.333

29.02 150

29.03 230

29.04 190

29.05 163.333

29.06 183.333

29.07 203.333

29.08 233.333

29.09 153.333

29.1 186.667

29.11 170

29.12 170

29.13 193.333

29.14 193.333

29.15 193.333

29.16 133.333

29.17 186.667

29.18 170

29.19 216.667

29.2 186.667

29.21 183.333

29.22 163.333

29.23 193.333

29.24 240

29.25 236.667

29.26 193.333

29.27 153.333

29.28 213.333

29.29 160

29.3 173.333

29.31 146.667

29.32 180

29.33 196.667

29.34 186.667

29.35 216.667

29.36 236.667

29.37 163.333

29.38 213.333

29.39 210

29.4 190

29.41 193.333

29.42 173.333

29.43 160

29.44 233.333

29.45 170

29.46 193.333

29.47 140

29.48 166.667

29.49 206.667

29.5 213.333

29.51 216.667

29.52 193.333

29.53 180

29.54 196.667

29.55 166.667

29.56 226.667

29.57 153.333

29.58 193.333

29.59 210

29.6 166.667

29.61 216.667

29.62 173.333

29.63 186.667

29.64 173.333

29.65 150

29.66 193.333

29.67 160

29.68 193.333

29.69 196.667

29.7 153.333

29.71 183.333

29.72 183.333

29.73 193.333

29.74 166.667

29.75 173.333

29.76 170

29.77 133.333

29.78 196.667

29.79 196.667

29.8 170

29.81 160

29.82 156.667

29.83 180

29.84 183.333

29.85 173.333

29.86 176.667

29.87 176.667

29.88 176.667

29.89 156.667

29.9 186.667

29.91 146.667

29.92 116.667

29.93 193.333

29.94 166.667

29.95 183.333

29.96 166.667

29.97 180

29.98 180

29.99 176.667

30 186.667

30.01 166.667

30.02 200

30.03 170

30.04 180

30.05 210

30.06 183.333

30.07 130

30.08 190

30.09 200

30.1 193.333

30.11 133.333

30.12 156.667

30.13 186.667

30.14 146.667

30.15 186.667

30.16 173.333

30.17 203.333

30.18 160

30.19 206.667

30.2 180

30.21 170

30.22 153.333

30.23 146.667

30.24 166.667

30.25 140

30.26 183.333

30.27 186.667

30.28 153.333

30.29 146.667

30.3 200

30.31 163.333

30.32 156.667

30.33 133.333

30.34 160

30.35 123.333

30.36 140

30.37 160

30.38 180

30.39 156.667

30.4 150

30.41 156.667

30.42 173.333

30.43 130

30.44 140

30.45 166.667

30.46 173.333

30.47 146.667

30.48 190

30.49 146.667

30.5 176.667

30.51 156.667

30.52 160

30.53 190

30.54 180

30.55 166.667

30.56 146.667

30.57 106.667

30.58 193.333

30.59 160

30.6 230

30.61 173.333

30.62 160

30.63 163.333

30.64 150

30.65 153.333

30.66 170

30.67 153.333

30.68 160

30.69 190

30.7 100

30.71 146.667

30.72 143.333

30.73 173.333

30.74 136.667

30.75 153.333

30.76 200

30.77 156.667

30.78 193.333

30.79 170

30.8 160

30.81 166.667

30.82 156.667

30.83 160

30.84 183.333

30.85 123.333

30.86 193.333

30.87 173.333

30.88 163.333

30.89 173.333

30.9 176.667

30.91 140

30.92 156.667

30.93 176.667

30.94 163.333

30.95 163.333

30.96 180

30.97 173.333

30.98 180

30.99 226.667

31 173.333

31.01 163.333

31.02 140

31.03 153.333

31.04 136.667

31.05 170

31.06 126.667

31.07 186.667

31.08 150

31.09 183.333

31.1 180

31.11 146.667

31.12 140

31.13 143.333

31.14 156.667

31.15 136.667

31.16 136.667

31.17 143.333

31.18 163.333

31.19 150

31.2 153.333

31.21 160

31.22 136.667

31.23 126.667

31.24 160

31.25 163.333

31.26 153.333

31.27 163.333

31.28 160

31.29 156.667

31.3 133.333

31.31 153.333

31.32 160

31.33 150

31.34 173.333

31.35 156.667

31.36 166.667

31.37 150

31.38 170

31.39 143.333

31.4 130

31.41 143.333

31.42 173.333

31.43 173.333

31.44 163.333

31.45 136.667

31.46 143.333

31.47 146.667

31.48 123.333

31.49 133.333

31.5 176.667

31.51 146.667

31.52 146.667

31.53 173.333

31.54 136.667

31.55 166.667

31.56 163.333

31.57 183.333

31.58 153.333

31.59 150

31.6 176.667

31.61 126.667

31.62 133.333

31.63 150

31.64 113.333

31.65 196.667

31.66 173.333

31.67 150

31.68 170

31.69 156.667

31.7 170

31.71 170

31.72 130

31.73 166.667

31.74 136.667

31.75 156.667

31.76 170

31.77 176.667

31.78 156.667

31.79 153.333

31.8 123.333

31.81 136.667

31.82 140

31.83 146.667

31.84 166.667

31.85 120

31.86 140

31.87 173.333

31.88 156.667

31.89 123.333

31.9 140

31.91 190

31.92 143.333

31.93 140

31.94 143.333

31.95 166.667

31.96 150

31.97 143.333

31.98 170

31.99 163.333

32 126.667

32.01 166.667

32.02 156.667

32.03 130

32.04 123.333

32.05 130

32.06 146.667

32.07 130

32.08 93.3333

32.09 113.333

32.1 146.667

32.11 136.667

32.12 136.667

32.13 140

32.14 120

32.15 140

32.16 166.667

32.17 120

32.18 176.667

32.19 156.667

32.2 106.667

32.21 173.333

32.22 156.667

32.23 153.333

32.24 140

32.25 133.333

32.26 123.333

32.27 163.333

32.28 103.333

32.29 146.667

32.3 173.333

32.31 146.667

32.32 103.333

32.33 130

32.34 120

32.35 153.333

32.36 150

32.37 136.667

32.38 120

32.39 123.333

32.4 153.333

32.41 100

32.42 113.333

32.43 163.333

32.44 123.333

32.45 130

32.46 133.333

32.47 153.333

32.48 166.667

32.49 176.667

32.5 156.667

32.51 136.667

32.52 156.667

32.53 150

32.54 133.333

32.55 126.667

32.56 133.333

32.57 126.667

32.58 143.333

32.59 156.667

32.6 140

32.61 156.667

32.62 116.667

32.63 176.667

32.64 133.333

32.65 123.333

32.66 133.333

32.67 116.667

32.68 140

32.69 143.333

32.7 156.667

32.71 120

32.72 130

32.73 180

32.74 143.333

32.75 153.333

32.76 150

32.77 120

32.78 146.667

32.79 140

32.8 136.667

32.81 163.333

32.82 153.333

32.83 180

32.84 123.333

32.85 163.333

32.86 140

32.87 150

32.88 116.667

32.89 130

32.9 156.667

32.91 153.333

32.92 116.667

32.93 83.3333

32.94 133.333

32.95 130

32.96 150

32.97 136.667

32.98 160

32.99 126.667

33 143.333

33.01 113.333

33.02 143.333

33.03 146.667

33.04 116.667

33.05 133.333

33.06 130

33.07 160

33.08 140

33.09 136.667

33.1 143.333

33.11 96.6667

33.12 136.667

33.13 133.333

33.14 136.667

33.15 140

33.16 133.333

33.17 126.667

33.18 130

33.19 150

33.2 153.333

33.21 116.667

33.22 106.667

33.23 93.3333

33.24 133.333

33.25 106.667

33.26 130

33.27 133.333

33.28 180

33.29 186.667

33.3 133.333

33.31 120

33.32 143.333

33.33 130

33.34 126.667

33.35 133.333

33.36 136.667

33.37 156.667

33.38 133.333

33.39 123.333

33.4 133.333

33.41 113.333

33.42 170

33.43 96.6667

33.44 140

33.45 133.333

33.46 143.333

33.47 143.333

33.48 123.333

33.49 143.333

33.5 120

33.51 130

33.52 120

33.53 136.667

33.54 113.333

33.55 153.333

33.56 130

33.57 106.667

33.58 136.667

33.59 113.333

33.6 133.333

33.61 110

33.62 150

33.63 120

33.64 173.333

33.65 136.667

33.66 123.333

33.67 153.333

33.68 123.333

33.69 130

33.7 126.667

33.71 163.333

33.72 113.333

33.73 133.333

33.74 120

33.75 123.333

33.76 160

33.77 133.333

33.78 103.333

33.79 136.667

33.8 110

33.81 143.333

33.82 113.333

33.83 106.667

33.84 150

33.85 133.333

33.86 113.333

33.87 133.333

33.88 176.667

33.89 140

33.9 130

33.91 136.667

33.92 133.333

33.93 136.667

33.94 116.667

33.95 153.333

33.96 113.333

33.97 86.6667

33.98 100

33.99 136.667

34 113.333

34.01 123.333

34.02 100

34.03 120

34.04 133.333

34.05 130

34.06 140

34.07 153.333

34.08 113.333

34.09 120

34.1 136.667

34.11 126.667

34.12 130

34.13 123.333

34.14 130

34.15 113.333

34.16 116.667

34.17 113.333

34.18 153.333

34.19 126.667

34.2 156.667

34.21 153.333

34.22 120

34.23 113.333

34.24 116.667

34.25 120

34.26 120

34.27 146.667

34.28 133.333

34.29 170

34.3 130

34.31 110

34.32 130

34.33 113.333

34.34 100

34.35 113.333

34.36 106.667

34.37 136.667

34.38 156.667

34.39 113.333

34.4 113.333

34.41 86.6667

34.42 110

34.43 136.667

34.44 103.333

34.45 100

34.46 80

34.47 116.667

34.48 146.667

34.49 126.667

34.5 116.667

34.51 170

34.52 130

34.53 133.333

34.54 123.333

34.55 156.667

34.56 133.333

34.57 90

34.58 96.6667

34.59 150

34.6 113.333

34.61 116.667

34.62 156.667

34.63 133.333

34.64 110

34.65 110

34.66 110

34.67 70

34.68 120

34.69 146.667

34.7 83.3333

34.71 123.333

34.72 90

34.73 73.3333

34.74 153.333

34.75 136.667

34.76 133.333

34.77 133.333

34.78 143.333

34.79 113.333

34.8 126.667

34.81 66.6667

34.82 96.6667

34.83 156.667

34.84 146.667

34.85 123.333

34.86 100

34.87 106.667

34.88 140

34.89 90

34.9 163.333

34.91 123.333

34.92 153.333

34.93 93.3333

34.94 90

34.95 116.667

34.96 126.667

34.97 106.667

34.98 126.667

34.99 103.333

35 120

35.01 130

35.02 120

35.03 103.333

35.04 130

35.05 96.6667

35.06 113.333

35.07 110

35.08 123.333

35.09 123.333

35.1 103.333

35.11 133.333

35.12 123.333

35.13 110

35.14 130

35.15 140

35.16 160

35.17 190

35.18 133.333

35.19 100

35.2 136.667

35.21 176.667

35.22 116.667

35.23 136.667

35.24 120

35.25 116.667

35.26 123.333

35.27 123.333

35.28 100

35.29 140

35.3 106.667

35.31 126.667

35.32 140

35.33 83.3333

35.34 136.667

35.35 126.667

35.36 130

35.37 103.333

35.38 96.6667

35.39 123.333

35.4 106.667

35.41 123.333

35.42 90

35.43 93.3333

35.44 153.333

35.45 113.333

35.46 133.333

35.47 136.667

35.48 103.333

35.49 106.667

35.5 153.333

35.51 143.333

35.52 150

35.53 116.667

35.54 130

35.55 103.333

35.56 106.667

35.57 113.333

35.58 130

35.59 153.333

35.6 123.333

35.61 110

35.62 103.333

35.63 130

35.64 130

35.65 136.667

35.66 116.667

35.67 140

35.68 113.333

35.69 140

35.7 156.667

35.71 120

35.72 96.6667

35.73 123.333

35.74 123.333

35.75 140

35.76 110

35.77 96.6667

35.78 136.667

35.79 116.667

35.8 143.333

35.81 116.667

35.82 113.333

35.83 150

35.84 106.667

35.85 110

35.86 116.667

35.87 136.667

35.88 103.333

35.89 116.667

35.9 123.333

35.91 96.6667

35.92 123.333

35.93 100

35.94 110

35.95 100

35.96 90

35.97 93.3333

35.98 110

35.99 130

36 123.333

36.01 100

36.02 103.333

36.03 106.667

36.04 90

36.05 93.3333

36.06 143.333

36.07 96.6667

36.08 106.667

36.09 103.333

36.1 103.333

36.11 86.6667

36.12 90

36.13 106.667

36.14 110

36.15 96.6667

36.16 110

36.17 116.667

36.18 113.333

36.19 106.667

36.2 120

36.21 103.333

36.22 106.667

36.23 133.333

36.24 106.667

36.25 70

36.26 136.667

36.27 90

36.28 143.333

36.29 120

36.3 96.6667

36.31 110

36.32 83.3333

36.33 136.667

36.34 90

36.35 76.6667

36.36 96.6667

36.37 110

36.38 103.333

36.39 130

36.4 103.333

36.41 146.667

36.42 86.6667

36.43 100

36.44 143.333

36.45 123.333

36.46 103.333

36.47 106.667

36.48 93.3333

36.49 123.333

36.5 100

36.51 133.333

36.52 96.6667

36.53 110

36.54 120

36.55 86.6667

36.56 90

36.57 113.333

36.58 103.333

36.59 96.6667

36.6 100

36.61 103.333

36.62 96.6667

36.63 103.333

36.64 93.3333

36.65 120

36.66 73.3333

36.67 120

36.68 110

36.69 116.667

36.7 93.3333

36.71 96.6667

36.72 120

36.73 100

36.74 110

36.75 156.667

36.76 103.333

36.77 113.333

36.78 73.3333

36.79 126.667

36.8 93.3333

36.81 130

36.82 86.6667

36.83 116.667

36.84 103.333

36.85 63.3333

36.86 90

36.87 76.6667

36.88 103.333

36.89 110

36.9 120

36.91 76.6667

36.92 110

36.93 103.333

36.94 120

36.95 110

36.96 143.333

36.97 110

36.98 76.6667

36.99 110

37 113.333

37.01 83.3333

37.02 80

37.03 120

37.04 140

37.05 113.333

37.06 110

37.07 140

37.08 106.667

37.09 106.667

37.1 106.667

37.11 90

37.12 130

37.13 93.3333

37.14 103.333

37.15 70

37.16 86.6667

37.17 113.333

37.18 120

37.19 96.6667

37.2 90

37.21 83.3333

37.22 116.667

37.23 90

37.24 63.3333

37.25 93.3333

37.26 73.3333

37.27 106.667

37.28 100

37.29 100

37.3 100

37.31 90

37.32 103.333

37.33 83.3333

37.34 90

37.35 90

37.36 116.667

37.37 103.333

37.38 110

37.39 90

37.4 163.333

37.41 113.333

37.42 83.3333

37.43 113.333

37.44 113.333

37.45 120

37.46 83.3333

37.47 110

37.48 120

37.49 80

37.5 100

37.51 100

37.52 83.3333

37.53 110

37.54 76.6667

37.55 100

37.56 103.333

37.57 93.3333

37.58 133.333

37.59 93.3333

37.6 86.6667

37.61 113.333

37.62 120

37.63 80

37.64 116.667

37.65 93.3333

37.66 113.333

37.67 96.6667

37.68 126.667

37.69 100

37.7 90

37.71 106.667

37.72 93.3333

37.73 86.6667

37.74 103.333

37.75 70

37.76 100

37.77 100

37.78 70

37.79 93.3333

37.8 106.667

37.81 93.3333

37.82 96.6667

37.83 113.333

37.84 120

37.85 110

37.86 80

37.87 106.667

37.88 93.3333

37.89 136.667

37.9 100

37.91 70

37.92 90

37.93 93.3333

37.94 110

37.95 123.333

37.96 126.667

37.97 83.3333

37.98 103.333

37.99 113.333

38 76.6667

38.01 93.3333

38.02 100

38.03 96.6667

38.04 140

38.05 110

38.06 90

38.07 93.3333

38.08 126.667

38.09 80

38.1 113.333

38.11 110

38.12 120

38.13 110

38.14 93.3333

38.15 66.6667

38.16 120

38.17 106.667

38.18 140

38.19 110

38.2 80

38.21 96.6667

38.22 110

38.23 60

38.24 80

38.25 76.6667

38.26 90

38.27 70

38.28 86.6667

38.29 93.3333

38.3 126.667

38.31 90

38.32 110

38.33 90

38.34 86.6667

38.35 90

38.36 90

38.37 83.3333

38.38 113.333

38.39 76.6667

38.4 93.3333

38.41 73.3333

38.42 86.6667

38.43 113.333

38.44 73.3333

38.45 80

38.46 116.667

38.47 83.3333

38.48 100

38.49 140

38.5 90

38.51 76.6667

38.52 86.6667

38.53 106.667

38.54 86.6667

38.55 63.3333

38.56 86.6667

38.57 103.333

38.58 66.6667

38.59 63.3333

38.6 70

38.61 60

38.62 100

38.63 60

38.64 126.667

38.65 116.667

38.66 110

38.67 50

38.68 83.3333

38.69 73.3333

38.7 106.667

38.71 106.667

38.72 83.3333

38.73 96.6667

38.74 96.6667

38.75 90

38.76 80

38.77 76.6667

38.78 66.6667

38.79 120

38.8 106.667

38.81 86.6667

38.82 93.3333

38.83 90

38.84 66.6667

38.85 76.6667

38.86 86.6667

38.87 66.6667

38.88 83.3333

38.89 113.333

38.9 106.667

38.91 73.3333

38.92 100

38.93 66.6667

38.94 93.3333

38.95 90

38.96 83.3333

38.97 93.3333

38.98 83.3333

38.99 90

39 86.6667

39.01 96.6667

39.02 93.3333

39.03 106.667

39.04 96.6667

39.05 140

39.06 83.3333

39.07 86.6667

39.08 96.6667

39.09 83.3333

39.1 53.3333

39.11 103.333

39.12 70

39.13 106.667

39.14 103.333

39.15 86.6667

39.16 83.3333

39.17 73.3333

39.18 63.3333

39.19 73.3333

39.2 86.6667

39.21 96.6667

39.22 93.3333

39.23 86.6667

39.24 96.6667

39.25 90

39.26 93.3333

39.27 100

39.28 106.667

39.29 83.3333

39.3 90

39.31 56.6667

39.32 73.3333

39.33 90

39.34 100

39.35 103.333

39.36 83.3333

39.37 80

39.38 93.3333

39.39 73.3333

39.4 70

39.41 70

39.42 90

39.43 60

39.44 93.3333

39.45 86.6667

39.46 66.6667

39.47 90

39.48 100

39.49 60

39.5 80

39.51 80

39.52 83.3333

39.53 73.3333

39.54 53.3333

39.55 73.3333

39.56 96.6667

39.57 56.6667

39.58 80

39.59 80

39.6 73.3333

39.61 66.6667

39.62 60

39.63 66.6667

39.64 40

39.65 110

39.66 93.3333

39.67 63.3333

39.68 86.6667

39.69 96.6667

39.7 93.3333

39.71 60

39.72 76.6667

39.73 73.3333

39.74 73.3333

39.75 73.3333

39.76 83.3333

39.77 103.333

39.78 70

39.79 46.6667

39.8 86.6667

39.81 60

39.82 83.3333

39.83 66.6667

39.84 80

39.85 96.6667

39.86 116.667

39.87 73.3333

39.88 83.3333

39.89 60

39.9 103.333

39.91 73.3333

39.92 106.667

39.93 60

39.94 50

39.95 110

39.96 90

39.97 93.3333

39.98 63.3333

39.99 83.3333

40 83.3333

40.01 96.6667

40.02 86.6667

40.03 50

40.04 93.3333

40.05 70

40.06 80

40.07 46.6667

40.08 73.3333

40.09 120

40.1 90

40.11 103.333

40.12 73.3333

40.13 73.3333

40.14 53.3333

40.15 83.3333

40.16 63.3333

40.17 90

40.18 93.3333

40.19 46.6667

40.2 93.3333

40.21 73.3333

40.22 86.6667

40.23 73.3333

40.24 73.3333

40.25 70

40.26 110

40.27 106.667

40.28 60

40.29 73.3333

40.3 96.6667

40.31 70

40.32 63.3333

40.33 53.3333

40.34 83.3333

40.35 100

40.36 86.6667

40.37 103.333

40.38 83.3333

40.39 100

40.4 66.6667

40.41 63.3333

40.42 80

40.43 70

40.44 80

40.45 66.6667

40.46 66.6667

40.47 50

40.48 66.6667

40.49 93.3333

40.5 70

40.51 76.6667

40.52 60

40.53 93.3333

40.54 73.3333

40.55 73.3333

40.56 100

40.57 96.6667

40.58 93.3333

40.59 66.6667

40.6 66.6667

40.61 86.6667

40.62 90

40.63 80

40.64 80

40.65 66.6667

40.66 100

40.67 80

40.68 70

40.69 96.6667

40.7 93.3333

40.71 80

40.72 70

40.73 66.6667

40.74 80

40.75 100

40.76 70

40.77 90

40.78 80

40.79 116.667

40.8 76.6667

40.81 66.6667

40.82 96.6667

40.83 66.6667

40.84 96.6667

40.85 96.6667

40.86 70

40.87 80

40.88 50

40.89 73.3333

40.9 63.3333

40.91 60

40.92 83.3333

40.93 73.3333

40.94 70

40.95 70

40.96 80

40.97 80

40.98 70

40.99 116.667

41 90

41.01 90

41.02 46.6667

41.03 100

41.04 106.667

41.05 60

41.06 63.3333

41.07 90

41.08 76.6667

41.09 96.6667

41.1 90

41.11 53.3333

41.12 60

41.13 106.667

41.14 76.6667

41.15 73.3333

41.16 66.6667

41.17 60

41.18 66.6667

41.19 86.6667

41.2 50

41.21 86.6667

41.22 46.6667

41.23 90

41.24 93.3333

41.25 96.6667

41.26 83.3333

41.27 73.3333

41.28 83.3333

41.29 80

41.3 70

41.31 73.3333

41.32 76.6667

41.33 90

41.34 73.3333

41.35 93.3333

41.36 63.3333

41.37 53.3333

41.38 86.6667

41.39 40

41.4 80

41.41 103.333

41.42 100

41.43 53.3333

41.44 80

41.45 80

41.46 106.667

41.47 63.3333

41.48 53.3333

41.49 96.6667

41.5 70

41.51 86.6667

41.52 76.6667

41.53 80

41.54 60

41.55 80

41.56 76.6667

41.57 66.6667

41.58 76.6667

41.59 86.6667

41.6 66.6667

41.61 76.6667

41.62 66.6667

41.63 93.3333

41.64 80

41.65 60

41.66 90

41.67 110

41.68 80

41.69 53.3333

41.7 86.6667

41.71 83.3333

41.72 93.3333

41.73 93.3333

41.74 80

41.75 100

41.76 83.3333

41.77 70

41.78 100

41.79 86.6667

41.8 83.3333

41.81 76.6667

41.82 80

41.83 66.6667

41.84 36.6667

41.85 70

41.86 66.6667

41.87 66.6667

41.88 80

41.89 73.3333

41.9 73.3333

41.91 80

41.92 113.333

41.93 60

41.94 73.3333

41.95 70

41.96 86.6667

41.97 73.3333

41.98 70

41.99 30

42 96.6667

42.01 116.667

42.02 50

42.03 76.6667

42.04 60

42.05 106.667

42.06 60

42.07 66.6667

42.08 103.333

42.09 46.6667

42.1 73.3333

42.11 80

42.12 60

42.13 103.333

42.14 76.6667

42.15 80

42.16 90

42.17 66.6667

42.18 56.6667

42.19 60

42.2 73.3333

42.21 100

42.22 80

42.23 133.333

42.24 66.6667

42.25 66.6667

42.26 80

42.27 66.6667

42.28 70

42.29 76.6667

42.3 60

42.31 100

42.32 56.6667

42.33 90

42.34 46.6667

42.35 63.3333

42.36 80

42.37 83.3333

42.38 80

42.39 83.3333

42.4 43.3333

42.41 60

42.42 120

42.43 83.3333

42.44 116.667

42.45 93.3333

42.46 106.667

42.47 80

42.48 50

42.49 66.6667

42.5 100

42.51 103.333

42.52 63.3333

42.53 50

42.54 113.333

42.55 83.3333

42.56 83.3333

42.57 76.6667

42.58 63.3333

42.59 90

42.6 56.6667

42.61 86.6667

42.62 80

42.63 66.6667

42.64 80

42.65 93.3333

42.66 70

42.67 63.3333

42.68 96.6667

42.69 76.6667

42.7 70

42.71 90

42.72 53.3333

42.73 73.3333

42.74 93.3333

42.75 83.3333

42.76 80

42.77 76.6667

42.78 50

42.79 100

42.8 76.6667

42.81 76.6667

42.82 53.3333

42.83 83.3333

42.84 73.3333

42.85 83.3333

42.86 96.6667

42.87 63.3333

42.88 66.6667

42.89 63.3333

42.9 83.3333

42.91 93.3333

42.92 103.333

42.93 83.3333

42.94 83.3333

42.95 70

42.96 100

42.97 66.6667

42.98 113.333

42.99 83.3333

43 123.333

43.01 96.6667

43.02 53.3333

43.03 53.3333

43.04 86.6667

43.05 80

43.06 63.3333

43.07 83.3333

43.08 83.3333

43.09 76.6667

43.1 70

43.11 100

43.12 86.6667

43.13 116.667

43.14 86.6667

43.15 93.3333

43.16 80

43.17 60

43.18 73.3333

43.19 50

43.2 93.3333

43.21 70

43.22 80

43.23 116.667

43.24 63.3333

43.25 66.6667

43.26 73.3333

43.27 90

43.28 83.3333

43.29 90

43.3 83.3333

43.31 93.3333

43.32 103.333

43.33 83.3333

43.34 110

43.35 76.6667

43.36 80

43.37 106.667

43.38 73.3333

43.39 73.3333

43.4 100

43.41 56.6667

43.42 90

43.43 100

43.44 73.3333

43.45 90

43.46 120

43.47 103.333

43.48 100

43.49 93.3333

43.5 83.3333

43.51 116.667

43.52 93.3333

43.53 93.3333

43.54 76.6667

43.55 110

43.56 73.3333

43.57 90

43.58 70

43.59 103.333

43.6 63.3333

43.61 60

43.62 96.6667

43.63 100

43.64 76.6667

43.65 86.6667

43.66 76.6667

43.67 116.667

43.68 106.667

43.69 86.6667

43.7 70

43.71 90

43.72 73.3333

43.73 83.3333

43.74 80

43.75 80

43.76 113.333

43.77 100

43.78 86.6667

43.79 93.3333

43.8 103.333

43.81 80

43.82 80

43.83 90

43.84 90

43.85 100

43.86 90

43.87 123.333

43.88 83.3333

43.89 90

43.9 73.3333

43.91 123.333

43.92 76.6667

43.93 90

43.94 110

43.95 126.667

43.96 73.3333

43.97 83.3333

43.98 100

43.99 76.6667

44 80

44.01 60

44.02 96.6667

44.03 90

44.04 113.333

44.05 113.333

44.06 133.333

44.07 116.667

44.08 106.667

44.09 90

44.1 76.6667

44.11 103.333

44.12 93.3333

44.13 116.667

44.14 90

44.15 130

44.16 136.667

44.17 93.3333

44.18 116.667

44.19 60

44.2 106.667

44.21 70

44.22 66.6667

44.23 93.3333

44.24 86.6667

44.25 120

44.26 90

44.27 123.333

44.28 103.333

44.29 126.667

44.3 76.6667

44.31 80

44.32 103.333

44.33 126.667

44.34 143.333

44.35 126.667

44.36 90

44.37 123.333

44.38 116.667

44.39 100

44.4 103.333

44.41 160

44.42 143.333

44.43 146.667

44.44 170

44.45 103.333

44.46 140

44.47 126.667

44.48 126.667

44.49 113.333

44.5 143.333

44.51 156.667

44.52 180

44.53 203.333

44.54 176.667

44.55 230

44.56 210

44.57 256.667

44.58 260

44.59 333.333

44.6 266.667

44.61 346.667

44.62 536.667

44.63 593.333

44.64 793.333

44.65 996.667

44.66 1366.67

44.67 1560

44.68 2103.33

44.69 2250

44.7 2740

44.71 2516.67

44.72 2550

44.73 2310

44.74 1876.67

44.75 1603.33

44.76 1286.67

44.77 1233.33

44.78 1076.67

44.79 1026.67

44.8 1223.33

44.81 1090

44.82 1216.67

44.83 1070

44.84 1063.33

44.85 893.333

44.86 740

44.87 640

44.88 463.333

44.89 366.667

44.9 283.333

44.91 193.333

44.92 173.333

44.93 173.333

44.94 143.333

44.95 193.333

44.96 136.667

44.97 133.333

44.98 140

44.99 130

45 133.333

45.01 133.333

45.02 130

45.03 80

45.04 93.3333

45.05 100

45.06 76.6667

45.07 103.333

45.08 113.333

45.09 73.3333

45.1 56.6667

45.11 110

45.12 96.6667

45.13 103.333

45.14 90

45.15 73.3333

45.16 73.3333

45.17 120

45.18 100

45.19 113.333

45.2 96.6667

45.21 90

45.22 90

45.23 103.333

45.24 83.3333

45.25 56.6667

45.26 106.667

45.27 83.3333

45.28 70

45.29 100

45.3 83.3333

45.31 73.3333

45.32 73.3333

45.33 100

45.34 70

45.35 80

45.36 83.3333

45.37 93.3333

45.38 93.3333

45.39 80

45.4 73.3333

45.41 66.6667

45.42 93.3333

45.43 66.6667

45.44 86.6667

45.45 86.6667

45.46 76.6667

45.47 76.6667

45.48 43.3333

45.49 66.6667

45.5 83.3333

45.51 100

45.52 56.6667

45.53 43.3333

45.54 86.6667

45.55 70

45.56 90

45.57 46.6667

45.58 53.3333

45.59 73.3333

45.6 66.6667

45.61 76.6667

45.62 86.6667

45.63 90

45.64 63.3333

45.65 70

45.66 80

45.67 96.6667

45.68 66.6667

45.69 63.3333

45.7 100

45.71 53.3333

45.72 63.3333

45.73 60

45.74 80

45.75 93.3333

45.76 50

45.77 113.333

45.78 56.6667

45.79 100

45.8 66.6667

45.81 73.3333

45.82 83.3333

45.83 73.3333

45.84 80

45.85 66.6667

45.86 63.3333

45.87 120

45.88 53.3333

45.89 80

45.9 80

45.91 113.333

45.92 83.3333

45.93 73.3333

45.94 76.6667

45.95 110

45.96 83.3333

45.97 103.333

45.98 83.3333

45.99 83.3333

46 100

46.01 100

46.02 83.3333

46.03 63.3333

46.04 80

46.05 86.6667

46.06 86.6667

46.07 73.3333

46.08 70

46.09 86.6667

46.1 66.6667

46.11 93.3333

46.12 70

46.13 93.3333

46.14 80

46.15 70

46.16 83.3333

46.17 93.3333

46.18 63.3333

46.19 70

46.2 50

46.21 123.333

46.22 100

46.23 70

46.24 60

46.25 43.3333

46.26 53.3333

46.27 76.6667

46.28 90

46.29 73.3333

46.3 66.6667

46.31 53.3333

46.32 66.6667

46.33 60

46.34 76.6667

46.35 70

46.36 70

46.37 76.6667

46.38 76.6667

46.39 103.333

46.4 66.6667

46.41 83.3333

46.42 86.6667

46.43 70

46.44 66.6667

46.45 66.6667

46.46 73.3333

46.47 76.6667

46.48 80

46.49 63.3333

46.5 103.333

46.51 76.6667

46.52 83.3333

46.53 86.6667

46.54 120

46.55 106.667

46.56 66.6667

46.57 103.333

46.58 73.3333

46.59 70

46.6 73.3333

46.61 63.3333

46.62 53.3333

46.63 80

46.64 80

46.65 76.6667

46.66 86.6667

46.67 66.6667

46.68 103.333

46.69 110

46.7 76.6667

46.71 83.3333

46.72 66.6667

46.73 76.6667

46.74 60

46.75 100

46.76 90

46.77 73.3333

46.78 100

46.79 63.3333

46.8 53.3333

46.81 76.6667

46.82 80

46.83 80

46.84 90

46.85 80

46.86 76.6667

46.87 63.3333

46.88 66.6667

46.89 70

46.9 103.333

46.91 86.6667

46.92 53.3333

46.93 103.333

46.94 90

46.95 90

46.96 93.3333

46.97 60

46.98 76.6667

46.99 70

47 53.3333

47.01 86.6667

47.02 76.6667

47.03 70

47.04 66.6667

47.05 90

47.06 80

47.07 83.3333

47.08 86.6667

47.09 40

47.1 93.3333

47.11 50

47.12 76.6667

47.13 56.6667

47.14 86.6667

47.15 56.6667

47.16 83.3333

47.17 66.6667

47.18 90

47.19 100

47.2 76.6667

47.21 86.6667

47.22 83.3333

47.23 93.3333

47.24 96.6667

47.25 76.6667

47.26 80

47.27 60

47.28 90

47.29 83.3333

47.3 60

47.31 96.6667

47.32 80

47.33 86.6667

47.34 80

47.35 80

47.36 90

47.37 90

47.38 70

47.39 76.6667

47.4 73.3333

47.41 93.3333

47.42 93.3333

47.43 73.3333

47.44 66.6667

47.45 63.3333

47.46 83.3333

47.47 60

47.48 86.6667

47.49 70

47.5 86.6667

47.51 63.3333

47.52 63.3333

47.53 73.3333

47.54 90

47.55 93.3333

47.56 83.3333

47.57 83.3333

47.58 70

47.59 43.3333

47.6 56.6667

47.61 80

47.62 73.3333

47.63 73.3333

47.64 90

47.65 120

47.66 106.667

47.67 66.6667

47.68 63.3333

47.69 56.6667

47.7 73.3333

47.71 70

47.72 83.3333

47.73 70

47.74 70

47.75 113.333

47.76 96.6667

47.77 90

47.78 60

47.79 66.6667

47.8 76.6667

47.81 80

47.82 76.6667

47.83 60

47.84 46.6667

47.85 86.6667

47.86 76.6667

47.87 86.6667

47.88 60

47.89 50

47.9 93.3333

47.91 46.6667

47.92 56.6667

47.93 63.3333

47.94 90

47.95 60

47.96 83.3333

47.97 113.333

47.98 66.6667

47.99 76.6667

48 66.6667

48.01 50

48.02 70

48.03 46.6667

48.04 93.3333

48.05 80

48.06 83.3333

48.07 63.3333

48.08 60

48.09 43.3333

48.1 56.6667

48.11 83.3333

48.12 46.6667

48.13 83.3333

48.14 70

48.15 83.3333

48.16 80

48.17 70

48.18 80

48.19 63.3333

48.2 90

48.21 93.3333

48.22 73.3333

48.23 110

48.24 76.6667

48.25 50

48.26 63.3333

48.27 90

48.28 86.6667

48.29 103.333

48.3 63.3333

48.31 90

48.32 76.6667

48.33 50

48.34 60

48.35 83.3333

48.36 70

48.37 93.3333

48.38 70

48.39 80

48.4 76.6667

48.41 76.6667

48.42 66.6667

48.43 70

48.44 80

48.45 53.3333

48.46 60

48.47 60

48.48 73.3333

48.49 93.3333

48.5 80

48.51 70

48.52 63.3333

48.53 66.6667

48.54 80

48.55 90

48.56 73.3333

48.57 80

48.58 90

48.59 83.3333

48.6 90

48.61 86.6667

48.62 76.6667

48.63 93.3333

48.64 83.3333

48.65 63.3333

48.66 80

48.67 120

48.68 86.6667

48.69 70

48.7 86.6667

48.71 53.3333

48.72 80

48.73 56.6667

48.74 73.3333

48.75 80

48.76 73.3333

48.77 80

48.78 113.333

48.79 86.6667

48.8 100

48.81 70

48.82 86.6667

48.83 50

48.84 93.3333

48.85 83.3333

48.86 76.6667

48.87 90

48.88 50

48.89 80

48.9 93.3333

48.91 70

48.92 70

48.93 50

48.94 86.6667

48.95 70

48.96 80

48.97 76.6667

48.98 80

48.99 76.6667

49 63.3333

49.01 50

49.02 60

49.03 73.3333

49.04 53.3333

49.05 76.6667

49.06 50

49.07 40

49.08 66.6667

49.09 80

49.1 90

49.11 66.6667

49.12 86.6667

49.13 40

49.14 70

49.15 76.6667

49.16 63.3333

49.17 86.6667

49.18 60

49.19 90

49.2 83.3333

49.21 53.3333

49.22 63.3333

49.23 80

49.24 46.6667

49.25 106.667

49.26 73.3333

49.27 80

49.28 80

49.29 80

49.3 66.6667

49.31 46.6667

49.32 93.3333

49.33 66.6667

49.34 86.6667

49.35 73.3333

49.36 73.3333

49.37 83.3333

49.38 83.3333

49.39 76.6667

49.4 60

49.41 70

49.42 53.3333

49.43 86.6667

49.44 80

49.45 66.6667

49.46 73.3333

49.47 83.3333

49.48 40

49.49 93.3333

49.5 50

49.51 53.3333

49.52 56.6667

49.53 80

49.54 93.3333

49.55 76.6667

49.56 70

49.57 66.6667

49.58 60

49.59 70

49.6 73.3333

49.61 106.667

49.62 133.333

49.63 73.3333

49.64 83.3333

49.65 86.6667

49.66 73.3333

49.67 76.6667

49.68 50

49.69 53.3333

49.7 83.3333

49.71 43.3333

49.72 53.3333

49.73 93.3333

49.74 76.6667

49.75 80

49.76 66.6667

49.77 53.3333

49.78 103.333

49.79 83.3333

49.8 50

49.81 83.3333

49.82 93.3333

49.83 50

49.84 86.6667

49.85 70

49.86 70

49.87 56.6667

49.88 76.6667

49.89 66.6667

49.9 70

49.91 76.6667

49.92 76.6667

49.93 70

49.94 86.6667

49.95 73.3333

49.96 60

49.97 46.6667

49.98 73.3333

49.99 53.3333

50 90

50.01 80

50.02 73.3333

50.03 76.6667

50.04 63.3333

50.05 50

50.06 53.3333

50.07 70

50.08 60

50.09 50

50.1 63.3333

50.11 93.3333

50.12 80

50.13 63.3333

50.14 66.6667

50.15 83.3333

50.16 63.3333

50.17 93.3333

50.18 83.3333

50.19 36.6667

50.2 86.6667

50.21 66.6667

50.22 86.6667

50.23 83.3333

50.24 73.3333

50.25 46.6667

50.26 86.6667

50.27 70

50.28 90

50.29 40

50.3 63.3333

50.31 90

50.32 40

50.33 73.3333

50.34 60

50.35 83.3333

50.36 80

50.37 83.3333

50.38 83.3333

50.39 83.3333

50.4 66.6667

50.41 63.3333

50.42 70

50.43 73.3333

50.44 100

50.45 60

50.46 50

50.47 66.6667

50.48 60

50.49 63.3333

50.5 86.6667

50.51 66.6667

50.52 93.3333

50.53 43.3333

50.54 96.6667

50.55 33.3333

50.56 76.6667

50.57 80

50.58 63.3333

50.59 96.6667

50.6 50

50.61 53.3333

50.62 43.3333

50.63 56.6667

50.64 43.3333

50.65 63.3333

50.66 93.3333

50.67 53.3333

50.68 116.667

50.69 70

50.7 100

50.71 103.333

50.72 56.6667

50.73 83.3333

50.74 56.6667

50.75 66.6667

50.76 86.6667

50.77 80

50.78 46.6667

50.79 80

50.8 83.3333

50.81 93.3333

50.82 73.3333

50.83 76.6667

50.84 76.6667

50.85 56.6667

50.86 76.6667

50.87 66.6667

50.88 70

50.89 100

50.9 83.3333

50.91 50

50.92 40

50.93 93.3333

50.94 103.333

50.95 66.6667

50.96 73.3333

50.97 100

50.98 86.6667

50.99 36.6667

51 90

51.01 93.3333

51.02 90

51.03 73.3333

51.04 96.6667

51.05 106.667

51.06 80

51.07 63.3333

51.08 103.333

51.09 76.6667

51.1 40

51.11 80

51.12 86.6667

51.13 63.3333

51.14 93.3333

51.15 50

51.16 56.6667

51.17 66.6667

51.18 76.6667

51.19 103.333

51.2 36.6667

51.21 30

51.22 106.667

51.23 56.6667

51.24 80

51.25 90

51.26 106.667

51.27 43.3333

51.28 60

51.29 46.6667

51.3 96.6667

51.31 46.6667

51.32 76.6667

51.33 83.3333

51.34 66.6667

51.35 56.6667

51.36 66.6667

51.37 56.6667

51.38 46.6667

51.39 83.3333

51.4 73.3333

51.41 80

51.42 70

51.43 63.3333

51.44 66.6667

51.45 63.3333

51.46 73.3333

51.47 70

51.48 90

51.49 90

51.5 56.6667

51.51 60

51.52 53.3333

51.53 73.3333

51.54 56.6667

51.55 50

51.56 66.6667

51.57 83.3333

51.58 66.6667

51.59 93.3333

51.6 66.6667

51.61 66.6667

51.62 56.6667

51.63 63.3333

51.64 76.6667

51.65 66.6667

51.66 83.3333

51.67 63.3333

51.68 66.6667

51.69 60

51.7 66.6667

51.71 66.6667

51.72 63.3333

51.73 80

51.74 56.6667

51.75 76.6667

51.76 43.3333

51.77 53.3333

51.78 80

51.79 46.6667

51.8 53.3333

51.81 56.6667

51.82 83.3333

51.83 46.6667

51.84 76.6667

51.85 60

51.86 76.6667

51.87 70

51.88 63.3333

51.89 63.3333

51.9 53.3333

51.91 56.6667

51.92 73.3333

51.93 86.6667

51.94 73.3333

51.95 63.3333

51.96 83.3333

51.97 73.3333

51.98 66.6667

51.99 80

52 70

52.01 76.6667

52.02 66.6667

52.03 83.3333

52.04 66.6667

52.05 70

52.06 70

52.07 60

52.08 73.3333

52.09 46.6667

52.1 70

52.11 60

52.12 56.6667

52.13 100

52.14 60

52.15 90

52.16 83.3333

52.17 73.3333

52.18 76.6667

52.19 83.3333

52.2 70

52.21 56.6667

52.22 66.6667

52.23 93.3333

52.24 46.6667

52.25 80

52.26 56.6667

52.27 56.6667

52.28 80

52.29 63.3333

52.3 46.6667

52.31 73.3333

52.32 96.6667

52.33 76.6667

52.34 63.3333

52.35 70

52.36 63.3333

52.37 60

52.38 60

52.39 86.6667

52.4 63.3333

52.41 70

52.42 90

52.43 76.6667

52.44 53.3333

52.45 73.3333

52.46 30

52.47 60

52.48 100

52.49 56.6667

52.5 46.6667

52.51 56.6667

52.52 76.6667

52.53 66.6667

52.54 66.6667

52.55 73.3333

52.56 80

52.57 73.3333

52.58 33.3333

52.59 50

52.6 76.6667

52.61 43.3333

52.62 103.333

52.63 80

52.64 66.6667

52.65 80

52.66 66.6667

52.67 96.6667

52.68 93.3333

52.69 60

52.7 53.3333

52.71 76.6667

52.72 73.3333

52.73 63.3333

52.74 66.6667

52.75 93.3333

52.76 70

52.77 93.3333

52.78 43.3333

52.79 40

52.8 73.3333

52.81 70

52.82 60

52.83 60

52.84 70

52.85 70

52.86 56.6667

52.87 83.3333

52.88 76.6667

52.89 56.6667

52.9 80

52.91 43.3333

52.92 73.3333

52.93 70

52.94 50

52.95 70

52.96 80

52.97 86.6667

52.98 106.667

52.99 90

53 83.3333

53.01 66.6667

53.02 76.6667

53.03 86.6667

53.04 53.3333

53.05 76.6667

53.06 80

53.07 56.6667

53.08 60

53.09 56.6667

53.1 70

53.11 66.6667

53.12 63.3333

53.13 56.6667

53.14 86.6667

53.15 50

53.16 56.6667

53.17 93.3333

53.18 56.6667

53.19 73.3333

53.2 96.6667

53.21 76.6667

53.22 70

53.23 86.6667

53.24 66.6667

53.25 80

53.26 70

53.27 53.3333

53.28 46.6667

53.29 50

53.3 66.6667

53.31 110

53.32 63.3333

53.33 73.3333

53.34 53.3333

53.35 46.6667

53.36 60

53.37 60

53.38 80

53.39 76.6667

53.4 76.6667

53.41 103.333

53.42 73.3333

53.43 83.3333

53.44 106.667

53.45 66.6667

53.46 73.3333

53.47 110

53.48 36.6667

53.49 53.3333

53.5 93.3333

53.51 66.6667

53.52 63.3333

53.53 43.3333

53.54 80

53.55 76.6667

53.56 63.3333

53.57 70

53.58 56.6667

53.59 60

53.6 83.3333

53.61 63.3333

53.62 93.3333

53.63 43.3333

53.64 56.6667

53.65 100

53.66 60

53.67 40

53.68 53.3333

53.69 90

53.7 83.3333

53.71 90

53.72 70

53.73 73.3333

53.74 80

53.75 60

53.76 66.6667

53.77 50

53.78 70

53.79 70

53.8 90

53.81 66.6667

53.82 63.3333

53.83 96.6667

53.84 93.3333

53.85 73.3333

53.86 60

53.87 50

53.88 83.3333

53.89 63.3333

53.9 73.3333

53.91 73.3333

53.92 80

53.93 80

53.94 86.6667

53.95 60

53.96 53.3333

53.97 76.6667

53.98 66.6667

53.99 83.3333

54 73.3333

54.01 80

54.02 76.6667

54.03 73.3333

54.04 90

54.05 76.6667

54.06 60

54.07 70

54.08 70

54.09 63.3333

54.1 46.6667

54.11 53.3333

54.12 60

54.13 80

54.14 63.3333

54.15 86.6667

54.16 60

54.17 63.3333

54.18 86.6667

54.19 63.3333

54.2 66.6667

54.21 50

54.22 76.6667

54.23 63.3333

54.24 36.6667

54.25 73.3333

54.26 66.6667

54.27 63.3333

54.28 76.6667

54.29 76.6667

54.3 50

54.31 60

54.32 50

54.33 70

54.34 80

54.35 76.6667

54.36 73.3333

54.37 66.6667

54.38 90

54.39 76.6667

54.4 66.6667

54.41 96.6667

54.42 93.3333

54.43 83.3333

54.44 53.3333

54.45 96.6667

54.46 90

54.47 53.3333

54.48 36.6667

54.49 70

54.5 80

54.51 70

54.52 60

54.53 83.3333

54.54 66.6667

54.55 46.6667

54.56 56.6667

54.57 60

54.58 73.3333

54.59 63.3333

54.6 63.3333

54.61 66.6667

54.62 86.6667

54.63 60

54.64 83.3333

54.65 56.6667

54.66 73.3333

54.67 83.3333

54.68 53.3333

54.69 66.6667

54.7 66.6667

54.71 33.3333

54.72 73.3333

54.73 76.6667

54.74 73.3333

54.75 86.6667

54.76 80

54.77 60

54.78 90

54.79 96.6667

54.8 63.3333

54.81 86.6667

54.82 106.667

54.83 63.3333

54.84 73.3333

54.85 56.6667

54.86 66.6667

54.87 73.3333

54.88 60

54.89 63.3333

54.9 50

54.91 70

54.92 76.6667

54.93 70

54.94 80

54.95 63.3333

54.96 50

54.97 56.6667

54.98 73.3333

54.99 70

55 86.6667

55.01 60

55.02 86.6667

55.03 70

55.04 56.6667

55.05 80

55.06 73.3333

55.07 86.6667

55.08 86.6667

55.09 56.6667

55.1 66.6667

55.11 70

55.12 80

55.13 90

55.14 73.3333

55.15 70

55.16 50

55.17 56.6667

55.18 93.3333

55.19 50

55.2 90

55.21 86.6667

55.22 76.6667

55.23 86.6667

55.24 76.6667

55.25 76.6667

55.26 56.6667

55.27 56.6667

55.28 50

55.29 86.6667

55.3 70

55.31 50

55.32 76.6667

55.33 63.3333

55.34 83.3333

55.35 40

55.36 56.6667

55.37 40

55.38 63.3333

55.39 70

55.4 83.3333

55.41 66.6667

55.42 70

55.43 43.3333

55.44 73.3333

55.45 80

55.46 80

55.47 70

55.48 50

55.49 76.6667

55.5 76.6667

55.51 66.6667

55.52 63.3333

55.53 70

55.54 96.6667

55.55 63.3333

55.56 80

55.57 80

55.58 63.3333

55.59 43.3333

55.6 63.3333

55.61 63.3333

55.62 56.6667

55.63 66.6667

55.64 46.6667

55.65 73.3333

55.66 86.6667

55.67 76.6667

55.68 66.6667

55.69 66.6667

55.7 83.3333

55.71 83.3333

55.72 80

55.73 90

55.74 53.3333

55.75 53.3333

55.76 66.6667

55.77 86.6667

55.78 43.3333

55.79 80

55.8 73.3333

55.81 63.3333

55.82 80

55.83 56.6667

55.84 76.6667

55.85 93.3333

55.86 83.3333

55.87 60

55.88 86.6667

55.89 73.3333

55.9 56.6667

55.91 60

55.92 63.3333

55.93 83.3333

55.94 73.3333

55.95 66.6667

55.96 56.6667

55.97 76.6667

55.98 90

55.99 63.3333

56 40

56.01 83.3333

56.02 66.6667

56.03 70

56.04 53.3333

56.05 83.3333

56.06 50

56.07 43.3333

56.08 60

56.09 80

56.1 63.3333

56.11 43.3333

56.12 86.6667

56.13 73.3333

56.14 70

56.15 66.6667

56.16 73.3333

56.17 73.3333

56.18 50

56.19 100

56.2 70

56.21 80

56.22 63.3333

56.23 66.6667

56.24 83.3333

56.25 56.6667

56.26 73.3333

56.27 50

56.28 50

56.29 86.6667

56.3 70

56.31 60

56.32 53.3333

56.33 93.3333

56.34 96.6667

56.35 90

56.36 56.6667

56.37 60

56.38 56.6667

56.39 60

56.4 46.6667

56.41 93.3333

56.42 100

56.43 96.6667

56.44 90

56.45 63.3333

56.46 60

56.47 106.667

56.48 83.3333

56.49 70

56.5 96.6667

56.51 73.3333

56.52 103.333

56.53 60

56.54 83.3333

56.55 86.6667

56.56 66.6667

56.57 73.3333

56.58 50

56.59 93.3333

56.6 63.3333

56.61 86.6667

56.62 66.6667

56.63 66.6667

56.64 50

56.65 66.6667

56.66 63.3333

56.67 46.6667

56.68 63.3333

56.69 60

56.7 80

56.71 76.6667

56.72 56.6667

56.73 66.6667

56.74 40

56.75 83.3333

56.76 50

56.77 83.3333

56.78 80

56.79 56.6667

56.8 63.3333

56.81 86.6667

56.82 70

56.83 56.6667

56.84 90

56.85 40

56.86 73.3333

56.87 63.3333

56.88 70

56.89 86.6667

56.9 76.6667

56.91 60

56.92 60

56.93 66.6667

56.94 56.6667

56.95 90

56.96 66.6667

56.97 100

56.98 73.3333

56.99 50

57 56.6667

57.01 73.3333

57.02 76.6667

57.03 56.6667

57.04 66.6667

57.05 70

57.06 56.6667

57.07 56.6667

57.08 66.6667

57.09 90

57.1 53.3333

57.11 56.6667

57.12 63.3333

57.13 80

57.14 83.3333

57.15 90

57.16 73.3333

57.17 36.6667

57.18 43.3333

57.19 80

57.2 40

57.21 63.3333

57.22 36.6667

57.23 83.3333

57.24 60

57.25 63.3333

57.26 66.6667

57.27 70

57.28 66.6667

57.29 86.6667

57.3 70

57.31 73.3333

57.32 70

57.33 63.3333

57.34 63.3333

57.35 40

57.36 50

57.37 76.6667

57.38 66.6667

57.39 56.6667

57.4 56.6667

57.41 80

57.42 46.6667

57.43 63.3333

57.44 56.6667

57.45 73.3333

57.46 73.3333

57.47 46.6667

57.48 60

57.49 83.3333

57.5 40

57.51 76.6667

57.52 43.3333

57.53 83.3333

57.54 70

57.55 60

57.56 120

57.57 70

57.58 66.6667

57.59 60

57.6 66.6667

57.61 63.3333

57.62 86.6667

57.63 90

57.64 63.3333

57.65 56.6667

57.66 50

57.67 76.6667

57.68 90

57.69 80

57.7 73.3333

57.71 30

57.72 83.3333

57.73 80

57.74 73.3333

57.75 73.3333

57.76 50

57.77 86.6667

57.78 80

57.79 90

57.8 76.6667

57.81 73.3333

57.82 80

57.83 63.3333

57.84 73.3333

57.85 46.6667

57.86 76.6667

57.87 53.3333

57.88 83.3333

57.89 80

57.9 110

57.91 56.6667

57.92 76.6667

57.93 66.6667

57.94 60

57.95 60

57.96 43.3333

57.97 86.6667

57.98 36.6667

57.99 83.3333

58 63.3333

58.01 73.3333

58.02 63.3333

58.03 56.6667

58.04 70

58.05 70

58.06 76.6667

58.07 60

58.08 86.6667

58.09 66.6667

58.1 56.6667

58.11 56.6667

58.12 83.3333

58.13 70

58.14 60

58.15 83.3333

58.16 70

58.17 80

58.18 76.6667

58.19 56.6667

58.2 80

58.21 103.333

58.22 60

58.23 66.6667

58.24 46.6667

58.25 63.3333

58.26 90

58.27 43.3333

58.28 56.6667

58.29 80

58.3 76.6667

58.31 66.6667

58.32 103.333

58.33 70

58.34 46.6667

58.35 80

58.36 63.3333

58.37 76.6667

58.38 60

58.39 70

58.4 63.3333

58.41 46.6667

58.42 63.3333

58.43 33.3333

58.44 70

58.45 60

58.46 70

58.47 90

58.48 83.3333

58.49 63.3333

58.5 73.3333

58.51 106.667

58.52 73.3333

58.53 73.3333

58.54 86.6667

58.55 70

58.56 96.6667

58.57 50

58.58 60

58.59 76.6667

58.6 50

58.61 73.3333

58.62 73.3333

58.63 100

58.64 90

58.65 60

58.66 100

58.67 53.3333

58.68 76.6667

58.69 73.3333

58.7 76.6667

58.71 80

58.72 76.6667

58.73 70

58.74 73.3333

58.75 60

58.76 80

58.77 83.3333

58.78 96.6667

58.79 53.3333

58.8 50

58.81 43.3333

58.82 90

58.83 76.6667

58.84 83.3333

58.85 66.6667

58.86 63.3333

58.87 76.6667

58.88 60

58.89 83.3333

58.9 66.6667

58.91 63.3333

58.92 53.3333

58.93 50

58.94 83.3333

58.95 73.3333

58.96 50

58.97 70

58.98 70

58.99 76.6667

59 73.3333

59.01 56.6667

59.02 76.6667

59.03 70

59.04 100

59.05 63.3333

59.06 63.3333

59.07 66.6667

59.08 73.3333

59.09 73.3333

59.1 46.6667

59.11 30

59.12 70

59.13 43.3333

59.14 56.6667

59.15 70

59.16 86.6667

59.17 76.6667

59.18 76.6667

59.19 100

59.2 66.6667

59.21 46.6667

59.22 63.3333

59.23 70

59.24 70

59.25 63.3333

59.26 63.3333

59.27 56.6667

59.28 123.333

59.29 66.6667

59.3 70

59.31 56.6667

59.32 90

59.33 66.6667

59.34 96.6667

59.35 76.6667

59.36 60

59.37 76.6667

59.38 53.3333

59.39 66.6667

59.4 83.3333

59.41 60

59.42 80

59.43 80

59.44 90

59.45 63.3333

59.46 90

59.47 80

59.48 96.6667

59.49 83.3333

59.5 70

59.51 60

59.52 76.6667

59.53 56.6667

59.54 86.6667

59.55 70

59.56 76.6667

59.57 73.3333

59.58 86.6667

59.59 63.3333

59.6 66.6667

59.61 96.6667

59.62 70

59.63 80

59.64 73.3333

59.65 70

59.66 70

59.67 76.6667

59.68 96.6667

59.69 70

59.7 66.6667

59.71 86.6667

59.72 90

59.73 83.3333

59.74 76.6667

59.75 70

59.76 63.3333

59.77 50

59.78 86.6667

59.79 50

59.8 76.6667

59.81 86.6667

59.82 70

59.83 56.6667

59.84 76.6667

59.85 73.3333

59.86 90

59.87 70

59.88 96.6667

59.89 66.6667

59.9 76.6667

59.91 73.3333

59.92 63.3333

59.93 23.3333

59.94 46.6667

59.95 63.3333

59.96 50

59.97 86.6667

59.98 73.3333

59.99 80

60 43.3333

60.01 93.3333

60.02 83.3333

60.03 80

60.04 106.667

60.05 80

60.06 53.3333

60.07 86.6667

60.08 63.3333

60.09 53.3333

60.1 86.6667

60.11 86.6667

60.12 73.3333

60.13 43.3333

60.14 73.3333

60.15 76.6667

60.16 56.6667

60.17 36.6667

60.18 93.3333

60.19 83.3333

60.2 73.3333

60.21 46.6667

60.22 73.3333

60.23 100

60.24 103.333

60.25 66.6667

60.26 73.3333

60.27 83.3333

60.28 50

60.29 70

60.3 80

60.31 70

60.32 53.3333

60.33 76.6667

60.34 110

60.35 63.3333

60.36 83.3333

60.37 53.3333

60.38 96.6667

60.39 60

60.4 90

60.41 60

60.42 73.3333

60.43 66.6667

60.44 100

60.45 76.6667

60.46 96.6667

60.47 70

60.48 83.3333

60.49 80

60.5 83.3333

60.51 50

60.52 90

60.53 66.6667

60.54 46.6667

60.55 53.3333

60.56 76.6667

60.57 63.3333

60.58 53.3333

60.59 70

60.6 50

60.61 66.6667

60.62 80

60.63 60

60.64 73.3333

60.65 80

60.66 50

60.67 90

60.68 63.3333

60.69 50

60.7 63.3333

60.71 60

60.72 83.3333

60.73 76.6667

60.74 53.3333

60.75 66.6667

60.76 60

60.77 93.3333

60.78 66.6667

60.79 90

60.8 86.6667

60.81 106.667

60.82 73.3333

60.83 76.6667

60.84 90

60.85 66.6667

60.86 86.6667

60.87 76.6667

60.88 76.6667

60.89 60

60.9 93.3333

60.91 73.3333

60.92 66.6667

60.93 80

60.94 66.6667

60.95 80

60.96 86.6667

60.97 96.6667

60.98 90

60.99 63.3333

61 63.3333

61.01 80

61.02 43.3333

61.03 63.3333

61.04 46.6667

61.05 93.3333

61.06 73.3333

61.07 66.6667

61.08 63.3333

61.09 83.3333

61.1 66.6667

61.11 63.3333

61.12 63.3333

61.13 60

61.14 50

61.15 100

61.16 53.3333

61.17 60

61.18 76.6667

61.19 73.3333

61.2 60

61.21 60

61.22 83.3333

61.23 83.3333

61.24 63.3333

61.25 60

61.26 100

61.27 66.6667

61.28 73.3333

61.29 63.3333

61.3 80

61.31 83.3333

61.32 53.3333

61.33 43.3333

61.34 90

61.35 53.3333

61.36 56.6667

61.37 70

61.38 60

61.39 60

61.4 70

61.41 76.6667

61.42 83.3333

61.43 66.6667

61.44 66.6667

61.45 110

61.46 83.3333

61.47 70

61.48 66.6667

61.49 73.3333

61.5 73.3333

61.51 86.6667

61.52 70

61.53 70

61.54 76.6667

61.55 70

61.56 96.6667

61.57 86.6667

61.58 73.3333

61.59 50

61.6 56.6667

61.61 80

61.62 73.3333

61.63 76.6667

61.64 66.6667

61.65 73.3333

61.66 80

61.67 63.3333

61.68 90

61.69 73.3333

61.7 73.3333

61.71 90

61.72 73.3333

61.73 73.3333

61.74 93.3333

61.75 63.3333

61.76 96.6667

61.77 93.3333

61.78 60

61.79 73.3333

61.8 83.3333

61.81 80

61.82 73.3333

61.83 60

61.84 83.3333

61.85 60

61.86 96.6667

61.87 76.6667

61.88 96.6667

61.89 76.6667

61.9 63.3333

61.91 80

61.92 96.6667

61.93 70

61.94 93.3333

61.95 86.6667

61.96 60

61.97 93.3333

61.98 76.6667

61.99 63.3333

62 76.6667

62.01 46.6667

62.02 70

62.03 53.3333

62.04 66.6667

62.05 56.6667

62.06 76.6667

62.07 93.3333

62.08 86.6667

62.09 66.6667

62.1 90

62.11 76.6667

62.12 60

62.13 43.3333

62.14 60

62.15 73.3333

62.16 50

62.17 60

62.18 73.3333

62.19 66.6667

62.2 66.6667

62.21 66.6667

62.22 73.3333

62.23 53.3333

62.24 90

62.25 63.3333

62.26 66.6667

62.27 73.3333

62.28 73.3333

62.29 83.3333

62.3 90

62.31 96.6667

62.32 93.3333

62.33 63.3333

62.34 76.6667

62.35 83.3333

62.36 66.6667

62.37 43.3333

62.38 73.3333

62.39 66.6667

62.4 80

62.41 70

62.42 100

62.43 93.3333

62.44 53.3333

62.45 100

62.46 63.3333

62.47 96.6667

62.48 93.3333

62.49 93.3333

62.5 70

62.51 76.6667

62.52 113.333

62.53 96.6667

62.54 90

62.55 63.3333

62.56 80

62.57 60

62.58 56.6667

62.59 90

62.6 66.6667

62.61 86.6667

62.62 73.3333

62.63 96.6667

62.64 66.6667

62.65 66.6667

62.66 86.6667

62.67 56.6667

62.68 50

62.69 60

62.7 80

62.71 63.3333

62.72 53.3333

62.73 70

62.74 60

62.75 86.6667

62.76 90

62.77 70

62.78 63.3333

62.79 63.3333

62.8 93.3333

62.81 66.6667

62.82 70

62.83 63.3333

62.84 80

62.85 73.3333

62.86 70

62.87 93.3333

62.88 63.3333

62.89 80

62.9 76.6667

62.91 63.3333

62.92 50

62.93 80

62.94 46.6667

62.95 80

62.96 63.3333

62.97 80

62.98 73.3333

62.99 93.3333

63 66.6667

63.01 53.3333

63.02 60

63.03 76.6667

63.04 103.333

63.05 63.3333

63.06 100

63.07 76.6667

63.08 70

63.09 80

63.1 60

63.11 53.3333

63.12 50

63.13 53.3333

63.14 73.3333

63.15 76.6667

63.16 46.6667

63.17 76.6667

63.18 80

63.19 80

63.2 50

63.21 70

63.22 60

63.23 86.6667

63.24 50

63.25 76.6667

63.26 63.3333

63.27 86.6667

63.28 46.6667

63.29 53.3333

63.3 76.6667

63.31 50

63.32 63.3333

63.33 66.6667

63.34 76.6667

63.35 80

63.36 63.3333

63.37 73.3333

63.38 46.6667

63.39 66.6667

63.4 83.3333

63.41 70

63.42 56.6667

63.43 96.6667

63.44 70

63.45 66.6667

63.46 76.6667

63.47 86.6667

63.48 76.6667

63.49 53.3333

63.5 66.6667

63.51 80

63.52 50

63.53 53.3333

63.54 56.6667

63.55 83.3333

63.56 63.3333

63.57 40

63.58 50

63.59 86.6667

63.6 86.6667

63.61 76.6667

63.62 53.3333

63.63 50

63.64 73.3333

63.65 96.6667

63.66 70

63.67 80

63.68 93.3333

63.69 86.6667

63.7 80

63.71 93.3333

63.72 76.6667

63.73 93.3333

63.74 83.3333

63.75 73.3333

63.76 60

63.77 76.6667

63.78 86.6667

63.79 60

63.8 86.6667

63.81 66.6667

63.82 60

63.83 86.6667

63.84 93.3333

63.85 66.6667

63.86 73.3333

63.87 83.3333

63.88 43.3333

63.89 83.3333

63.9 86.6667

63.91 70

63.92 60

63.93 100

63.94 86.6667

63.95 73.3333

63.96 60

63.97 66.6667

63.98 100

63.99 53.3333

64 70

64.01 96.6667

64.02 90

64.03 93.3333

64.04 70

64.05 93.3333

64.06 70

64.07 70

64.08 66.6667

64.09 83.3333

64.1 50

64.11 93.3333

64.12 86.6667

64.13 60

64.14 76.6667

64.15 60

64.16 93.3333

64.17 63.3333

64.18 80

64.19 86.6667

64.2 90

64.21 80

64.22 100

64.23 56.6667

64.24 76.6667

64.25 96.6667

64.26 90

64.27 80

64.28 80

64.29 80

64.3 96.6667

64.31 80

64.32 56.6667

64.33 53.3333

64.34 56.6667

64.35 73.3333

64.36 60

64.37 70

64.38 56.6667

64.39 53.3333

64.4 70

64.41 86.6667

64.42 100

64.43 66.6667

64.44 103.333

64.45 50

64.46 86.6667

64.47 86.6667

64.48 73.3333

64.49 113.333

64.5 63.3333

64.51 93.3333

64.52 73.3333

64.53 63.3333

64.54 60

64.55 66.6667

64.56 56.6667

64.57 66.6667

64.58 83.3333

64.59 100

64.6 80

64.61 56.6667

64.62 86.6667

64.63 73.3333

64.64 110

64.65 120

64.66 76.6667

64.67 46.6667

64.68 70

64.69 76.6667

64.7 73.3333

64.71 76.6667

64.72 43.3333

64.73 100

64.74 73.3333

64.75 86.6667

64.76 110

64.77 50

64.78 100

64.79 106.667

64.8 80

64.81 86.6667

64.82 93.3333

64.83 103.333

64.84 83.3333

64.85 86.6667

64.86 50

64.87 90

64.88 93.3333

64.89 96.6667

64.9 120

64.91 110

64.92 96.6667

64.93 110

64.94 110

64.95 96.6667

64.96 140

64.97 146.667

64.98 220

64.99 180

65 230

65.01 223.333

65.02 333.333

65.03 393.333

65.04 370

65.05 440

65.06 393.333

65.07 400

65.08 343.333

65.09 323.333

65.1 256.667

65.11 210

65.12 180

65.13 213.333

65.14 143.333

65.15 163.333

65.16 170

65.17 130

65.18 136.667

65.19 196.667

65.2 183.333

65.21 160

65.22 216.667

65.23 200

65.24 263.333

65.25 173.333

65.26 200

65.27 153.333

65.28 170

65.29 153.333

65.3 106.667

65.31 123.333

65.32 130

65.33 100

65.34 143.333

65.35 100

65.36 110

65.37 103.333

65.38 63.3333

65.39 73.3333

65.4 100

65.41 56.6667

65.42 136.667

65.43 73.3333

65.44 100

65.45 76.6667

65.46 63.3333

65.47 56.6667

65.48 90

65.49 96.6667

65.5 53.3333

65.51 113.333

65.52 96.6667

65.53 123.333

65.54 76.6667

65.55 90

65.56 90

65.57 103.333

65.58 86.6667

65.59 83.3333

65.6 73.3333

65.61 60

65.62 80

65.63 90

65.64 63.3333

65.65 50

65.66 70

65.67 70

65.68 93.3333

65.69 76.6667

65.7 83.3333

65.71 93.3333

65.72 96.6667

65.73 70

65.74 103.333

65.75 86.6667

65.76 70

65.77 70

65.78 80

65.79 73.3333

65.8 73.3333

65.81 70

65.82 76.6667

65.83 86.6667

65.84 80

65.85 66.6667

65.86 76.6667

65.87 93.3333

65.88 90

65.89 93.3333

65.9 73.3333

65.91 56.6667

65.92 46.6667

65.93 83.3333

65.94 73.3333

65.95 93.3333

65.96 83.3333

65.97 46.6667

65.98 100

65.99 46.6667

66 73.3333

66.01 70

66.02 53.3333

66.03 90

66.04 90

66.05 106.667

66.06 63.3333

66.07 103.333

66.08 43.3333

66.09 66.6667

66.1 86.6667

66.11 53.3333

66.12 76.6667

66.13 90

66.14 86.6667

66.15 96.6667

66.16 86.6667

66.17 83.3333

66.18 80

66.19 80

66.2 76.6667

66.21 86.6667

66.22 66.6667

66.23 76.6667

66.24 60

66.25 63.3333

66.26 56.6667

66.27 90

66.28 76.6667

66.29 46.6667

66.3 46.6667

66.31 63.3333

66.32 63.3333

66.33 46.6667

66.34 76.6667

66.35 60

66.36 83.3333

66.37 70

66.38 76.6667

66.39 60

66.4 66.6667

66.41 70

66.42 73.3333

66.43 66.6667

66.44 83.3333

66.45 76.6667

66.46 63.3333

66.47 73.3333

66.48 73.3333

66.49 80

66.5 83.3333

66.51 83.3333

66.52 76.6667

66.53 53.3333

66.54 46.6667

66.55 116.667

66.56 63.3333

66.57 70

66.58 83.3333

66.59 76.6667

66.6 83.3333

66.61 66.6667

66.62 60

66.63 80

66.64 76.6667

66.65 60

66.66 70

66.67 73.3333

66.68 63.3333

66.69 66.6667

66.7 116.667

66.71 53.3333

66.72 80

66.73 80

66.74 73.3333

66.75 56.6667

66.76 90

66.77 56.6667

66.78 90

66.79 86.6667

66.8 46.6667

66.81 80

66.82 56.6667

66.83 50

66.84 46.6667

66.85 70

66.86 70

66.87 66.6667

66.88 70

66.89 80

66.9 73.3333

66.91 73.3333

66.92 76.6667

66.93 76.6667

66.94 76.6667

66.95 73.3333

66.96 83.3333

66.97 80

66.98 66.6667

66.99 73.3333

67 96.6667

67.01 86.6667

67.02 80

67.03 50

67.04 46.6667

67.05 56.6667

67.06 73.3333

67.07 73.3333

67.08 40

67.09 90

67.1 80

67.11 80

67.12 83.3333

67.13 66.6667

67.14 66.6667

67.15 63.3333

67.16 86.6667

67.17 80

67.18 110

67.19 83.3333

67.2 70

67.21 66.6667

67.22 70

67.23 96.6667

67.24 83.3333

67.25 56.6667

67.26 93.3333

67.27 76.6667

67.28 56.6667

67.29 66.6667

67.3 60

67.31 60

67.32 50

67.33 83.3333

67.34 66.6667

67.35 83.3333

67.36 66.6667

67.37 86.6667

67.38 76.6667

67.39 86.6667

67.4 83.3333

67.41 70

67.42 76.6667

67.43 103.333

67.44 93.3333

67.45 83.3333

67.46 46.6667

67.47 103.333

67.48 73.3333

67.49 66.6667

67.5 63.3333

67.51 96.6667

67.52 76.6667

67.53 63.3333

67.54 70

67.55 90

67.56 50

67.57 93.3333

67.58 93.3333

67.59 66.6667

67.6 83.3333

67.61 80

67.62 60

67.63 56.6667

67.64 70

67.65 96.6667

67.66 76.6667

67.67 43.3333

67.68 90

67.69 103.333

67.7 73.3333

67.71 53.3333

67.72 66.6667

67.73 63.3333

67.74 73.3333

67.75 56.6667

67.76 46.6667

67.77 90

67.78 83.3333

67.79 70

67.8 80

67.81 76.6667

67.82 70

67.83 86.6667

67.84 50

67.85 60

67.86 93.3333

67.87 76.6667

67.88 90

67.89 60

67.9 63.3333

67.91 66.6667

67.92 100

67.93 86.6667

67.94 53.3333

67.95 76.6667

67.96 56.6667

67.97 50

67.98 80

67.99 63.3333

68 56.6667

68.01 50

68.02 63.3333

68.03 46.6667

68.04 66.6667

68.05 46.6667

68.06 120

68.07 103.333

68.08 56.6667

68.09 70

68.1 70

68.11 56.6667

68.12 80

68.13 70

68.14 66.6667

68.15 70

68.16 96.6667

68.17 73.3333

68.18 66.6667

68.19 93.3333

68.2 50

68.21 90

68.22 70

68.23 66.6667

68.24 63.3333

68.25 86.6667

68.26 96.6667

68.27 56.6667

68.28 60

68.29 86.6667

68.3 73.3333

68.31 76.6667

68.32 70

68.33 66.6667

68.34 96.6667

68.35 103.333

68.36 90

68.37 63.3333

68.38 66.6667

68.39 53.3333

68.4 83.3333

68.41 73.3333

68.42 60

68.43 53.3333

68.44 76.6667

68.45 93.3333

68.46 46.6667

68.47 126.667

68.48 126.667

68.49 66.6667

68.5 73.3333

68.51 66.6667

68.52 103.333

68.53 66.6667

68.54 50

68.55 63.3333

68.56 86.6667

68.57 50

68.58 70

68.59 76.6667

68.6 80

68.61 76.6667

68.62 80

68.63 86.6667

68.64 70

68.65 70

68.66 73.3333

68.67 83.3333

68.68 73.3333

68.69 56.6667

68.7 60

68.71 66.6667

68.72 86.6667

68.73 56.6667

68.74 60

68.75 86.6667

68.76 70

68.77 86.6667

68.78 56.6667

68.79 86.6667

68.8 86.6667

68.81 70

68.82 110

68.83 70

68.84 76.6667

68.85 93.3333

68.86 53.3333

68.87 103.333

68.88 66.6667

68.89 56.6667

68.9 43.3333

68.91 86.6667

68.92 60

68.93 73.3333

68.94 70

68.95 73.3333

68.96 53.3333

68.97 83.3333

68.98 50

68.99 60

69 66.6667

69.01 83.3333

69.02 50

69.03 70

69.04 90

69.05 50

69.06 73.3333

69.07 50

69.08 73.3333

69.09 83.3333

69.1 80

69.11 43.3333

69.12 66.6667

69.13 80

69.14 93.3333

69.15 73.3333

69.16 80

69.17 73.3333

69.18 80

69.19 83.3333

69.2 50

69.21 60

69.22 76.6667

69.23 103.333

69.24 93.3333

69.25 60

69.26 80

69.27 96.6667

69.28 46.6667

69.29 83.3333

69.3 80

69.31 66.6667

69.32 56.6667

69.33 60

69.34 103.333

69.35 96.6667

69.36 56.6667

69.37 90

69.38 63.3333

69.39 43.3333

69.4 90

69.41 73.3333

69.42 53.3333

69.43 70

69.44 66.6667

69.45 76.6667

69.46 83.3333

69.47 60

69.48 73.3333

69.49 76.6667

69.5 76.6667

69.51 80

69.52 73.3333

69.53 86.6667

69.54 60

69.55 96.6667

69.56 80

69.57 73.3333

69.58 63.3333

69.59 83.3333

69.6 100

69.61 56.6667

69.62 50

69.63 73.3333

69.64 60

69.65 73.3333

69.66 53.3333

69.67 56.6667

69.68 83.3333

69.69 73.3333

69.7 50

69.71 73.3333

69.72 40

69.73 80

69.74 90

69.75 73.3333

69.76 56.6667

69.77 40

69.78 73.3333

69.79 56.6667

69.8 43.3333

69.81 46.6667

69.82 90

69.83 50

69.84 53.3333

69.85 70

69.86 73.3333

69.87 76.6667

69.88 73.3333

69.89 63.3333

69.9 86.6667

69.91 86.6667

69.92 73.3333

69.93 76.6667

69.94 73.3333

69.95 80

69.96 106.667

69.97 50

69.98 70

69.99 76.6667

70 46.6667

**Raw data 6**. XRD raw data of the powder obtained after heat-treatment of as-sprayed powders prepared from the solution with citric acid at 500 ℃.

Goniometer RINT2000 vertical goniometer

Attachment Auto sample changer type B(6 samples)

Monochromater Fixed Monochromator

ScanningMode 2Theta/Theta

ScanningType Continuos Scanning

X-Ray 40kV/100mA

DivSlit 1 deg.

DivH.L.Slit 10mm

SctSlit 1 deg.

RecSlit 0.15mm

Monochro RS No Use

K-beta filter

Start 20

Stop 70

Step 0.01

20 336.667

20.01 476.667

20.02 443.333

20.03 463.333

20.04 426.667

20.05 423.333

20.06 420

20.07 436.667

20.08 346.667

20.09 403.333

20.1 423.333

20.11 426.667

20.12 443.333

20.13 376.667

20.14 396.667

20.15 393.333

20.16 480

20.17 396.667

20.18 443.333

20.19 436.667

20.2 423.333

20.21 343.333

20.22 400

20.23 473.333

20.24 463.333

20.25 410

20.26 370

20.27 396.667

20.28 430

20.29 386.667

20.3 366.667

20.31 516.667

20.32 366.667

20.33 396.667

20.34 420

20.35 390

20.36 433.333

20.37 403.333

20.38 380

20.39 410

20.4 413.333

20.41 433.333

20.42 390

20.43 480

20.44 393.333

20.45 430

20.46 383.333

20.47 456.667

20.48 453.333

20.49 400

20.5 440

20.51 450

20.52 410

20.53 363.333

20.54 436.667

20.55 370

20.56 403.333

20.57 466.667

20.58 390

20.59 456.667

20.6 400

20.61 443.333

20.62 423.333

20.63 400

20.64 350

20.65 460

20.66 463.333

20.67 443.333

20.68 430

20.69 406.667

20.7 393.333

20.71 423.333

20.72 430

20.73 410

20.74 443.333

20.75 420

20.76 403.333

20.77 436.667

20.78 450

20.79 383.333

20.8 376.667

20.81 420

20.82 433.333

20.83 403.333

20.84 446.667

20.85 436.667

20.86 456.667

20.87 373.333

20.88 403.333

20.89 450

20.9 373.333

20.91 406.667

20.92 380

20.93 476.667

20.94 416.667

20.95 440

20.96 373.333

20.97 430

20.98 466.667

20.99 433.333

21 386.667

21.01 420

21.02 416.667

21.03 380

21.04 406.667

21.05 413.333

21.06 443.333

21.07 310

21.08 396.667

21.09 423.333

21.1 446.667

21.11 430

21.12 416.667

21.13 423.333

21.14 346.667

21.15 423.333

21.16 460

21.17 416.667

21.18 430

21.19 450

21.2 373.333

21.21 423.333

21.22 440

21.23 393.333

21.24 386.667

21.25 466.667

21.26 440

21.27 443.333

21.28 426.667

21.29 403.333

21.3 426.667

21.31 406.667

21.32 360

21.33 346.667

21.34 440

21.35 390

21.36 403.333

21.37 403.333

21.38 440

21.39 336.667

21.4 400

21.41 426.667

21.42 376.667

21.43 366.667

21.44 396.667

21.45 423.333

21.46 446.667

21.47 356.667

21.48 503.333

21.49 420

21.5 356.667

21.51 386.667

21.52 376.667

21.53 390

21.54 410

21.55 376.667

21.56 386.667

21.57 383.333

21.58 396.667

21.59 360

21.6 373.333

21.61 426.667

21.62 476.667

21.63 390

21.64 400

21.65 430

21.66 380

21.67 450

21.68 376.667

21.69 396.667

21.7 403.333

21.71 450

21.72 460

21.73 416.667

21.74 463.333

21.75 440

21.76 386.667

21.77 396.667

21.78 386.667

21.79 443.333

21.8 410

21.81 396.667

21.82 403.333

21.83 420

21.84 356.667

21.85 433.333

21.86 443.333

21.87 393.333

21.88 380

21.89 403.333

21.9 383.333

21.91 390

21.92 403.333

21.93 443.333

21.94 363.333

21.95 303.333

21.96 396.667

21.97 413.333

21.98 400

21.99 346.667

22 466.667

22.01 380

22.02 363.333

22.03 340

22.04 370

22.05 336.667

22.06 370

22.07 366.667

22.08 393.333

22.09 353.333

22.1 326.667

22.11 370

22.12 400

22.13 336.667

22.14 353.333

22.15 393.333

22.16 293.333

22.17 406.667

22.18 330

22.19 380

22.2 390

22.21 386.667

22.22 346.667

22.23 386.667

22.24 456.667

22.25 400

22.26 376.667

22.27 363.333

22.28 370

22.29 373.333

22.3 353.333

22.31 386.667

22.32 400

22.33 303.333

22.34 360

22.35 380

22.36 336.667

22.37 343.333

22.38 373.333

22.39 363.333

22.4 333.333

22.41 396.667

22.42 356.667

22.43 330

22.44 393.333

22.45 376.667

22.46 376.667

22.47 333.333

22.48 313.333

22.49 410

22.5 333.333

22.51 353.333

22.52 420

22.53 323.333

22.54 316.667

22.55 416.667

22.56 390

22.57 350

22.58 276.667

22.59 363.333

22.6 316.667

22.61 316.667

22.62 380

22.63 356.667

22.64 296.667

22.65 463.333

22.66 420

22.67 330

22.68 343.333

22.69 353.333

22.7 360

22.71 333.333

22.72 353.333

22.73 306.667

22.74 330

22.75 366.667

22.76 303.333

22.77 366.667

22.78 340

22.79 363.333

22.8 326.667

22.81 290

22.82 396.667

22.83 353.333

22.84 373.333

22.85 296.667

22.86 330

22.87 410

22.88 353.333

22.89 343.333

22.9 333.333

22.91 363.333

22.92 336.667

22.93 353.333

22.94 343.333

22.95 333.333

22.96 346.667

22.97 313.333

22.98 413.333

22.99 306.667

23 310

23.01 320

23.02 316.667

23.03 340

23.04 323.333

23.05 303.333

23.06 386.667

23.07 333.333

23.08 386.667

23.09 330

23.1 313.333

23.11 326.667

23.12 370

23.13 360

23.14 310

23.15 350

23.16 400

23.17 303.333

23.18 340

23.19 323.333

23.2 330

23.21 343.333

23.22 323.333

23.23 296.667

23.24 373.333

23.25 293.333

23.26 310

23.27 286.667

23.28 300

23.29 330

23.3 313.333

23.31 316.667

23.32 283.333

23.33 273.333

23.34 366.667

23.35 266.667

23.36 320

23.37 330

23.38 340

23.39 363.333

23.4 310

23.41 260

23.42 276.667

23.43 326.667

23.44 286.667

23.45 246.667

23.46 276.667

23.47 276.667

23.48 273.333

23.49 266.667

23.5 280

23.51 346.667

23.52 336.667

23.53 293.333

23.54 326.667

23.55 280

23.56 346.667

23.57 303.333

23.58 293.333

23.59 326.667

23.6 303.333

23.61 286.667

23.62 330

23.63 303.333

23.64 310

23.65 303.333

23.66 256.667

23.67 316.667

23.68 296.667

23.69 276.667

23.7 263.333

23.71 233.333

23.72 313.333

23.73 306.667

23.74 253.333

23.75 280

23.76 293.333

23.77 326.667

23.78 273.333

23.79 320

23.8 236.667

23.81 280

23.82 303.333

23.83 306.667

23.84 290

23.85 283.333

23.86 270

23.87 276.667

23.88 273.333

23.89 336.667

23.9 306.667

23.91 276.667

23.92 296.667

23.93 306.667

23.94 300

23.95 293.333

23.96 260

23.97 303.333

23.98 260

23.99 310

24 303.333

24.01 246.667

24.02 220

24.03 246.667

24.04 303.333

24.05 220

24.06 233.333

24.07 283.333

24.08 273.333

24.09 316.667

24.1 253.333

24.11 296.667

24.12 256.667

24.13 246.667

24.14 270

24.15 230

24.16 296.667

24.17 266.667

24.18 273.333

24.19 266.667

24.2 300

24.21 260

24.22 363.333

24.23 260

24.24 246.667

24.25 246.667

24.26 220

24.27 286.667

24.28 256.667

24.29 290

24.3 286.667

24.31 266.667

24.32 263.333

24.33 193.333

24.34 300

24.35 236.667

24.36 286.667

24.37 256.667

24.38 246.667

24.39 256.667

24.4 240

24.41 230

24.42 250

24.43 326.667

24.44 220

24.45 266.667

24.46 260

24.47 213.333

24.48 243.333

24.49 246.667

24.5 236.667

24.51 236.667

24.52 240

24.53 210

24.54 246.667

24.55 266.667

24.56 230

24.57 280

24.58 260

24.59 216.667

24.6 233.333

24.61 266.667

24.62 250

24.63 266.667

24.64 283.333

24.65 270

24.66 230

24.67 223.333

24.68 270

24.69 230

24.7 183.333

24.71 216.667

24.72 190

24.73 246.667

24.74 226.667

24.75 243.333

24.76 256.667

24.77 280

24.78 256.667

24.79 246.667

24.8 283.333

24.81 196.667

24.82 266.667

24.83 243.333

24.84 240

24.85 253.333

24.86 223.333

24.87 240

24.88 203.333

24.89 286.667

24.9 216.667

24.91 240

24.92 233.333

24.93 270

24.94 210

24.95 246.667

24.96 203.333

24.97 226.667

24.98 246.667

24.99 230

25 220

25.01 230

25.02 286.667

25.03 270

25.04 223.333

25.05 220

25.06 196.667

25.07 163.333

25.08 230

25.09 240

25.1 213.333

25.11 186.667

25.12 176.667

25.13 246.667

25.14 213.333

25.15 183.333

25.16 203.333

25.17 190

25.18 200

25.19 233.333

25.2 236.667

25.21 220

25.22 270

25.23 206.667

25.24 186.667

25.25 210

25.26 193.333

25.27 206.667

25.28 200

25.29 260

25.3 173.333

25.31 206.667

25.32 210

25.33 216.667

25.34 193.333

25.35 233.333

25.36 266.667

25.37 266.667

25.38 256.667

25.39 240

25.4 193.333

25.41 193.333

25.42 163.333

25.43 206.667

25.44 150

25.45 220

25.46 163.333

25.47 193.333

25.48 203.333

25.49 180

25.5 203.333

25.51 190

25.52 176.667

25.53 216.667

25.54 196.667

25.55 210

25.56 216.667

25.57 213.333

25.58 213.333

25.59 176.667

25.6 186.667

25.61 243.333

25.62 163.333

25.63 206.667

25.64 203.333

25.65 200

25.66 203.333

25.67 166.667

25.68 163.333

25.69 193.333

25.7 173.333

25.71 233.333

25.72 190

25.73 170

25.74 206.667

25.75 203.333

25.76 190

25.77 180

25.78 213.333

25.79 156.667

25.8 186.667

25.81 166.667

25.82 180

25.83 230

25.84 210

25.85 216.667

25.86 190

25.87 186.667

25.88 210

25.89 160

25.9 180

25.91 146.667

25.92 226.667

25.93 183.333

25.94 203.333

25.95 180

25.96 213.333

25.97 213.333

25.98 203.333

25.99 213.333

26 130

26.01 236.667

26.02 150

26.03 183.333

26.04 176.667

26.05 203.333

26.06 186.667

26.07 183.333

26.08 160

26.09 226.667

26.1 190

26.11 223.333

26.12 160

26.13 160

26.14 193.333

26.15 186.667

26.16 193.333

26.17 170

26.18 150

26.19 163.333

26.2 186.667

26.21 183.333

26.22 196.667

26.23 180

26.24 160

26.25 160

26.26 156.667

26.27 153.333

26.28 176.667

26.29 176.667

26.3 190

26.31 180

26.32 200

26.33 160

26.34 180

26.35 170

26.36 170

26.37 183.333

26.38 160

26.39 156.667

26.4 170

26.41 156.667

26.42 153.333

26.43 163.333

26.44 203.333

26.45 173.333

26.46 150

26.47 173.333

26.48 133.333

26.49 136.667

26.5 173.333

26.51 173.333

26.52 190

26.53 153.333

26.54 170

26.55 163.333

26.56 213.333

26.57 156.667

26.58 143.333

26.59 163.333

26.6 163.333

26.61 196.667

26.62 153.333

26.63 226.667

26.64 163.333

26.65 143.333

26.66 150

26.67 166.667

26.68 166.667

26.69 150

26.7 143.333

26.71 183.333

26.72 136.667

26.73 183.333

26.74 166.667

26.75 160

26.76 143.333

26.77 153.333

26.78 130

26.79 196.667

26.8 173.333

26.81 143.333

26.82 156.667

26.83 153.333

26.84 156.667

26.85 193.333

26.86 173.333

26.87 176.667

26.88 110

26.89 123.333

26.9 110

26.91 183.333

26.92 173.333

26.93 133.333

26.94 163.333

26.95 130

26.96 133.333

26.97 146.667

26.98 153.333

26.99 210

27 196.667

27.01 143.333

27.02 126.667

27.03 186.667

27.04 140

27.05 146.667

27.06 126.667

27.07 143.333

27.08 153.333

27.09 113.333

27.1 143.333

27.11 176.667

27.12 136.667

27.13 166.667

27.14 136.667

27.15 150

27.16 123.333

27.17 123.333

27.18 130

27.19 120

27.2 146.667

27.21 160

27.22 113.333

27.23 163.333

27.24 143.333

27.25 166.667

27.26 133.333

27.27 153.333

27.28 113.333

27.29 143.333

27.3 163.333

27.31 126.667

27.32 146.667

27.33 123.333

27.34 123.333

27.35 143.333

27.36 163.333

27.37 166.667

27.38 140

27.39 156.667

27.4 146.667

27.41 133.333

27.42 143.333

27.43 133.333

27.44 146.667

27.45 120

27.46 126.667

27.47 136.667

27.48 133.333

27.49 156.667

27.5 160

27.51 153.333

27.52 120

27.53 166.667

27.54 163.333

27.55 86.6667

27.56 133.333

27.57 136.667

27.58 136.667

27.59 103.333

27.6 160

27.61 126.667

27.62 170

27.63 166.667

27.64 106.667

27.65 153.333

27.66 110

27.67 160

27.68 113.333

27.69 133.333

27.7 140

27.71 113.333

27.72 113.333

27.73 110

27.74 176.667

27.75 136.667

27.76 126.667

27.77 146.667

27.78 123.333

27.79 123.333

27.8 126.667

27.81 143.333

27.82 106.667

27.83 126.667

27.84 140

27.85 90

27.86 116.667

27.87 163.333

27.88 113.333

27.89 166.667

27.9 113.333

27.91 153.333

27.92 130

27.93 143.333

27.94 140

27.95 136.667

27.96 136.667

27.97 123.333

27.98 133.333

27.99 160

28 133.333

28.01 126.667

28.02 116.667

28.03 143.333

28.04 100

28.05 163.333

28.06 113.333

28.07 186.667

28.08 133.333

28.09 106.667

28.1 153.333

28.11 143.333

28.12 133.333

28.13 103.333

28.14 153.333

28.15 130

28.16 103.333

28.17 100

28.18 183.333

28.19 110

28.2 140

28.21 136.667

28.22 160

28.23 106.667

28.24 136.667

28.25 170

28.26 86.6667

28.27 83.3333

28.28 140

28.29 133.333

28.3 130

28.31 93.3333

28.32 140

28.33 93.3333

28.34 140

28.35 136.667

28.36 156.667

28.37 156.667

28.38 106.667

28.39 136.667

28.4 123.333

28.41 110

28.42 126.667

28.43 156.667

28.44 136.667

28.45 96.6667

28.46 90

28.47 83.3333

28.48 140

28.49 86.6667

28.5 160

28.51 173.333

28.52 120

28.53 126.667

28.54 136.667

28.55 93.3333

28.56 123.333

28.57 103.333

28.58 123.333

28.59 120

28.6 120

28.61 130

28.62 120

28.63 136.667

28.64 123.333

28.65 90

28.66 116.667

28.67 110

28.68 113.333

28.69 133.333

28.7 146.667

28.71 113.333

28.72 103.333

28.73 103.333

28.74 126.667

28.75 113.333

28.76 153.333

28.77 116.667

28.78 106.667

28.79 113.333

28.8 113.333

28.81 80

28.82 116.667

28.83 103.333

28.84 123.333

28.85 106.667

28.86 120

28.87 133.333

28.88 173.333

28.89 83.3333

28.9 130

28.91 113.333

28.92 113.333

28.93 116.667

28.94 86.6667

28.95 120

28.96 160

28.97 136.667

28.98 96.6667

28.99 93.3333

29 126.667

29.01 140

29.02 113.333

29.03 133.333

29.04 116.667

29.05 116.667

29.06 100

29.07 96.6667

29.08 76.6667

29.09 100

29.1 80

29.11 116.667

29.12 103.333

29.13 103.333

29.14 73.3333

29.15 80

29.16 130

29.17 116.667

29.18 103.333

29.19 130

29.2 130

29.21 93.3333

29.22 140

29.23 106.667

29.24 73.3333

29.25 130

29.26 86.6667

29.27 73.3333

29.28 106.667

29.29 120

29.3 86.6667

29.31 93.3333

29.32 130

29.33 110

29.34 153.333

29.35 100

29.36 90

29.37 120

29.38 116.667

29.39 110

29.4 120

29.41 123.333

29.42 126.667

29.43 96.6667

29.44 103.333

29.45 90

29.46 93.3333

29.47 126.667

29.48 86.6667

29.49 120

29.5 83.3333

29.51 90

29.52 120

29.53 110

29.54 73.3333

29.55 90

29.56 86.6667

29.57 143.333

29.58 80

29.59 113.333

29.6 90

29.61 93.3333

29.62 93.3333

29.63 96.6667

29.64 70

29.65 110

29.66 123.333

29.67 110

29.68 96.6667

29.69 96.6667

29.7 126.667

29.71 96.6667

29.72 103.333

29.73 80

29.74 106.667

29.75 80

29.76 106.667

29.77 96.6667

29.78 103.333

29.79 90

29.8 90

29.81 106.667

29.82 143.333

29.83 100

29.84 120

29.85 100

29.86 123.333

29.87 130

29.88 116.667

29.89 126.667

29.9 106.667

29.91 103.333

29.92 90

29.93 93.3333

29.94 80

29.95 103.333

29.96 83.3333

29.97 120

29.98 83.3333

29.99 110

30 113.333

30.01 123.333

30.02 106.667

30.03 86.6667

30.04 93.3333

30.05 66.6667

30.06 120

30.07 90

30.08 113.333

30.09 80

30.1 93.3333

30.11 106.667

30.12 83.3333

30.13 110

30.14 116.667

30.15 73.3333

30.16 106.667

30.17 83.3333

30.18 123.333

30.19 110

30.2 73.3333

30.21 96.6667

30.22 63.3333

30.23 126.667

30.24 113.333

30.25 106.667

30.26 76.6667

30.27 100

30.28 80

30.29 106.667

30.3 96.6667

30.31 103.333

30.32 100

30.33 133.333

30.34 100

30.35 96.6667

30.36 76.6667

30.37 63.3333

30.38 90

30.39 53.3333

30.4 90

30.41 86.6667

30.42 103.333

30.43 66.6667

30.44 103.333

30.45 73.3333

30.46 123.333

30.47 133.333

30.48 103.333

30.49 93.3333

30.5 86.6667

30.51 80

30.52 73.3333

30.53 100

30.54 86.6667

30.55 76.6667

30.56 126.667

30.57 76.6667

30.58 96.6667

30.59 83.3333

30.6 90

30.61 93.3333

30.62 70

30.63 76.6667

30.64 93.3333

30.65 96.6667

30.66 96.6667

30.67 86.6667

30.68 100

30.69 86.6667

30.7 116.667

30.71 73.3333

30.72 63.3333

30.73 76.6667

30.74 106.667

30.75 86.6667

30.76 116.667

30.77 73.3333

30.78 66.6667

30.79 76.6667

30.8 136.667

30.81 100

30.82 76.6667

30.83 96.6667

30.84 123.333

30.85 116.667

30.86 86.6667

30.87 60

30.88 83.3333

30.89 83.3333

30.9 126.667

30.91 100

30.92 73.3333

30.93 103.333

30.94 83.3333

30.95 76.6667

30.96 126.667

30.97 130

30.98 80

30.99 100

31 56.6667

31.01 106.667

31.02 73.3333

31.03 73.3333

31.04 116.667

31.05 86.6667

31.06 80

31.07 73.3333

31.08 100

31.09 83.3333

31.1 116.667

31.11 90

31.12 103.333

31.13 76.6667

31.14 73.3333

31.15 66.6667

31.16 116.667

31.17 60

31.18 83.3333

31.19 96.6667

31.2 76.6667

31.21 126.667

31.22 113.333

31.23 83.3333

31.24 90

31.25 73.3333

31.26 70

31.27 133.333

31.28 93.3333

31.29 73.3333

31.3 83.3333

31.31 60

31.32 90

31.33 56.6667

31.34 93.3333

31.35 80

31.36 80

31.37 90

31.38 66.6667

31.39 113.333

31.4 100

31.41 93.3333

31.42 96.6667

31.43 86.6667

31.44 73.3333

31.45 90

31.46 73.3333

31.47 53.3333

31.48 70

31.49 86.6667

31.5 116.667

31.51 63.3333

31.52 73.3333

31.53 80

31.54 103.333

31.55 83.3333

31.56 76.6667

31.57 70

31.58 70

31.59 76.6667

31.6 80

31.61 53.3333

31.62 66.6667

31.63 103.333

31.64 96.6667

31.65 86.6667

31.66 93.3333

31.67 113.333

31.68 93.3333

31.69 63.3333

31.7 100

31.71 93.3333

31.72 96.6667

31.73 103.333

31.74 90

31.75 53.3333

31.76 90

31.77 83.3333

31.78 56.6667

31.79 100

31.8 90

31.81 66.6667

31.82 93.3333

31.83 60

31.84 50

31.85 66.6667

31.86 63.3333

31.87 96.6667

31.88 53.3333

31.89 86.6667

31.9 93.3333

31.91 70

31.92 86.6667

31.93 63.3333

31.94 83.3333

31.95 93.3333

31.96 76.6667

31.97 86.6667

31.98 83.3333

31.99 100

32 116.667

32.01 73.3333

32.02 90

32.03 56.6667

32.04 53.3333

32.05 86.6667

32.06 93.3333

32.07 96.6667

32.08 70

32.09 90

32.1 80

32.11 53.3333

32.12 70

32.13 53.3333

32.14 73.3333

32.15 63.3333

32.16 100

32.17 53.3333

32.18 103.333

32.19 116.667

32.2 96.6667

32.21 60

32.22 66.6667

32.23 86.6667

32.24 100

32.25 103.333

32.26 96.6667

32.27 66.6667

32.28 90

32.29 86.6667

32.3 40

32.31 86.6667

32.32 70

32.33 56.6667

32.34 76.6667

32.35 53.3333

32.36 73.3333

32.37 83.3333

32.38 76.6667

32.39 56.6667

32.4 56.6667

32.41 76.6667

32.42 76.6667

32.43 93.3333

32.44 113.333

32.45 70

32.46 76.6667

32.47 56.6667

32.48 83.3333

32.49 73.3333

32.5 63.3333

32.51 90

32.52 63.3333

32.53 53.3333

32.54 100

32.55 73.3333

32.56 76.6667

32.57 70

32.58 56.6667

32.59 73.3333

32.6 83.3333

32.61 80

32.62 70

32.63 76.6667

32.64 63.3333

32.65 83.3333

32.66 60

32.67 96.6667

32.68 46.6667

32.69 73.3333

32.7 73.3333

32.71 56.6667

32.72 63.3333

32.73 63.3333

32.74 93.3333

32.75 76.6667

32.76 60

32.77 66.6667

32.78 56.6667

32.79 76.6667

32.8 130

32.81 116.667

32.82 96.6667

32.83 83.3333

32.84 40

32.85 100

32.86 90

32.87 70

32.88 60

32.89 76.6667

32.9 56.6667

32.91 63.3333

32.92 93.3333

32.93 96.6667

32.94 73.3333

32.95 70

32.96 83.3333

32.97 66.6667

32.98 96.6667

32.99 70

33 90

33.01 80

33.02 53.3333

33.03 90

33.04 60

33.05 56.6667

33.06 100

33.07 83.3333

33.08 73.3333

33.09 86.6667

33.1 63.3333

33.11 66.6667

33.12 80

33.13 103.333

33.14 86.6667

33.15 96.6667

33.16 76.6667

33.17 73.3333

33.18 73.3333

33.19 96.6667

33.2 80

33.21 110

33.22 60

33.23 46.6667

33.24 90

33.25 73.3333

33.26 80

33.27 93.3333

33.28 53.3333

33.29 53.3333

33.3 56.6667

33.31 70

33.32 66.6667

33.33 90

33.34 63.3333

33.35 60

33.36 56.6667

33.37 70

33.38 93.3333

33.39 66.6667

33.4 90

33.41 90

33.42 80

33.43 86.6667

33.44 50

33.45 46.6667

33.46 90

33.47 40

33.48 90

33.49 90

33.5 80

33.51 80

33.52 100

33.53 106.667

33.54 60

33.55 76.6667

33.56 90

33.57 56.6667

33.58 66.6667

33.59 63.3333

33.6 66.6667

33.61 70

33.62 83.3333

33.63 83.3333

33.64 53.3333

33.65 76.6667

33.66 63.3333

33.67 70

33.68 66.6667

33.69 63.3333

33.7 70

33.71 56.6667

33.72 43.3333

33.73 56.6667

33.74 53.3333

33.75 83.3333

33.76 76.6667

33.77 116.667

33.78 70

33.79 53.3333

33.8 70

33.81 53.3333

33.82 73.3333

33.83 76.6667

33.84 70

33.85 70

33.86 103.333

33.87 76.6667

33.88 56.6667

33.89 66.6667

33.9 73.3333

33.91 86.6667

33.92 93.3333

33.93 110

33.94 76.6667

33.95 70

33.96 60

33.97 83.3333

33.98 90

33.99 76.6667

34 70

34.01 76.6667

34.02 73.3333

34.03 73.3333

34.04 66.6667

34.05 53.3333

34.06 70

34.07 36.6667

34.08 66.6667

34.09 80

34.1 73.3333

34.11 70

34.12 46.6667

34.13 83.3333

34.14 63.3333

34.15 56.6667

34.16 50

34.17 80

34.18 53.3333

34.19 56.6667

34.2 56.6667

34.21 60

34.22 93.3333

34.23 50

34.24 73.3333

34.25 56.6667

34.26 73.3333

34.27 53.3333

34.28 83.3333

34.29 53.3333

34.3 63.3333

34.31 90

34.32 80

34.33 63.3333

34.34 60

34.35 76.6667

34.36 83.3333

34.37 76.6667

34.38 33.3333

34.39 56.6667

34.4 70

34.41 83.3333

34.42 70

34.43 93.3333

34.44 86.6667

34.45 56.6667

34.46 53.3333

34.47 70

34.48 80

34.49 73.3333

34.5 50

34.51 70

34.52 60

34.53 66.6667

34.54 50

34.55 90

34.56 63.3333

34.57 66.6667

34.58 56.6667

34.59 73.3333

34.6 60

34.61 60

34.62 63.3333

34.63 66.6667

34.64 80

34.65 100

34.66 53.3333

34.67 66.6667

34.68 66.6667

34.69 73.3333

34.7 46.6667

34.71 56.6667

34.72 90

34.73 56.6667

34.74 96.6667

34.75 43.3333

34.76 60

34.77 60

34.78 63.3333

34.79 46.6667

34.8 60

34.81 80

34.82 66.6667

34.83 80

34.84 86.6667

34.85 73.3333

34.86 86.6667

34.87 43.3333

34.88 50

34.89 53.3333

34.9 80

34.91 63.3333

34.92 66.6667

34.93 43.3333

34.94 50

34.95 50

34.96 60

34.97 60

34.98 63.3333

34.99 70

35 63.3333

35.01 63.3333

35.02 66.6667

35.03 70

35.04 53.3333

35.05 56.6667

35.06 60

35.07 43.3333

35.08 66.6667

35.09 40

35.1 86.6667

35.11 76.6667

35.12 56.6667

35.13 63.3333

35.14 66.6667

35.15 76.6667

35.16 63.3333

35.17 100

35.18 90

35.19 63.3333

35.2 90

35.21 70

35.22 60

35.23 60

35.24 83.3333

35.25 76.6667

35.26 66.6667

35.27 63.3333

35.28 56.6667

35.29 90

35.3 50

35.31 76.6667

35.32 83.3333

35.33 70

35.34 36.6667

35.35 60

35.36 46.6667

35.37 53.3333

35.38 53.3333

35.39 63.3333

35.4 36.6667

35.41 46.6667

35.42 60

35.43 76.6667

35.44 63.3333

35.45 53.3333

35.46 50

35.47 46.6667

35.48 90

35.49 56.6667

35.5 53.3333

35.51 43.3333

35.52 60

35.53 63.3333

35.54 50

35.55 83.3333

35.56 86.6667

35.57 56.6667

35.58 73.3333

35.59 60

35.6 43.3333

35.61 83.3333

35.62 40

35.63 60

35.64 83.3333

35.65 66.6667

35.66 43.3333

35.67 60

35.68 66.6667

35.69 60

35.7 53.3333

35.71 63.3333

35.72 60

35.73 60

35.74 43.3333

35.75 73.3333

35.76 80

35.77 86.6667

35.78 63.3333

35.79 50

35.8 46.6667

35.81 73.3333

35.82 53.3333

35.83 43.3333

35.84 70

35.85 76.6667

35.86 80

35.87 90

35.88 50

35.89 56.6667

35.9 70

35.91 66.6667

35.92 40

35.93 70

35.94 83.3333

35.95 60

35.96 60

35.97 36.6667

35.98 43.3333

35.99 50

36 46.6667

36.01 76.6667

36.02 70

36.03 86.6667

36.04 40

36.05 63.3333

36.06 63.3333

36.07 56.6667

36.08 43.3333

36.09 50

36.1 26.6667

36.11 60

36.12 86.6667

36.13 50

36.14 46.6667

36.15 36.6667

36.16 53.3333

36.17 56.6667

36.18 56.6667

36.19 73.3333

36.2 43.3333

36.21 33.3333

36.22 43.3333

36.23 60

36.24 43.3333

36.25 80

36.26 36.6667

36.27 53.3333

36.28 66.6667

36.29 56.6667

36.3 46.6667

36.31 46.6667

36.32 66.6667

36.33 63.3333

36.34 96.6667

36.35 46.6667

36.36 50

36.37 76.6667

36.38 73.3333

36.39 50

36.4 70

36.41 53.3333

36.42 70

36.43 43.3333

36.44 43.3333

36.45 73.3333

36.46 46.6667

36.47 43.3333

36.48 50

36.49 56.6667

36.5 60

36.51 70

36.52 50

36.53 53.3333

36.54 43.3333

36.55 36.6667

36.56 53.3333

36.57 50

36.58 43.3333

36.59 56.6667

36.6 70

36.61 30

36.62 46.6667

36.63 50

36.64 40

36.65 56.6667

36.66 43.3333

36.67 43.3333

36.68 60

36.69 46.6667

36.7 66.6667

36.71 60

36.72 56.6667

36.73 70

36.74 73.3333

36.75 43.3333

36.76 43.3333

36.77 16.6667

36.78 66.6667

36.79 53.3333

36.8 53.3333

36.81 46.6667

36.82 50

36.83 30

36.84 60

36.85 56.6667

36.86 40

36.87 46.6667

36.88 40

36.89 36.6667

36.9 40

36.91 60

36.92 53.3333

36.93 60

36.94 50

36.95 63.3333

36.96 53.3333

36.97 46.6667

36.98 53.3333

36.99 53.3333

37 56.6667

37.01 70

37.02 36.6667

37.03 66.6667

37.04 56.6667

37.05 36.6667

37.06 63.3333

37.07 90

37.08 70

37.09 63.3333

37.1 53.3333

37.11 50

37.12 50

37.13 73.3333

37.14 40

37.15 36.6667

37.16 60

37.17 63.3333

37.18 53.3333

37.19 50

37.2 66.6667

37.21 43.3333

37.22 53.3333

37.23 53.3333

37.24 56.6667

37.25 46.6667

37.26 63.3333

37.27 56.6667

37.28 56.6667

37.29 60

37.3 26.6667

37.31 53.3333

37.32 30

37.33 80

37.34 46.6667

37.35 60

37.36 43.3333

37.37 60

37.38 60

37.39 36.6667

37.4 36.6667

37.41 83.3333

37.42 46.6667

37.43 40

37.44 46.6667

37.45 50

37.46 53.3333

37.47 43.3333

37.48 30

37.49 66.6667

37.5 30

37.51 66.6667

37.52 56.6667

37.53 60

37.54 53.3333

37.55 40

37.56 63.3333

37.57 60

37.58 46.6667

37.59 63.3333

37.6 40

37.61 46.6667

37.62 63.3333

37.63 43.3333

37.64 53.3333

37.65 73.3333

37.66 53.3333

37.67 53.3333

37.68 30

37.69 36.6667

37.7 66.6667

37.71 33.3333

37.72 66.6667

37.73 30

37.74 63.3333

37.75 76.6667

37.76 60

37.77 46.6667

37.78 70

37.79 53.3333

37.8 40

37.81 33.3333

37.82 63.3333

37.83 46.6667

37.84 36.6667

37.85 40

37.86 90

37.87 40

37.88 50

37.89 50

37.9 60

37.91 43.3333

37.92 50

37.93 83.3333

37.94 60

37.95 40

37.96 33.3333

37.97 83.3333

37.98 46.6667

37.99 40

38 63.3333

38.01 36.6667

38.02 70

38.03 66.6667

38.04 46.6667

38.05 70

38.06 50

38.07 30

38.08 46.6667

38.09 30

38.1 13.3333

38.11 76.6667

38.12 50

38.13 73.3333

38.14 36.6667

38.15 63.3333

38.16 50

38.17 26.6667

38.18 50

38.19 36.6667

38.2 46.6667

38.21 66.6667

38.22 36.6667

38.23 46.6667

38.24 26.6667

38.25 46.6667

38.26 56.6667

38.27 43.3333

38.28 40

38.29 76.6667

38.3 50

38.31 43.3333

38.32 43.3333

38.33 50

38.34 36.6667

38.35 23.3333

38.36 46.6667

38.37 30

38.38 43.3333

38.39 50

38.4 50

38.41 56.6667

38.42 60

38.43 50

38.44 70

38.45 56.6667

38.46 30

38.47 40

38.48 60

38.49 40

38.5 36.6667

38.51 56.6667

38.52 20

38.53 40

38.54 50

38.55 56.6667

38.56 36.6667

38.57 43.3333

38.58 36.6667

38.59 43.3333

38.6 43.3333

38.61 43.3333

38.62 66.6667

38.63 53.3333

38.64 40

38.65 33.3333

38.66 43.3333

38.67 33.3333

38.68 53.3333

38.69 40

38.7 26.6667

38.71 40

38.72 73.3333

38.73 36.6667

38.74 60

38.75 63.3333

38.76 46.6667

38.77 40

38.78 76.6667

38.79 56.6667

38.8 46.6667

38.81 66.6667

38.82 40

38.83 43.3333

38.84 36.6667

38.85 30

38.86 36.6667

38.87 66.6667

38.88 50

38.89 56.6667

38.9 36.6667

38.91 33.3333

38.92 60

38.93 53.3333

38.94 60

38.95 43.3333

38.96 33.3333

38.97 46.6667

38.98 70

38.99 36.6667

39 40

39.01 53.3333

39.02 40

39.03 23.3333

39.04 43.3333

39.05 36.6667

39.06 46.6667

39.07 40

39.08 53.3333

39.09 40

39.1 40

39.11 60

39.12 40

39.13 30

39.14 46.6667

39.15 60

39.16 30

39.17 53.3333

39.18 23.3333

39.19 36.6667

39.2 56.6667

39.21 43.3333

39.22 43.3333

39.23 56.6667

39.24 60

39.25 50

39.26 16.6667

39.27 43.3333

39.28 56.6667

39.29 33.3333

39.3 60

39.31 46.6667

39.32 23.3333

39.33 30

39.34 43.3333

39.35 56.6667

39.36 26.6667

39.37 33.3333

39.38 56.6667

39.39 33.3333

39.4 46.6667

39.41 40

39.42 53.3333

39.43 43.3333

39.44 33.3333

39.45 30

39.46 36.6667

39.47 36.6667

39.48 50

39.49 30

39.5 26.6667

39.51 50

39.52 40

39.53 40

39.54 30

39.55 33.3333

39.56 33.3333

39.57 50

39.58 43.3333

39.59 43.3333

39.6 46.6667

39.61 66.6667

39.62 60

39.63 53.3333

39.64 30

39.65 33.3333

39.66 40

39.67 26.6667

39.68 43.3333

39.69 46.6667

39.7 46.6667

39.71 53.3333

39.72 30

39.73 30

39.74 50

39.75 50

39.76 63.3333

39.77 50

39.78 66.6667

39.79 46.6667

39.8 46.6667

39.81 70

39.82 50

39.83 53.3333

39.84 56.6667

39.85 63.3333

39.86 16.6667

39.87 76.6667

39.88 50

39.89 60

39.9 43.3333

39.91 56.6667

39.92 50

39.93 36.6667

39.94 36.6667

39.95 36.6667

39.96 33.3333

39.97 50

39.98 23.3333

39.99 20

40 53.3333

40.01 63.3333

40.02 23.3333

40.03 40

40.04 33.3333

40.05 20

40.06 50

40.07 30

40.08 46.6667

40.09 23.3333

40.1 30

40.11 43.3333

40.12 43.3333

40.13 33.3333

40.14 23.3333

40.15 40

40.16 40

40.17 63.3333

40.18 50

40.19 33.3333

40.2 66.6667

40.21 36.6667

40.22 33.3333

40.23 40

40.24 36.6667

40.25 43.3333

40.26 56.6667

40.27 46.6667

40.28 26.6667

40.29 33.3333

40.3 23.3333

40.31 43.3333

40.32 63.3333

40.33 33.3333

40.34 26.6667

40.35 60

40.36 36.6667

40.37 33.3333

40.38 20

40.39 33.3333

40.4 60

40.41 33.3333

40.42 60

40.43 40

40.44 33.3333

40.45 60

40.46 33.3333

40.47 33.3333

40.48 50

40.49 56.6667

40.5 60

40.51 26.6667

40.52 50

40.53 23.3333

40.54 33.3333

40.55 30

40.56 36.6667

40.57 40

40.58 30

40.59 53.3333

40.6 43.3333

40.61 26.6667

40.62 53.3333

40.63 76.6667

40.64 53.3333

40.65 13.3333

40.66 46.6667

40.67 43.3333

40.68 46.6667

40.69 30

40.7 26.6667

40.71 33.3333

40.72 26.6667

40.73 30

40.74 30

40.75 46.6667

40.76 23.3333

40.77 40

40.78 46.6667

40.79 53.3333

40.8 16.6667

40.81 40

40.82 66.6667

40.83 36.6667

40.84 53.3333

40.85 46.6667

40.86 56.6667

40.87 30

40.88 70

40.89 33.3333

40.9 53.3333

40.91 36.6667

40.92 46.6667

40.93 40

40.94 33.3333

40.95 33.3333

40.96 43.3333

40.97 40

40.98 50

40.99 40

41 43.3333

41.01 50

41.02 36.6667

41.03 43.3333

41.04 30

41.05 60

41.06 66.6667

41.07 33.3333

41.08 20

41.09 23.3333

41.1 56.6667

41.11 46.6667

41.12 56.6667

41.13 33.3333

41.14 43.3333

41.15 33.3333

41.16 43.3333

41.17 66.6667

41.18 13.3333

41.19 26.6667

41.2 50

41.21 50

41.22 50

41.23 50

41.24 53.3333

41.25 23.3333

41.26 33.3333

41.27 33.3333

41.28 40

41.29 33.3333

41.3 30

41.31 30

41.32 33.3333

41.33 23.3333

41.34 36.6667

41.35 40

41.36 33.3333

41.37 36.6667

41.38 33.3333

41.39 36.6667

41.4 50

41.41 36.6667

41.42 46.6667

41.43 33.3333

41.44 40

41.45 50

41.46 46.6667

41.47 63.3333

41.48 23.3333

41.49 43.3333

41.5 40

41.51 23.3333

41.52 30

41.53 33.3333

41.54 70

41.55 40

41.56 26.6667

41.57 60

41.58 33.3333

41.59 33.3333

41.6 43.3333

41.61 36.6667

41.62 36.6667

41.63 33.3333

41.64 53.3333

41.65 70

41.66 43.3333

41.67 43.3333

41.68 46.6667

41.69 26.6667

41.7 50

41.71 26.6667

41.72 40

41.73 50

41.74 36.6667

41.75 30

41.76 43.3333

41.77 30

41.78 43.3333

41.79 26.6667

41.8 70

41.81 40

41.82 26.6667

41.83 36.6667

41.84 40

41.85 46.6667

41.86 50

41.87 26.6667

41.88 70

41.89 33.3333

41.9 26.6667

41.91 63.3333

41.92 43.3333

41.93 56.6667

41.94 53.3333

41.95 50

41.96 43.3333

41.97 56.6667

41.98 33.3333

41.99 36.6667

42 36.6667

42.01 26.6667

42.02 26.6667

42.03 33.3333

42.04 56.6667

42.05 36.6667

42.06 36.6667

42.07 40

42.08 26.6667

42.09 40

42.1 23.3333

42.11 43.3333

42.12 36.6667

42.13 30

42.14 30

42.15 46.6667

42.16 53.3333

42.17 40

42.18 23.3333

42.19 36.6667

42.2 40

42.21 30

42.22 30

42.23 43.3333

42.24 36.6667

42.25 36.6667

42.26 36.6667

42.27 43.3333

42.28 40

42.29 33.3333

42.3 20

42.31 46.6667

42.32 56.6667

42.33 50

42.34 46.6667

42.35 33.3333

42.36 63.3333

42.37 46.6667

42.38 23.3333

42.39 43.3333

42.4 30

42.41 36.6667

42.42 33.3333

42.43 23.3333

42.44 53.3333

42.45 26.6667

42.46 36.6667

42.47 60

42.48 60

42.49 33.3333

42.5 36.6667

42.51 30

42.52 60

42.53 70

42.54 23.3333

42.55 43.3333

42.56 53.3333

42.57 26.6667

42.58 53.3333

42.59 33.3333

42.6 33.3333

42.61 40

42.62 36.6667

42.63 23.3333

42.64 23.3333

42.65 40

42.66 60

42.67 60

42.68 33.3333

42.69 36.6667

42.7 40

42.71 46.6667

42.72 56.6667

42.73 60

42.74 63.3333

42.75 23.3333

42.76 60

42.77 40

42.78 56.6667

42.79 36.6667

42.8 50

42.81 53.3333

42.82 26.6667

42.83 80

42.84 23.3333

42.85 43.3333

42.86 60

42.87 30

42.88 36.6667

42.89 46.6667

42.9 33.3333

42.91 56.6667

42.92 53.3333

42.93 36.6667

42.94 23.3333

42.95 36.6667

42.96 40

42.97 26.6667

42.98 36.6667

42.99 30

43 50

43.01 30

43.02 60

43.03 46.6667

43.04 30

43.05 23.3333

43.06 40

43.07 50

43.08 40

43.09 56.6667

43.1 16.6667

43.11 56.6667

43.12 40

43.13 26.6667

43.14 46.6667

43.15 30

43.16 36.6667

43.17 46.6667

43.18 30

43.19 53.3333

43.2 40

43.21 53.3333

43.22 53.3333

43.23 46.6667

43.24 33.3333

43.25 40

43.26 43.3333

43.27 43.3333

43.28 30

43.29 36.6667

43.3 53.3333

43.31 50

43.32 60

43.33 56.6667

43.34 40

43.35 60

43.36 36.6667

43.37 56.6667

43.38 43.3333

43.39 60

43.4 50

43.41 43.3333

43.42 60

43.43 36.6667

43.44 63.3333

43.45 63.3333

43.46 60

43.47 60

43.48 66.6667

43.49 60

43.5 66.6667

43.51 66.6667

43.52 50

43.53 70

43.54 50

43.55 46.6667

43.56 50

43.57 36.6667

43.58 40

43.59 53.3333

43.6 43.3333

43.61 46.6667

43.62 106.667

43.63 40

43.64 56.6667

43.65 56.6667

43.66 33.3333

43.67 53.3333

43.68 56.6667

43.69 50

43.7 53.3333

43.71 60

43.72 60

43.73 43.3333

43.74 90

43.75 53.3333

43.76 43.3333

43.77 56.6667

43.78 50

43.79 50

43.8 56.6667

43.81 56.6667

43.82 73.3333

43.83 80

43.84 90

43.85 66.6667

43.86 40

43.87 46.6667

43.88 56.6667

43.89 63.3333

43.9 60

43.91 63.3333

43.92 66.6667

43.93 63.3333

43.94 46.6667

43.95 66.6667

43.96 60

43.97 66.6667

43.98 60

43.99 66.6667

44 46.6667

44.01 63.3333

44.02 73.3333

44.03 86.6667

44.04 76.6667

44.05 50

44.06 60

44.07 70

44.08 90

44.09 46.6667

44.1 50

44.11 56.6667

44.12 93.3333

44.13 76.6667

44.14 96.6667

44.15 96.6667

44.16 26.6667

44.17 70

44.18 76.6667

44.19 60

44.2 60

44.21 116.667

44.22 66.6667

44.23 60

44.24 70

44.25 73.3333

44.26 43.3333

44.27 63.3333

44.28 96.6667

44.29 106.667

44.3 70

44.31 96.6667

44.32 60

44.33 90

44.34 110

44.35 70

44.36 106.667

44.37 80

44.38 93.3333

44.39 96.6667

44.4 126.667

44.41 83.3333

44.42 110

44.43 160

44.44 126.667

44.45 96.6667

44.46 143.333

44.47 140

44.48 170

44.49 180

44.5 193.333

44.51 206.667

44.52 196.667

44.53 266.667

44.54 303.333

44.55 336.667

44.56 326.667

44.57 346.667

44.58 483.333

44.59 576.667

44.6 636.667

44.61 926.667

44.62 1093.33

44.63 1340

44.64 1636.67

44.65 2096.67

44.66 2340

44.67 2770

44.68 2840

44.69 3113.33

44.7 2863.33

44.71 2930

44.72 2336.67

44.73 2020

44.74 1833.33

44.75 1566.67

44.76 1450

44.77 1303.33

44.78 1396.67

44.79 1466.67

44.8 1506.67

44.81 1360

44.82 1303.33

44.83 1123.33

44.84 1033.33

44.85 850

44.86 546.667

44.87 486.667

44.88 373.333

44.89 306.667

44.9 266.667

44.91 193.333

44.92 183.333

44.93 133.333

44.94 156.667

44.95 140

44.96 143.333

44.97 96.6667

44.98 100

44.99 83.3333

45 93.3333

45.01 100

45.02 86.6667

45.03 76.6667

45.04 96.6667

45.05 103.333

45.06 46.6667

45.07 43.3333

45.08 100

45.09 70

45.1 83.3333

45.11 40

45.12 53.3333

45.13 93.3333

45.14 66.6667

45.15 73.3333

45.16 53.3333

45.17 50

45.18 76.6667

45.19 66.6667

45.2 33.3333

45.21 50

45.22 36.6667

45.23 50

45.24 56.6667

45.25 63.3333

45.26 43.3333

45.27 30

45.28 40

45.29 46.6667

45.3 36.6667

45.31 36.6667

45.32 40

45.33 50

45.34 60

45.35 56.6667

45.36 30

45.37 30

45.38 63.3333

45.39 36.6667

45.4 46.6667

45.41 50

45.42 46.6667

45.43 53.3333

45.44 60

45.45 70

45.46 43.3333

45.47 30

45.48 46.6667

45.49 40

45.5 50

45.51 46.6667

45.52 33.3333

45.53 30

45.54 43.3333

45.55 40

45.56 36.6667

45.57 40

45.58 53.3333

45.59 33.3333

45.6 36.6667

45.61 33.3333

45.62 56.6667

45.63 53.3333

45.64 53.3333

45.65 26.6667

45.66 60

45.67 46.6667

45.68 70

45.69 40

45.7 63.3333

45.71 30

45.72 60

45.73 83.3333

45.74 36.6667

45.75 40

45.76 43.3333

45.77 23.3333

45.78 43.3333

45.79 26.6667

45.8 46.6667

45.81 33.3333

45.82 40

45.83 30

45.84 36.6667

45.85 30

45.86 33.3333

45.87 40

45.88 40

45.89 23.3333

45.9 36.6667

45.91 30

45.92 50

45.93 43.3333

45.94 43.3333

45.95 46.6667

45.96 46.6667

45.97 40

45.98 43.3333

45.99 26.6667

46 30

46.01 20

46.02 30

46.03 23.3333

46.04 40

46.05 33.3333

46.06 53.3333

46.07 36.6667

46.08 30

46.09 53.3333

46.1 66.6667

46.11 36.6667

46.12 46.6667

46.13 30

46.14 46.6667

46.15 23.3333

46.16 40

46.17 50

46.18 46.6667

46.19 30

46.2 36.6667

46.21 26.6667

46.22 30

46.23 46.6667

46.24 30

46.25 46.6667

46.26 33.3333

46.27 43.3333

46.28 56.6667

46.29 10

46.3 36.6667

46.31 26.6667

46.32 33.3333

46.33 36.6667

46.34 36.6667

46.35 53.3333

46.36 43.3333

46.37 23.3333

46.38 33.3333

46.39 50

46.4 20

46.41 40

46.42 23.3333

46.43 36.6667

46.44 33.3333

46.45 23.3333

46.46 46.6667

46.47 46.6667

46.48 30

46.49 66.6667

46.5 33.3333

46.51 36.6667

46.52 53.3333

46.53 46.6667

46.54 26.6667

46.55 50

46.56 36.6667

46.57 46.6667

46.58 26.6667

46.59 50

46.6 23.3333

46.61 33.3333

46.62 26.6667

46.63 30

46.64 36.6667

46.65 53.3333

46.66 56.6667

46.67 36.6667

46.68 46.6667

46.69 46.6667

46.7 56.6667

46.71 53.3333

46.72 40

46.73 36.6667

46.74 33.3333

46.75 26.6667

46.76 33.3333

46.77 63.3333

46.78 50

46.79 23.3333

46.8 40

46.81 50

46.82 40

46.83 40

46.84 40

46.85 30

46.86 23.3333

46.87 36.6667

46.88 26.6667

46.89 20

46.9 26.6667

46.91 30

46.92 20

46.93 30

46.94 23.3333

46.95 70

46.96 33.3333

46.97 23.3333

46.98 30

46.99 20

47 23.3333

47.01 43.3333

47.02 30

47.03 26.6667

47.04 63.3333

47.05 23.3333

47.06 20

47.07 36.6667

47.08 33.3333

47.09 40

47.1 53.3333

47.11 26.6667

47.12 20

47.13 40

47.14 16.6667

47.15 20

47.16 53.3333

47.17 36.6667

47.18 36.6667

47.19 36.6667

47.2 30

47.21 23.3333

47.22 30

47.23 50

47.24 43.3333

47.25 33.3333

47.26 30

47.27 43.3333

47.28 33.3333

47.29 33.3333

47.3 30

47.31 40

47.32 36.6667

47.33 43.3333

47.34 36.6667

47.35 36.6667

47.36 36.6667

47.37 36.6667

47.38 43.3333

47.39 26.6667

47.4 36.6667

47.41 40

47.42 43.3333

47.43 50

47.44 33.3333

47.45 30

47.46 43.3333

47.47 53.3333

47.48 33.3333

47.49 36.6667

47.5 33.3333

47.51 33.3333

47.52 36.6667

47.53 53.3333

47.54 40

47.55 53.3333

47.56 40

47.57 36.6667

47.58 30

47.59 46.6667

47.6 33.3333

47.61 20

47.62 43.3333

47.63 30

47.64 30

47.65 40

47.66 40

47.67 40

47.68 20

47.69 43.3333

47.7 43.3333

47.71 20

47.72 13.3333

47.73 33.3333

47.74 50

47.75 43.3333

47.76 33.3333

47.77 33.3333

47.78 26.6667

47.79 26.6667

47.8 36.6667

47.81 33.3333

47.82 36.6667

47.83 46.6667

47.84 36.6667

47.85 33.3333

47.86 46.6667

47.87 53.3333

47.88 33.3333

47.89 40

47.9 50

47.91 40

47.92 30

47.93 40

47.94 30

47.95 30

47.96 33.3333

47.97 23.3333

47.98 50

47.99 33.3333

48 46.6667

48.01 33.3333

48.02 23.3333

48.03 23.3333

48.04 36.6667

48.05 50

48.06 23.3333

48.07 23.3333

48.08 30

48.09 36.6667

48.1 50

48.11 20

48.12 40

48.13 13.3333

48.14 26.6667

48.15 20

48.16 43.3333

48.17 33.3333

48.18 23.3333

48.19 36.6667

48.2 50

48.21 46.6667

48.22 50

48.23 40

48.24 36.6667

48.25 53.3333

48.26 20

48.27 26.6667

48.28 23.3333

48.29 46.6667

48.3 33.3333

48.31 30

48.32 30

48.33 23.3333

48.34 40

48.35 53.3333

48.36 43.3333

48.37 23.3333

48.38 36.6667

48.39 33.3333

48.4 33.3333

48.41 40

48.42 46.6667

48.43 26.6667

48.44 36.6667

48.45 30

48.46 40

48.47 43.3333

48.48 36.6667

48.49 40

48.5 26.6667

48.51 33.3333

48.52 13.3333

48.53 23.3333

48.54 46.6667

48.55 43.3333

48.56 43.3333

48.57 30

48.58 26.6667

48.59 36.6667

48.6 43.3333

48.61 40

48.62 36.6667

48.63 40

48.64 36.6667

48.65 36.6667

48.66 53.3333

48.67 30

48.68 23.3333

48.69 36.6667

48.7 10

48.71 33.3333

48.72 66.6667

48.73 30

48.74 36.6667

48.75 50

48.76 36.6667

48.77 36.6667

48.78 26.6667

48.79 40

48.8 46.6667

48.81 50

48.82 40

48.83 40

48.84 33.3333

48.85 43.3333

48.86 56.6667

48.87 46.6667

48.88 43.3333

48.89 23.3333

48.9 36.6667

48.91 46.6667

48.92 33.3333

48.93 33.3333

48.94 30

48.95 33.3333

48.96 36.6667

48.97 36.6667

48.98 43.3333

48.99 46.6667

49 50

49.01 33.3333

49.02 26.6667

49.03 23.3333

49.04 33.3333

49.05 43.3333

49.06 33.3333

49.07 30

49.08 16.6667

49.09 36.6667

49.1 23.3333

49.11 50

49.12 40

49.13 36.6667

49.14 30

49.15 36.6667

49.16 36.6667

49.17 40

49.18 23.3333

49.19 40

49.2 33.3333

49.21 30

49.22 26.6667

49.23 50

49.24 13.3333

49.25 33.3333

49.26 36.6667

49.27 36.6667

49.28 33.3333

49.29 36.6667

49.3 36.6667

49.31 36.6667

49.32 40

49.33 26.6667

49.34 26.6667

49.35 26.6667

49.36 43.3333

49.37 36.6667

49.38 33.3333

49.39 36.6667

49.4 20

49.41 46.6667

49.42 33.3333

49.43 20

49.44 30

49.45 33.3333

49.46 16.6667

49.47 36.6667

49.48 26.6667

49.49 56.6667

49.5 26.6667

49.51 53.3333

49.52 46.6667

49.53 23.3333

49.54 26.6667

49.55 30

49.56 23.3333

49.57 56.6667

49.58 26.6667

49.59 33.3333

49.6 36.6667

49.61 36.6667

49.62 30

49.63 40

49.64 53.3333

49.65 40

49.66 36.6667

49.67 40

49.68 53.3333

49.69 33.3333

49.7 53.3333

49.71 20

49.72 30

49.73 26.6667

49.74 40

49.75 26.6667

49.76 30

49.77 40

49.78 30

49.79 26.6667

49.8 33.3333

49.81 23.3333

49.82 16.6667

49.83 23.3333

49.84 23.3333

49.85 46.6667

49.86 33.3333

49.87 43.3333

49.88 50

49.89 23.3333

49.9 36.6667

49.91 50

49.92 23.3333

49.93 36.6667

49.94 40

49.95 33.3333

49.96 26.6667

49.97 43.3333

49.98 43.3333

49.99 16.6667

50 56.6667

50.01 33.3333

50.02 30

50.03 33.3333

50.04 56.6667

50.05 43.3333

50.06 26.6667

50.07 50

50.08 16.6667

50.09 43.3333

50.1 26.6667

50.11 40

50.12 30

50.13 40

50.14 26.6667

50.15 30

50.16 30

50.17 16.6667

50.18 36.6667

50.19 36.6667

50.2 43.3333

50.21 23.3333

50.22 33.3333

50.23 30

50.24 43.3333

50.25 43.3333

50.26 73.3333

50.27 36.6667

50.28 46.6667

50.29 43.3333

50.3 33.3333

50.31 43.3333

50.32 33.3333

50.33 60

50.34 33.3333

50.35 23.3333

50.36 56.6667

50.37 26.6667

50.38 30

50.39 26.6667

50.4 30

50.41 30

50.42 46.6667

50.43 63.3333

50.44 20

50.45 26.6667

50.46 20

50.47 60

50.48 10

50.49 43.3333

50.5 40

50.51 33.3333

50.52 43.3333

50.53 33.3333

50.54 40

50.55 36.6667

50.56 23.3333

50.57 43.3333

50.58 60

50.59 26.6667

50.6 43.3333

50.61 30

50.62 36.6667

50.63 50

50.64 26.6667

50.65 16.6667

50.66 30

50.67 30

50.68 23.3333

50.69 40

50.7 16.6667

50.71 40

50.72 20

50.73 33.3333

50.74 50

50.75 40

50.76 20

50.77 33.3333

50.78 36.6667

50.79 43.3333

50.8 46.6667

50.81 10

50.82 36.6667

50.83 43.3333

50.84 23.3333

50.85 43.3333

50.86 43.3333

50.87 46.6667

50.88 30

50.89 33.3333

50.9 43.3333

50.91 30

50.92 20

50.93 26.6667

50.94 30

50.95 40

50.96 33.3333

50.97 33.3333

50.98 23.3333

50.99 26.6667

51 26.6667

51.01 33.3333

51.02 33.3333

51.03 43.3333

51.04 36.6667

51.05 23.3333

51.06 20

51.07 13.3333

51.08 26.6667

51.09 40

51.1 33.3333

51.11 23.3333

51.12 36.6667

51.13 23.3333

51.14 46.6667

51.15 40

51.16 33.3333

51.17 36.6667

51.18 20

51.19 33.3333

51.2 36.6667

51.21 43.3333

51.22 26.6667

51.23 36.6667

51.24 43.3333

51.25 26.6667

51.26 36.6667

51.27 26.6667

51.28 33.3333

51.29 33.3333

51.3 43.3333

51.31 16.6667

51.32 43.3333

51.33 36.6667

51.34 43.3333

51.35 33.3333

51.36 53.3333

51.37 23.3333

51.38 23.3333

51.39 30

51.4 50

51.41 40

51.42 36.6667

51.43 40

51.44 50

51.45 33.3333

51.46 20

51.47 20

51.48 46.6667

51.49 20

51.5 40

51.51 60

51.52 43.3333

51.53 20

51.54 30

51.55 40

51.56 26.6667

51.57 26.6667

51.58 26.6667

51.59 40

51.6 36.6667

51.61 43.3333

51.62 30

51.63 46.6667

51.64 26.6667

51.65 33.3333

51.66 20

51.67 16.6667

51.68 33.3333

51.69 56.6667

51.7 33.3333

51.71 33.3333

51.72 46.6667

51.73 36.6667

51.74 53.3333

51.75 20

51.76 16.6667

51.77 30

51.78 6.66667

51.79 26.6667

51.8 20

51.81 46.6667

51.82 26.6667

51.83 23.3333

51.84 23.3333

51.85 16.6667

51.86 30

51.87 16.6667

51.88 36.6667

51.89 16.6667

51.9 26.6667

51.91 26.6667

51.92 36.6667

51.93 26.6667

51.94 13.3333

51.95 23.3333

51.96 13.3333

51.97 30

51.98 10

51.99 36.6667

52 16.6667

52.01 40

52.02 36.6667

52.03 36.6667

52.04 46.6667

52.05 26.6667

52.06 16.6667

52.07 30

52.08 26.6667

52.09 26.6667

52.1 50

52.11 36.6667

52.12 43.3333

52.13 26.6667

52.14 53.3333

52.15 36.6667

52.16 43.3333

52.17 36.6667

52.18 30

52.19 46.6667

52.2 30

52.21 46.6667

52.22 36.6667

52.23 50

52.24 26.6667

52.25 46.6667

52.26 23.3333

52.27 30

52.28 26.6667

52.29 46.6667

52.3 36.6667

52.31 36.6667

52.32 23.3333

52.33 23.3333

52.34 26.6667

52.35 43.3333

52.36 33.3333

52.37 30

52.38 50

52.39 30

52.4 26.6667

52.41 20

52.42 43.3333

52.43 30

52.44 56.6667

52.45 50

52.46 33.3333

52.47 53.3333

52.48 23.3333

52.49 43.3333

52.5 33.3333

52.51 36.6667

52.52 40

52.53 43.3333

52.54 26.6667

52.55 46.6667

52.56 33.3333

52.57 50

52.58 40

52.59 23.3333

52.6 33.3333

52.61 26.6667

52.62 33.3333

52.63 46.6667

52.64 33.3333

52.65 33.3333

52.66 26.6667

52.67 30

52.68 33.3333

52.69 20

52.7 26.6667

52.71 30

52.72 26.6667

52.73 26.6667

52.74 33.3333

52.75 43.3333

52.76 33.3333

52.77 30

52.78 33.3333

52.79 33.3333

52.8 23.3333

52.81 23.3333

52.82 40

52.83 23.3333

52.84 43.3333

52.85 23.3333

52.86 33.3333

52.87 33.3333

52.88 20

52.89 26.6667

52.9 46.6667

52.91 46.6667

52.92 30

52.93 56.6667

52.94 30

52.95 23.3333

52.96 26.6667

52.97 50

52.98 20

52.99 50

53 33.3333

53.01 33.3333

53.02 26.6667

53.03 23.3333

53.04 20

53.05 20

53.06 40

53.07 30

53.08 26.6667

53.09 16.6667

53.1 46.6667

53.11 40

53.12 53.3333

53.13 26.6667

53.14 13.3333

53.15 26.6667

53.16 30

53.17 43.3333

53.18 16.6667

53.19 33.3333

53.2 36.6667

53.21 13.3333

53.22 33.3333

53.23 23.3333

53.24 40

53.25 33.3333

53.26 30

53.27 36.6667

53.28 43.3333

53.29 26.6667

53.3 46.6667

53.31 43.3333

53.32 23.3333

53.33 23.3333

53.34 36.6667

53.35 20

53.36 26.6667

53.37 23.3333

53.38 36.6667

53.39 36.6667

53.4 30

53.41 43.3333

53.42 13.3333

53.43 33.3333

53.44 40

53.45 26.6667

53.46 26.6667

53.47 33.3333

53.48 30

53.49 26.6667

53.5 36.6667

53.51 36.6667

53.52 36.6667

53.53 36.6667

53.54 26.6667

53.55 33.3333

53.56 33.3333

53.57 43.3333

53.58 33.3333

53.59 26.6667

53.6 26.6667

53.61 33.3333

53.62 13.3333

53.63 16.6667

53.64 30

53.65 43.3333

53.66 26.6667

53.67 46.6667

53.68 46.6667

53.69 23.3333

53.7 36.6667

53.71 46.6667

53.72 10

53.73 36.6667

53.74 36.6667

53.75 30

53.76 30

53.77 30

53.78 33.3333

53.79 36.6667

53.8 23.3333

53.81 16.6667

53.82 40

53.83 33.3333

53.84 30

53.85 23.3333

53.86 20

53.87 30

53.88 46.6667

53.89 46.6667

53.9 30

53.91 26.6667

53.92 40

53.93 20

53.94 13.3333

53.95 50

53.96 16.6667

53.97 43.3333

53.98 40

53.99 26.6667

54 33.3333

54.01 33.3333

54.02 20

54.03 53.3333

54.04 46.6667

54.05 20

54.06 36.6667

54.07 13.3333

54.08 36.6667

54.09 33.3333

54.1 30

54.11 30

54.12 33.3333

54.13 33.3333

54.14 26.6667

54.15 13.3333

54.16 43.3333

54.17 26.6667

54.18 36.6667

54.19 16.6667

54.2 16.6667

54.21 36.6667

54.22 16.6667

54.23 46.6667

54.24 16.6667

54.25 43.3333

54.26 43.3333

54.27 30

54.28 23.3333

54.29 33.3333

54.3 26.6667

54.31 36.6667

54.32 33.3333

54.33 33.3333

54.34 60

54.35 36.6667

54.36 33.3333

54.37 20

54.38 33.3333

54.39 26.6667

54.4 23.3333

54.41 33.3333

54.42 36.6667

54.43 20

54.44 20

54.45 46.6667

54.46 50

54.47 43.3333

54.48 30

54.49 60

54.5 36.6667

54.51 30

54.52 16.6667

54.53 30

54.54 23.3333

54.55 40

54.56 36.6667

54.57 33.3333

54.58 56.6667

54.59 36.6667

54.6 26.6667

54.61 26.6667

54.62 36.6667

54.63 33.3333

54.64 33.3333

54.65 30

54.66 50

54.67 20

54.68 26.6667

54.69 26.6667

54.7 13.3333

54.71 46.6667

54.72 43.3333

54.73 23.3333

54.74 40

54.75 23.3333

54.76 30

54.77 30

54.78 36.6667

54.79 30

54.8 30

54.81 50

54.82 40

54.83 40

54.84 10

54.85 20

54.86 33.3333

54.87 40

54.88 26.6667

54.89 23.3333

54.9 23.3333

54.91 43.3333

54.92 26.6667

54.93 33.3333

54.94 30

54.95 53.3333

54.96 40

54.97 33.3333

54.98 26.6667

54.99 3.33333

55 23.3333

55.01 23.3333

55.02 16.6667

55.03 46.6667

55.04 40

55.05 46.6667

55.06 23.3333

55.07 36.6667

55.08 26.6667

55.09 46.6667

55.1 56.6667

55.11 43.3333

55.12 43.3333

55.13 40

55.14 23.3333

55.15 23.3333

55.16 13.3333

55.17 33.3333

55.18 36.6667

55.19 43.3333

55.2 23.3333

55.21 43.3333

55.22 23.3333

55.23 40

55.24 13.3333

55.25 63.3333

55.26 40

55.27 43.3333

55.28 20

55.29 36.6667

55.3 30

55.31 43.3333

55.32 30

55.33 46.6667

55.34 20

55.35 43.3333

55.36 23.3333

55.37 20

55.38 36.6667

55.39 40

55.4 30

55.41 20

55.42 40

55.43 43.3333

55.44 30

55.45 36.6667

55.46 26.6667

55.47 30

55.48 26.6667

55.49 23.3333

55.5 23.3333

55.51 43.3333

55.52 33.3333

55.53 43.3333

55.54 36.6667

55.55 36.6667

55.56 43.3333

55.57 46.6667

55.58 6.66667

55.59 33.3333

55.6 33.3333

55.61 30

55.62 46.6667

55.63 33.3333

55.64 46.6667

55.65 20

55.66 30

55.67 26.6667

55.68 30

55.69 46.6667

55.7 46.6667

55.71 23.3333

55.72 20

55.73 33.3333

55.74 20

55.75 23.3333

55.76 46.6667

55.77 40

55.78 33.3333

55.79 23.3333

55.8 23.3333

55.81 30

55.82 53.3333

55.83 43.3333

55.84 33.3333

55.85 30

55.86 33.3333

55.87 43.3333

55.88 13.3333

55.89 26.6667

55.9 43.3333

55.91 33.3333

55.92 43.3333

55.93 43.3333

55.94 46.6667

55.95 46.6667

55.96 26.6667

55.97 30

55.98 13.3333

55.99 23.3333

56 13.3333

56.01 13.3333

56.02 60

56.03 40

56.04 23.3333

56.05 13.3333

56.06 46.6667

56.07 46.6667

56.08 16.6667

56.09 23.3333

56.1 26.6667

56.11 43.3333

56.12 36.6667

56.13 40

56.14 43.3333

56.15 20

56.16 36.6667

56.17 20

56.18 40

56.19 26.6667

56.2 53.3333

56.21 43.3333

56.22 46.6667

56.23 13.3333

56.24 40

56.25 33.3333

56.26 40

56.27 53.3333

56.28 43.3333

56.29 23.3333

56.3 40

56.31 40

56.32 26.6667

56.33 3.33333

56.34 30

56.35 33.3333

56.36 26.6667

56.37 46.6667

56.38 30

56.39 53.3333

56.4 23.3333

56.41 26.6667

56.42 26.6667

56.43 26.6667

56.44 26.6667

56.45 23.3333

56.46 20

56.47 43.3333

56.48 43.3333

56.49 26.6667

56.5 30

56.51 16.6667

56.52 33.3333

56.53 26.6667

56.54 30

56.55 40

56.56 40

56.57 20

56.58 43.3333

56.59 46.6667

56.6 26.6667

56.61 33.3333

56.62 26.6667

56.63 10

56.64 26.6667

56.65 50

56.66 26.6667

56.67 23.3333

56.68 23.3333

56.69 23.3333

56.7 23.3333

56.71 20

56.72 46.6667

56.73 36.6667

56.74 33.3333

56.75 40

56.76 16.6667

56.77 33.3333

56.78 30

56.79 46.6667

56.8 13.3333

56.81 36.6667

56.82 30

56.83 40

56.84 26.6667

56.85 36.6667

56.86 43.3333

56.87 13.3333

56.88 20

56.89 23.3333

56.9 20

56.91 13.3333

56.92 36.6667

56.93 33.3333

56.94 30

56.95 26.6667

56.96 26.6667

56.97 26.6667

56.98 40

56.99 23.3333

57 16.6667

57.01 26.6667

57.02 33.3333

57.03 16.6667

57.04 26.6667

57.05 36.6667

57.06 30

57.07 20

57.08 30

57.09 43.3333

57.1 53.3333

57.11 26.6667

57.12 30

57.13 26.6667

57.14 50

57.15 50

57.16 40

57.17 36.6667

57.18 16.6667

57.19 26.6667

57.2 40

57.21 23.3333

57.22 33.3333

57.23 30

57.24 33.3333

57.25 26.6667

57.26 13.3333

57.27 40

57.28 40

57.29 36.6667

57.3 20

57.31 23.3333

57.32 33.3333

57.33 50

57.34 43.3333

57.35 40

57.36 40

57.37 33.3333

57.38 36.6667

57.39 30

57.4 43.3333

57.41 13.3333

57.42 43.3333

57.43 46.6667

57.44 53.3333

57.45 30

57.46 40

57.47 33.3333

57.48 60

57.49 60

57.5 46.6667

57.51 46.6667

57.52 36.6667

57.53 23.3333

57.54 36.6667

57.55 33.3333

57.56 26.6667

57.57 53.3333

57.58 33.3333

57.59 33.3333

57.6 40

57.61 36.6667

57.62 46.6667

57.63 60

57.64 16.6667

57.65 33.3333

57.66 36.6667

57.67 23.3333

57.68 23.3333

57.69 30

57.7 36.6667

57.71 33.3333

57.72 30

57.73 43.3333

57.74 23.3333

57.75 16.6667

57.76 20

57.77 46.6667

57.78 6.66667

57.79 33.3333

57.8 33.3333

57.81 23.3333

57.82 33.3333

57.83 46.6667

57.84 36.6667

57.85 66.6667

57.86 30

57.87 23.3333

57.88 46.6667

57.89 33.3333

57.9 26.6667

57.91 43.3333

57.92 30

57.93 33.3333

57.94 46.6667

57.95 33.3333

57.96 36.6667

57.97 30

57.98 26.6667

57.99 70

58 33.3333

58.01 33.3333

58.02 36.6667

58.03 53.3333

58.04 40

58.05 30

58.06 63.3333

58.07 30

58.08 26.6667

58.09 23.3333

58.1 70

58.11 23.3333

58.12 36.6667

58.13 36.6667

58.14 30

58.15 36.6667

58.16 40

58.17 16.6667

58.18 26.6667

58.19 46.6667

58.2 40

58.21 30

58.22 43.3333

58.23 36.6667

58.24 43.3333

58.25 30

58.26 33.3333

58.27 33.3333

58.28 50

58.29 23.3333

58.3 26.6667

58.31 36.6667

58.32 30

58.33 40

58.34 30

58.35 40

58.36 23.3333

58.37 26.6667

58.38 40

58.39 36.6667

58.4 36.6667

58.41 40

58.42 56.6667

58.43 26.6667

58.44 40

58.45 30

58.46 26.6667

58.47 36.6667

58.48 20

58.49 23.3333

58.5 16.6667

58.51 23.3333

58.52 30

58.53 43.3333

58.54 43.3333

58.55 33.3333

58.56 30

58.57 43.3333

58.58 36.6667

58.59 40

58.6 26.6667

58.61 36.6667

58.62 30

58.63 43.3333

58.64 36.6667

58.65 40

58.66 53.3333

58.67 56.6667

58.68 23.3333

58.69 30

58.7 26.6667

58.71 36.6667

58.72 33.3333

58.73 26.6667

58.74 26.6667

58.75 63.3333

58.76 43.3333

58.77 26.6667

58.78 33.3333

58.79 56.6667

58.8 30

58.81 36.6667

58.82 53.3333

58.83 26.6667

58.84 43.3333

58.85 43.3333

58.86 26.6667

58.87 26.6667

58.88 60

58.89 40

58.9 56.6667

58.91 63.3333

58.92 20

58.93 30

58.94 23.3333

58.95 60

58.96 16.6667

58.97 23.3333

58.98 33.3333

58.99 43.3333

59 30

59.01 16.6667

59.02 33.3333

59.03 33.3333

59.04 23.3333

59.05 36.6667

59.06 30

59.07 26.6667

59.08 33.3333

59.09 46.6667

59.1 23.3333

59.11 33.3333

59.12 36.6667

59.13 40

59.14 46.6667

59.15 16.6667

59.16 33.3333

59.17 20

59.18 26.6667

59.19 40

59.2 13.3333

59.21 23.3333

59.22 40

59.23 30

59.24 50

59.25 30

59.26 33.3333

59.27 20

59.28 20

59.29 33.3333

59.3 33.3333

59.31 33.3333

59.32 36.6667

59.33 43.3333

59.34 40

59.35 10

59.36 33.3333

59.37 40

59.38 50

59.39 43.3333

59.4 43.3333

59.41 16.6667

59.42 43.3333

59.43 36.6667

59.44 26.6667

59.45 26.6667

59.46 53.3333

59.47 30

59.48 26.6667

59.49 43.3333

59.5 30

59.51 33.3333

59.52 43.3333

59.53 30

59.54 40

59.55 26.6667

59.56 30

59.57 60

59.58 26.6667

59.59 20

59.6 40

59.61 33.3333

59.62 66.6667

59.63 33.3333

59.64 23.3333

59.65 40

59.66 36.6667

59.67 30

59.68 23.3333

59.69 16.6667

59.7 30

59.71 33.3333

59.72 53.3333

59.73 33.3333

59.74 50

59.75 63.3333

59.76 43.3333

59.77 46.6667

59.78 40

59.79 50

59.8 30

59.81 36.6667

59.82 33.3333

59.83 40

59.84 36.6667

59.85 36.6667

59.86 43.3333

59.87 40

59.88 26.6667

59.89 36.6667

59.9 26.6667

59.91 40

59.92 26.6667

59.93 30

59.94 16.6667

59.95 40

59.96 26.6667

59.97 33.3333

59.98 33.3333

59.99 33.3333

60 36.6667

60.01 30

60.02 23.3333

60.03 46.6667

60.04 33.3333

60.05 10

60.06 23.3333

60.07 43.3333

60.08 23.3333

60.09 13.3333

60.1 40

60.11 40

60.12 23.3333

60.13 26.6667

60.14 30

60.15 33.3333

60.16 50

60.17 26.6667

60.18 26.6667

60.19 13.3333

60.2 50

60.21 40

60.22 46.6667

60.23 40

60.24 46.6667

60.25 33.3333

60.26 40

60.27 33.3333

60.28 33.3333

60.29 36.6667

60.3 30

60.31 30

60.32 16.6667

60.33 16.6667

60.34 50

60.35 20

60.36 30

60.37 30

60.38 30

60.39 40

60.4 30

60.41 36.6667

60.42 30

60.43 40

60.44 46.6667

60.45 40

60.46 46.6667

60.47 43.3333

60.48 33.3333

60.49 36.6667

60.5 16.6667

60.51 30

60.52 26.6667

60.53 13.3333

60.54 30

60.55 10

60.56 46.6667

60.57 26.6667

60.58 30

60.59 33.3333

60.6 56.6667

60.61 46.6667

60.62 43.3333

60.63 33.3333

60.64 16.6667

60.65 30

60.66 56.6667

60.67 36.6667

60.68 30

60.69 36.6667

60.7 16.6667

60.71 36.6667

60.72 33.3333

60.73 36.6667

60.74 30

60.75 46.6667

60.76 36.6667

60.77 36.6667

60.78 40

60.79 30

60.8 43.3333

60.81 40

60.82 26.6667

60.83 33.3333

60.84 26.6667

60.85 23.3333

60.86 33.3333

60.87 36.6667

60.88 33.3333

60.89 26.6667

60.9 23.3333

60.91 43.3333

60.92 40

60.93 20

60.94 43.3333

60.95 36.6667

60.96 26.6667

60.97 30

60.98 40

60.99 26.6667

61 36.6667

61.01 36.6667

61.02 43.3333

61.03 33.3333

61.04 43.3333

61.05 53.3333

61.06 23.3333

61.07 20

61.08 40

61.09 36.6667

61.1 36.6667

61.11 60

61.12 33.3333

61.13 26.6667

61.14 33.3333

61.15 36.6667

61.16 26.6667

61.17 30

61.18 40

61.19 46.6667

61.2 30

61.21 53.3333

61.22 43.3333

61.23 23.3333

61.24 36.6667

61.25 43.3333

61.26 30

61.27 33.3333

61.28 50

61.29 33.3333

61.3 40

61.31 43.3333

61.32 36.6667

61.33 36.6667

61.34 60

61.35 56.6667

61.36 30

61.37 50

61.38 30

61.39 50

61.4 43.3333

61.41 26.6667

61.42 33.3333

61.43 43.3333

61.44 36.6667

61.45 56.6667

61.46 30

61.47 50

61.48 33.3333

61.49 26.6667

61.5 33.3333

61.51 23.3333

61.52 30

61.53 50

61.54 36.6667

61.55 33.3333

61.56 50

61.57 40

61.58 33.3333

61.59 30

61.6 56.6667

61.61 30

61.62 43.3333

61.63 46.6667

61.64 63.3333

61.65 20

61.66 30

61.67 20

61.68 33.3333

61.69 26.6667

61.7 26.6667

61.71 26.6667

61.72 23.3333

61.73 16.6667

61.74 43.3333

61.75 46.6667

61.76 30

61.77 43.3333

61.78 40

61.79 30

61.8 20

61.81 36.6667

61.82 26.6667

61.83 36.6667

61.84 33.3333

61.85 46.6667

61.86 36.6667

61.87 43.3333

61.88 33.3333

61.89 40

61.9 50

61.91 33.3333

61.92 16.6667

61.93 36.6667

61.94 46.6667

61.95 30

61.96 53.3333

61.97 20

61.98 26.6667

61.99 36.6667

62 46.6667

62.01 40

62.02 26.6667

62.03 43.3333

62.04 26.6667

62.05 40

62.06 33.3333

62.07 43.3333

62.08 40

62.09 60

62.1 40

62.11 30

62.12 46.6667

62.13 43.3333

62.14 23.3333

62.15 46.6667

62.16 43.3333

62.17 43.3333

62.18 40

62.19 20

62.2 33.3333

62.21 16.6667

62.22 40

62.23 23.3333

62.24 26.6667

62.25 26.6667

62.26 40

62.27 33.3333

62.28 36.6667

62.29 33.3333

62.3 40

62.31 26.6667

62.32 56.6667

62.33 40

62.34 40

62.35 30

62.36 30

62.37 40

62.38 60

62.39 43.3333

62.4 40

62.41 23.3333

62.42 36.6667

62.43 23.3333

62.44 36.6667

62.45 20

62.46 36.6667

62.47 73.3333

62.48 30

62.49 66.6667

62.5 50

62.51 33.3333

62.52 50

62.53 20

62.54 43.3333

62.55 23.3333

62.56 56.6667

62.57 23.3333

62.58 26.6667

62.59 26.6667

62.6 56.6667

62.61 26.6667

62.62 50

62.63 33.3333

62.64 63.3333

62.65 36.6667

62.66 26.6667

62.67 40

62.68 43.3333

62.69 36.6667

62.7 50

62.71 46.6667

62.72 36.6667

62.73 36.6667

62.74 66.6667

62.75 30

62.76 30

62.77 20

62.78 40

62.79 43.3333

62.8 36.6667

62.81 33.3333

62.82 40

62.83 46.6667

62.84 30

62.85 50

62.86 30

62.87 16.6667

62.88 13.3333

62.89 23.3333

62.9 43.3333

62.91 33.3333

62.92 46.6667

62.93 20

62.94 30

62.95 16.6667

62.96 36.6667

62.97 66.6667

62.98 16.6667

62.99 30

63 33.3333

63.01 10

63.02 20

63.03 50

63.04 60

63.05 23.3333

63.06 46.6667

63.07 46.6667

63.08 26.6667

63.09 46.6667

63.1 30

63.11 26.6667

63.12 43.3333

63.13 30

63.14 26.6667

63.15 36.6667

63.16 33.3333

63.17 36.6667

63.18 30

63.19 40

63.2 70

63.21 20

63.22 40

63.23 43.3333

63.24 40

63.25 30

63.26 66.6667

63.27 46.6667

63.28 43.3333

63.29 33.3333

63.3 36.6667

63.31 50

63.32 40

63.33 33.3333

63.34 43.3333

63.35 26.6667

63.36 46.6667

63.37 40

63.38 43.3333

63.39 20

63.4 23.3333

63.41 33.3333

63.42 20

63.43 23.3333

63.44 36.6667

63.45 40

63.46 36.6667

63.47 43.3333

63.48 43.3333

63.49 30

63.5 30

63.51 30

63.52 43.3333

63.53 20

63.54 46.6667

63.55 50

63.56 36.6667

63.57 43.3333

63.58 40

63.59 50

63.6 20

63.61 26.6667

63.62 30

63.63 26.6667

63.64 30

63.65 56.6667

63.66 10

63.67 26.6667

63.68 30

63.69 36.6667

63.7 53.3333

63.71 46.6667

63.72 26.6667

63.73 40

63.74 30

63.75 26.6667

63.76 50

63.77 23.3333

63.78 56.6667

63.79 53.3333

63.8 60

63.81 46.6667

63.82 53.3333

63.83 40

63.84 53.3333

63.85 36.6667

63.86 40

63.87 56.6667

63.88 36.6667

63.89 23.3333

63.9 40

63.91 33.3333

63.92 60

63.93 20

63.94 33.3333

63.95 60

63.96 40

63.97 40

63.98 26.6667

63.99 43.3333

64 53.3333

64.01 30

64.02 50

64.03 53.3333

64.04 50

64.05 20

64.06 46.6667

64.07 36.6667

64.08 50

64.09 46.6667

64.1 33.3333

64.11 60

64.12 26.6667

64.13 36.6667

64.14 33.3333

64.15 33.3333

64.16 40

64.17 36.6667

64.18 33.3333

64.19 46.6667

64.2 26.6667

64.21 50

64.22 33.3333

64.23 30

64.24 46.6667

64.25 20

64.26 53.3333

64.27 40

64.28 26.6667

64.29 50

64.3 56.6667

64.31 33.3333

64.32 46.6667

64.33 30

64.34 43.3333

64.35 33.3333

64.36 50

64.37 16.6667

64.38 26.6667

64.39 53.3333

64.4 26.6667

64.41 46.6667

64.42 23.3333

64.43 46.6667

64.44 26.6667

64.45 36.6667

64.46 16.6667

64.47 53.3333

64.48 40

64.49 26.6667

64.5 23.3333

64.51 36.6667

64.52 43.3333

64.53 43.3333

64.54 16.6667

64.55 43.3333

64.56 26.6667

64.57 53.3333

64.58 43.3333

64.59 56.6667

64.6 23.3333

64.61 43.3333

64.62 46.6667

64.63 50

64.64 46.6667

64.65 46.6667

64.66 33.3333

64.67 60

64.68 43.3333

64.69 63.3333

64.7 66.6667

64.71 60

64.72 56.6667

64.73 50

64.74 63.3333

64.75 60

64.76 40

64.77 70

64.78 66.6667

64.79 36.6667

64.8 46.6667

64.81 63.3333

64.82 60

64.83 63.3333

64.84 56.6667

64.85 53.3333

64.86 53.3333

64.87 56.6667

64.88 46.6667

64.89 76.6667

64.9 86.6667

64.91 90

64.92 93.3333

64.93 116.667

64.94 110

64.95 113.333

64.96 150

64.97 200

64.98 243.333

64.99 293.333

65 340

65.01 446.667

65.02 433.333

65.03 413.333

65.04 490

65.05 516.667

65.06 390

65.07 430

65.08 346.667

65.09 280

65.1 240

65.11 216.667

65.12 180

65.13 163.333

65.14 116.667

65.15 126.667

65.16 186.667

65.17 170

65.18 136.667

65.19 226.667

65.2 196.667

65.21 220

65.22 220

65.23 253.333

65.24 176.667

65.25 193.333

65.26 166.667

65.27 160

65.28 133.333

65.29 120

65.3 93.3333

65.31 90

65.32 53.3333

65.33 86.6667

65.34 46.6667

65.35 73.3333

65.36 66.6667

65.37 53.3333

65.38 66.6667

65.39 50

65.4 36.6667

65.41 53.3333

65.42 63.3333

65.43 36.6667

65.44 40

65.45 43.3333

65.46 36.6667

65.47 43.3333

65.48 66.6667

65.49 43.3333

65.5 46.6667

65.51 86.6667

65.52 43.3333

65.53 36.6667

65.54 53.3333

65.55 36.6667

65.56 30

65.57 30

65.58 20

65.59 40

65.6 33.3333

65.61 40

65.62 43.3333

65.63 30

65.64 73.3333

65.65 30

65.66 26.6667

65.67 33.3333

65.68 30

65.69 40

65.7 46.6667

65.71 56.6667

65.72 33.3333

65.73 50

65.74 23.3333

65.75 33.3333

65.76 36.6667

65.77 43.3333

65.78 43.3333

65.79 40

65.8 50

65.81 46.6667

65.82 26.6667

65.83 40

65.84 46.6667

65.85 60

65.86 36.6667

65.87 33.3333

65.88 26.6667

65.89 46.6667

65.9 56.6667

65.91 53.3333

65.92 26.6667

65.93 36.6667

65.94 43.3333

65.95 56.6667

65.96 20

65.97 43.3333

65.98 40

65.99 43.3333

66 46.6667

66.01 36.6667

66.02 46.6667

66.03 43.3333

66.04 33.3333

66.05 43.3333

66.06 63.3333

66.07 30

66.08 33.3333

66.09 40

66.1 23.3333

66.11 53.3333

66.12 16.6667

66.13 36.6667

66.14 23.3333

66.15 23.3333

66.16 26.6667

66.17 26.6667

66.18 26.6667

66.19 53.3333

66.2 40

66.21 13.3333

66.22 40

66.23 56.6667

66.24 23.3333

66.25 50

66.26 46.6667

66.27 50

66.28 30

66.29 46.6667

66.3 43.3333

66.31 60

66.32 33.3333

66.33 30

66.34 36.6667

66.35 26.6667

66.36 40

66.37 26.6667

66.38 33.3333

66.39 40

66.4 33.3333

66.41 16.6667
[truncated: 139,021 more chars]
